# Potential causal association between gut microbiome and posttraumatic stress disorder

**DOI:** 10.1038/s41398-024-02765-7

**Published:** 2024-01-31

**Authors:** Qiang He, Wenjing Wang, Dingkang Xu, Yang Xiong, Chuanyuan Tao, Chao You, Lu Ma, Junpeng Ma

**Affiliations:** 1 Department of Neurosurgery, West China Hospital, Sichuan University, 37 Guoxue Lane, Wuhou District, Chengdu, 610041 Sichuan China; 2 Department of Pharmacy, Institute of Metabolic Diseases and Pharmacotherapy, West China Hospital, Sichuan University, 37 Guoxue Lane, Wuhou District, Chengdu, China; 3 Department of Neurosurgery, Beijing Hospital, National Center of Gerontology, Institute of Geriatric Medicine, Chinese Academy of Medical Sciences, Beijing, China; 4 Department of Urology, West China Hospital, Sichuan University, Chengdu, China

**Keywords:** Depression, Clinical genetics

## Abstract

**Background:**

The causal effects of gut microbiome and the development of posttraumatic stress disorder (PTSD) are still unknown. This study aimed to clarify their potential causal association using mendelian randomization (MR).

**Methods:**

The summary-level statistics for gut microbiome were retrieved from a genome-wide association study (GWAS) of the MiBioGen consortium. As to PTSD, the Freeze 2 datasets were originated from the Psychiatric Genomics Consortium Posttraumatic Stress Disorder Working Group (PGC-PTSD), and the replicated datasets were obtained from FinnGen consortium. Single nucleotide polymorphisms meeting MR assumptions were selected as instrumental variables. The inverse variance weighting (IVW) method was employed as the main approach, supplemented by sensitivity analyses to evaluate potential pleiotropy and heterogeneity and ensure the robustness of the MR results. We also performed reverse MR analyses to explore PTSD’s causal effects on the relative abundances of specific features of the gut microbiome.

**Results:**

In Freeze 2 datasets from PGC-PTSD, eight bacterial traits revealed a potential causal association between gut microbiome and PTSD (IVW, all *P* < 0.05). In addition, *Genus.Dorea* and *genus.Sellimonas* were replicated in FinnGen datasets, in which eight bacterial traits revealed a potential causal association between gut microbiome and the occurrence of PTSD. The heterogeneity and pleiotropy analyses further supported the robustness of the IVW findings, providing additional evidence for their reliability.

**Conclusion:**

Our study provides the potential causal impact of gut microbiomes on the development of PTSD, shedding new light on the understanding of the dysfunctional gut-brain axis in this disorder. Our findings present novel evidence and call for investigations to confirm the association between their links, as well as to illuminate the underlying mechanisms.

## Introduction

Posttraumatic stress disorder (PTSD) manifests as the reliving of traumatic experiences, avoidance of trauma triggers, and heightened states of arousal, significantly influencing cognition, mood, and physiologic state [1]. With nearly 70% of individuals having experienced at least one traumatic accident in their lifetime, and a reported lifetime rate of 5.6% among those exposed to trauma in 26 countries, it is evident that PTSD affects a substantial portion of the population, with many experiencing persistent symptoms [2]. This chronic condition severely compromises psychological functioning, often leading to additional comorbidities such as depression and anxiety [3, 4]. The resulting social and healthcare burden cannot be ignored. However, the specific factors that contribute to the development of PTSD in certain individuals while sparing others remain elusive [5]. Given the immense impact of PTSD on both health and the economy, it is imperative to explore innovative strategies for treatment and prevention.

The gut microbiome exerts a profound impact on the intricate interplay of the gut-brain axis. It plays a significant role in shaping bidirectional communication between the gastrointestinal system and the brain, orchestrating a delicate balance. On the one hand, the gut microbiome possesses the power to influence cognitive function, memory, and intricate patterns of behavior [6, 7]. Conversely, stress can disturb the composition and diversity of the gut microbiome, leading to an intricate cascade of events that affect stress reactivity and response [8]. Notably, alterations in the gut microbiome have been observed to elicit changes in key neurotransmitter systems within the central nervous system, encompassing plasticity-related mechanisms [9, 10], serotonergic singling pathways [11, 12], and GABAergic modulation [13, 14]. Intriguingly, the dysbiosis of the bacterial traits in gut has emerged as a significant factor in the development of PTSD [8, 15].

To date, the exploration of the relationship between gut microbiome and PTSD remains relatively limited, with few studies devoted to this fascinating connection. This dysbiosis appears to have enduring effects on the immune system and other physiological processes, rendering individuals more vulnerable to developing PTSD following exposure to traumatic events, ultimately contributing to the manifestation of the disorder [16]. In a pioneering clinical study spearheaded by Sian M.J. Hemmings and her esteemed colleagues, an intricate exploration was undertaken, encompassing 18 individuals afflicted with PTSD and 12 control participants who endured traumatic experiences. This endeavor unveiled an intriguing revelation: a discernible diminishment in the prevalence of three phyla, namely *Actinobacteria*, *Lentisphaerae*, and *Verrucomicrobia*, displayed a noteworthy association with the emergence of PTSD [15]. Additionally, investigations focusing on cirrhosis patients with and without PTSD uncovered intriguing results, indicating lower microbial diversity and high levels of pathobionts in patients in patients with PTSD [17]. Furthermore, an association existed between the presence of *Escherichia-Shigella* and impaired cognition in patients with PTSD. Within the realm of frontline healthcare workers (FHWs) directly exposed to the impact of the COVID-19 pandemic, a remarkable correlation surfaces profound alterations in the gut microbiome and stands as a significant determinant in the onset of PTSD, anxiety, depression, and sleep-related symptoms when contrasted against control groups. Astonishingly, the dysbiosis of alpha diversity of the gut microbiome exhibits a lingering persistence even after a six-month follow-up [18]. The alteration of the gut microbiomes was also observed in the preclinical PTSD model. Qin Zhou and his colleagues found that changes in *Firmicutes*, *Bacteroidetes*, *Cyanobacteria*, and *Proteobacteria* levels were most relevant to the exhibited fear-like and anxiety-like behaviors after a single prolonged stress (SPS) exposure compared to control groups in rats [19]. It is important to note that all these studies are mainly observational, subject to potential confounding factors such as retrospective research, the study design, and limited sample size, which may influence the interpretation of these findings. Further research is warranted to elucidate this captivating relationship more comprehensively.

Mendelian randomization (MR) serves as a powerful and robust approach to explore the causal effects of gut microbiome on PTSD, utilizing genetic variants known as single-nucleotide polymorphisms (SNPs) [20]. By capitalizing on the random assortment of SNPs during embryonic development, MR can minimize confounding factors, measurement errors, and reverse causation, providing more robust and reliable causal inferences [12, 21, 22]. Notably, previous MR studies have successfully established causal relationships between gut microbiome and various neuropsychiatric disorders, such as major depressive disorders [23], schizophrenia [24], autism spectrum disorder [24], and bipolar disorder [24], underscoring the profound influence of the gut-brain axis. Excitingly, advancements in gene-wide association study (GWAS) have enabled the availability of summary-level datasets examining the links between the gut microbiome and PTSD. Nevertheless, the potential causal association between gut microbiota and PTSD remains largely uncharted. Hence, this study seeks to unravel the potential causal association between specific bacterial taxa and PTSD through meticulous MR analyses, thereby enhancing our understanding of the intricate involvement of the gut-brain axis and providing novel insights for the treatment and prevention of PTSD.

## Methods

### The overall overview of this MR study

**Fig. 1:**
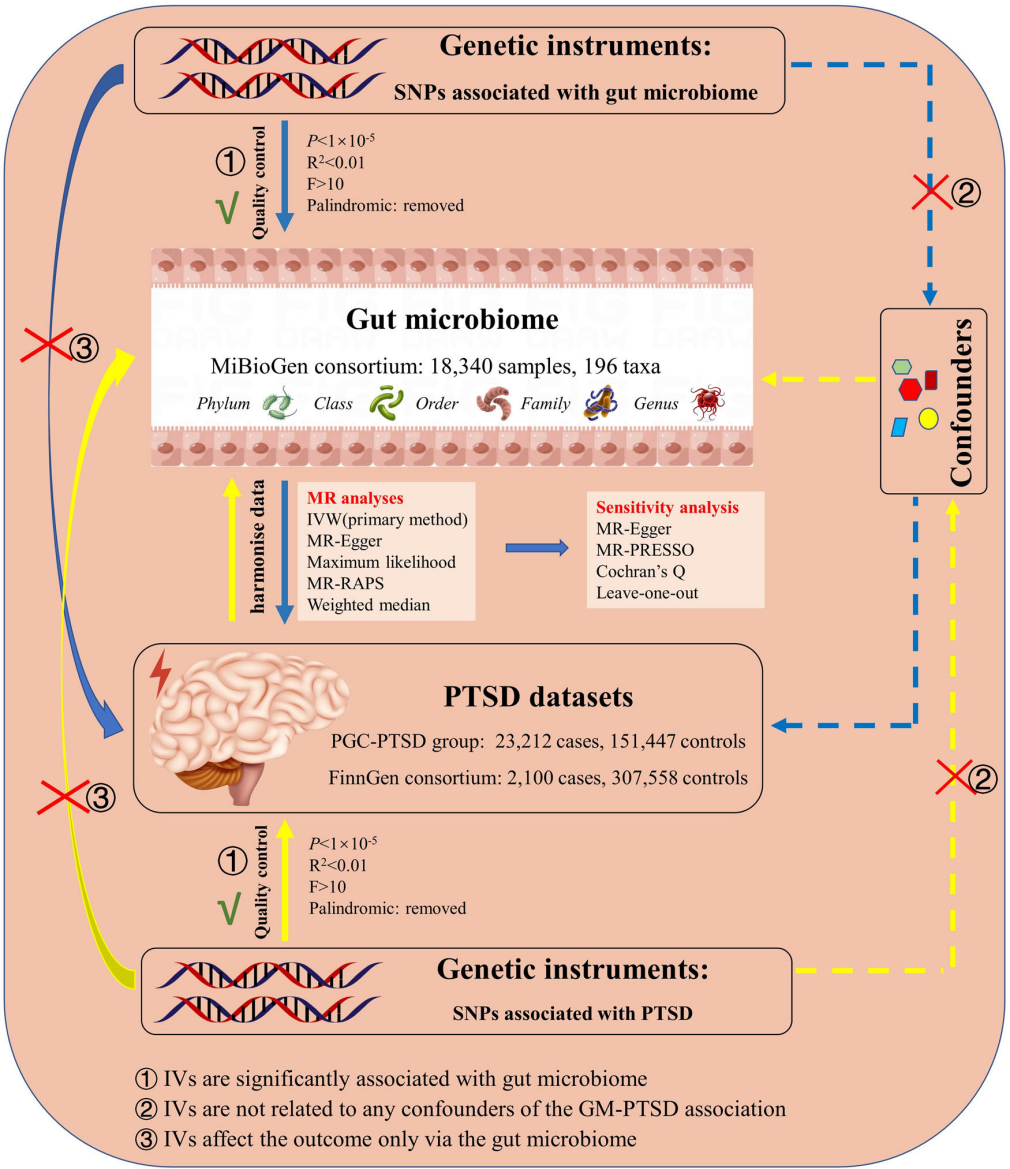
Study overview of the two-sample Mendelian randomization for the potential causal association between gut microbiome and PTSD. GM gut microbiome, PTSD post-traumatic stress disorder, SNP single nucleotide polymorphism, IV instrumental variables, IVW IVW, inverse variance weighted, RAPS robust adjusted profile score, MR-PRESSO MR Pleiotropy RESidual Sum and Outlier.

As depicted in Fig. 1, this MR study comprises two crucial components. In the first part, the MR analysis aimed to explore the causal relationship between gut microbiome and PTSD. In the reverse MR analysis, we calculated the causal estimates of PTSD on the composition of gut microbiome. To ensure the reliability of our findings, three important assumptions regarding the selected instrumental variables (IVs) have to be satisfied in the MR study: (1) IVs demonstrated significant associations with the exposure variable of interest; (2) IVs remained independent of any potential confounding factors that could influence the gut microbiome-PTSD association; (3) IVs solely affected the outcome through their effects on the exposure variable.

### Data sources of gut microbiome

The MiBioGen consortium has performed an extensive analysis, encompassing 24 cohorts and involving a remarkable 18,340 participants. This GWAS study ranks as the most expansive and comprehensive GWAS ever conducted on the gut microbiome, offering unprecedented insights into its intricacies [25]. Through the utilization of microbiome quantitative trait loci (mbQTL) mapping analysis, only taxa observed in at least 10% of the participants were included, resulting in a remarkable compilation of 211 distinct gut microbiomes. These microbiomes were further categorized into five hierarchical classifications, ranging from genus to phylum level, encompassing 131 genera, 16 classes, 35 families, 20 orders, and 9 phyla. The association of bacterial richness was utilized by multivariate linear regression analysis. To ensure the accuracy of the findings, crucial covariates, including sex, age, genetic principal components (PCs), and cohort-specific potential microbiome batch effects, were adjusted for across all cohorts [25]. For more detailed information about the gut microbiome, the website “https://mibiogen.gcc.rug.nl” provides a wealth of valuable resources and data.

### Data sources of PTSD

The summary-level datasets concerning PTSD were meticulously collected from two distinct sources. Firstly, the GWAS meta-analysis of the freeze 2 analysis was conducted by the esteemed Psychiatric Genomics Consortium Posttraumatic Stress Disorder (PGC-PTSD) Working Group [26]. This comprehensive study encompassed an assessment of PTSD based on lifetime and/or current PTSD status, employing the Diagnostic and Statistical Manual of Mental Disorders (Third Edition Revised, Fourth Edition, and Fifth Edition) as a reference. GWAS analysis was performed by linear regression with the first 10 principal components, age, and sex used as covariates. The detailed information is found in original article [26]. The PGC-PTSD dataset comprised a substantial sample size, consisting of 23,212 cases and 151,447 controls of European ancestry. Rigorous quality control procedures were implemented through the PGC pipeline, ensuring the reliability and validity of the findings.

In addition, the FinnGen consortium also offered the PTSD dataset, further enriching our understanding of this complex condition. This dataset comprised 2100 individuals diagnosed with PTSD, complemented by a robust control group comprising 307,558 individuals. These invaluable summary statistics from the FinnGen consortium were made available on December 1, 2022, and can be accessed through their official website (https://www.finngen.fi/en/access_results).

### Ethical approvement

The summary level of PTSD and gut microbiome datasets in this MR study were retracted from de-identified public data/studies. Ethical approval and informed consent were obtained by the ethics committee in original articles [25, 26]. Hence, ethical approval was thus exempted from our study.

### Genetic instrument selection

Initially, we conducted a rigorous filtering process to collect candidate single SNPs with a strong association with bacterial traits as IVs. This selection was carried out at a genome-wide significance level, meeting a *P*-value of less than 1 × 10^–5^. To mitigate the impact of linkage disequilibrium, we employed an r^2^ < 0.01 threshold and a clumping window of 10,000 kb based on the European population as a reference. These criteria enabled us to obtain independent SNPs as IVs. A comprehensive summary of the selected IVs can be found in Table S1. Additionally, we performed MR Pleiotropy RESidual Sum and Outlier (MR-PRESSO) analysis to identify significant SNPs that might exhibit pleiotropic effects [27]. Any significant outliers identified during this analysis were subsequently eliminated. It is important to note that no SNPs were excluded at this step. To assess the strength of our MR approach, we employed the F-statistics = (Bets/Se)^2^. If the F-statistics fell below 10, indicating insufficient strength, the corresponding SNP was not considered [28].

### Main statistical analyses

The primary approach employed for exploring potential causal associations was the inverse variance weighting (IVW) method, which involved translating MR estimates into a weighted regression that analyzed the effects of SNPs on the outcome concerning their effects on the exposure [29, 30]. The statistical significance level was defined as *P* < 0.05. All statistical analyses were performed using various R packages, including “TwoSampleMR,” “mr.raps,” “MRPRESSO,” “frostplot,” and “ggplot2,” within the R software (version 4.3.0, The R Foundation, Vienna, Austria).

### Sensitivity analyses

Sensitivity analyses were conducted to examine the potential causal association between the gut microbiome and PTSD, employing multiple methods such as MR-Egger, weighted median, maximum likelihood, MR robust adjusted profile score (MR-RAPS), and MR-PRESSO. In the MR-Egger analysis, the introduction of an intercept term allowed for the evaluation of the Instrument Strength Independent of the Direct Effect (InSIDE) assumption. A *P* value less than 0.05 indicated potential horizontal pleiotropy. The weighted median analysis corrected for the estimation of the causal effect when at least half of the IVs were invalid [31, 32]. Maximum likelihood estimation provided unbiased results under the assumptions of no heterogeneity and absence of horizontal pleiotropy [33]. The global test in MR-PRESSO was employed to assess overall horizontal pleiotropy and correct estimates by removing significant outliers [27]. MR-RAPS enhanced statistical power by accounting for weak instrumental variables [34]. We also performed Cochran’s *Q* test to explore the heterogeneity among variant-specific estimates. In addition, a leave-one-out analysis was conducted to verify the robustness of the conclusion.

## Results

### Genetic instrument variables for gut microbiome

In this step, a comprehensive assortment of 211 bacterial taxa was compiled following a careful genetic instrument selection process. However, due to the limited understanding of unknown bacterial traits, fifteen features were subsequently eliminated. As a result, a total of 196 distinct bacterial traits were specifically chosen for exploring their potential association with PTSD in this MR study. For each bacterial trait, a set of SNPs was meticulously employed as IVs. The detailed inventory of these SNPs can be found in Table S1.

### Causal effects of the genetically predicted gut microbiome on PTSD in Freeze 2 datasets in MR analysis

**Fig. 2:**
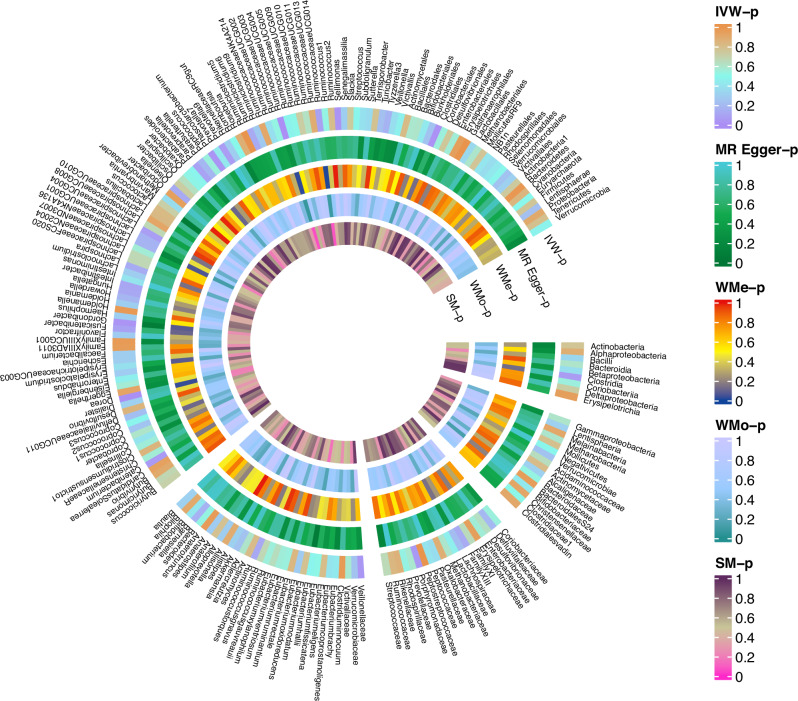
Causal effect of the gut microbiome on PTSD in Freeze 2 datasets based on MR analyses. From outside to inside, the *P* values of IVW, MR Egger, WMe, WMo, and SM are represented. PTSD post-traumatic stress disorder, IVW inverse variance weighted, WMe weighted median, WMo weighted mode, SM simple mode. MR mendelian randomization.

**Fig. 3:**
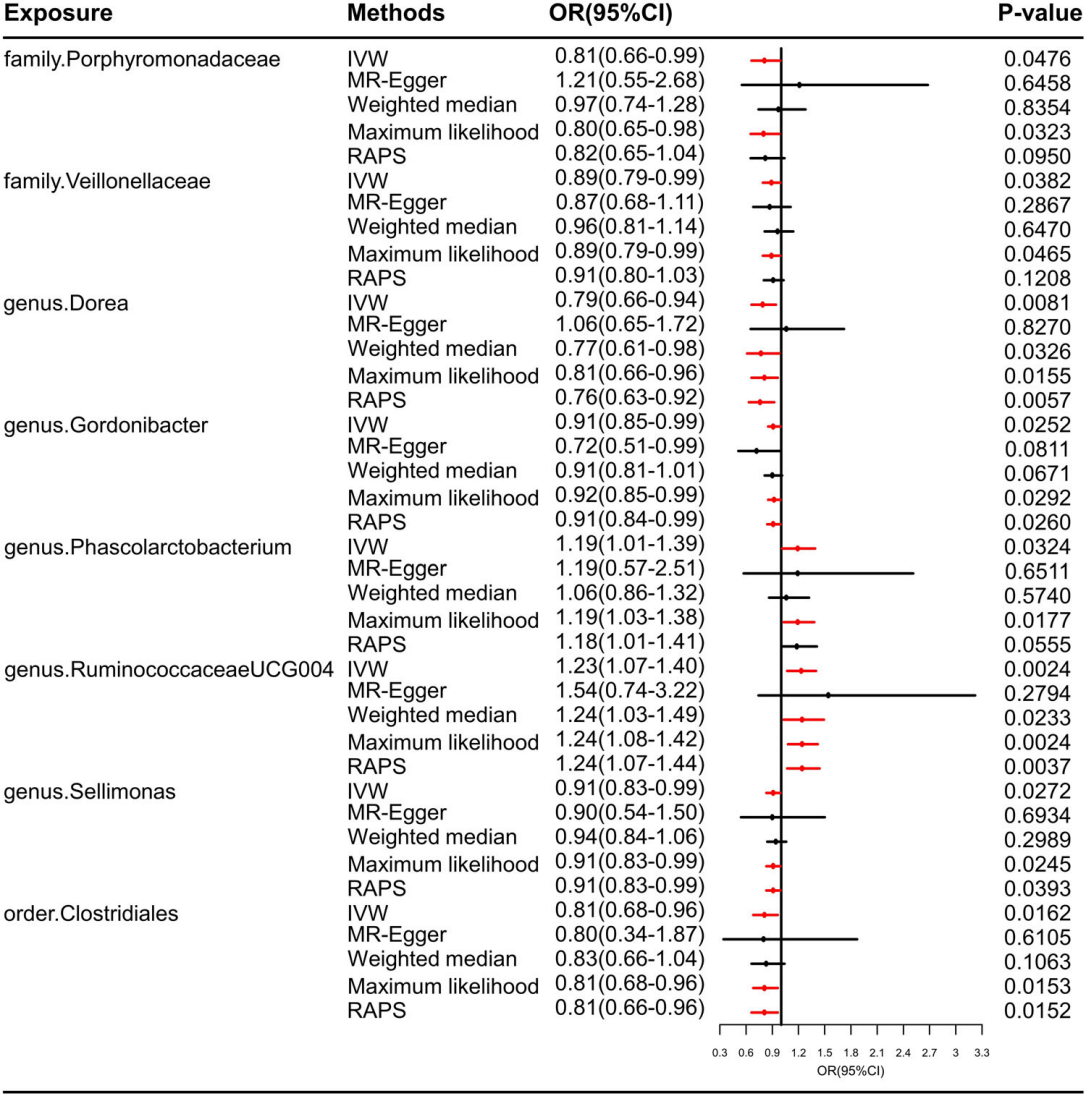
The frost of positive MR results for the causal effects of the gut microbiome on PTSD in Freeze 2 datasets in MR analyses. PTSD post-traumatic stress disorder, IVW inverse variance weighted, RAPS robust adjusted profile score.

The influence of 196 bacterial taxa on the risk of developing PTSD in the Freeze 2 datasets was examined, and the findings are presented in Table S2 and Fig. 2. Remarkably, Table 1 and Fig. 3 highlight the positive results obtained from MR analysis, demonstrating a causal relationship between the gut microbiome and PTSD. By the IVW method, it was found that eight specific bacterial traits exhibited a potential causal association with PTSD.

**Table 1:** Positive MR results of causal links between gut microbiome and PTSD in Freeze 2 datasets in MR analysis.

Outcome	Exposure	Method	No. SNP	*P*val	OR (95%CI)
**PTSD**	family				
	Porphyromonadaceae	IVW	10	0.0476	0.81 (0.66–0.99)
	Veillonellaceae	IVW	19	0.0382	0.89 (0.79–0.99)
	genus				
	Dorea	IVW	10	0.0081	0.79 (0.66–0.94)
	Gordonibacter	IVW	12	0.0252	0.91 (0.85–0.99)
	Phascolarctobacterium	IVW	11	0.0324	1.19 (1.01–1.39)
	RuminococcaceaeUCG004	IVW	11	0.0024	1.23 (1.07–1.40)
	Sellimonas	IVW	9	0.0272	0.91 (0.83–0.99)
	order				
	Clostridiales	IVW	13	0.0162	0.81 (0.68–0.96)

*MR* mendelian randomization, *OR* odds ratio, *CI* confidential interval, *PTSD* post-traumatic stress disorder.

As shown in Fig. 3, genetically predicted *family.Porphyromonadaceae* (odds ratios(ORs)=0.81, 95% confidential interval[CI] = 0.66–0.99, *P* = 0.0476), *family.Veillonellaceae* (ORs=0.89, 95%CI = 0.79–0.99, *P* = 0.0382), *genus.Dorea* (ORs = 0.79, 95%CI = 0.66–0.94, *P* = 0.0081), *genus.Gordonibacter* (ORs=0.91, 95%CI = 0.85–0.99, *P* = 0.0252), *genus.Sellimonas* (ORs = 0.91, 95%CI = 0.83–0.99, *P* = 0.0272), *order.Clostridiales* (ORs=0.81, 95%CI = 0.68–0.96, *P* = 0.0162) decreased the risk of PTSD, while *genus.Phascolarctobacterium* (ORs = 1.19, 95%CI = 1.01–1.39, *P* = 0.0324) and *genus.RuminococcaceaeUCG004* (ORs=1.23, 95%CI = 1.07–1.40, *P* = 0.0024) increased the PTSD risk. Maximum likelihood analyses revealed a similar trend.

During the sensitivity analyses, a meticulous leave-one-out approach was employed to evaluate the robustness of the results. Notably, this analysis revealed no significant SNPs associated with PTSD in the Freeze 2 datasets during the MR analysis. The outcomes of this analysis can be observed in Figure S1, reaffirming the reliability and validity of the findings. Furthermore, the MR-Egger analysis, as shown in Table S3, demonstrated no indications of pleiotropy. Moreover, Cochran’s Q test provided no evidence of substantial variability. These comprehensive sensitivity analyses further uphold the consistency and integrity of the research outcomes.

### Causal effects of the genetically predicted gut microbiome on PTSD in FinnGen datasets in MR analysis

**Fig. 4:**
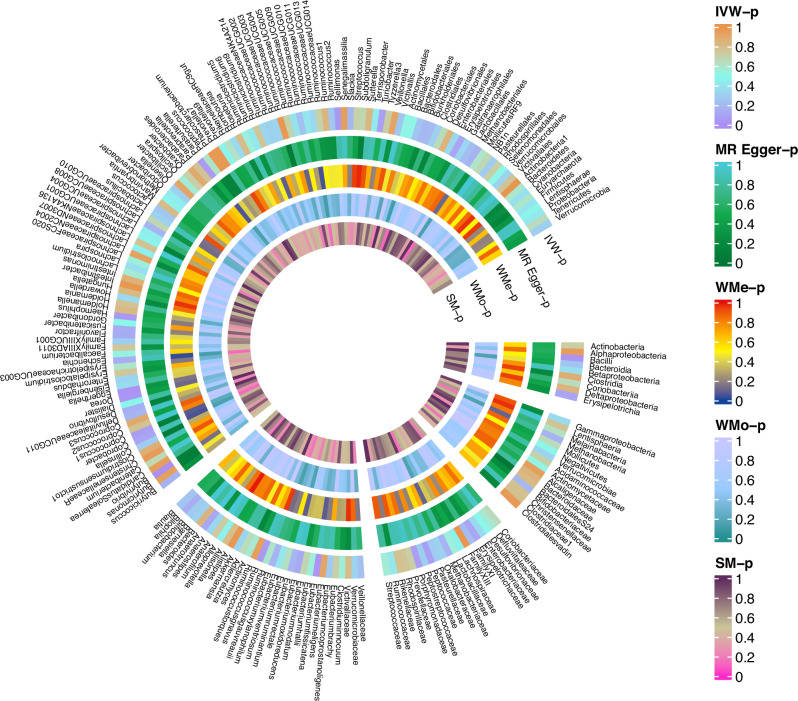
Causal effect of the gut microbiome on PTSD in FinnGen datasets based on MR analyses. From outside to inside, the *P* values of IVW, MR Egger, WMe, WMo, and SM are represented. PTSD post-traumatic stress disorder, IVW inverse variance weighted, WMe weighted median, WMo weighted mode, SM simple mode. MR mendelian randomization.

**Fig. 5:**
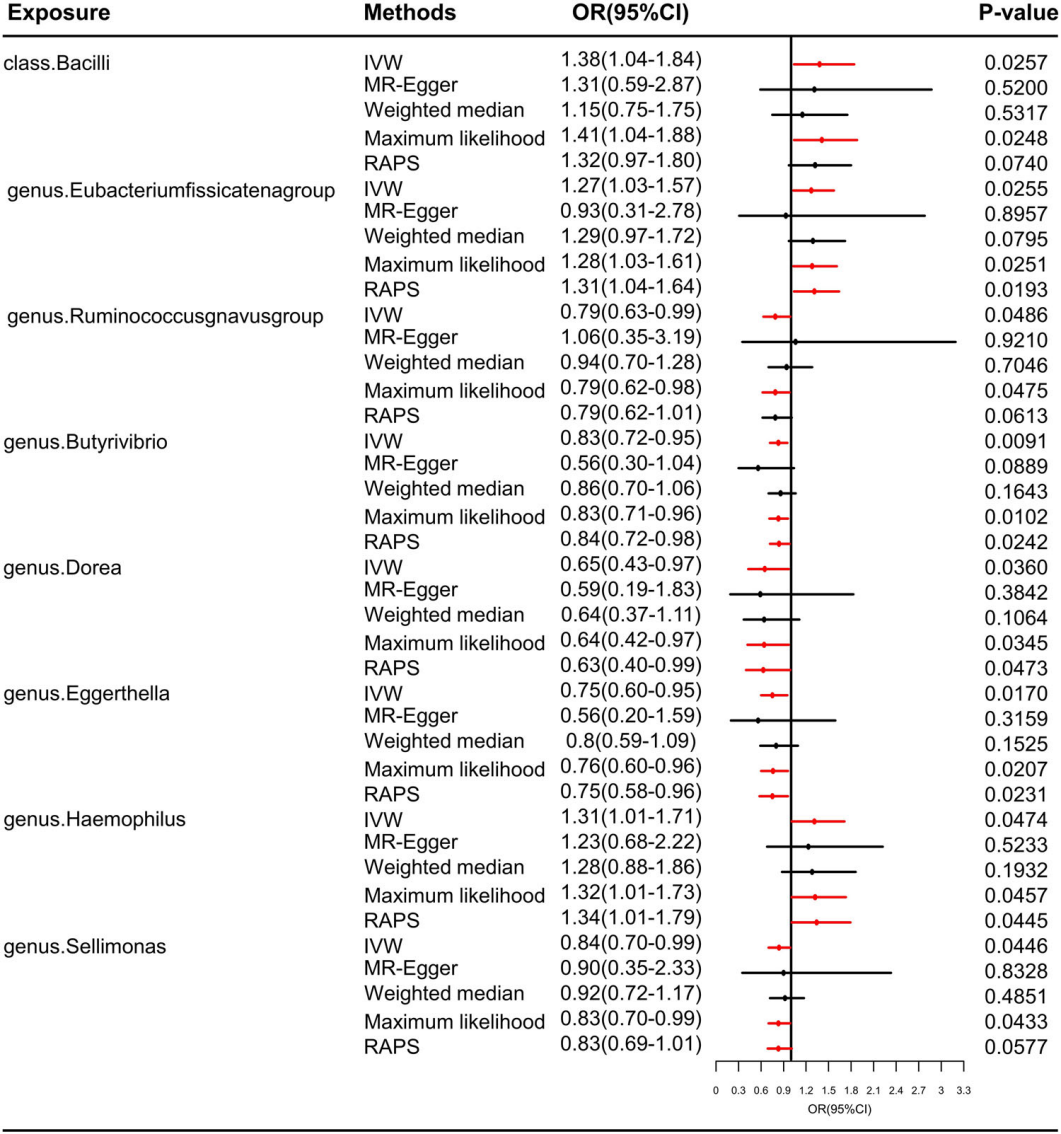
The frost of positive MR results for the causal effects of the gut microbiome on PTSD in FinnGen datasets in MR analyses. PTSD post-traumatic stress disorder, IVW inverse variance weighted, RAPS robust adjusted profile score.

Table S4 and Fig. 4 elegantly illustrate the causal effects of 196 bacterial taxa on the risk of PTSD in the FinnGen datasets. Furthermore, Table 2 and Fig. 5 present compelling evidence from the MR analysis, indicating a positive relationship between the gut microbiome and PTSD in the FinnGen datasets. Remarkably, the results obtained through the IVW method reveal that eight specific bacterial traits exhibit a direct potential causal association between the gut microbiome and PTSD in the FinnGen datasets.

**Table 2:** Positive MR results of causal links between gut microbiome and PTSD in FinnGen datasets in MR analysis.

Outcome	Exposure	Method	No. SNP	*P*val	OR (95%CI)
**PTSD**	class				
	Bacilli	IVW	18	0.0257	1.38 (1.04–1.84)
	genus				
	Eubacteriumfissicatenagroup	IVW	9	0.0255	1.27 (1.03–1.57)
	Ruminococcusgnavusgroup	IVW	11	0.0486	0.79 (0.63–0.99)
	Butyrivibrio	IVW	15	0.0091	0.83 (0.72–0.95)
	Dorea	IVW	10	0.0360	0.65 (0.43–0.97)
	Eggerthella	IVW	9	0.0170	0.75 (0.60–0.95)
	Haemophilus	IVW	9	0.0474	1.31 (1.01–1.71)
	Sellimonas	IVW	9	0.0446	0.84 (0.70–0.99)

*MR* mendelian randomization, *OR* odds ratio, *CI* confidential interval, *PTSD* post-traumatic stress disorder.

Specifically, PTSD risk was increased by *class. Bacilli* (ORs = 1.38, 95%CI = 1.04–1.84 *P* = 0.0257), *genus.Eubacteriumfissicatenagroup* (ORs = 1.27, 95%CI = 1.03–1.57, *P* = 0.0255), and *genus.Haemophilus* (ORs = 1.31, 95%CI = 1.01–1.71, *P* = 0.0474), while the risk of PTSD was decreased by *genus.Ruminococcusgnavusgroup* (ORs=0.79, 95%CI = 0.63–0.99, *P* = 0.0486), *genus.Butyrivibrio* (ORs=0.83, 95%CI = 0.72–0.95, *P* = 0.0091), *genus.Dorea* (ORs=0.65, 95%CI = 0.43–0.97, *P* = 0.0360), *genus.Eggerthella* (ORs=0.75, 95%CI = 0.60–0.95, *P* = 0.0170), and *genus.Sellimonas* (ORs=0.84, 95%CI = 0.70–0.99, *P* = 0.0446).

Through rigorous leave-one-out analyses on PTSD within the FinnGen datasets, no statistically significant SNPs were identified, as evidenced in Figure S2. Furthermore, employing the MR-Egger method revealed no indications of pleiotropy, as outlined in Table S5. Moreover, the outcomes of Cochran’s Q test further solidify the robustness and consistency of these findings by demonstrating a lack of heterogeneity, as detailed in Table S5.

### Common bacterial taxa between Freeze 2 datasets and FinnGen datasets regarding PTSD in MR analysis

**Fig. 6:**
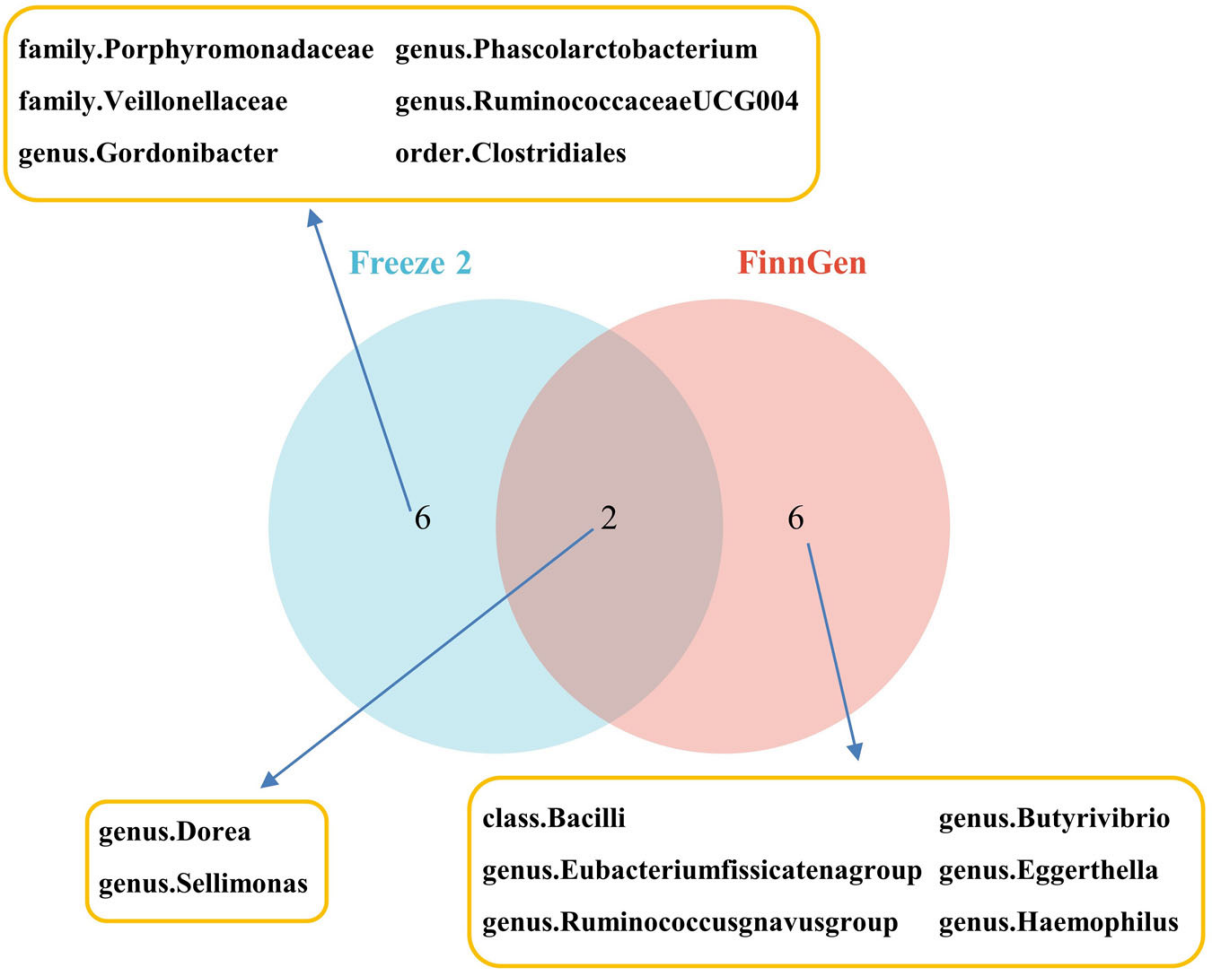
The common bacterial traits in Freeze 2 datasets and FinnGen datasets for PTSD. PTSD post-traumatic stress disorder.

Figure 6 reveals the remarkable discovery of shared bacterial traits, specifically *genus.Dorea* and *genus.Sellimonas*, in the context of PTSD by comparing the Freeze 2 datasets and FinnGen datasets through MR analysis. Intriguingly, both of these bacterial traits exhibited a profound protective influence against the development of PTSD.

### Causal effects of the genetically predicted PTSD on gut microbiome in Freeze 2 and FinnGen datasets in reverse MR analysis

Tables S6, S7 provide a comprehensive summary of the SNPs utilized as IVs in the Freeze and FinnGen datasets, respectively. In Figs. S3, S5, these visual representations demonstrate the absence of any discernible causal effects of PTSD on the gut microbiome. Moreover, meticulous scrutiny in Tables S8, S9 signifies the absence of pleiotropy and heterogeneity within the analyzed data sets. Additionally, through the thorough leave-one-out analysis depicted in Figs. S4, S6, no significant outliers were detected.

## Discussion

In this MR study, we embarked on a pioneering exploration of the intricate causal relationship between the gut microbiome and PTSD by leveraging multiple PTSD GWAS datasets. Specifically, focusing on the esteemed Freeze 2 GWAS datasets, our findings unveiled a fascinating revelation: the presence of eight distinct bacterial entities within the gut exerts profound and causal effects on the development of PTSD. Our comprehensive MR analysis revealed intriguing insights into the relationship between specific bacterial features and the risk of developing PTSD. Notably, certain genetic predictors, including *family.Porphyromonadaceae*, *family.Veillonellaceae*, *genus.Dorea*, *genus.Gordonibacter*, *genus.Sellimonas*, and *order.Clostridiales*, exhibited a pronounced decrease in PTSD risk. Conversely, the presence of *genus.Phascolarctobacterium* and *genus.RuminococcaceaeUCG004* heightened the likelihood of developing PTSD. Two particular bacterial traits, namely *genus.Dorea* and *genus.Sellimonas*, were consistently identified across both the Freeze 2 and FinnGen datasets, indicating a protective role against PTSD development. Specifically, PTSD risk was increased by *class.Bacilli*, *genus.Eubacteriumfissicatenagroup*, and *genus.Haemophilus*, while the risk of PTSD was decreased by *genus.Ruminococcusgnavusgroup*, *genus.Butyrivibrio*, *genus.Dorea*, *genus.Eggerthella*, and *genus.Sellimonas*. In reverse MR analysis, no causal effects of PTSD on gut microbiome were identified. Taken together, our study provides valuable new clues that advance our understanding of the causal relationship between specific bacterial features and the initiation and progression of PTSD. Moreover, it opens the door to exciting prospects for leveraging fecal microbiota transplantation (FMT) techniques to regulate these targeted bacterial traits, offering promising avenues for both the prevention and treatment of PTSD.

A notable distinction emerges when comparing individuals with PTSD to those without the condition. Both preclinical and clinical investigations have uncovered compelling evidence pointing towards a disruption in the balance of gut microbiota during early stages of life. A noteworthy study identified potential bacterial traits, such as *Actinobacteria*, *Lentisphaerae*, and *Verrucomicrobia*, which may be helpful in distinguishing patients with PTSD [15]. However, it is important to acknowledge several notable confounding factors. Firstly, the study’s cross-sectional design imposes limitations on establishing causal relationships between the gut microbiome and PTSD. Furthermore, the relatively small sample size of 18 participants with PTSD and 12 controls raises concerns about the statistical power and generalizability of the findings. Additionally, the inclusion of control participants with comorbid psychiatric conditions, such as major depressive disorder, introduces a potential source of bias that could significantly impact the results. Moreover, the unbalanced inclusion criteria further complicate the interpretation of the study’s outcomes. These considerations underscore the necessity for future research endeavors to employ rigorous methodologies, larger and more diverse participant cohorts, and carefully controlled inclusion criteria to elucidate the intricate connections between gut microbiota and the development of PTSD. In a study concerning the impact of the COVID-19 pandemic, a significant correlation exists between the onset of PTSD and the dysbiosis of gut microbiome. The dysbiosis symptoms persists even after a six-month follow-up. This revelation signifies a vital stepping stone towards unraveling the intricate mechanisms that underlie the bidirectional relationship between the gut microbiome and mental health outcomes, shedding light on potential avenues for targeted therapeutic interventions and personalized approaches in mitigating the detrimental effects of trauma-induced disorders [18]. In this MR study, we found that the development of PTSD is causally related to the dysbiosis of bacterial features in gut. The results remain stable in sensitivity and reverse MR analyses. The preclinical study also presented the changes in *Firmicutes*, *Bacteroidetes*, *Cyanobacteria*, and *Proteobacteria* levels, which were most relevant to the exhibited fear-like and anxiety-like behaviors in rats [19]. It is worth noting that the study’s sample size is relatively modest, and not all rats in each group underwent comprehensive testing. As a consequence, the results obtained from this investigation necessitate validation and replication in future studies with larger and more comprehensive cohorts.

Current first-line treatments for PTSD, such as selective serotonin reuptake inhibitors (SSRIs) and α1-adrenoreceptor antagonists, have shown limited effectiveness in managing symptoms. There is a pressing need for alternative approaches and innovative therapies to address the complex nature of PTSD and provide better outcomes for individuals affected by this condition [35]. Unfortunately, these results are unable to be replicated [36]. 3,4-methylenedioxymethamphetamine (MDMA), as the designation of Breakthrough Therapy for promising treatment granted by the FDA in 2017, has shown to be effective and well tolerated in several clinical trials [37, 38]. In a rat model of PTSD, researchers led by Emily A Ridge found that MDMA directly impacts the components of the gut microbiome. Moreover, treatment with MDMA was shown to rapidly restore the composition of the gut microbiome [39]. Clinical interventions targeting gut microbiome, which has been revealed in depression and anxiety [40–42], may benefit the improvement of symptoms in patients with PTSD [43]. A clinical trial conducted in the United States (Identifier: NCT04150380) used *Lactobacillus rhamnosus*, as a gram-positive immunoregulatory species with anti-inflammatory and immunoregulatory properties, to treat PTSD [44]. The study emphasized the need to investigate inflammatory, metabolic, and mitochondrial dysfunctional pathways as part of PTSD therapeutics, as these areas have received limited attention the microbiome. This highlights a promising direction for future research to develop innovative treatments targeting these pathways in PTSD [45]. Overall, the findings suggest that restoring the gut microbiome could be a beneficial approach for treating PTSD.

Indeed, heightened inflammation during trauma exposure has been identified as a crucial factor in the development of PTSD. Some phenomena regarding inflammatory factors, such as preexisting increasing CRP levels [46] and elevated IL-6 measures within 24 h after trauma [47], have been shown to predict subsequent symptoms of PTSD. IL-6 release before psychosocial stress is comparable between PTSD rodent models and individuals with PTSD, suggesting its involvement in the pathogenesis of the disorder [48]. Preventing stress-induced exaggeration of IL-6 release may ameliorate the development of a PTSD-like syndrome [8]. The impairment of immunoregulation for Treg cells may result in the imbalance of the host immune system, which will lead to increased gut permeability, colitis, and serious PTSD symptoms after trauma exposure [49–52].

Dysfunction in the gut-brain axis is implicated in various psychoneurological conditions such as major depressive disorders, schizophrenia, autism spectrum disorder, and bipolar disorder, highlighting its involvement. Our study offers new evidence supporting the role of the gut-brain axis in the development of PTSD. Additionally, our MR study first clarifies the causal effects of gut microbiota in the occurrence of PTSD. The robustness of our results exists in multiple PTSD GWAS datasets. Two bacterial traits, *genus.Dorea* and *genus.Sellimonas*, showed a protective role in the development of PTSD in both Freeze 2 and FinnGen datasets. *Genus.Dorea* has been found to be associated with immune activation, such as the increasing level of IFN-γ, and its colonizing mucin regions of the gut can degrade mucins and metabolize the sialic acids [53]. The role of *genus.Sellimonas* is mainly focused on tumor. It is reported that the overrepresented of the bacterial trait in fecal specimens was observed in patients with aggressive tumors [54]. However, the role of *Genus.Dorea* and *genus.Sellimonas* in PTSD remain unknown. Future studies need to clarify the effects of these bacteria on PTSD. Furthermore, the non-invasive assessment of gut microbiome through bacterial trait analysis may be utilized in the future to evaluate the risk of PTSD, particularly in individuals with risk factors such as physical and medical conditions like cardiovascular disease, metabolic syndrome, and type II diabetes mellitus [55]. The composition of the gut microbiome can be influenced by a regular diet, and an imbalanced gut microbiome can potentially be restored through the use of probiotic supplements. The identification of specific bacterial traits can offer valuable insights for targeted therapeutic interventions. In our MR analyses, we identified several bacterial traits, and the combined benefits of these traits can potentially be harnessed through FMT.

In our MR study, we acknowledge several limitations. Firstly, to conduct sensitivity and horizontal analyses and include more SNPs, the filtering threshold for IVs was set at a relatively low significance level of *P* < 1 × 10^–5^. Secondly, the generalizability of our findings may be limited as we used GWAS summary-level data from European participants. Thirdly, not applying false discovery rate (FDR) correction could lead to potential false negatives or conservative outcomes. Future studies with strict criteria, multi-ancestry cohorts, and larger sample sizes are necessary to validate our findings and elucidate underlying mechanisms.

## Conclusion

Our study offers significant insights into the role of the gut-brain axis in the development of PTSD. We establish causal relationships between the gut microbiome and the occurrence of PTSD. The composition of the gut microbiome shows potential as biomarkers and therapeutic targets for PTSD. Further research is necessary to validate the potential causal association between the gut microbiome and PTSD and to elucidate the underlying mechanisms.

## Supplementary information

Supplementary materials

## Data Availability

The exposure and outcome datasets in this MR study are available in the MiBioGen repository (https://mibiogen.gcc.rug.nl/) [25], the original article [26], and the FinnGen consortium.
