# Correction: Potential causal association between gut microbiome and posttraumatic stress disorder

**DOI:** 10.1038/s41398-025-03628-5

**Published:** 2025-09-30

**Authors:** Qiang He, Wenjing Wang, Dingkang Xu, Yang Xiong, Chuanyuan Tao, Chao You, Lu Ma, Junpeng Ma

**Affiliations:** 1 Department of Neurosurgery, West China Hospital, Sichuan University, 37 Guoxue Lane, Wuhou District, Chengdu, 610041 Sichuan China; 2 Department of Pharmacy, Institute of Metabolic Diseases and Pharmacotherapy, West China Hospital, Sichuan University, 37 Guoxue Lane, Wuhou District, Chengdu, China; 3 Department of Neurosurgery, Beijing Hospital, National Center of Gerontology, Institute of Geriatric Medicine, Chinese Academy of Medical Sciences, Beijing, China; 4 Department of Urology, West China Hospital, Sichuan University, Chengdu, China

**Keywords:** Depression, Clinical genetics

Correction to: *Translational Psychiatry* 10.1038/s41398-024-02765-7, published online 31 January 2024

In this article the Psychiatric Genomics Consortium Posttraumatic Stress Disorder Working Group was incorrectly listed as an author. The original article has been corrected.

