# Supplementary material for: Potential causal association between gut microbiome and posttraumatic stress disorder
Source: Transl Psychiatry. 2024 Jan 31;14:67. doi: 10.1038/s41398-024-02765-7 (PMC10831060; doi:10.1038/s41398-024-02765-7)
Supplement: Supplementary file 1 — Supplementary materials [file 41398_2024_2765_MOESM1_ESM.docx]

**Description**

**Table S1** Single-nucleotide polymorphisms used as instrumental variables in MR analysis

**Table S2** MR results of causal links between gut microbiome and PTSD in Freeze 2 datasets in MR analysis

**Table S3** The MR-Egger and Cochran’s Q test of gut microbiome on PTSD in Freeze 2 datasets in the MR analysis

**Table S4** MR results of causal links between gut microbiome and PTSD in FinnGen datasets in MR analysis

**Table S5** The MR-Egger and Cochran’s Q test of gut microbiome on PTSD in FinnGen datasets in the MR analysis

**Table S6** Single-nucleotide polymorphisms used as instrumental variables for Freeze 2 datasets in the reverse MR analysis

**Table S7** Single-nucleotide polymorphisms used as instrumental variables for FinnGen datasets in the reverse MR analysis

**Table S8** The MR-Egger and Cochran’s Q test of PTSD on gut microbiome in Freeze 2 datasets in reverse MR analysis

**Table S9** The MR-Egger and Cochran’s Q test of PTSD on gut microbiome in FinnGen datasets in reverse MR analysis

**Figure S1** The leave-one-out analyses of gut microbiome on PTSD for Freeze 2 datasets in MR analysis

**Figure S2** The leave-one-out analyses of gut microbiome on PTSD for FinnGen datasets in MR analysis

**Figure S3** Causal effect estimates of the PTSD on gut microbiome in Freeze 2 datasets in reverse MR analysis

**Figure S4** The leave-one-out analyses of PTSD on gut microbiome for Freeze 2 datasets in the reverse MR analysis

**Figure S5** Causal effect estimates of the PTSD on gut microbiome in FinnGen datasets in reverse MR analysis

**Figure S6** The leave-one-out analyses of PTSD on gut microbiome in FinnGen datasets in reverse MR analysis

**Table S1** Single-nucleotide polymorphisms used as instrumental variables in MR analysis

| **Classificationon** | **Exposure** | **SNP** | **Other allelee** | **Effect allelee** | **Beta** | **Se** | **Pval** |
| --- | --- | --- | --- | --- | --- | --- | --- |
| class | Actinobacteria | rs182549 | T | C | 0.1115 | 0.0121 | 3.79E-20 |
| class | Actinobacteria | rs6660520 | G | A | 0.0711 | 0.0134 | 1.11E-07 |
| class | Actinobacteria | rs8047955 | G | A | 0.0576 | 0.0117 | 8.34E-07 |
| class | Actinobacteria | rs7322849 | C | T | 0.0944 | 0.0193 | 6.21E-07 |
| class | Actinobacteria | rs72767435 | C | T | -0.1264 | 0.0274 | 2.57E-06 |
| class | Actinobacteria | rs134366 | A | G | 0.1119 | 0.0235 | 1.50E-06 |
| class | Actinobacteria | rs10841473 | C | G | -0.0584 | 0.0123 | 2.59E-06 |
| class | Actinobacteria | rs11745923 | T | G | 0.0564 | 0.0116 | 1.58E-06 |
| class | Actinobacteria | rs1515761 | C | T | 0.0762 | 0.0170 | 4.96E-06 |
| class | Actinobacteria | rs1961273 | T | C | 0.0603 | 0.0126 | 1.75E-06 |
| class | Actinobacteria | rs12899991 | T | A | 0.0720 | 0.0155 | 4.03E-06 |
| class | Actinobacteria | rs12049045 | G | A | 0.0510 | 0.0114 | 8.63E-06 |
| class | Actinobacteria | rs1376754 | A | G | 0.0509 | 0.0113 | 6.71E-06 |
| class | Actinobacteria | rs11655079 | C | T | -0.0561 | 0.0124 | 5.93E-06 |
| class | Actinobacteria | rs7700479 | A | T | -0.0770 | 0.0174 | 8.62E-06 |
| class | Actinobacteria | rs80083040 | G | T | 0.1558 | 0.0348 | 8.62E-06 |
| class | Actinobacteria | rs961091 | A | G | 0.0501 | 0.0113 | 8.68E-06 |
| class | Actinobacteria | rs4945008 | G | A | 0.0541 | 0.0121 | 5.39E-06 |
| class | Actinobacteria | rs857444 | T | C | 0.0510 | 0.0115 | 8.92E-06 |
| class | Alphaproteobacteria | rs9813022 | G | A | -0.0751 | 0.0154 | 1.05E-06 |
| class | Alphaproteobacteria | rs76784716 | G | A | 0.1333 | 0.0268 | 5.09E-07 |
| class | Alphaproteobacteria | rs140912403 | T | C | -0.1607 | 0.0318 | 6.20E-07 |
| class | Alphaproteobacteria | rs34569731 | G | A | 0.0705 | 0.0158 | 7.38E-06 |
| class | Alphaproteobacteria | rs10803434 | T | A | -0.0693 | 0.0149 | 3.55E-06 |
| class | Alphaproteobacteria | rs55876211 | T | C | -0.0808 | 0.0184 | 7.54E-06 |
| class | Alphaproteobacteria | rs7960664 | A | G | 0.0969 | 0.0215 | 8.84E-06 |
| class | Alphaproteobacteria | rs17061716 | C | G | 0.0927 | 0.0208 | 9.33E-06 |
| class | Alphaproteobacteria | rs12977163 | C | G | 0.0690 | 0.0154 | 7.14E-06 |
| class | Alphaproteobacteria | rs62285697 | T | C | 0.0805 | 0.0181 | 9.76E-06 |
| class | Bacilli | rs2952251 | G | A | -0.0599 | 0.0124 | 1.08E-06 |
| class | Bacilli | rs77558518 | G | A | -0.1073 | 0.0223 | 1.34E-06 |
| class | Bacilli | rs57872228 | T | C | -0.0715 | 0.0147 | 9.22E-07 |
| class | Bacilli | rs35344081 | A | G | 0.0620 | 0.0127 | 1.01E-06 |
| class | Bacilli | rs78938557 | C | T | 0.1080 | 0.0233 | 1.07E-06 |
| class | Bacilli | rs11730038 | A | G | -0.0631 | 0.0129 | 1.96E-06 |
| class | Bacilli | rs76717940 | A | T | 0.1563 | 0.0326 | 1.07E-06 |
| class | Bacilli | rs1962325 | G | C | 0.0554 | 0.0115 | 1.38E-06 |
| class | Bacilli | rs74352383 | A | T | -0.1045 | 0.0217 | 1.53E-06 |
| class | Bacilli | rs9581006 | T | C | 0.2253 | 0.0468 | 1.79E-06 |
| class | Bacilli | rs11110282 | G | A | -0.1012 | 0.0217 | 4.85E-06 |
| class | Bacilli | rs4028634 | C | T | 0.0521 | 0.0110 | 2.21E-06 |
| class | Bacilli | rs694949 | G | A | -0.0811 | 0.0180 | 7.60E-06 |
| class | Bacilli | rs12797734 | C | T | 0.0572 | 0.0127 | 7.21E-06 |
| class | Bacilli | rs28564647 | G | T | -0.0613 | 0.0138 | 7.81E-06 |
| class | Bacilli | rs7666190 | A | C | -0.1040 | 0.0248 | 8.47E-06 |
| class | Bacilli | rs4459992 | C | T | 0.0536 | 0.0116 | 4.30E-06 |
| class | Bacilli | rs34989881 | G | A | 0.1111 | 0.0246 | 6.55E-06 |
| class | Bacilli | rs111552159 | G | C | 0.1280 | 0.0287 | 9.13E-06 |
| class | Bacilli | rs13068444 | G | A | 0.0601 | 0.0136 | 9.53E-06 |
| class | Bacilli | rs1595463 | A | C | 0.0477 | 0.0109 | 7.97E-06 |
| class | Bacilli | rs74663707 | T | C | 0.0979 | 0.0224 | 8.46E-06 |
| class | Bacteroidia | rs55773148 | A | G | -0.1215 | 0.0237 | 3.90E-07 |
| class | Bacteroidia | rs7631304 | A | G | -0.0646 | 0.0133 | 8.37E-07 |
| class | Bacteroidia | rs73975615 | A | G | -0.2070 | 0.0443 | 1.22E-06 |
| class | Bacteroidia | rs111845179 | C | T | 0.1026 | 0.0214 | 9.24E-07 |
| class | Bacteroidia | rs2032750 | C | T | -0.0508 | 0.0107 | 1.92E-06 |
| class | Bacteroidia | rs7546249 | A | T | -0.0567 | 0.0118 | 1.55E-06 |
| class | Bacteroidia | rs929878 | T | C | -0.0549 | 0.0122 | 4.73E-06 |
| class | Bacteroidia | rs13291169 | G | C | 0.0690 | 0.0149 | 3.75E-06 |
| class | Bacteroidia | rs17343978 | C | A | -0.0552 | 0.0120 | 8.36E-06 |
| class | Bacteroidia | rs72706335 | C | T | -0.2224 | 0.0493 | 7.66E-06 |
| class | Bacteroidia | rs11146701 | G | A | 0.0474 | 0.0105 | 7.08E-06 |
| class | Bacteroidia | rs62531359 | G | T | 0.0656 | 0.0150 | 9.09E-06 |
| class | Bacteroidia | rs4146051 | G | A | -0.1074 | 0.0246 | 8.76E-06 |
| class | Bacteroidia | rs79585701 | C | A | 0.0647 | 0.0150 | 9.99E-06 |
| class | Bacteroidia | rs4916508 | A | G | -0.0467 | 0.0105 | 8.47E-06 |
| class | Bacteroidia | rs62575403 | T | C | 0.1401 | 0.0311 | 7.06E-06 |
| class | Betaproteobacteria | rs4033856 | T | C | 0.0832 | 0.0167 | 5.17E-07 |
| class | Betaproteobacteria | rs2367850 | C | G | 0.0640 | 0.0130 | 9.12E-07 |
| class | Betaproteobacteria | rs12467854 | C | G | 0.0641 | 0.0132 | 1.10E-06 |
| class | Betaproteobacteria | rs6087811 | G | T | -0.0978 | 0.0199 | 7.44E-07 |
| class | Betaproteobacteria | rs62395635 | C | T | 0.1097 | 0.0236 | 2.94E-06 |
| class | Betaproteobacteria | rs2613606 | T | C | -0.0513 | 0.0109 | 2.20E-06 |
| class | Betaproteobacteria | rs1928341 | G | A | 0.0526 | 0.0110 | 2.02E-06 |
| class | Betaproteobacteria | rs72747231 | G | C | -0.1373 | 0.0290 | 2.06E-06 |
| class | Betaproteobacteria | rs11128180 | G | A | 0.0593 | 0.0129 | 3.67E-06 |
| class | Betaproteobacteria | rs2321387 | A | G | -0.0495 | 0.0109 | 5.80E-06 |
| class | Betaproteobacteria | rs1511453 | G | A | 0.0923 | 0.0199 | 4.76E-06 |
| class | Betaproteobacteria | rs9964679 | G | A | 0.0529 | 0.0115 | 4.85E-06 |
| class | Betaproteobacteria | rs56386628 | T | C | -0.0622 | 0.0136 | 5.87E-06 |
| class | Betaproteobacteria | rs320161 | G | A | 0.0572 | 0.0126 | 7.33E-06 |
| class | Betaproteobacteria | rs75242906 | T | C | -0.1211 | 0.0281 | 9.27E-06 |
| class | Clostridia | rs6815608 | C | T | 0.1038 | 0.0211 | 4.02E-07 |
| class | Clostridia | rs112334273 | A | G | 0.0641 | 0.0127 | 3.81E-07 |
| class | Clostridia | rs6814436 | C | T | 0.0740 | 0.0151 | 9.65E-07 |
| class | Clostridia | rs72915163 | C | T | -0.0581 | 0.0121 | 1.34E-06 |
| class | Clostridia | rs2273429 | G | A | -0.0724 | 0.0153 | 4.52E-06 |
| class | Clostridia | rs10774377 | G | A | 0.0527 | 0.0114 | 3.24E-06 |
| class | Clostridia | rs13179700 | C | T | 0.0512 | 0.0110 | 3.37E-06 |
| class | Clostridia | rs6934062 | C | G | 0.0524 | 0.0116 | 6.53E-06 |
| class | Clostridia | rs1842454 | A | G | -0.0547 | 0.0127 | 8.72E-06 |
| class | Clostridia | rs10209007 | C | G | 0.0794 | 0.0182 | 9.89E-06 |
| class | Clostridia | rs76767978 | A | G | 0.0699 | 0.0156 | 8.40E-06 |
| class | Clostridia | rs13105690 | C | T | -0.0529 | 0.0118 | 8.78E-06 |
| class | Clostridia | rs76860606 | G | C | 0.0947 | 0.0225 | 7.53E-06 |
| class | Clostridia | rs72738886 | C | T | 0.0866 | 0.0190 | 8.24E-06 |
| class | Clostridia | rs7269302 | T | A | -0.0964 | 0.0212 | 9.04E-06 |
| class | Clostridia | rs6797343 | T | G | 0.0592 | 0.0135 | 9.36E-06 |
| class | Clostridia | rs13421739 | G | C | 0.0888 | 0.0180 | 9.54E-06 |
| class | Coriobacteriia | rs719099 | G | A | 0.0778 | 0.0156 | 5.43E-07 |
| class | Coriobacteriia | rs240104 | C | T | -0.0603 | 0.0127 | 1.52E-06 |
| class | Coriobacteriia | rs11250875 | C | T | 0.0607 | 0.0131 | 4.83E-06 |
| class | Coriobacteriia | rs34739816 | T | G | 0.0965 | 0.0208 | 3.88E-06 |
| class | Coriobacteriia | rs76779974 | G | C | 0.0775 | 0.0172 | 5.63E-06 |
| class | Coriobacteriia | rs80046645 | G | C | 0.2558 | 0.0562 | 4.66E-06 |
| class | Coriobacteriia | rs45480394 | G | T | -0.0504 | 0.0113 | 9.71E-06 |
| class | Coriobacteriia | rs1816223 | G | A | -0.0586 | 0.0129 | 4.84E-06 |
| class | Coriobacteriia | rs7898585 | C | T | 0.0732 | 0.0164 | 5.25E-06 |
| class | Coriobacteriia | rs67561917 | G | A | -0.0714 | 0.0154 | 5.39E-06 |
| class | Coriobacteriia | rs3025411 | G | A | 0.0927 | 0.0209 | 8.27E-06 |
| class | Coriobacteriia | rs11656361 | C | A | 0.0773 | 0.0176 | 8.02E-06 |
| class | Coriobacteriia | rs1397793 | A | G | -0.0499 | 0.0112 | 9.77E-06 |
| class | Coriobacteriia | rs11073596 | G | T | 0.0510 | 0.0114 | 8.14E-06 |
| class | Coriobacteriia | rs2442778 | A | G | -0.1164 | 0.0259 | 9.03E-06 |
| class | Coriobacteriia | rs7540303 | T | C | 0.0483 | 0.0109 | 9.62E-06 |
| class | Coriobacteriia | rs62448869 | A | T | -0.0486 | 0.0109 | 7.87E-06 |
| class | Coriobacteriia | rs13307134 | T | C | 0.0566 | 0.0126 | 7.80E-06 |
| class | Coriobacteriia | rs4880783 | T | G | 0.0565 | 0.0125 | 5.90E-06 |
| class | Coriobacteriia | rs12974142 | A | G | 0.0790 | 0.0177 | 8.51E-06 |
| class | Coriobacteriia | rs8010111 | A | G | -0.1034 | 0.0229 | 6.90E-06 |
| class | Deltaproteobacteria | rs6058181 | T | C | 0.0826 | 0.0166 | 3.40E-07 |
| class | Deltaproteobacteria | rs4506934 | T | C | -0.0937 | 0.0201 | 3.59E-06 |
| class | Deltaproteobacteria | rs17791387 | G | A | -0.0736 | 0.0154 | 1.60E-06 |
| class | Deltaproteobacteria | rs2838334 | A | G | 0.0562 | 0.0124 | 5.45E-06 |
| class | Deltaproteobacteria | rs9928243 | A | C | -0.0538 | 0.0118 | 5.02E-06 |
| class | Deltaproteobacteria | rs11599763 | C | T | -0.0544 | 0.0117 | 3.94E-06 |
| class | Deltaproteobacteria | rs112381107 | T | C | 0.2066 | 0.0457 | 4.63E-06 |
| class | Deltaproteobacteria | rs17084793 | A | G | -0.0711 | 0.0160 | 5.69E-06 |
| class | Deltaproteobacteria | rs2692012 | G | A | 0.1103 | 0.0253 | 3.14E-06 |
| class | Deltaproteobacteria | rs1035691 | G | A | -0.0552 | 0.0122 | 9.65E-06 |
| class | Deltaproteobacteria | rs62020470 | G | A | -0.0585 | 0.0129 | 4.85E-06 |
| class | Deltaproteobacteria | rs16851319 | C | G | -0.0706 | 0.0151 | 5.68E-06 |
| class | Deltaproteobacteria | rs3935584 | T | C | -0.0523 | 0.0116 | 7.50E-06 |
| class | Deltaproteobacteria | rs55744759 | G | A | -0.0780 | 0.0171 | 7.31E-06 |
| class | Erysipelotrichia | rs62504403 | T | C | 0.0681 | 0.0128 | 1.12E-07 |
| class | Erysipelotrichia | rs7234058 | C | T | -0.0946 | 0.0194 | 9.12E-07 |
| class | Erysipelotrichia | rs17530232 | G | A | 0.1030 | 0.0225 | 2.79E-06 |
| class | Erysipelotrichia | rs2300774 | A | G | 0.0524 | 0.0107 | 8.95E-07 |
| class | Erysipelotrichia | rs35161940 | C | T | -0.0806 | 0.0168 | 1.85E-06 |
| class | Erysipelotrichia | rs10781552 | T | C | -0.0552 | 0.0116 | 2.33E-06 |
| class | Erysipelotrichia | rs8003149 | T | C | 0.0539 | 0.0117 | 4.08E-06 |
| class | Erysipelotrichia | rs4078432 | T | C | -0.0609 | 0.0134 | 4.23E-06 |
| class | Erysipelotrichia | rs1074800 | G | A | 0.0492 | 0.0109 | 6.15E-06 |
| class | Erysipelotrichia | rs56970041 | G | T | 0.0724 | 0.0164 | 5.40E-06 |
| class | Erysipelotrichia | rs7826267 | G | T | -0.0839 | 0.0199 | 9.28E-06 |
| class | Erysipelotrichia | rs1884466 | T | C | -0.0475 | 0.0107 | 9.53E-06 |
| class | Erysipelotrichia | rs290833 | G | T | -0.0497 | 0.0111 | 8.03E-06 |
| class | Gammaproteobacteria | rs6706173 | C | A | 0.0743 | 0.0146 | 1.99E-07 |
| class | Gammaproteobacteria | rs11181912 | A | G | -0.0579 | 0.0119 | 9.95E-07 |
| class | Gammaproteobacteria | rs79795896 | G | A | -0.1592 | 0.0351 | 7.92E-06 |
| class | Gammaproteobacteria | rs2234691 | C | T | 0.0763 | 0.0172 | 8.39E-06 |
| class | Gammaproteobacteria | rs6469506 | A | T | 0.0541 | 0.0116 | 3.29E-06 |
| class | Gammaproteobacteria | rs12404135 | G | A | -0.0789 | 0.0172 | 8.89E-06 |
| class | Gammaproteobacteria | rs9973122 | A | T | 0.0741 | 0.0163 | 6.77E-06 |
| class | Gammaproteobacteria | rs9494710 | T | C | -0.0550 | 0.0121 | 4.55E-06 |
| class | Gammaproteobacteria | rs75101789 | T | C | 0.0729 | 0.0163 | 8.79E-06 |
| class | Lentisphaeria | rs2825714 | G | A | -0.1374 | 0.0289 | 1.72E-06 |
| class | Lentisphaeria | rs17114848 | A | G | 0.1524 | 0.0324 | 4.06E-06 |
| class | Lentisphaeria | rs77599476 | G | A | 0.2303 | 0.0480 | 1.86E-06 |
| class | Lentisphaeria | rs11770843 | T | C | 0.1094 | 0.0235 | 1.91E-06 |
| class | Lentisphaeria | rs72640280 | G | A | 0.2202 | 0.0486 | 5.18E-06 |
| class | Lentisphaeria | rs62570196 | T | C | -0.2164 | 0.0440 | 1.08E-06 |
| class | Lentisphaeria | rs2731834 | G | C | 0.1094 | 0.0237 | 4.24E-06 |
| class | Lentisphaeria | rs2031282 | G | A | 0.1224 | 0.0270 | 4.38E-06 |
| class | Lentisphaeria | rs1002941 | A | G | 0.1050 | 0.0233 | 8.15E-06 |
| class | Lentisphaeria | rs73113483 | A | T | -0.1312 | 0.0289 | 8.66E-06 |
| class | Melainabacteria | rs9864379 | C | T | -0.1597 | 0.0293 | 5.36E-08 |
| class | Melainabacteria | rs11150282 | C | T | 0.0989 | 0.0197 | 6.03E-07 |
| class | Melainabacteria | rs16851659 | C | G | -0.0896 | 0.0186 | 1.27E-06 |
| class | Melainabacteria | rs73074665 | T | A | 0.1662 | 0.0356 | 2.87E-06 |
| class | Melainabacteria | rs4129395 | A | G | 0.0896 | 0.0185 | 1.48E-06 |
| class | Melainabacteria | rs79790072 | C | T | 0.2267 | 0.0488 | 3.29E-06 |
| class | Melainabacteria | rs10738747 | A | G | 0.0815 | 0.0184 | 9.96E-06 |
| class | Melainabacteria | rs1221147 | A | T | 0.1240 | 0.0279 | 7.11E-06 |
| class | Melainabacteria | rs113884518 | C | T | -0.2054 | 0.0455 | 8.06E-06 |
| class | Melainabacteria | rs789069 | C | A | -0.1035 | 0.0234 | 6.85E-06 |
| class | Melainabacteria | rs367480 | A | G | -0.0838 | 0.0186 | 8.20E-06 |
| class | Melainabacteria | rs28678345 | C | T | 0.2148 | 0.0471 | 6.69E-06 |
| class | Melainabacteria | rs10148250 | A | G | 0.0862 | 0.0193 | 8.67E-06 |
| class | Methanobacteria | rs10202904 | G | T | -0.1218 | 0.0235 | 3.01E-07 |
| class | Methanobacteria | rs73457410 | G | A | 0.2153 | 0.0437 | 1.41E-06 |
| class | Methanobacteria | rs6776814 | C | T | -0.1996 | 0.0412 | 1.63E-06 |
| class | Methanobacteria | rs894996 | A | C | 0.2170 | 0.0449 | 1.88E-06 |
| class | Methanobacteria | rs75208022 | T | C | -0.2272 | 0.0488 | 5.92E-06 |
| class | Methanobacteria | rs12825290 | G | C | -0.2168 | 0.0493 | 6.08E-06 |
| class | Methanobacteria | rs56131665 | A | G | 0.1787 | 0.0393 | 6.18E-06 |
| class | Methanobacteria | rs11018665 | T | A | 0.1115 | 0.0251 | 6.52E-06 |
| class | Methanobacteria | rs4257531 | A | G | 0.1645 | 0.0365 | 7.44E-06 |
| class | Methanobacteria | rs6508769 | C | T | 0.1535 | 0.0345 | 8.23E-06 |
| class | Methanobacteria | rs73068003 | T | G | -0.1581 | 0.0352 | 8.45E-06 |
| class | Methanobacteria | rs10424197 | A | G | -0.1113 | 0.0248 | 9.28E-06 |
| class | Mollicutes | rs74603314 | C | T | 0.2216 | 0.0463 | 1.56E-06 |
| class | Mollicutes | rs10108398 | A | G | 0.0769 | 0.0154 | 1.09E-06 |
| class | Mollicutes | rs11890098 | G | A | 0.0744 | 0.0153 | 9.57E-07 |
| class | Mollicutes | rs72901605 | C | T | -0.0842 | 0.0178 | 3.26E-06 |
| class | Mollicutes | rs3768491 | G | A | -0.0681 | 0.0149 | 4.23E-06 |
| class | Mollicutes | rs17214486 | A | C | 0.0610 | 0.0136 | 6.61E-06 |
| class | Mollicutes | rs12566890 | G | T | -0.1011 | 0.0231 | 3.65E-06 |
| class | Mollicutes | rs78169027 | G | A | -0.1083 | 0.0237 | 5.88E-06 |
| class | Mollicutes | rs4885016 | C | T | -0.0820 | 0.0182 | 7.27E-06 |
| class | Mollicutes | rs28537087 | A | G | 0.0821 | 0.0188 | 8.07E-06 |
| class | Mollicutes | rs2464826 | C | A | 0.0944 | 0.0212 | 8.40E-06 |
| class | Mollicutes | rs6043847 | C | T | -0.1149 | 0.0249 | 4.55E-06 |
| class | Negativicutes | rs1643968 | C | T | -0.0565 | 0.0112 | 4.15E-07 |
| class | Negativicutes | rs60274479 | C | T | -0.0660 | 0.0134 | 1.16E-06 |
| class | Negativicutes | rs13086907 | A | G | 0.0625 | 0.0132 | 1.95E-06 |
| class | Negativicutes | rs73232831 | A | G | -0.1518 | 0.0315 | 1.87E-06 |
| class | Negativicutes | rs71405394 | A | G | -0.1142 | 0.0240 | 2.17E-06 |
| class | Negativicutes | rs61249479 | C | A | 0.0777 | 0.0169 | 2.95E-06 |
| class | Negativicutes | rs1447205 | C | G | -0.0502 | 0.0108 | 2.93E-06 |
| class | Negativicutes | rs4722181 | G | T | 0.0501 | 0.0106 | 2.00E-06 |
| class | Negativicutes | rs4463806 | C | T | -0.0544 | 0.0129 | 7.81E-06 |
| class | Negativicutes | rs9423647 | A | G | 0.0478 | 0.0105 | 6.06E-06 |
| class | Negativicutes | rs2834062 | G | A | 0.0489 | 0.0109 | 8.44E-06 |
| class | Negativicutes | rs1649999 | G | A | 0.0749 | 0.0166 | 7.58E-06 |
| class | Negativicutes | rs1135612 | A | G | 0.0530 | 0.0119 | 9.26E-06 |
| class | Verrucomicrobiae | rs11729256 | C | T | 0.0750 | 0.0150 | 6.73E-07 |
| class | Verrucomicrobiae | rs4936098 | G | A | 0.0649 | 0.0136 | 1.12E-06 |
| class | Verrucomicrobiae | rs9349825 | G | A | -0.0704 | 0.0147 | 2.54E-06 |
| class | Verrucomicrobiae | rs74542928 | C | T | 0.1122 | 0.0236 | 1.63E-06 |
| class | Verrucomicrobiae | rs3995795 | T | C | 0.0641 | 0.0141 | 5.19E-06 |
| class | Verrucomicrobiae | rs2602429 | T | C | 0.0747 | 0.0156 | 2.58E-06 |
| class | Verrucomicrobiae | rs4242783 | A | G | 0.0689 | 0.0148 | 2.64E-06 |
| class | Verrucomicrobiae | rs11184341 | C | G | 0.0655 | 0.0142 | 4.13E-06 |
| class | Verrucomicrobiae | rs61779207 | A | G | -0.0758 | 0.0168 | 6.72E-06 |
| class | Verrucomicrobiae | rs117107102 | G | A | 0.2047 | 0.0432 | 2.92E-06 |
| class | Verrucomicrobiae | rs12908520 | A | G | 0.0619 | 0.0131 | 2.17E-06 |
| class | Verrucomicrobiae | rs111862613 | C | T | 0.0907 | 0.0197 | 3.74E-06 |
| class | Verrucomicrobiae | rs941682 | A | G | -0.0631 | 0.0144 | 9.61E-06 |
| family | Acidaminococcaceae | rs6923842 | C | T | -0.0796 | 0.0169 | 2.21E-06 |
| family | Acidaminococcaceae | rs6589457 | G | A | 0.1659 | 0.0350 | 2.32E-06 |
| family | Acidaminococcaceae | rs2933324 | G | A | -0.0663 | 0.0140 | 2.24E-06 |
| family | Acidaminococcaceae | rs262812 | C | T | -0.0657 | 0.0142 | 3.25E-06 |
| family | Acidaminococcaceae | rs74540770 | A | G | -0.1090 | 0.0244 | 7.09E-06 |
| family | Acidaminococcaceae | rs6427992 | C | G | -0.0596 | 0.0130 | 4.24E-06 |
| family | Acidaminococcaceae | rs45497800 | C | T | -0.1179 | 0.0257 | 5.86E-06 |
| family | Acidaminococcaceae | rs78702810 | C | T | -0.1438 | 0.0323 | 9.16E-06 |
| family | Actinomycetaceae | rs35011108 | G | A | 0.2418 | 0.0504 | 1.83E-06 |
| family | Actinomycetaceae | rs2889192 | T | G | 0.0888 | 0.0195 | 3.64E-06 |
| family | Actinomycetaceae | rs34583783 | T | G | 0.1238 | 0.0264 | 5.48E-06 |
| family | Actinomycetaceae | rs58484246 | C | T | 0.0761 | 0.0169 | 6.12E-06 |
| family | Actinomycetaceae | rs4073240 | A | G | 0.0748 | 0.0165 | 6.05E-06 |
| family | Alcaligenaceae | rs62191117 | G | A | 0.0684 | 0.0134 | 2.76E-07 |
| family | Alcaligenaceae | rs9537886 | C | A | -0.0571 | 0.0111 | 2.35E-07 |
| family | Alcaligenaceae | rs4033856 | T | C | 0.0818 | 0.0169 | 1.03E-06 |
| family | Alcaligenaceae | rs67737557 | G | C | 0.0738 | 0.0152 | 1.04E-06 |
| family | Alcaligenaceae | rs17000015 | T | A | -0.0609 | 0.0125 | 1.13E-06 |
| family | Alcaligenaceae | rs2367850 | C | G | 0.0629 | 0.0132 | 1.85E-06 |
| family | Alcaligenaceae | rs7638039 | C | T | 0.0603 | 0.0128 | 2.70E-06 |
| family | Alcaligenaceae | rs62395635 | C | T | 0.1106 | 0.0239 | 3.35E-06 |
| family | Alcaligenaceae | rs112159068 | T | A | 0.1315 | 0.0281 | 3.56E-06 |
| family | Alcaligenaceae | rs6969323 | C | A | -0.0594 | 0.0129 | 3.89E-06 |
| family | Alcaligenaceae | rs7302582 | C | G | -0.1068 | 0.0233 | 3.34E-06 |
| family | Alcaligenaceae | rs1153990 | G | A | -0.0587 | 0.0128 | 5.97E-06 |
| family | Alcaligenaceae | rs9964679 | G | A | 0.0537 | 0.0116 | 4.47E-06 |
| family | Alcaligenaceae | rs112135816 | G | T | -0.0775 | 0.0172 | 5.28E-06 |
| family | Alcaligenaceae | rs2040996 | C | T | -0.0491 | 0.0110 | 7.80E-06 |
| family | Alcaligenaceae | rs147968 | T | C | 0.0489 | 0.0110 | 9.13E-06 |
| family | Alcaligenaceae | rs74776516 | G | T | -0.0944 | 0.0213 | 6.85E-06 |
| family | Alcaligenaceae | rs28480294 | C | T | -0.0517 | 0.0116 | 6.61E-06 |
| family | Alcaligenaceae | rs79846372 | G | A | 0.0882 | 0.0198 | 6.99E-06 |
| family | Bacteroidaceae | rs6795673 | T | C | 0.0539 | 0.0105 | 3.38E-07 |
| family | Bacteroidaceae | rs28757219 | A | T | 0.0818 | 0.0170 | 1.29E-06 |
| family | Bacteroidaceae | rs9507307 | T | C | 0.0604 | 0.0129 | 2.13E-06 |
| family | Bacteroidaceae | rs66474973 | T | G | 0.0813 | 0.0164 | 6.81E-07 |
| family | Bacteroidaceae | rs11585893 | G | A | -0.0741 | 0.0148 | 1.80E-06 |
| family | Bacteroidaceae | rs495004 | G | C | -0.0607 | 0.0130 | 3.42E-06 |
| family | Bacteroidaceae | rs17619981 | G | T | 0.0881 | 0.0187 | 2.69E-06 |
| family | Bacteroidaceae | rs2023437 | C | T | -0.0782 | 0.0168 | 5.02E-06 |
| family | Bacteroidaceae | rs66710942 | T | C | 0.0488 | 0.0107 | 5.86E-06 |
| family | Bacteroidaceae | rs13207588 | G | A | -0.0592 | 0.0131 | 7.49E-06 |
| family | Bacteroidaceae | rs2366421 | A | T | -0.0528 | 0.0117 | 7.65E-06 |
| family | Bacteroidaceae | rs1340391 | C | T | -0.0592 | 0.0132 | 6.73E-06 |
| family | BacteroidalesS24 | rs738193 | C | T | 0.0847 | 0.0166 | 3.82E-07 |
| family | BacteroidalesS24 | rs941000 | T | C | 0.0850 | 0.0163 | 3.16E-07 |
| family | BacteroidalesS24 | rs689695 | A | C | 0.0815 | 0.0167 | 1.28E-06 |
| family | BacteroidalesS24 | rs17043785 | C | T | -0.1762 | 0.0347 | 5.12E-07 |
| family | BacteroidalesS24 | rs10872669 | G | A | -0.1231 | 0.0276 | 9.49E-06 |
| family | BacteroidalesS24 | rs12748533 | T | G | -0.0821 | 0.0173 | 2.59E-06 |
| family | BacteroidalesS24 | rs6831034 | A | T | -0.0957 | 0.0205 | 6.10E-06 |
| family | BacteroidalesS24 | rs61508842 | C | T | 0.1226 | 0.0272 | 7.83E-06 |
| family | BacteroidalesS24 | rs78609301 | G | A | -0.0867 | 0.0196 | 7.09E-06 |
| family | BacteroidalesS24 | rs11135366 | G | C | 0.0842 | 0.0184 | 8.78E-06 |
| family | BacteroidalesS24 | rs7217209 | T | C | 0.0843 | 0.0187 | 8.43E-06 |
| family | Bifidobacteriaceae | rs182549 | T | C | 0.1171 | 0.0127 | 5.94E-20 |
| family | Bifidobacteriaceae | rs7322849 | C | T | 0.1107 | 0.0201 | 1.74E-08 |
| family | Bifidobacteriaceae | rs1961273 | T | C | 0.0688 | 0.0132 | 1.70E-07 |
| family | Bifidobacteriaceae | rs76671854 | G | C | -0.0893 | 0.0183 | 1.04E-06 |
| family | Bifidobacteriaceae | rs4957061 | C | T | 0.0570 | 0.0117 | 1.15E-06 |
| family | Bifidobacteriaceae | rs10841473 | C | G | -0.0613 | 0.0129 | 2.28E-06 |
| family | Bifidobacteriaceae | rs4567981 | A | T | 0.0578 | 0.0117 | 8.88E-07 |
| family | Bifidobacteriaceae | rs13020688 | A | G | 0.0584 | 0.0122 | 1.57E-06 |
| family | Bifidobacteriaceae | rs677208 | C | G | 0.0729 | 0.0165 | 6.00E-06 |
| family | Bifidobacteriaceae | rs12446429 | C | T | 0.0810 | 0.0191 | 8.53E-06 |
| family | Bifidobacteriaceae | rs11655079 | C | T | -0.0586 | 0.0130 | 5.92E-06 |
| family | Bifidobacteriaceae | rs540489 | G | T | -0.0633 | 0.0138 | 5.37E-06 |
| family | Bifidobacteriaceae | rs857444 | T | C | 0.0554 | 0.0121 | 3.82E-06 |
| family | Bifidobacteriaceae | rs10857328 | A | T | 0.0562 | 0.0126 | 7.24E-06 |
| family | Bifidobacteriaceae | rs10831953 | A | G | 0.0538 | 0.0124 | 9.95E-06 |
| family | Bifidobacteriaceae | rs7174549 | T | C | 0.0552 | 0.0125 | 6.87E-06 |
| family | Bifidobacteriaceae | rs73797465 | G | T | -0.0943 | 0.0208 | 4.85E-06 |
| family | Bifidobacteriaceae | rs55888705 | G | A | 0.0537 | 0.0121 | 8.66E-06 |
| family | Bifidobacteriaceae | rs6899771 | G | A | -0.0914 | 0.0203 | 7.28E-06 |
| family | Bifidobacteriaceae | rs7588568 | C | A | -0.0607 | 0.0131 | 3.73E-06 |
| family | Christensenellaceae | rs62573205 | A | G | -0.0655 | 0.0134 | 1.49E-06 |
| family | Christensenellaceae | rs870002 | T | C | 0.0489 | 0.0110 | 6.54E-06 |
| family | Christensenellaceae | rs12657403 | G | A | 0.0781 | 0.0172 | 5.59E-06 |
| family | Christensenellaceae | rs117186816 | A | G | -0.2065 | 0.0436 | 6.52E-06 |
| family | Christensenellaceae | rs9608766 | C | T | -0.0682 | 0.0150 | 3.33E-06 |
| family | Christensenellaceae | rs4076564 | A | G | -0.1886 | 0.0392 | 8.06E-06 |
| family | Christensenellaceae | rs4805124 | G | C | 0.0487 | 0.0109 | 8.36E-06 |
| family | Christensenellaceae | rs72706624 | T | G | 0.0883 | 0.0198 | 6.38E-06 |
| family | Christensenellaceae | rs77867022 | T | G | -0.1604 | 0.0380 | 8.82E-06 |
| family | Christensenellaceae | rs7211194 | T | C | 0.0492 | 0.0111 | 9.94E-06 |
| family | Christensenellaceae | rs892686 | G | A | 0.0506 | 0.0110 | 4.12E-06 |
| family | Christensenellaceae | rs12380890 | G | A | -0.0504 | 0.0111 | 5.78E-06 |
| family | Clostridiaceae1 | rs12186080 | A | G | 0.0748 | 0.0162 | 5.34E-06 |
| family | Clostridiaceae1 | rs2795528 | A | G | -0.1810 | 0.0391 | 3.81E-06 |
| family | Clostridiaceae1 | rs550843 | C | T | -0.0735 | 0.0168 | 7.09E-06 |
| family | Clostridiaceae1 | rs12341505 | A | G | 0.0815 | 0.0179 | 4.54E-06 |
| family | Clostridiaceae1 | rs4723021 | C | T | -0.1064 | 0.0242 | 7.42E-06 |
| family | Clostridiaceae1 | rs881532 | G | A | -0.0533 | 0.0119 | 7.90E-06 |
| family | Clostridiaceae1 | rs2817172 | T | C | 0.0563 | 0.0124 | 5.27E-06 |
| family | Clostridiaceae1 | rs10875374 | T | C | -0.0537 | 0.0119 | 8.10E-06 |
| family | Clostridiaceae1 | rs12490337 | G | C | -0.0617 | 0.0137 | 6.91E-06 |
| family | Clostridiaceae1 | rs62397761 | G | A | 0.0616 | 0.0136 | 9.08E-06 |
| family | Clostridiaceae1 | rs56188186 | G | A | 0.0968 | 0.0217 | 8.24E-06 |
| family | ClostridialesvadinBB60group | rs55682560 | T | C | -0.1315 | 0.0261 | 4.97E-07 |
| family | ClostridialesvadinBB60group | rs9979874 | C | G | -0.0739 | 0.0151 | 1.05E-06 |
| family | ClostridialesvadinBB60group | rs28691777 | T | C | 0.1371 | 0.0267 | 6.96E-07 |
| family | ClostridialesvadinBB60group | rs6588624 | A | G | -0.0662 | 0.0138 | 1.79E-06 |
| family | ClostridialesvadinBB60group | rs7538034 | G | T | -0.0786 | 0.0166 | 2.37E-06 |
| family | ClostridialesvadinBB60group | rs66714985 | C | A | 0.1169 | 0.0252 | 4.85E-06 |
| family | ClostridialesvadinBB60group | rs118104867 | T | C | 0.2145 | 0.0455 | 3.44E-06 |
| family | ClostridialesvadinBB60group | rs2191834 | T | G | 0.0746 | 0.0159 | 2.50E-06 |
| family | ClostridialesvadinBB60group | rs7725895 | G | A | -0.1162 | 0.0240 | 3.94E-06 |
| family | ClostridialesvadinBB60group | rs7226487 | G | A | -0.0644 | 0.0139 | 3.58E-06 |
| family | ClostridialesvadinBB60group | rs10517600 | G | T | 0.0627 | 0.0139 | 6.83E-06 |
| family | ClostridialesvadinBB60group | rs17121075 | A | G | 0.0769 | 0.0172 | 7.91E-06 |
| family | ClostridialesvadinBB60group | rs10904722 | T | C | -0.0672 | 0.0147 | 5.05E-06 |
| family | ClostridialesvadinBB60group | rs6755871 | G | C | -0.0614 | 0.0139 | 9.33E-06 |
| family | ClostridialesvadinBB60group | rs34088226 | G | A | -0.1178 | 0.0269 | 7.66E-06 |
| family | ClostridialesvadinBB60group | rs13409132 | G | A | -0.1654 | 0.0352 | 4.37E-06 |
| family | ClostridialesvadinBB60group | rs989682 | G | A | 0.0702 | 0.0155 | 6.85E-06 |
| family | Coriobacteriaceae | rs719099 | G | A | 0.0778 | 0.0156 | 5.43E-07 |
| family | Coriobacteriaceae | rs240104 | C | T | -0.0603 | 0.0127 | 1.52E-06 |
| family | Coriobacteriaceae | rs11250875 | C | T | 0.0607 | 0.0131 | 4.83E-06 |
| family | Coriobacteriaceae | rs34739816 | T | G | 0.0965 | 0.0208 | 3.88E-06 |
| family | Coriobacteriaceae | rs76779974 | G | C | 0.0775 | 0.0172 | 5.63E-06 |
| family | Coriobacteriaceae | rs80046645 | G | C | 0.2558 | 0.0562 | 4.66E-06 |
| family | Coriobacteriaceae | rs45480394 | G | T | -0.0504 | 0.0113 | 9.71E-06 |
| family | Coriobacteriaceae | rs1816223 | G | A | -0.0586 | 0.0129 | 4.84E-06 |
| family | Coriobacteriaceae | rs7898585 | C | T | 0.0732 | 0.0164 | 5.25E-06 |
| family | Coriobacteriaceae | rs67561917 | G | A | -0.0714 | 0.0154 | 5.39E-06 |
| family | Coriobacteriaceae | rs3025411 | G | A | 0.0927 | 0.0209 | 8.27E-06 |
| family | Coriobacteriaceae | rs11656361 | C | A | 0.0773 | 0.0176 | 8.02E-06 |
| family | Coriobacteriaceae | rs1397793 | A | G | -0.0499 | 0.0112 | 9.77E-06 |
| family | Coriobacteriaceae | rs11073596 | G | T | 0.0510 | 0.0114 | 8.14E-06 |
| family | Coriobacteriaceae | rs2442778 | A | G | -0.1164 | 0.0259 | 9.03E-06 |
| family | Coriobacteriaceae | rs7540303 | T | C | 0.0483 | 0.0109 | 9.62E-06 |
| family | Coriobacteriaceae | rs62448869 | A | T | -0.0486 | 0.0109 | 7.87E-06 |
| family | Coriobacteriaceae | rs13307134 | T | C | 0.0566 | 0.0126 | 7.80E-06 |
| family | Coriobacteriaceae | rs4880783 | T | G | 0.0565 | 0.0125 | 5.90E-06 |
| family | Coriobacteriaceae | rs12974142 | A | G | 0.0790 | 0.0177 | 8.51E-06 |
| family | Coriobacteriaceae | rs8010111 | A | G | -0.1034 | 0.0229 | 6.90E-06 |
| family | Defluviitaleaceae | rs72731813 | T | C | -0.1498 | 0.0293 | 2.76E-07 |
| family | Defluviitaleaceae | rs4677103 | G | A | 0.0977 | 0.0197 | 9.42E-07 |
| family | Defluviitaleaceae | rs55658617 | C | T | 0.1773 | 0.0362 | 1.41E-06 |
| family | Defluviitaleaceae | rs9725395 | G | A | -0.1384 | 0.0295 | 3.41E-06 |
| family | Defluviitaleaceae | rs17051335 | T | C | -0.1343 | 0.0292 | 4.58E-06 |
| family | Defluviitaleaceae | rs112893842 | C | T | 0.1108 | 0.0233 | 2.75E-06 |
| family | Defluviitaleaceae | rs28696126 | T | A | -0.1071 | 0.0238 | 5.56E-06 |
| family | Defluviitaleaceae | rs1582238 | C | T | 0.0802 | 0.0167 | 1.69E-06 |
| family | Defluviitaleaceae | rs1908593 | C | T | 0.0702 | 0.0157 | 7.86E-06 |
| family | Defluviitaleaceae | rs9608282 | G | T | 0.1390 | 0.0299 | 4.61E-06 |
| family | Defluviitaleaceae | rs4344384 | T | G | 0.0709 | 0.0156 | 5.86E-06 |
| family | Defluviitaleaceae | rs540220 | C | T | -0.1240 | 0.0290 | 9.48E-06 |
| family | Desulfovibrionaceae | rs6058181 | T | C | 0.0835 | 0.0166 | 2.70E-07 |
| family | Desulfovibrionaceae | rs4506934 | T | C | -0.0943 | 0.0201 | 3.16E-06 |
| family | Desulfovibrionaceae | rs2692012 | G | A | 0.1142 | 0.0254 | 1.56E-06 |
| family | Desulfovibrionaceae | rs11599763 | C | T | -0.0556 | 0.0117 | 2.50E-06 |
| family | Desulfovibrionaceae | rs2838334 | A | G | 0.0571 | 0.0124 | 3.82E-06 |
| family | Desulfovibrionaceae | rs112381107 | T | C | 0.2114 | 0.0457 | 2.82E-06 |
| family | Desulfovibrionaceae | rs16851319 | C | G | -0.0733 | 0.0151 | 2.40E-06 |
| family | Desulfovibrionaceae | rs17791387 | G | A | -0.0729 | 0.0154 | 2.10E-06 |
| family | Desulfovibrionaceae | rs9928243 | A | C | -0.0542 | 0.0118 | 4.48E-06 |
| family | Desulfovibrionaceae | rs7164160 | T | A | -0.0574 | 0.0129 | 7.34E-06 |
| family | Desulfovibrionaceae | rs3935584 | T | C | -0.0526 | 0.0116 | 6.78E-06 |
| family | Desulfovibrionaceae | rs72647048 | C | T | -0.0770 | 0.0171 | 9.61E-06 |
| family | Enterobacteriaceae | rs78143293 | G | A | -0.0849 | 0.0170 | 1.20E-06 |
| family | Enterobacteriaceae | rs111229068 | T | A | 0.1106 | 0.0242 | 3.65E-06 |
| family | Enterobacteriaceae | rs62210023 | G | A | 0.0607 | 0.0130 | 3.13E-06 |
| family | Enterobacteriaceae | rs504442 | G | T | 0.0842 | 0.0189 | 5.17E-06 |
| family | Enterobacteriaceae | rs80319214 | G | C | 0.0990 | 0.0216 | 6.95E-06 |
| family | Enterobacteriaceae | rs11026530 | C | T | 0.0822 | 0.0186 | 9.43E-06 |
| family | Enterobacteriaceae | rs2374342 | A | C | 0.0583 | 0.0126 | 4.52E-06 |
| family | Enterobacteriaceae | rs79757635 | A | C | 0.0759 | 0.0171 | 9.32E-06 |
| family | Enterobacteriaceae | rs35673018 | A | G | 0.0900 | 0.0203 | 7.63E-06 |
| family | Enterobacteriaceae | rs61973590 | G | C | -0.0605 | 0.0134 | 8.54E-06 |
| family | Enterobacteriaceae | rs4792380 | T | A | 0.1156 | 0.0258 | 9.49E-06 |
| family | Erysipelotrichaceae | rs62504403 | T | C | 0.0681 | 0.0128 | 1.12E-07 |
| family | Erysipelotrichaceae | rs7234058 | C | T | -0.0946 | 0.0194 | 9.12E-07 |
| family | Erysipelotrichaceae | rs17530232 | G | A | 0.1030 | 0.0225 | 2.79E-06 |
| family | Erysipelotrichaceae | rs2300774 | A | G | 0.0524 | 0.0107 | 8.95E-07 |
| family | Erysipelotrichaceae | rs35161940 | C | T | -0.0806 | 0.0168 | 1.85E-06 |
| family | Erysipelotrichaceae | rs10781552 | T | C | -0.0552 | 0.0116 | 2.33E-06 |
| family | Erysipelotrichaceae | rs8003149 | T | C | 0.0539 | 0.0117 | 4.08E-06 |
| family | Erysipelotrichaceae | rs4078432 | T | C | -0.0609 | 0.0134 | 4.23E-06 |
| family | Erysipelotrichaceae | rs1074800 | G | A | 0.0492 | 0.0109 | 6.15E-06 |
| family | Erysipelotrichaceae | rs56970041 | G | T | 0.0724 | 0.0164 | 5.40E-06 |
| family | Erysipelotrichaceae | rs7826267 | G | T | -0.0839 | 0.0199 | 9.28E-06 |
| family | Erysipelotrichaceae | rs1884466 | T | C | -0.0475 | 0.0107 | 9.53E-06 |
| family | Erysipelotrichaceae | rs290833 | G | T | -0.0497 | 0.0111 | 8.03E-06 |
| family | FamilyXI | rs2155352 | G | A | -0.1505 | 0.0302 | 6.63E-07 |
| family | FamilyXI | rs697771 | G | A | -0.1177 | 0.0251 | 3.19E-06 |
| family | FamilyXI | rs10759623 | T | C | -0.1621 | 0.0322 | 5.78E-07 |
| family | FamilyXI | rs11547158 | G | A | -0.1776 | 0.0373 | 2.70E-06 |
| family | FamilyXI | rs488164 | G | T | 0.1180 | 0.0255 | 4.80E-06 |
| family | FamilyXI | rs3733511 | G | A | 0.1283 | 0.0275 | 3.39E-06 |
| family | FamilyXI | rs6025153 | C | G | 0.1186 | 0.0262 | 7.07E-06 |
| family | FamilyXI | rs78222136 | C | G | 0.2248 | 0.0512 | 8.41E-06 |
| family | FamilyXI | rs17379710 | C | T | -0.1164 | 0.0252 | 3.97E-06 |
| family | FamilyXI | rs2156611 | C | T | -0.1125 | 0.0251 | 9.43E-06 |
| family | FamilyXIII | rs6501525 | G | A | 0.0562 | 0.0116 | 1.24E-06 |
| family | FamilyXIII | rs118170811 | G | A | 0.1516 | 0.0317 | 1.80E-06 |
| family | FamilyXIII | rs12643275 | T | A | -0.0551 | 0.0116 | 2.42E-06 |
| family | FamilyXIII | rs1999289 | T | A | -0.0637 | 0.0139 | 2.54E-06 |
| family | FamilyXIII | rs482905 | T | G | 0.0597 | 0.0127 | 3.72E-06 |
| family | FamilyXIII | rs3098182 | T | G | 0.0509 | 0.0110 | 4.04E-06 |
| family | FamilyXIII | rs6797051 | T | C | -0.0806 | 0.0171 | 4.89E-06 |
| family | FamilyXIII | rs66753613 | A | G | 0.0651 | 0.0144 | 8.08E-06 |
| family | FamilyXIII | rs7514702 | C | T | -0.0663 | 0.0142 | 3.92E-06 |
| family | FamilyXIII | rs7076829 | A | T | -0.0516 | 0.0112 | 4.76E-06 |
| family | FamilyXIII | rs10404377 | A | C | -0.0503 | 0.0112 | 6.99E-06 |
| family | FamilyXIII | rs4293737 | G | A | -0.1121 | 0.0271 | 8.38E-06 |
| family | FamilyXIII | rs1887048 | A | G | 0.0580 | 0.0130 | 9.65E-06 |
| family | FamilyXIII | rs12904405 | G | A | -0.0852 | 0.0193 | 7.67E-06 |
| family | Lachnospiraceae | rs9929145 | A | G | -0.1257 | 0.0245 | 2.84E-07 |
| family | Lachnospiraceae | rs11755180 | G | C | -0.0492 | 0.0105 | 2.69E-06 |
| family | Lachnospiraceae | rs35524804 | C | T | -0.0607 | 0.0125 | 2.45E-06 |
| family | Lachnospiraceae | rs11979110 | C | T | -0.0501 | 0.0105 | 1.82E-06 |
| family | Lachnospiraceae | rs79086868 | C | T | 0.0777 | 0.0164 | 3.01E-06 |
| family | Lachnospiraceae | rs112040820 | G | A | 0.0550 | 0.0117 | 2.42E-06 |
| family | Lachnospiraceae | rs2159863 | G | A | -0.0586 | 0.0129 | 3.70E-06 |
| family | Lachnospiraceae | rs11139361 | C | T | 0.0494 | 0.0111 | 4.26E-06 |
| family | Lachnospiraceae | rs1205443 | G | A | 0.0501 | 0.0112 | 7.29E-06 |
| family | Lachnospiraceae | rs7359994 | C | T | -0.0503 | 0.0112 | 5.36E-06 |
| family | Lachnospiraceae | rs146660815 | C | T | -0.2151 | 0.0477 | 6.53E-06 |
| family | Lachnospiraceae | rs959845 | T | C | -0.0494 | 0.0108 | 5.17E-06 |
| family | Lachnospiraceae | rs10402491 | T | C | 0.0662 | 0.0149 | 7.58E-06 |
| family | Lachnospiraceae | rs13005175 | G | A | 0.0993 | 0.0218 | 8.37E-06 |
| family | Lachnospiraceae | rs2910921 | C | T | 0.1603 | 0.0358 | 8.42E-06 |
| family | Lachnospiraceae | rs12760724 | C | A | -0.0485 | 0.0108 | 7.27E-06 |
| family | Lachnospiraceae | rs3127230 | T | C | -0.0504 | 0.0112 | 6.20E-06 |
| family | Lachnospiraceae | rs11841382 | T | G | -0.0718 | 0.0175 | 9.58E-06 |
| family | Lactobacillaceae | rs16861661 | A | G | -0.1933 | 0.0379 | 2.70E-07 |
| family | Lactobacillaceae | rs921925 | C | A | 0.0999 | 0.0202 | 5.77E-07 |
| family | Lactobacillaceae | rs768253 | G | T | -0.0792 | 0.0171 | 3.61E-06 |
| family | Lactobacillaceae | rs6092149 | T | A | -0.0826 | 0.0170 | 1.38E-06 |
| family | Lactobacillaceae | rs328312 | A | T | 0.0825 | 0.0168 | 8.69E-07 |
| family | Lactobacillaceae | rs11674854 | T | C | -0.0830 | 0.0175 | 2.60E-06 |
| family | Lactobacillaceae | rs820999 | G | T | -0.0758 | 0.0170 | 8.89E-06 |
| family | Lactobacillaceae | rs77478751 | G | A | -0.2192 | 0.0474 | 5.96E-06 |
| family | Lactobacillaceae | rs74599091 | G | A | 0.1916 | 0.0425 | 7.70E-06 |
| family | Lactobacillaceae | rs9345899 | G | A | -0.1240 | 0.0279 | 9.45E-06 |
| family | Lactobacillaceae | rs1590596 | G | C | -0.1038 | 0.0220 | 3.90E-06 |
| family | Lactobacillaceae | rs1530559 | A | G | 0.0772 | 0.0177 | 9.65E-06 |
| family | Lactobacillaceae | rs62314653 | A | C | 0.1771 | 0.0392 | 6.59E-06 |
| family | Methanobacteriaceae | rs10202904 | G | T | -0.1218 | 0.0235 | 3.01E-07 |
| family | Methanobacteriaceae | rs73457410 | G | A | 0.2153 | 0.0437 | 1.41E-06 |
| family | Methanobacteriaceae | rs6776814 | C | T | -0.1996 | 0.0412 | 1.63E-06 |
| family | Methanobacteriaceae | rs894996 | A | C | 0.2170 | 0.0449 | 1.88E-06 |
| family | Methanobacteriaceae | rs75208022 | T | C | -0.2272 | 0.0488 | 5.92E-06 |
| family | Methanobacteriaceae | rs12825290 | G | C | -0.2168 | 0.0493 | 6.08E-06 |
| family | Methanobacteriaceae | rs56131665 | A | G | 0.1787 | 0.0393 | 6.18E-06 |
| family | Methanobacteriaceae | rs11018665 | T | A | 0.1115 | 0.0251 | 6.52E-06 |
| family | Methanobacteriaceae | rs4257531 | A | G | 0.1645 | 0.0365 | 7.44E-06 |
| family | Methanobacteriaceae | rs6508769 | C | T | 0.1535 | 0.0345 | 8.23E-06 |
| family | Methanobacteriaceae | rs73068003 | T | G | -0.1581 | 0.0352 | 8.45E-06 |
| family | Methanobacteriaceae | rs10424197 | A | G | -0.1113 | 0.0248 | 9.28E-06 |
| family | Oxalobacteraceae | rs4428215 | A | G | 0.1256 | 0.0230 | 4.88E-08 |
| family | Oxalobacteraceae | rs1569853 | C | T | -0.1400 | 0.0282 | 7.45E-07 |
| family | Oxalobacteraceae | rs736744 | T | C | 0.1056 | 0.0201 | 1.49E-07 |
| family | Oxalobacteraceae | rs6000536 | T | C | -0.1184 | 0.0241 | 7.39E-07 |
| family | Oxalobacteraceae | rs36057338 | T | G | 0.1816 | 0.0399 | 6.26E-06 |
| family | Oxalobacteraceae | rs7993559 | A | C | 0.0921 | 0.0201 | 5.04E-06 |
| family | Oxalobacteraceae | rs12002250 | C | A | 0.1961 | 0.0445 | 5.53E-06 |
| family | Oxalobacteraceae | rs111966731 | C | T | 0.2040 | 0.0446 | 4.56E-06 |
| family | Oxalobacteraceae | rs17138946 | T | G | -0.1894 | 0.0430 | 8.09E-06 |
| family | Oxalobacteraceae | rs11246212 | C | T | 0.1361 | 0.0292 | 4.51E-06 |
| family | Oxalobacteraceae | rs934049 | A | G | 0.1098 | 0.0239 | 4.21E-06 |
| family | Oxalobacteraceae | rs12509763 | G | C | -0.1633 | 0.0361 | 8.05E-06 |
| family | Oxalobacteraceae | rs561239 | G | A | 0.1055 | 0.0237 | 7.19E-06 |
| family | Oxalobacteraceae | rs62435498 | A | C | 0.1815 | 0.0401 | 7.46E-06 |
| family | Oxalobacteraceae | rs80330081 | C | A | -0.1876 | 0.0424 | 6.64E-06 |
| family | Pasteurellaceae | rs12191680 | G | C | 0.1022 | 0.0197 | 3.05E-07 |
| family | Pasteurellaceae | rs9382510 | T | C | -0.0882 | 0.0170 | 2.48E-07 |
| family | Pasteurellaceae | rs10840326 | G | C | -0.0718 | 0.0149 | 1.44E-06 |
| family | Pasteurellaceae | rs76022354 | T | C | 0.2429 | 0.0500 | 1.83E-06 |
| family | Pasteurellaceae | rs72756943 | A | G | 0.1399 | 0.0303 | 3.35E-06 |
| family | Pasteurellaceae | rs78909003 | C | T | -0.2412 | 0.0498 | 2.05E-06 |
| family | Pasteurellaceae | rs6092684 | T | A | 0.0685 | 0.0147 | 3.44E-06 |
| family | Pasteurellaceae | rs4822728 | C | T | 0.0685 | 0.0149 | 4.72E-06 |
| family | Pasteurellaceae | rs35510 | G | A | 0.1227 | 0.0265 | 4.02E-06 |
| family | Pasteurellaceae | rs10965428 | A | C | -0.1199 | 0.0258 | 4.29E-06 |
| family | Pasteurellaceae | rs16970009 | G | A | 0.1874 | 0.0430 | 7.32E-06 |
| family | Pasteurellaceae | rs12050685 | G | A | -0.0671 | 0.0152 | 9.19E-06 |
| family | Pasteurellaceae | rs73139353 | C | A | -0.2226 | 0.0485 | 8.71E-06 |
| family | Pasteurellaceae | rs6972479 | G | A | -0.0782 | 0.0175 | 7.75E-06 |
| family | Pasteurellaceae | rs9895850 | C | T | -0.1764 | 0.0410 | 9.08E-06 |
| family | Pasteurellaceae | rs111582866 | A | G | -0.1140 | 0.0256 | 7.07E-06 |
| family | Pasteurellaceae | rs62568866 | A | T | -0.1180 | 0.0259 | 7.23E-06 |
| family | Pasteurellaceae | rs731534 | G | C | -0.0987 | 0.0216 | 9.46E-06 |
| family | Pasteurellaceae | rs9938097 | C | T | -0.0710 | 0.0158 | 8.23E-06 |
| family | Peptococcaceae | rs35703006 | T | G | 0.0813 | 0.0164 | 4.95E-07 |
| family | Peptococcaceae | rs4990837 | G | A | 0.0910 | 0.0186 | 1.74E-06 |
| family | Peptococcaceae | rs150600492 | C | A | 0.1358 | 0.0290 | 2.31E-06 |
| family | Peptococcaceae | rs12634826 | G | T | -0.0740 | 0.0151 | 1.01E-06 |
| family | Peptococcaceae | rs12992764 | G | T | 0.0684 | 0.0141 | 1.46E-06 |
| family | Peptococcaceae | rs75898026 | G | A | -0.0822 | 0.0174 | 2.02E-06 |
| family | Peptococcaceae | rs117452796 | G | A | -0.2580 | 0.0550 | 3.15E-06 |
| family | Peptococcaceae | rs75430375 | T | C | -0.1479 | 0.0317 | 3.41E-06 |
| family | Peptococcaceae | rs12144792 | T | C | 0.0644 | 0.0141 | 5.82E-06 |
| family | Peptococcaceae | rs11787651 | G | A | 0.1289 | 0.0289 | 8.29E-06 |
| family | Peptostreptococcaceae | rs61841503 | A | G | 0.0920 | 0.0161 | 9.80E-09 |
| family | Peptostreptococcaceae | rs4692811 | T | C | 0.0642 | 0.0127 | 4.21E-07 |
| family | Peptostreptococcaceae | rs12377846 | A | C | -0.2520 | 0.0512 | 7.26E-07 |
| family | Peptostreptococcaceae | rs2644627 | G | C | -0.0559 | 0.0112 | 6.33E-07 |
| family | Peptostreptococcaceae | rs59987323 | T | C | -0.0537 | 0.0114 | 2.70E-06 |
| family | Peptostreptococcaceae | rs9573937 | G | A | -0.0694 | 0.0143 | 1.71E-06 |
| family | Peptostreptococcaceae | rs75819860 | A | T | 0.1263 | 0.0268 | 2.49E-06 |
| family | Peptostreptococcaceae | rs117020988 | T | C | 0.1824 | 0.0372 | 1.03E-06 |
| family | Peptostreptococcaceae | rs1520207 | T | C | 0.0526 | 0.0112 | 3.17E-06 |
| family | Peptostreptococcaceae | rs76982728 | C | T | 0.1242 | 0.0267 | 3.24E-06 |
| family | Peptostreptococcaceae | rs12986312 | G | T | 0.0572 | 0.0126 | 5.77E-06 |
| family | Peptostreptococcaceae | rs10805326 | A | G | 0.0567 | 0.0123 | 4.03E-06 |
| family | Peptostreptococcaceae | rs1467258 | A | G | 0.0726 | 0.0162 | 7.90E-06 |
| family | Peptostreptococcaceae | rs59865771 | T | C | -0.0574 | 0.0127 | 7.69E-06 |
| family | Peptostreptococcaceae | rs77540684 | G | T | 0.1067 | 0.0246 | 8.14E-06 |
| family | Peptostreptococcaceae | rs6721459 | G | A | 0.0510 | 0.0112 | 5.08E-06 |
| family | Porphyromonadaceae | rs10119172 | G | C | -0.0737 | 0.0143 | 1.32E-07 |
| family | Porphyromonadaceae | rs17065783 | G | A | -0.0591 | 0.0122 | 1.79E-06 |
| family | Porphyromonadaceae | rs6953849 | G | A | 0.0718 | 0.0151 | 2.44E-06 |
| family | Porphyromonadaceae | rs10858364 | T | G | 0.0553 | 0.0121 | 4.31E-06 |
| family | Porphyromonadaceae | rs12486680 | C | A | 0.0985 | 0.0219 | 4.29E-06 |
| family | Porphyromonadaceae | rs3111851 | G | C | 0.0582 | 0.0121 | 2.19E-06 |
| family | Porphyromonadaceae | rs7330827 | C | T | -0.1038 | 0.0237 | 8.05E-06 |
| family | Porphyromonadaceae | rs35961441 | C | A | 0.0915 | 0.0207 | 8.37E-06 |
| family | Porphyromonadaceae | rs10762312 | A | G | -0.0524 | 0.0119 | 8.70E-06 |
| family | Porphyromonadaceae | rs35233670 | C | T | -0.0474 | 0.0106 | 7.91E-06 |
| family | Porphyromonadaceae | rs864093 | C | A | -0.0528 | 0.0117 | 9.60E-06 |
| family | Porphyromonadaceae | rs1980561 | G | A | -0.0485 | 0.0109 | 8.95E-06 |
| family | Prevotellaceae | rs9958960 | A | G | -0.0915 | 0.0174 | 1.06E-07 |
| family | Prevotellaceae | rs4493272 | C | T | -0.0605 | 0.0118 | 3.02E-07 |
| family | Prevotellaceae | rs3860225 | G | A | 0.0839 | 0.0168 | 5.50E-07 |
| family | Prevotellaceae | rs912860 | A | G | -0.2290 | 0.0483 | 9.30E-07 |
| family | Prevotellaceae | rs12118202 | C | T | -0.0752 | 0.0147 | 5.54E-07 |
| family | Prevotellaceae | rs2206482 | G | T | -0.0569 | 0.0117 | 1.30E-06 |
| family | Prevotellaceae | rs9586501 | A | G | 0.0593 | 0.0127 | 2.59E-06 |
| family | Prevotellaceae | rs12057990 | T | C | 0.0590 | 0.0132 | 8.97E-06 |
| family | Prevotellaceae | rs148376875 | G | T | 0.0845 | 0.0179 | 2.08E-06 |
| family | Prevotellaceae | rs4685827 | C | T | -0.0680 | 0.0145 | 2.77E-06 |
| family | Prevotellaceae | rs10131942 | G | C | 0.0701 | 0.0158 | 8.61E-06 |
| family | Prevotellaceae | rs34660375 | G | A | -0.0806 | 0.0178 | 7.40E-06 |
| family | Prevotellaceae | rs7252711 | G | A | -0.0741 | 0.0162 | 5.57E-06 |
| family | Prevotellaceae | rs3758087 | C | T | 0.0564 | 0.0125 | 8.61E-06 |
| family | Prevotellaceae | rs34071565 | A | T | -0.1567 | 0.0348 | 5.60E-06 |
| family | Prevotellaceae | rs13069367 | C | A | -0.0540 | 0.0120 | 7.39E-06 |
| family | Prevotellaceae | rs7975087 | A | C | -0.0601 | 0.0135 | 7.59E-06 |
| family | Prevotellaceae | rs2278540 | A | G | 0.0554 | 0.0123 | 8.44E-06 |
| family | Rhodospirillaceae | rs9813022 | G | A | -0.0842 | 0.0164 | 2.53E-07 |
| family | Rhodospirillaceae | rs3754624 | T | C | 0.0971 | 0.0200 | 1.71E-06 |
| family | Rhodospirillaceae | rs55876211 | T | C | -0.0912 | 0.0196 | 2.87E-06 |
| family | Rhodospirillaceae | rs76784716 | G | A | 0.1360 | 0.0286 | 1.49E-06 |
| family | Rhodospirillaceae | rs4278423 | C | T | 0.1075 | 0.0236 | 3.12E-06 |
| family | Rhodospirillaceae | rs1549633 | C | A | 0.0999 | 0.0219 | 4.70E-06 |
| family | Rhodospirillaceae | rs11591293 | T | G | 0.0743 | 0.0159 | 2.67E-06 |
| family | Rhodospirillaceae | rs74354280 | T | C | -0.0912 | 0.0205 | 6.67E-06 |
| family | Rhodospirillaceae | rs61933850 | A | G | 0.1648 | 0.0361 | 7.23E-06 |
| family | Rhodospirillaceae | rs7001029 | T | C | 0.1174 | 0.0261 | 5.35E-06 |
| family | Rhodospirillaceae | rs1035406 | A | G | -0.1135 | 0.0251 | 5.84E-06 |
| family | Rhodospirillaceae | rs72714493 | G | A | 0.0817 | 0.0181 | 7.35E-06 |
| family | Rhodospirillaceae | rs12977163 | C | G | 0.0731 | 0.0163 | 7.63E-06 |
| family | Rhodospirillaceae | rs13336560 | T | C | -0.0702 | 0.0158 | 9.17E-06 |
| family | Rhodospirillaceae | rs4822789 | C | G | 0.0734 | 0.0161 | 7.01E-06 |
| family | Rhodospirillaceae | rs6679026 | C | T | 0.1120 | 0.0251 | 9.95E-06 |
| family | Rhodospirillaceae | rs1923415 | G | A | -0.0999 | 0.0227 | 9.64E-06 |
| family | Rikenellaceae | rs9603208 | T | G | 0.0821 | 0.0160 | 1.92E-07 |
| family | Rikenellaceae | rs1939881 | A | G | -0.1056 | 0.0206 | 5.64E-07 |
| family | Rikenellaceae | rs67705352 | G | T | -0.0550 | 0.0111 | 6.58E-07 |
| family | Rikenellaceae | rs4264350 | C | T | -0.0526 | 0.0109 | 1.35E-06 |
| family | Rikenellaceae | rs62532512 | A | C | -0.0504 | 0.0107 | 2.76E-06 |
| family | Rikenellaceae | rs67281112 | C | G | 0.0636 | 0.0137 | 3.63E-06 |
| family | Rikenellaceae | rs6837275 | G | A | 0.0570 | 0.0119 | 1.45E-06 |
| family | Rikenellaceae | rs9578457 | A | G | -0.1415 | 0.0316 | 3.99E-06 |
| family | Rikenellaceae | rs2833282 | A | G | 0.0711 | 0.0157 | 4.31E-06 |
| family | Rikenellaceae | rs77885767 | T | C | -0.1562 | 0.0336 | 2.85E-06 |
| family | Rikenellaceae | rs59663348 | A | G | 0.0571 | 0.0125 | 6.12E-06 |
| family | Rikenellaceae | rs8130320 | G | A | -0.0489 | 0.0107 | 4.73E-06 |
| family | Rikenellaceae | rs74474130 | G | T | 0.1377 | 0.0296 | 3.61E-06 |
| family | Rikenellaceae | rs7242694 | T | C | -0.0619 | 0.0135 | 5.81E-06 |
| family | Rikenellaceae | rs36021379 | G | A | -0.0656 | 0.0145 | 7.20E-06 |
| family | Rikenellaceae | rs10217435 | T | C | -0.0884 | 0.0198 | 6.51E-06 |
| family | Rikenellaceae | rs2447496 | A | G | -0.0549 | 0.0122 | 6.09E-06 |
| family | Rikenellaceae | rs35909684 | C | A | -0.0850 | 0.0192 | 7.10E-06 |
| family | Rikenellaceae | rs4783173 | G | C | 0.0482 | 0.0110 | 7.06E-06 |
| family | Rikenellaceae | rs7832304 | G | T | -0.0724 | 0.0161 | 8.71E-06 |
| family | Rikenellaceae | rs10832801 | C | A | -0.0535 | 0.0123 | 7.50E-06 |
| family | Rikenellaceae | rs9389714 | T | C | -0.0637 | 0.0143 | 8.79E-06 |
| family | Rikenellaceae | rs6744030 | T | C | 0.0697 | 0.0157 | 9.32E-06 |
| family | Ruminococcaceae | rs55793120 | C | T | 0.1382 | 0.0266 | 1.44E-07 |
| family | Ruminococcaceae | rs2113833 | C | T | 0.1691 | 0.0355 | 1.14E-06 |
| family | Ruminococcaceae | rs17376049 | C | T | 0.0849 | 0.0172 | 7.30E-07 |
| family | Ruminococcaceae | rs56199908 | C | T | -0.1993 | 0.0410 | 1.66E-06 |
| family | Ruminococcaceae | rs10093275 | T | C | 0.0535 | 0.0117 | 5.35E-06 |
| family | Ruminococcaceae | rs76724913 | G | T | 0.0901 | 0.0203 | 9.60E-06 |
| family | Ruminococcaceae | rs1612733 | C | T | 0.1087 | 0.0238 | 4.22E-06 |
| family | Ruminococcaceae | rs2426816 | A | T | -0.0480 | 0.0106 | 6.04E-06 |
| family | Ruminococcaceae | rs3009418 | A | C | 0.0928 | 0.0210 | 8.69E-06 |
| family | Ruminococcaceae | rs10166469 | C | T | -0.0533 | 0.0120 | 8.52E-06 |
| family | Ruminococcaceae | rs1158100 | G | A | -0.0491 | 0.0110 | 8.61E-06 |
| family | Ruminococcaceae | rs499272 | C | G | 0.0706 | 0.0160 | 9.29E-06 |
| family | Streptococcaceae | rs11110281 | C | T | -0.1306 | 0.0226 | 1.40E-08 |
| family | Streptococcaceae | rs2952251 | G | A | -0.0639 | 0.0127 | 3.72E-07 |
| family | Streptococcaceae | rs4968759 | G | A | -0.0544 | 0.0111 | 8.92E-07 |
| family | Streptococcaceae | rs395407 | C | G | -0.0827 | 0.0173 | 1.33E-06 |
| family | Streptococcaceae | rs35344081 | A | G | 0.0609 | 0.0130 | 2.64E-06 |
| family | Streptococcaceae | rs2370083 | T | G | -0.0843 | 0.0185 | 4.26E-06 |
| family | Streptococcaceae | rs77558518 | G | A | -0.1042 | 0.0228 | 3.72E-06 |
| family | Streptococcaceae | rs76717940 | A | T | 0.1506 | 0.0334 | 3.09E-06 |
| family | Streptococcaceae | rs10028567 | T | C | -0.0934 | 0.0190 | 3.72E-06 |
| family | Streptococcaceae | rs72739637 | G | A | 0.0928 | 0.0192 | 1.82E-06 |
| family | Streptococcaceae | rs16950051 | G | A | 0.1070 | 0.0237 | 5.34E-06 |
| family | Streptococcaceae | rs77968078 | A | G | -0.0993 | 0.0225 | 7.93E-06 |
| family | Streptococcaceae | rs28718126 | G | A | 0.1091 | 0.0247 | 9.41E-06 |
| family | Streptococcaceae | rs7916711 | G | A | 0.0960 | 0.0215 | 6.33E-06 |
| family | Streptococcaceae | rs957755 | G | T | -0.0642 | 0.0143 | 7.42E-06 |
| family | Streptococcaceae | rs6806351 | C | T | -0.0619 | 0.0136 | 6.94E-06 |
| family | Streptococcaceae | rs9903102 | A | C | -0.0693 | 0.0154 | 4.92E-06 |
| family | Streptococcaceae | rs57646748 | A | G | -0.0880 | 0.0199 | 7.88E-06 |
| family | Streptococcaceae | rs6563952 | C | G | 0.0802 | 0.0179 | 8.71E-06 |
| family | Veillonellaceae | rs12741784 | T | C | -0.0621 | 0.0119 | 1.28E-07 |
| family | Veillonellaceae | rs79535861 | C | A | 0.1007 | 0.0207 | 1.58E-06 |
| family | Veillonellaceae | rs111810795 | T | C | -0.0867 | 0.0181 | 1.73E-06 |
| family | Veillonellaceae | rs12186441 | A | G | 0.2081 | 0.0454 | 4.53E-06 |
| family | Veillonellaceae | rs4797169 | C | T | 0.0588 | 0.0128 | 4.49E-06 |
| family | Veillonellaceae | rs12668619 | G | A | 0.0553 | 0.0118 | 2.57E-06 |
| family | Veillonellaceae | rs2175069 | G | A | -0.0527 | 0.0115 | 4.64E-06 |
| family | Veillonellaceae | rs1442060 | A | G | -0.0514 | 0.0112 | 4.51E-06 |
| family | Veillonellaceae | rs1447205 | C | G | -0.0521 | 0.0116 | 7.35E-06 |
| family | Veillonellaceae | rs114889439 | G | A | -0.2538 | 0.0539 | 6.19E-06 |
| family | Veillonellaceae | rs4461038 | G | A | -0.0555 | 0.0119 | 3.73E-06 |
| family | Veillonellaceae | rs6909981 | T | C | -0.0639 | 0.0142 | 5.48E-06 |
| family | Veillonellaceae | rs2561116 | G | T | -0.0836 | 0.0187 | 7.89E-06 |
| family | Veillonellaceae | rs61264131 | C | A | 0.2024 | 0.0464 | 6.75E-06 |
| family | Veillonellaceae | rs9345168 | A | C | 0.0509 | 0.0113 | 8.49E-06 |
| family | Veillonellaceae | rs11700976 | A | C | 0.0503 | 0.0114 | 9.25E-06 |
| family | Veillonellaceae | rs6692542 | G | A | 0.0534 | 0.0118 | 8.68E-06 |
| family | Veillonellaceae | rs2585520 | T | G | -0.0905 | 0.0200 | 5.27E-06 |
| family | Veillonellaceae | rs75768969 | G | C | -0.0704 | 0.0157 | 7.19E-06 |
| family | Veillonellaceae | rs1693340 | C | T | 0.0819 | 0.0182 | 9.25E-06 |
| family | Veillonellaceae | rs4263802 | A | G | 0.0509 | 0.0115 | 7.45E-06 |
| family | Verrucomicrobiaceae | rs11729256 | C | T | 0.0750 | 0.0150 | 6.73E-07 |
| family | Verrucomicrobiaceae | rs4936098 | G | A | 0.0649 | 0.0136 | 1.13E-06 |
| family | Verrucomicrobiaceae | rs9349825 | G | A | -0.0704 | 0.0147 | 2.51E-06 |
| family | Verrucomicrobiaceae | rs74542928 | C | T | 0.1121 | 0.0236 | 1.65E-06 |
| family | Verrucomicrobiaceae | rs3995795 | T | C | 0.0642 | 0.0141 | 5.03E-06 |
| family | Verrucomicrobiaceae | rs2602429 | T | C | 0.0746 | 0.0156 | 2.70E-06 |
| family | Verrucomicrobiaceae | rs4242783 | A | G | 0.0688 | 0.0148 | 2.75E-06 |
| family | Verrucomicrobiaceae | rs11184341 | C | G | 0.0655 | 0.0142 | 4.14E-06 |
| family | Verrucomicrobiaceae | rs61779207 | A | G | -0.0759 | 0.0168 | 6.63E-06 |
| family | Verrucomicrobiaceae | rs117107102 | G | A | 0.2047 | 0.0432 | 2.92E-06 |
| family | Verrucomicrobiaceae | rs12908520 | A | G | 0.0619 | 0.0131 | 2.15E-06 |
| family | Verrucomicrobiaceae | rs111862613 | C | T | 0.0907 | 0.0197 | 3.73E-06 |
| family | Verrucomicrobiaceae | rs941682 | A | G | -0.0632 | 0.0144 | 9.58E-06 |
| family | Victivallaceae | rs4396289 | T | C | -0.1529 | 0.0289 | 1.54E-07 |
| family | Victivallaceae | rs2944282 | C | T | -0.1242 | 0.0257 | 1.57E-06 |
| family | Victivallaceae | rs11764871 | T | G | 0.1271 | 0.0257 | 7.49E-07 |
| family | Victivallaceae | rs6545794 | G | A | -0.1978 | 0.0410 | 5.97E-07 |
| family | Victivallaceae | rs2546105 | T | A | 0.1266 | 0.0257 | 1.03E-06 |
| family | Victivallaceae | rs62570196 | T | C | -0.2461 | 0.0483 | 2.70E-07 |
| family | Victivallaceae | rs7077363 | A | G | 0.1489 | 0.0318 | 2.83E-06 |
| family | Victivallaceae | rs67832247 | T | C | -0.1235 | 0.0267 | 3.24E-06 |
| family | Victivallaceae | rs34962571 | C | A | -0.1869 | 0.0418 | 6.25E-06 |
| family | Victivallaceae | rs7860510 | C | G | 0.1649 | 0.0375 | 9.60E-06 |
| family | Victivallaceae | rs7627405 | T | C | -0.1342 | 0.0301 | 8.19E-06 |
| family | Victivallaceae | rs61702987 | C | T | 0.1455 | 0.0300 | 3.08E-06 |
| family | Victivallaceae | rs7314815 | A | G | 0.1011 | 0.0226 | 6.40E-06 |
| family | Victivallaceae | rs11671100 | C | A | -0.1600 | 0.0349 | 4.08E-06 |
| genus | Clostridiuminnocuumgroup | rs6890185 | C | T | 0.1134 | 0.0233 | 1.12E-06 |
| genus | Clostridiuminnocuumgroup | rs61267978 | C | T | 0.1471 | 0.0321 | 5.59E-06 |
| genus | Clostridiuminnocuumgroup | rs1948423 | A | T | -0.1089 | 0.0234 | 3.49E-06 |
| genus | Clostridiuminnocuumgroup | rs6577484 | A | G | 0.1604 | 0.0361 | 8.41E-06 |
| genus | Clostridiuminnocuumgroup | rs10074000 | C | T | -0.1026 | 0.0228 | 7.00E-06 |
| genus | Clostridiuminnocuumgroup | rs1942371 | A | G | -0.1579 | 0.0342 | 4.06E-06 |
| genus | Clostridiuminnocuumgroup | rs40656 | T | C | 0.1427 | 0.0311 | 8.62E-06 |
| genus | Clostridiuminnocuumgroup | rs71564433 | A | T | -0.1267 | 0.0275 | 7.80E-06 |
| genus | Clostridiuminnocuumgroup | rs77845139 | G | A | -0.1150 | 0.0257 | 8.41E-06 |
| genus | Clostridiuminnocuumgroup | rs10506058 | G | A | 0.0997 | 0.0222 | 8.92E-06 |
| genus | Clostridiuminnocuumgroup | rs4869133 | A | G | -0.1806 | 0.0410 | 7.24E-06 |
| genus | Eubacteriumbrachygroup | rs112617308 | C | T | -0.1709 | 0.0363 | 2.38E-06 |
| genus | Eubacteriumbrachygroup | rs9613196 | A | T | -0.2387 | 0.0529 | 4.99E-06 |
| genus | Eubacteriumbrachygroup | rs62348779 | C | T | -0.2015 | 0.0433 | 3.78E-06 |
| genus | Eubacteriumbrachygroup | rs2913110 | T | C | 0.1051 | 0.0229 | 4.56E-06 |
| genus | Eubacteriumbrachygroup | rs4862235 | A | G | 0.1048 | 0.0226 | 3.73E-06 |
| genus | Eubacteriumbrachygroup | rs12151423 | G | A | 0.1013 | 0.0227 | 9.27E-06 |
| genus | Eubacteriumbrachygroup | rs73199919 | C | T | -0.2367 | 0.0531 | 8.16E-06 |
| genus | Eubacteriumbrachygroup | rs6591893 | A | G | 0.1082 | 0.0240 | 7.34E-06 |
| genus | Eubacteriumbrachygroup | rs720439 | G | A | -0.1119 | 0.0251 | 7.03E-06 |
| genus | Eubacteriumbrachygroup | rs13139592 | C | T | -0.1460 | 0.0327 | 7.97E-06 |
| genus | Eubacteriumbrachygroup | rs1384962 | G | A | 0.1209 | 0.0266 | 6.99E-06 |
| genus | Eubacteriumcoprostanoligenesgroup | rs17159861 | T | C | 0.0962 | 0.0168 | 1.04E-08 |
| genus | Eubacteriumcoprostanoligenesgroup | rs9648214 | C | T | -0.0829 | 0.0164 | 2.52E-07 |
| genus | Eubacteriumcoprostanoligenesgroup | rs115325767 | C | G | 0.0783 | 0.0175 | 2.62E-06 |
| genus | Eubacteriumcoprostanoligenesgroup | rs12906958 | T | C | -0.0533 | 0.0116 | 4.35E-06 |
| genus | Eubacteriumcoprostanoligenesgroup | rs4076415 | G | T | 0.0515 | 0.0110 | 1.99E-06 |
| genus | Eubacteriumcoprostanoligenesgroup | rs6762473 | A | C | 0.0522 | 0.0112 | 4.26E-06 |
| genus | Eubacteriumcoprostanoligenesgroup | rs73109678 | A | G | -0.0797 | 0.0172 | 3.66E-06 |
| genus | Eubacteriumcoprostanoligenesgroup | rs10444197 | G | A | -0.0506 | 0.0113 | 5.98E-06 |
| genus | Eubacteriumcoprostanoligenesgroup | rs2644213 | A | G | 0.0539 | 0.0121 | 9.86E-06 |
| genus | Eubacteriumcoprostanoligenesgroup | rs11720857 | T | C | 0.0631 | 0.0144 | 9.26E-06 |
| genus | Eubacteriumcoprostanoligenesgroup | rs4717831 | T | A | 0.0787 | 0.0174 | 9.18E-06 |
| genus | Eubacteriumcoprostanoligenesgroup | rs62024432 | T | C | -0.0770 | 0.0172 | 7.50E-06 |
| genus | Eubacteriumcoprostanoligenesgroup | rs76898927 | A | G | 0.1231 | 0.0266 | 4.79E-06 |
| genus | Eubacteriumcoprostanoligenesgroup | rs11052069 | C | T | 0.0478 | 0.0108 | 9.38E-06 |
| genus | Eubacteriumcoprostanoligenesgroup | rs79895140 | C | T | -0.0641 | 0.0141 | 8.62E-06 |
| genus | Eubacteriumeligensgroup | rs4583233 | C | A | 0.0670 | 0.0128 | 2.84E-07 |
| genus | Eubacteriumeligensgroup | rs265534 | G | T | -0.0564 | 0.0120 | 2.27E-06 |
| genus | Eubacteriumeligensgroup | rs72839198 | G | C | 0.1557 | 0.0376 | 3.14E-06 |
| genus | Eubacteriumeligensgroup | rs74606150 | G | C | -0.1965 | 0.0426 | 4.25E-06 |
| genus | Eubacteriumeligensgroup | rs6923695 | G | T | 0.1033 | 0.0230 | 4.87E-06 |
| genus | Eubacteriumeligensgroup | rs56080211 | T | C | 0.1231 | 0.0283 | 9.14E-06 |
| genus | Eubacteriumeligensgroup | rs158115 | C | G | 0.0915 | 0.0191 | 9.74E-06 |
| genus | Eubacteriumeligensgroup | rs2200429 | G | A | -0.0889 | 0.0198 | 5.30E-06 |
| genus | Eubacteriumeligensgroup | rs182318 | A | G | -0.0825 | 0.0196 | 8.40E-06 |
| genus | Eubacteriumeligensgroup | rs12719051 | G | A | 0.0917 | 0.0208 | 7.12E-06 |
| genus | Eubacteriumfissicatenagroup | rs3771393 | T | C | 0.1308 | 0.0267 | 7.38E-07 |
| genus | Eubacteriumfissicatenagroup | rs2733072 | A | G | 0.1096 | 0.0228 | 1.49E-06 |
| genus | Eubacteriumfissicatenagroup | rs7104872 | A | G | 0.1386 | 0.0292 | 2.73E-06 |
| genus | Eubacteriumfissicatenagroup | rs151257695 | G | A | 0.2095 | 0.0455 | 3.10E-06 |
| genus | Eubacteriumfissicatenagroup | rs11876297 | C | T | 0.1315 | 0.0282 | 2.67E-06 |
| genus | Eubacteriumfissicatenagroup | rs6934739 | G | A | 0.1115 | 0.0253 | 9.75E-06 |
| genus | Eubacteriumfissicatenagroup | rs10147907 | G | T | 0.1723 | 0.0396 | 8.27E-06 |
| genus | Eubacteriumfissicatenagroup | rs1768152 | C | T | 0.1395 | 0.0316 | 8.70E-06 |
| genus | Eubacteriumfissicatenagroup | rs11818408 | A | G | 0.1059 | 0.0237 | 8.20E-06 |
| genus | Eubacteriumhalliigroup | rs13116360 | C | T | 0.1541 | 0.0297 | 2.94E-07 |
| genus | Eubacteriumhalliigroup | rs949971 | G | T | -0.0540 | 0.0116 | 3.29E-06 |
| genus | Eubacteriumhalliigroup | rs10798999 | T | C | 0.0602 | 0.0127 | 2.61E-06 |
| genus | Eubacteriumhalliigroup | rs60254196 | G | A | -0.0523 | 0.0112 | 2.70E-06 |
| genus | Eubacteriumhalliigroup | rs10808115 | C | A | -0.0505 | 0.0110 | 4.42E-06 |
| genus | Eubacteriumhalliigroup | rs74018587 | T | C | 0.2089 | 0.0438 | 3.70E-06 |
| genus | Eubacteriumhalliigroup | rs6550770 | C | T | -0.1981 | 0.0444 | 4.82E-06 |
| genus | Eubacteriumhalliigroup | rs10501370 | T | C | -0.1156 | 0.0252 | 5.42E-06 |
| genus | Eubacteriumhalliigroup | rs281379 | G | A | -0.0500 | 0.0112 | 9.33E-06 |
| genus | Eubacteriumhalliigroup | rs78056098 | T | G | -0.0507 | 0.0114 | 8.29E-06 |
| genus | Eubacteriumhalliigroup | rs138531890 | G | A | 0.1531 | 0.0349 | 5.43E-06 |
| genus | Eubacteriumhalliigroup | rs28584818 | G | A | 0.1261 | 0.0269 | 4.43E-06 |
| genus | Eubacteriumhalliigroup | rs117748144 | C | T | -0.1266 | 0.0287 | 7.86E-06 |
| genus | Eubacteriumhalliigroup | rs17474256 | A | G | 0.0811 | 0.0185 | 9.45E-06 |
| genus | Eubacteriumhalliigroup | rs630939 | T | C | -0.0509 | 0.0114 | 9.16E-06 |
| genus | Eubacteriumhalliigroup | rs17074066 | C | T | -0.0814 | 0.0189 | 9.35E-06 |
| genus | Eubacteriumnodatumgroup | rs34297067 | G | A | -0.1869 | 0.0341 | 6.60E-08 |
| genus | Eubacteriumnodatumgroup | rs113893692 | T | C | -0.1851 | 0.0404 | 5.76E-06 |
| genus | Eubacteriumnodatumgroup | rs77910827 | T | C | 0.2018 | 0.0414 | 9.05E-07 |
| genus | Eubacteriumnodatumgroup | rs61841040 | T | G | 0.1606 | 0.0342 | 3.56E-06 |
| genus | Eubacteriumnodatumgroup | rs9425984 | C | T | -0.1302 | 0.0292 | 7.21E-06 |
| genus | Eubacteriumnodatumgroup | rs10263623 | T | C | 0.1935 | 0.0439 | 8.91E-06 |
| genus | Eubacteriumnodatumgroup | rs11006576 | G | A | -0.1102 | 0.0246 | 7.99E-06 |
| genus | Eubacteriumnodatumgroup | rs7880204 | C | T | -0.1255 | 0.0275 | 6.84E-06 |
| genus | Eubacteriumnodatumgroup | rs6818880 | G | A | -0.1101 | 0.0246 | 7.83E-06 |
| genus | Eubacteriumnodatumgroup | rs10458299 | C | T | -0.1878 | 0.0420 | 8.37E-06 |
| genus | Eubacteriumnodatumgroup | rs7827125 | T | C | 0.1223 | 0.0271 | 7.17E-06 |
| genus | Eubacteriumoxidoreducensgroup | rs12423772 | T | G | 0.1410 | 0.0295 | 2.63E-06 |
| genus | Eubacteriumoxidoreducensgroup | rs2973294 | T | G | 0.0924 | 0.0195 | 2.39E-06 |
| genus | Eubacteriumoxidoreducensgroup | rs34561138 | A | G | 0.2161 | 0.0460 | 2.51E-06 |
| genus | Eubacteriumoxidoreducensgroup | rs440215 | T | C | 0.0933 | 0.0195 | 1.65E-06 |
| genus | Eubacteriumoxidoreducensgroup | rs1425962 | C | G | 0.0906 | 0.0201 | 7.32E-06 |
| genus | Eubacteriumoxidoreducensgroup | rs12129908 | A | C | 0.0893 | 0.0198 | 5.80E-06 |
| genus | Eubacteriumrectalegroup | rs35398954 | G | A | -0.0901 | 0.0175 | 5.40E-07 |
| genus | Eubacteriumrectalegroup | rs117151453 | C | G | -0.1131 | 0.0244 | 1.84E-06 |
| genus | Eubacteriumrectalegroup | rs16960159 | G | C | -0.1566 | 0.0336 | 4.10E-06 |
| genus | Eubacteriumrectalegroup | rs314726 | C | T | 0.0529 | 0.0109 | 1.38E-06 |
| genus | Eubacteriumrectalegroup | rs10797540 | G | A | 0.0503 | 0.0108 | 3.53E-06 |
| genus | Eubacteriumrectalegroup | rs10248854 | A | C | -0.0528 | 0.0113 | 4.21E-06 |
| genus | Eubacteriumrectalegroup | rs3980709 | T | A | -0.0623 | 0.0141 | 6.86E-06 |
| genus | Eubacteriumrectalegroup | rs2884897 | G | A | -0.1294 | 0.0289 | 6.44E-06 |
| genus | Eubacteriumrectalegroup | rs10892089 | G | C | -0.0636 | 0.0140 | 6.22E-06 |
| genus | Eubacteriumrectalegroup | rs143694765 | C | T | 0.0870 | 0.0198 | 9.75E-06 |
| genus | Eubacteriumrectalegroup | rs59427698 | G | A | -0.0576 | 0.0131 | 5.37E-06 |
| genus | Eubacteriumrectalegroup | rs62547233 | G | A | 0.0536 | 0.0120 | 9.90E-06 |
| genus | Eubacteriumrectalegroup | rs58498416 | T | C | -0.0517 | 0.0118 | 9.68E-06 |
| genus | Eubacteriumruminantiumgroup | rs2116427 | G | A | 0.0911 | 0.0182 | 4.67E-07 |
| genus | Eubacteriumruminantiumgroup | rs139749 | T | C | -0.0845 | 0.0172 | 8.59E-07 |
| genus | Eubacteriumruminantiumgroup | rs72836424 | T | C | -0.1398 | 0.0301 | 2.62E-06 |
| genus | Eubacteriumruminantiumgroup | rs2229917 | G | A | 0.1535 | 0.0324 | 2.16E-06 |
| genus | Eubacteriumruminantiumgroup | rs10131724 | C | A | -0.1998 | 0.0415 | 2.39E-06 |
| genus | Eubacteriumruminantiumgroup | rs16891896 | A | G | -0.1748 | 0.0391 | 2.38E-06 |
| genus | Eubacteriumruminantiumgroup | rs7000472 | G | A | -0.0762 | 0.0165 | 4.07E-06 |
| genus | Eubacteriumruminantiumgroup | rs13025464 | C | T | -0.0737 | 0.0164 | 6.97E-06 |
| genus | Eubacteriumruminantiumgroup | rs2418654 | T | C | -0.0749 | 0.0166 | 6.17E-06 |
| genus | Eubacteriumruminantiumgroup | rs6676699 | T | G | -0.0888 | 0.0196 | 6.38E-06 |
| genus | Eubacteriumruminantiumgroup | rs73139629 | C | A | -0.1151 | 0.0248 | 5.36E-06 |
| genus | Eubacteriumruminantiumgroup | rs112375806 | A | T | 0.1431 | 0.0294 | 5.82E-06 |
| genus | Eubacteriumruminantiumgroup | rs606117 | G | A | 0.0833 | 0.0181 | 4.82E-06 |
| genus | Eubacteriumruminantiumgroup | rs57340348 | C | T | -0.0979 | 0.0212 | 4.93E-06 |
| genus | Eubacteriumruminantiumgroup | rs2817174 | T | C | -0.0734 | 0.0164 | 7.87E-06 |
| genus | Eubacteriumruminantiumgroup | rs10923018 | A | G | 0.0726 | 0.0161 | 6.80E-06 |
| genus | Eubacteriumruminantiumgroup | rs11637981 | T | G | -0.0733 | 0.0161 | 5.44E-06 |
| genus | Eubacteriumruminantiumgroup | rs17519472 | T | C | 0.1078 | 0.0234 | 4.70E-06 |
| genus | Eubacteriumruminantiumgroup | rs209813 | A | G | -0.1035 | 0.0236 | 9.23E-06 |
| genus | Eubacteriumventriosumgroup | rs6048195 | T | A | -0.0604 | 0.0117 | 2.50E-07 |
| genus | Eubacteriumventriosumgroup | rs73615400 | C | T | -0.0956 | 0.0193 | 9.54E-07 |
| genus | Eubacteriumventriosumgroup | rs57199565 | C | T | 0.0783 | 0.0160 | 7.97E-07 |
| genus | Eubacteriumventriosumgroup | rs11617697 | G | A | -0.1433 | 0.0286 | 7.22E-07 |
| genus | Eubacteriumventriosumgroup | rs16884680 | T | G | -0.0906 | 0.0192 | 1.74E-06 |
| genus | Eubacteriumventriosumgroup | rs12964517 | A | G | 0.0587 | 0.0123 | 2.07E-06 |
| genus | Eubacteriumventriosumgroup | rs876734 | T | C | -0.0619 | 0.0133 | 2.89E-06 |
| genus | Eubacteriumventriosumgroup | rs3809430 | C | T | -0.0548 | 0.0118 | 3.55E-06 |
| genus | Eubacteriumventriosumgroup | rs72783037 | A | C | 0.0659 | 0.0144 | 6.55E-06 |
| genus | Eubacteriumventriosumgroup | rs73849225 | C | T | 0.0976 | 0.0224 | 5.21E-06 |
| genus | Eubacteriumventriosumgroup | rs9316536 | G | T | -0.0817 | 0.0183 | 7.84E-06 |
| genus | Eubacteriumventriosumgroup | rs35179274 | T | C | -0.0627 | 0.0138 | 5.76E-06 |
| genus | Eubacteriumventriosumgroup | rs78250280 | A | G | 0.0750 | 0.0164 | 3.36E-06 |
| genus | Eubacteriumventriosumgroup | rs66746423 | T | C | 0.0752 | 0.0165 | 6.11E-06 |
| genus | Eubacteriumventriosumgroup | rs6704822 | G | A | 0.0739 | 0.0167 | 6.62E-06 |
| genus | Eubacteriumventriosumgroup | rs13082419 | T | C | -0.0716 | 0.0161 | 9.56E-06 |
| genus | Eubacteriumventriosumgroup | rs66830358 | A | T | 0.0529 | 0.0118 | 6.87E-06 |
| genus | Eubacteriumxylanophilumgroup | rs17830032 | A | G | -0.1606 | 0.0311 | 2.39E-07 |
| genus | Eubacteriumxylanophilumgroup | rs13239072 | A | G | 0.0687 | 0.0143 | 1.82E-06 |
| genus | Eubacteriumxylanophilumgroup | rs79582700 | G | C | -0.0952 | 0.0200 | 2.41E-06 |
| genus | Eubacteriumxylanophilumgroup | rs12980122 | G | C | -0.1083 | 0.0238 | 5.02E-06 |
| genus | Eubacteriumxylanophilumgroup | rs2012708 | G | A | 0.0573 | 0.0127 | 6.53E-06 |
| genus | Eubacteriumxylanophilumgroup | rs2213117 | G | T | 0.0878 | 0.0189 | 4.21E-06 |
| genus | Eubacteriumxylanophilumgroup | rs10140184 | C | A | 0.0577 | 0.0126 | 4.96E-06 |
| genus | Eubacteriumxylanophilumgroup | rs10917203 | C | A | 0.0613 | 0.0131 | 3.15E-06 |
| genus | Eubacteriumxylanophilumgroup | rs112176119 | T | C | -0.1135 | 0.0246 | 3.33E-06 |
| genus | Eubacteriumxylanophilumgroup | rs1999224 | T | G | -0.0949 | 0.0204 | 3.75E-06 |
| genus | Eubacteriumxylanophilumgroup | rs75586835 | G | A | -0.1145 | 0.0263 | 9.39E-06 |
| genus | Eubacteriumxylanophilumgroup | rs4654122 | G | C | 0.0554 | 0.0124 | 7.20E-06 |
| genus | Ruminococcusgauvreauiigroup | rs2047242 | G | A | -0.0676 | 0.0134 | 2.46E-07 |
| genus | Ruminococcusgauvreauiigroup | rs71386687 | G | T | 0.1210 | 0.0239 | 2.91E-07 |
| genus | Ruminococcusgauvreauiigroup | rs9870933 | G | A | 0.0622 | 0.0126 | 8.49E-07 |
| genus | Ruminococcusgauvreauiigroup | rs1391597 | T | C | 0.0590 | 0.0125 | 1.86E-06 |
| genus | Ruminococcusgauvreauiigroup | rs431418 | G | A | -0.0947 | 0.0210 | 5.54E-06 |
| genus | Ruminococcusgauvreauiigroup | rs10931481 | A | G | 0.0610 | 0.0130 | 3.38E-06 |
| genus | Ruminococcusgauvreauiigroup | rs289410 | A | G | -0.0655 | 0.0139 | 2.27E-06 |
| genus | Ruminococcusgauvreauiigroup | rs2105937 | G | A | 0.0580 | 0.0128 | 5.10E-06 |
| genus | Ruminococcusgauvreauiigroup | rs2166943 | C | A | 0.0567 | 0.0123 | 5.28E-06 |
| genus | Ruminococcusgauvreauiigroup | rs12539819 | T | C | 0.1107 | 0.0241 | 4.49E-06 |
| genus | Ruminococcusgauvreauiigroup | rs12079579 | G | A | 0.0955 | 0.0213 | 5.04E-06 |
| genus | Ruminococcusgauvreauiigroup | rs73802842 | A | C | 0.0737 | 0.0170 | 7.48E-06 |
| genus | Ruminococcusgauvreauiigroup | rs13188803 | A | T | 0.0709 | 0.0157 | 7.28E-06 |
| genus | Ruminococcusgnavusgroup | rs13163520 | A | G | -0.1274 | 0.0234 | 5.61E-08 |
| genus | Ruminococcusgnavusgroup | rs934940 | C | A | -0.1050 | 0.0230 | 2.74E-06 |
| genus | Ruminococcusgnavusgroup | rs9872758 | C | T | 0.0849 | 0.0177 | 1.66E-06 |
| genus | Ruminococcusgnavusgroup | rs2909242 | A | C | -0.0910 | 0.0184 | 7.41E-07 |
| genus | Ruminococcusgnavusgroup | rs3124783 | G | A | -0.1160 | 0.0249 | 2.67E-06 |
| genus | Ruminococcusgnavusgroup | rs62167033 | C | T | 0.1853 | 0.0396 | 3.50E-06 |
| genus | Ruminococcusgnavusgroup | rs12989336 | A | G | -0.0847 | 0.0188 | 7.12E-06 |
| genus | Ruminococcusgnavusgroup | rs12136548 | T | C | 0.0902 | 0.0196 | 3.10E-06 |
| genus | Ruminococcusgnavusgroup | rs78399089 | C | T | 0.1444 | 0.0327 | 6.63E-06 |
| genus | Ruminococcusgnavusgroup | rs11597105 | G | A | 0.1147 | 0.0251 | 6.95E-06 |
| genus | Ruminococcusgnavusgroup | rs11864644 | C | T | -0.1398 | 0.0318 | 5.01E-06 |
| genus | Ruminococcusgnavusgroup | rs4388134 | T | C | -0.0905 | 0.0204 | 9.12E-06 |
| genus | Ruminococcustorquesgroup | rs35866622 | C | T | -0.0612 | 0.0109 | 2.21E-08 |
| genus | Ruminococcustorquesgroup | rs773123 | A | T | 0.0824 | 0.0174 | 1.59E-06 |
| genus | Ruminococcustorquesgroup | rs10904297 | G | A | -0.1678 | 0.0390 | 2.69E-06 |
| genus | Ruminococcustorquesgroup | rs8080469 | A | G | 0.0491 | 0.0107 | 3.50E-06 |
| genus | Ruminococcustorquesgroup | rs77034621 | G | T | -0.1516 | 0.0336 | 6.07E-06 |
| genus | Ruminococcustorquesgroup | rs73130967 | T | A | 0.0770 | 0.0168 | 3.71E-06 |
| genus | Ruminococcustorquesgroup | rs1972694 | A | T | -0.0614 | 0.0137 | 8.93E-06 |
| genus | Ruminococcustorquesgroup | rs12434631 | G | A | 0.0747 | 0.0153 | 2.77E-06 |
| genus | Ruminococcustorquesgroup | rs158487 | G | A | 0.0532 | 0.0116 | 7.52E-06 |
| genus | Ruminococcustorquesgroup | rs4073731 | C | T | 0.0652 | 0.0142 | 4.05E-06 |
| genus | Ruminococcustorquesgroup | rs60603763 | G | C | 0.1243 | 0.0275 | 5.87E-06 |
| genus | Ruminococcustorquesgroup | rs13154778 | A | T | 0.0563 | 0.0130 | 7.16E-06 |
| genus | Ruminococcustorquesgroup | rs1475330 | C | T | 0.0523 | 0.0118 | 8.13E-06 |
| genus | Ruminococcustorquesgroup | rs10967781 | A | C | 0.0508 | 0.0113 | 8.37E-06 |
| genus | Ruminococcustorquesgroup | rs8141465 | G | A | 0.0481 | 0.0107 | 9.65E-06 |
| genus | Actinomyces | rs34583783 | T | G | 0.1266 | 0.0268 | 4.49E-06 |
| genus | Actinomyces | rs2715439 | T | C | 0.0747 | 0.0165 | 6.27E-06 |
| genus | Actinomyces | rs4146653 | A | G | 0.0985 | 0.0214 | 4.50E-06 |
| genus | Actinomyces | rs35011108 | G | A | 0.2326 | 0.0512 | 6.34E-06 |
| genus | Actinomyces | rs4073240 | A | G | 0.0750 | 0.0167 | 7.94E-06 |
| genus | Actinomyces | rs7915461 | C | T | 0.1878 | 0.0402 | 5.92E-06 |
| genus | Actinomyces | rs71315246 | G | A | -0.0970 | 0.0219 | 9.83E-06 |
| genus | Actinomyces | rs10787984 | C | G | 0.0943 | 0.0214 | 9.62E-06 |
| genus | Adlercreutzia | rs7680684 | T | C | -0.0834 | 0.0169 | 9.77E-07 |
| genus | Adlercreutzia | rs2147798 | G | C | 0.0923 | 0.0192 | 1.40E-06 |
| genus | Adlercreutzia | rs80078995 | T | A | -0.1132 | 0.0233 | 1.57E-06 |
| genus | Adlercreutzia | rs2717140 | T | C | -0.1192 | 0.0251 | 2.05E-06 |
| genus | Adlercreutzia | rs9490822 | T | C | -0.0735 | 0.0156 | 2.54E-06 |
| genus | Adlercreutzia | rs12522517 | T | A | -0.1049 | 0.0235 | 4.41E-06 |
| genus | Adlercreutzia | rs13231526 | A | C | 0.1432 | 0.0312 | 4.81E-06 |
| genus | Adlercreutzia | rs6664405 | C | T | -0.0953 | 0.0211 | 5.23E-06 |
| genus | Adlercreutzia | rs1046175 | G | C | 0.1128 | 0.0256 | 6.36E-06 |
| genus | Adlercreutzia | rs9915817 | C | T | 0.0749 | 0.0168 | 8.22E-06 |
| genus | Adlercreutzia | rs55719207 | A | G | -0.0699 | 0.0158 | 9.61E-06 |
| genus | Adlercreutzia | rs11604400 | T | C | -0.1025 | 0.0235 | 9.74E-06 |
| genus | Akkermansia | rs11729256 | C | T | 0.0750 | 0.0150 | 6.58E-07 |
| genus | Akkermansia | rs4936098 | G | A | 0.0649 | 0.0136 | 1.10E-06 |
| genus | Akkermansia | rs9349825 | G | A | -0.0703 | 0.0147 | 2.60E-06 |
| genus | Akkermansia | rs74542928 | C | T | 0.1126 | 0.0236 | 1.48E-06 |
| genus | Akkermansia | rs3995795 | T | C | 0.0641 | 0.0141 | 5.22E-06 |
| genus | Akkermansia | rs2602429 | T | C | 0.0745 | 0.0156 | 2.72E-06 |
| genus | Akkermansia | rs61779207 | A | G | -0.0761 | 0.0168 | 6.32E-06 |
| genus | Akkermansia | rs11184341 | C | G | 0.0656 | 0.0142 | 4.06E-06 |
| genus | Akkermansia | rs4242783 | A | G | 0.0685 | 0.0148 | 3.00E-06 |
| genus | Akkermansia | rs117107102 | G | A | 0.2044 | 0.0432 | 3.01E-06 |
| genus | Akkermansia | rs12908520 | A | G | 0.0618 | 0.0131 | 2.26E-06 |
| genus | Akkermansia | rs111862613 | C | T | 0.0911 | 0.0197 | 3.39E-06 |
| genus | Akkermansia | rs941682 | A | G | -0.0633 | 0.0144 | 9.17E-06 |
| genus | Bacteroides | rs6795673 | T | C | 0.0539 | 0.0105 | 3.38E-07 |
| genus | Bacteroides | rs28757219 | A | T | 0.0818 | 0.0170 | 1.29E-06 |
| genus | Bacteroides | rs9507307 | T | C | 0.0604 | 0.0129 | 2.13E-06 |
| genus | Bacteroides | rs66474973 | T | G | 0.0813 | 0.0164 | 6.81E-07 |
| genus | Bacteroides | rs11585893 | G | A | -0.0741 | 0.0148 | 1.80E-06 |
| genus | Bacteroides | rs495004 | G | C | -0.0607 | 0.0130 | 3.42E-06 |
| genus | Bacteroides | rs17619981 | G | T | 0.0881 | 0.0187 | 2.69E-06 |
| genus | Bacteroides | rs2023437 | C | T | -0.0782 | 0.0168 | 5.02E-06 |
| genus | Bacteroides | rs66710942 | T | C | 0.0488 | 0.0107 | 5.86E-06 |
| genus | Bacteroides | rs13207588 | G | A | -0.0592 | 0.0131 | 7.49E-06 |
| genus | Bacteroides | rs2366421 | A | T | -0.0528 | 0.0117 | 7.65E-06 |
| genus | Bacteroides | rs1340391 | C | T | -0.0592 | 0.0132 | 6.73E-06 |
| genus | Barnesiella | rs2276875 | G | A | -0.0697 | 0.0140 | 4.65E-07 |
| genus | Barnesiella | rs2428166 | A | G | -0.1659 | 0.0337 | 8.51E-07 |
| genus | Barnesiella | rs60316894 | T | C | -0.1215 | 0.0252 | 1.19E-06 |
| genus | Barnesiella | rs35177866 | G | A | 0.0917 | 0.0190 | 2.95E-06 |
| genus | Barnesiella | rs13242616 | C | T | -0.0584 | 0.0123 | 2.29E-06 |
| genus | Barnesiella | rs79795328 | G | A | -0.0819 | 0.0176 | 4.23E-06 |
| genus | Barnesiella | rs2057922 | C | G | 0.0915 | 0.0195 | 3.83E-06 |
| genus | Barnesiella | rs199035 | A | G | 0.0559 | 0.0120 | 3.00E-06 |
| genus | Barnesiella | rs62251337 | G | A | -0.0691 | 0.0149 | 4.24E-06 |
| genus | Barnesiella | rs77455852 | G | T | -0.0891 | 0.0196 | 3.16E-06 |
| genus | Barnesiella | rs11155559 | C | T | 0.0956 | 0.0213 | 8.92E-06 |
| genus | Barnesiella | rs12909713 | T | C | -0.0551 | 0.0120 | 4.95E-06 |
| genus | Barnesiella | rs113258194 | G | A | 0.0990 | 0.0214 | 7.31E-06 |
| genus | Barnesiella | rs28479800 | T | A | -0.0915 | 0.0210 | 7.93E-06 |
| genus | Barnesiella | rs76181748 | T | C | -0.0779 | 0.0172 | 6.78E-06 |
| genus | Barnesiella | rs72684847 | C | T | -0.1144 | 0.0254 | 6.76E-06 |
| genus | Barnesiella | rs28418786 | G | C | -0.0791 | 0.0175 | 6.48E-06 |
| genus | Bifidobacterium | rs182549 | T | C | 0.1197 | 0.0127 | 1.28E-20 |
| genus | Bifidobacterium | rs7322849 | C | T | 0.1124 | 0.0202 | 1.08E-08 |
| genus | Bifidobacterium | rs1961273 | T | C | 0.0674 | 0.0132 | 3.51E-07 |
| genus | Bifidobacterium | rs10841473 | C | G | -0.0624 | 0.0129 | 1.65E-06 |
| genus | Bifidobacterium | rs56108664 | C | T | 0.0730 | 0.0158 | 2.44E-06 |
| genus | Bifidobacterium | rs2686790 | C | T | 0.0707 | 0.0158 | 7.50E-06 |
| genus | Bifidobacterium | rs4567981 | A | T | 0.0562 | 0.0118 | 1.93E-06 |
| genus | Bifidobacterium | rs2491158 | A | G | 0.0713 | 0.0160 | 8.05E-06 |
| genus | Bifidobacterium | rs76671854 | G | C | -0.0846 | 0.0184 | 3.96E-06 |
| genus | Bifidobacterium | rs75344046 | T | C | 0.2324 | 0.0506 | 4.86E-06 |
| genus | Bifidobacterium | rs540489 | G | T | -0.0638 | 0.0139 | 5.19E-06 |
| genus | Bifidobacterium | rs857444 | T | C | 0.0558 | 0.0121 | 3.57E-06 |
| genus | Bifidobacterium | rs13020688 | A | G | 0.0563 | 0.0123 | 4.07E-06 |
| genus | Bifidobacterium | rs12022129 | A | G | 0.0619 | 0.0139 | 8.00E-06 |
| genus | Bifidobacterium | rs62181700 | A | G | -0.0625 | 0.0131 | 2.17E-06 |
| genus | Bifidobacterium | rs55888705 | G | A | 0.0546 | 0.0121 | 6.67E-06 |
| genus | Bifidobacterium | rs5746486 | C | T | -0.0536 | 0.0121 | 9.00E-06 |
| genus | Bifidobacterium | rs73797465 | G | T | -0.0954 | 0.0209 | 4.38E-06 |
| genus | Bilophila | rs116261629 | C | G | 0.1281 | 0.0261 | 8.62E-07 |
| genus | Bilophila | rs1571225 | T | C | 0.0827 | 0.0171 | 1.12E-06 |
| genus | Bilophila | rs3827020 | T | C | 0.0766 | 0.0161 | 1.79E-06 |
| genus | Bilophila | rs1241171 | A | G | -0.0693 | 0.0150 | 4.24E-06 |
| genus | Bilophila | rs7802841 | A | C | 0.0670 | 0.0138 | 1.77E-06 |
| genus | Bilophila | rs8013541 | T | A | -0.0572 | 0.0126 | 5.48E-06 |
| genus | Bilophila | rs6793291 | A | C | 0.1127 | 0.0242 | 3.11E-06 |
| genus | Bilophila | rs1917709 | T | A | 0.1185 | 0.0267 | 7.37E-06 |
| genus | Bilophila | rs542415 | C | T | -0.0614 | 0.0133 | 4.71E-06 |
| genus | Bilophila | rs72676854 | C | T | 0.1232 | 0.0269 | 5.62E-06 |
| genus | Bilophila | rs4798126 | A | G | 0.0733 | 0.0168 | 7.15E-06 |
| genus | Bilophila | rs1969927 | A | G | 0.0565 | 0.0127 | 9.07E-06 |
| genus | Bilophila | rs60178956 | A | G | -0.0625 | 0.0141 | 8.06E-06 |
| genus | Bilophila | rs2728491 | T | G | -0.0627 | 0.0139 | 6.33E-06 |
| genus | Bilophila | rs2713349 | T | A | 0.0617 | 0.0140 | 8.63E-06 |
| genus | Bilophila | rs9899990 | G | A | -0.1027 | 0.0234 | 9.07E-06 |
| genus | Bilophila | rs11069458 | C | T | -0.0681 | 0.0155 | 7.72E-06 |
| genus | Blautia | rs11149971 | T | C | 0.1176 | 0.0234 | 1.04E-06 |
| genus | Blautia | rs12453000 | T | C | 0.0625 | 0.0130 | 1.26E-06 |
| genus | Blautia | rs115043014 | A | G | -0.2066 | 0.0440 | 5.19E-06 |
| genus | Blautia | rs67794373 | T | C | 0.0602 | 0.0123 | 1.00E-06 |
| genus | Blautia | rs117001700 | C | T | 0.1964 | 0.0441 | 8.84E-06 |
| genus | Blautia | rs72973581 | G | A | 0.1252 | 0.0265 | 1.74E-06 |
| genus | Blautia | rs4926264 | C | T | 0.0826 | 0.0178 | 5.10E-06 |
| genus | Blautia | rs7860714 | G | A | -0.0502 | 0.0110 | 4.09E-06 |
| genus | Blautia | rs682885 | G | A | -0.0493 | 0.0107 | 4.49E-06 |
| genus | Blautia | rs2788271 | G | T | -0.0576 | 0.0133 | 7.16E-06 |
| genus | Blautia | rs113271346 | T | C | 0.0783 | 0.0172 | 6.85E-06 |
| genus | Blautia | rs3005511 | G | A | 0.0501 | 0.0111 | 6.19E-06 |
| genus | Blautia | rs16892041 | C | T | -0.0623 | 0.0142 | 8.82E-06 |
| genus | Butyricicoccus | rs56221232 | C | T | 0.0828 | 0.0167 | 7.62E-07 |
| genus | Butyricicoccus | rs2017189 | T | G | -0.0507 | 0.0110 | 3.87E-06 |
| genus | Butyricicoccus | rs7322368 | C | T | 0.0816 | 0.0183 | 5.52E-06 |
| genus | Butyricicoccus | rs75238760 | A | T | 0.0619 | 0.0140 | 6.80E-06 |
| genus | Butyricicoccus | rs12585793 | C | T | -0.2622 | 0.0565 | 5.79E-06 |
| genus | Butyricicoccus | rs62478070 | G | T | 0.2240 | 0.0495 | 5.94E-06 |
| genus | Butyricicoccus | rs4962426 | T | G | 0.0614 | 0.0136 | 7.38E-06 |
| genus | Butyricicoccus | rs10084203 | G | A | 0.0550 | 0.0124 | 8.59E-06 |
| genus | Butyricicoccus | rs12034718 | G | A | 0.0701 | 0.0158 | 9.58E-06 |
| genus | Butyricimonas | rs113054641 | A | G | -0.1449 | 0.0275 | 1.74E-07 |
| genus | Butyricimonas | rs62390301 | C | T | -0.0873 | 0.0175 | 7.42E-07 |
| genus | Butyricimonas | rs7083431 | C | A | 0.0704 | 0.0144 | 8.85E-07 |
| genus | Butyricimonas | rs2642760 | C | G | 0.0713 | 0.0145 | 8.58E-07 |
| genus | Butyricimonas | rs782080 | A | T | 0.0653 | 0.0138 | 2.23E-06 |
| genus | Butyricimonas | rs1862649 | A | G | 0.1131 | 0.0248 | 4.76E-06 |
| genus | Butyricimonas | rs12304031 | A | G | -0.0863 | 0.0197 | 6.70E-06 |
| genus | Butyricimonas | rs1701950 | C | G | -0.1051 | 0.0241 | 7.68E-06 |
| genus | Butyricimonas | rs71428626 | T | G | -0.1332 | 0.0290 | 4.80E-06 |
| genus | Butyricimonas | rs78453362 | G | A | -0.1495 | 0.0327 | 4.06E-06 |
| genus | Butyricimonas | rs326049 | G | C | 0.0760 | 0.0168 | 8.19E-06 |
| genus | Butyricimonas | rs62130338 | A | G | -0.0733 | 0.0158 | 3.90E-06 |
| genus | Butyricimonas | rs9657374 | T | C | 0.0681 | 0.0148 | 4.50E-06 |
| genus | Butyricimonas | rs12458763 | C | A | 0.1220 | 0.0270 | 6.37E-06 |
| genus | Butyricimonas | rs2114713 | T | G | 0.0627 | 0.0139 | 6.88E-06 |
| genus | Butyricimonas | rs270727 | C | G | -0.0694 | 0.0151 | 5.38E-06 |
| genus | Butyricimonas | rs72814525 | G | A | 0.0664 | 0.0150 | 8.25E-06 |
| genus | Butyricimonas | rs11228830 | G | A | 0.1353 | 0.0298 | 6.55E-06 |
| genus | Butyrivibrio | rs72723662 | T | C | 0.2241 | 0.0449 | 7.86E-07 |
| genus | Butyrivibrio | rs7412979 | G | C | 0.1868 | 0.0389 | 1.71E-06 |
| genus | Butyrivibrio | rs7752361 | G | A | -0.1192 | 0.0240 | 7.69E-07 |
| genus | Butyrivibrio | rs4537857 | C | T | -0.1246 | 0.0261 | 1.80E-06 |
| genus | Butyrivibrio | rs11761679 | C | T | 0.1548 | 0.0322 | 2.20E-06 |
| genus | Butyrivibrio | rs16941336 | T | C | 0.1275 | 0.0268 | 1.53E-06 |
| genus | Butyrivibrio | rs77356209 | C | T | 0.2169 | 0.0483 | 6.66E-06 |
| genus | Butyrivibrio | rs74622183 | G | A | -0.2010 | 0.0428 | 2.46E-06 |
| genus | Butyrivibrio | rs7763512 | A | G | 0.1198 | 0.0253 | 3.11E-06 |
| genus | Butyrivibrio | rs142855850 | G | A | 0.2050 | 0.0457 | 6.86E-06 |
| genus | Butyrivibrio | rs9349693 | G | A | 0.1180 | 0.0260 | 5.55E-06 |
| genus | Butyrivibrio | rs486484 | G | A | -0.1083 | 0.0240 | 6.61E-06 |
| genus | Butyrivibrio | rs17163238 | A | G | 0.1410 | 0.0309 | 5.51E-06 |
| genus | Butyrivibrio | rs4928024 | G | A | -0.1747 | 0.0389 | 8.19E-06 |
| genus | Butyrivibrio | rs1007475 | T | G | 0.1181 | 0.0261 | 7.92E-06 |
| genus | Butyrivibrio | rs16934069 | C | T | -0.1338 | 0.0299 | 8.86E-06 |
| genus | CandidatusSoleaferrea | rs4294381 | C | T | 0.1122 | 0.0232 | 1.37E-06 |
| genus | CandidatusSoleaferrea | rs10090365 | G | A | -0.0834 | 0.0181 | 4.17E-06 |
| genus | CandidatusSoleaferrea | rs4678258 | C | T | 0.0986 | 0.0216 | 5.53E-06 |
| genus | CandidatusSoleaferrea | rs10108780 | G | A | -0.0928 | 0.0200 | 3.64E-06 |
| genus | CandidatusSoleaferrea | rs386526 | G | C | 0.0818 | 0.0180 | 8.33E-06 |
| genus | CandidatusSoleaferrea | rs36155147 | T | C | 0.1050 | 0.0241 | 5.41E-06 |
| genus | CandidatusSoleaferrea | rs6881988 | C | G | -0.0819 | 0.0182 | 9.23E-06 |
| genus | CandidatusSoleaferrea | rs11153159 | C | G | -0.1281 | 0.0285 | 4.42E-06 |
| genus | CandidatusSoleaferrea | rs10809135 | C | T | 0.0835 | 0.0182 | 5.47E-06 |
| genus | CandidatusSoleaferrea | rs9973954 | G | A | 0.0892 | 0.0195 | 5.95E-06 |
| genus | CandidatusSoleaferrea | rs6489992 | G | A | -0.0840 | 0.0187 | 7.89E-06 |
| genus | CandidatusSoleaferrea | rs6494306 | G | A | -0.0969 | 0.0214 | 5.80E-06 |
| genus | CandidatusSoleaferrea | rs12500231 | T | A | 0.0814 | 0.0183 | 7.68E-06 |
| genus | CandidatusSoleaferrea | rs7400877 | C | T | -0.0951 | 0.0213 | 9.29E-06 |
| genus | CandidatusSoleaferrea | rs2193878 | A | T | 0.2283 | 0.0509 | 9.46E-06 |
| genus | CandidatusSoleaferrea | rs830149 | G | C | 0.1846 | 0.0397 | 9.58E-06 |
| genus | Catenibacterium | rs12404911 | T | C | 0.1407 | 0.0304 | 2.80E-06 |
| genus | Catenibacterium | rs77285108 | A | G | -0.1618 | 0.0353 | 3.63E-06 |
| genus | Catenibacterium | rs73128290 | G | A | 0.1297 | 0.0285 | 4.29E-06 |
| genus | Catenibacterium | rs212393 | A | G | -0.1353 | 0.0286 | 3.62E-06 |
| genus | Catenibacterium | rs7742829 | T | C | 0.1141 | 0.0251 | 5.61E-06 |
| genus | ChristensenellaceaeR | rs999354 | A | T | 0.0580 | 0.0117 | 7.01E-07 |
| genus | ChristensenellaceaeR | rs78521377 | T | C | 0.1250 | 0.0275 | 5.61E-06 |
| genus | ChristensenellaceaeR | rs62467127 | T | C | 0.1141 | 0.0252 | 3.25E-06 |
| genus | ChristensenellaceaeR | rs17081797 | G | A | -0.0904 | 0.0204 | 3.34E-06 |
| genus | ChristensenellaceaeR | rs62132810 | G | A | -0.0829 | 0.0180 | 5.67E-06 |
| genus | ChristensenellaceaeR | rs60954665 | G | T | 0.0498 | 0.0111 | 7.13E-06 |
| genus | ChristensenellaceaeR | rs10461257 | G | A | -0.0552 | 0.0122 | 6.51E-06 |
| genus | ChristensenellaceaeR | rs79150079 | A | C | 0.1215 | 0.0271 | 9.42E-06 |
| genus | ChristensenellaceaeR | rs62190261 | C | A | 0.0958 | 0.0215 | 8.74E-06 |
| genus | ChristensenellaceaeR | rs892686 | G | A | 0.0514 | 0.0111 | 3.97E-06 |
| genus | ChristensenellaceaeR | rs73952017 | T | C | -0.0862 | 0.0194 | 8.46E-06 |
| genus | Clostridiumsensustricto1 | rs550843 | C | T | -0.0783 | 0.0169 | 2.05E-06 |
| genus | Clostridiumsensustricto1 | rs2795528 | A | G | -0.1843 | 0.0392 | 2.72E-06 |
| genus | Clostridiumsensustricto1 | rs2817172 | T | C | 0.0581 | 0.0124 | 2.77E-06 |
| genus | Clostridiumsensustricto1 | rs115807074 | G | A | -0.2274 | 0.0493 | 4.32E-06 |
| genus | Clostridiumsensustricto1 | rs116847295 | T | C | 0.1100 | 0.0246 | 4.58E-06 |
| genus | Clostridiumsensustricto1 | rs12341505 | A | G | 0.0811 | 0.0180 | 4.82E-06 |
| genus | Clostridiumsensustricto1 | rs11586026 | T | A | 0.1110 | 0.0250 | 8.85E-06 |
| genus | Clostridiumsensustricto1 | rs11264403 | A | G | -0.1391 | 0.0334 | 7.76E-06 |
| genus | Clostridiumsensustricto1 | rs12490337 | G | C | -0.0617 | 0.0138 | 7.49E-06 |
| genus | Collinsella | rs9541268 | A | C | 0.0960 | 0.0197 | 8.79E-07 |
| genus | Collinsella | rs2671662 | G | C | -0.0568 | 0.0119 | 2.22E-06 |
| genus | Collinsella | rs73052258 | A | G | 0.0930 | 0.0203 | 1.72E-06 |
| genus | Collinsella | rs2103510 | A | G | 0.0787 | 0.0168 | 2.42E-06 |
| genus | Collinsella | rs75672793 | G | A | -0.1089 | 0.0241 | 6.14E-06 |
| genus | Collinsella | rs10890671 | C | T | -0.0537 | 0.0119 | 6.52E-06 |
| genus | Collinsella | rs12921100 | T | A | 0.0564 | 0.0127 | 8.23E-06 |
| genus | Collinsella | rs59414781 | G | C | 0.0669 | 0.0150 | 9.15E-06 |
| genus | Collinsella | rs62448871 | A | C | -0.0540 | 0.0120 | 6.78E-06 |
| genus | Collinsella | rs1496626 | C | T | -0.0722 | 0.0162 | 6.78E-06 |
| genus | Collinsella | rs149807560 | A | C | -0.1043 | 0.0236 | 7.10E-06 |
| genus | Collinsella | rs11597285 | T | G | -0.0538 | 0.0121 | 9.38E-06 |
| genus | Coprobacter | rs305411 | G | A | 0.1292 | 0.0265 | 1.01E-06 |
| genus | Coprobacter | rs3828477 | T | G | -0.0912 | 0.0196 | 2.89E-06 |
| genus | Coprobacter | rs55672356 | A | T | -0.1934 | 0.0414 | 2.74E-06 |
| genus | Coprobacter | rs143662916 | T | C | 0.2533 | 0.0540 | 3.07E-06 |
| genus | Coprobacter | rs213863 | T | C | -0.0887 | 0.0188 | 2.35E-06 |
| genus | Coprobacter | rs72821405 | C | T | -0.1474 | 0.0320 | 4.76E-06 |
| genus | Coprobacter | rs11532348 | T | C | -0.1039 | 0.0227 | 5.71E-06 |
| genus | Coprobacter | rs12996055 | C | A | 0.0922 | 0.0209 | 8.08E-06 |
| genus | Coprobacter | rs12684609 | C | T | 0.1008 | 0.0220 | 6.10E-06 |
| genus | Coprobacter | rs5011652 | C | G | 0.0900 | 0.0200 | 5.51E-06 |
| genus | Coprobacter | rs74919520 | A | G | 0.1257 | 0.0276 | 5.76E-06 |
| genus | Coprobacter | rs28402691 | C | T | 0.1107 | 0.0251 | 9.56E-06 |
| genus | Coprobacter | rs189356 | A | G | 0.0781 | 0.0172 | 6.26E-06 |
| genus | Coprobacter | rs76001613 | G | C | 0.2159 | 0.0493 | 9.13E-06 |
| genus | Coprococcus1 | rs4277593 | A | G | -0.0586 | 0.0110 | 1.14E-07 |
| genus | Coprococcus1 | rs56405618 | G | A | -0.0896 | 0.0187 | 1.57E-06 |
| genus | Coprococcus1 | rs74101919 | C | T | -0.0719 | 0.0145 | 1.03E-06 |
| genus | Coprococcus1 | rs1010560 | A | C | 0.0580 | 0.0123 | 1.96E-06 |
| genus | Coprococcus1 | rs73031725 | C | T | 0.1676 | 0.0355 | 1.98E-06 |
| genus | Coprococcus1 | rs1519491 | C | T | 0.0499 | 0.0114 | 8.95E-06 |
| genus | Coprococcus1 | rs1576241 | G | A | -0.0510 | 0.0110 | 3.33E-06 |
| genus | Coprococcus1 | rs73167075 | C | T | 0.0573 | 0.0128 | 8.57E-06 |
| genus | Coprococcus1 | rs12794898 | T | G | 0.0903 | 0.0197 | 4.92E-06 |
| genus | Coprococcus1 | rs946513 | T | C | 0.2059 | 0.0460 | 8.62E-06 |
| genus | Coprococcus1 | rs1762123 | T | C | -0.0892 | 0.0199 | 8.01E-06 |
| genus | Coprococcus1 | rs7784490 | G | C | -0.0519 | 0.0113 | 4.55E-06 |
| genus | Coprococcus1 | rs12886051 | C | G | -0.0522 | 0.0118 | 8.01E-06 |
| genus | Coprococcus1 | rs2907920 | G | A | 0.0561 | 0.0127 | 7.65E-06 |
| genus | Coprococcus2 | rs59936925 | T | A | 0.1171 | 0.0234 | 9.38E-07 |
| genus | Coprococcus2 | rs6677933 | T | C | -0.0804 | 0.0164 | 1.19E-06 |
| genus | Coprococcus2 | rs1958519 | A | T | 0.0665 | 0.0139 | 1.58E-06 |
| genus | Coprococcus2 | rs72680320 | C | T | -0.0649 | 0.0139 | 2.27E-06 |
| genus | Coprococcus2 | rs2482516 | T | C | 0.0754 | 0.0165 | 4.72E-06 |
| genus | Coprococcus2 | rs35890118 | G | A | -0.0665 | 0.0148 | 8.26E-06 |
| genus | Coprococcus2 | rs10121347 | G | C | 0.0926 | 0.0219 | 8.31E-06 |
| genus | Coprococcus2 | rs9426473 | G | A | 0.0727 | 0.0162 | 6.31E-06 |
| genus | Coprococcus2 | rs6894272 | C | T | -0.1135 | 0.0253 | 9.53E-06 |
| genus | Coprococcus2 | rs12634070 | C | T | 0.0736 | 0.0165 | 9.95E-06 |
| genus | Coprococcus2 | rs61823518 | C | A | -0.0955 | 0.0216 | 6.68E-06 |
| genus | Coprococcus2 | rs10070053 | G | A | 0.0594 | 0.0135 | 7.65E-06 |
| genus | Coprococcus3 | rs8100692 | C | T | 0.0577 | 0.0113 | 4.16E-07 |
| genus | Coprococcus3 | rs62481985 | C | G | -0.0582 | 0.0115 | 4.18E-07 |
| genus | Coprococcus3 | rs178271 | C | T | 0.1453 | 0.0294 | 7.81E-07 |
| genus | Coprococcus3 | rs13394391 | T | C | -0.0709 | 0.0151 | 2.20E-06 |
| genus | Coprococcus3 | rs11080344 | T | C | 0.0517 | 0.0113 | 4.79E-06 |
| genus | Coprococcus3 | rs7521171 | A | G | -0.0596 | 0.0129 | 4.32E-06 |
| genus | Coprococcus3 | rs11077359 | C | T | -0.0645 | 0.0149 | 9.64E-06 |
| genus | Coprococcus3 | rs6994742 | T | C | 0.0551 | 0.0124 | 7.84E-06 |
| genus | Coprococcus3 | rs13247359 | A | G | 0.0512 | 0.0113 | 7.33E-06 |
| genus | Coprococcus3 | rs4575475 | A | G | 0.0620 | 0.0138 | 7.04E-06 |
| genus | Coprococcus3 | rs10810043 | G | A | 0.0516 | 0.0116 | 9.27E-06 |
| genus | DefluviitaleaceaeUCG011 | rs4677103 | G | A | 0.0978 | 0.0197 | 9.60E-07 |
| genus | DefluviitaleaceaeUCG011 | rs72731813 | T | C | -0.1474 | 0.0294 | 4.33E-07 |
| genus | DefluviitaleaceaeUCG011 | rs112893842 | C | T | 0.1138 | 0.0233 | 1.45E-06 |
| genus | DefluviitaleaceaeUCG011 | rs9725395 | G | A | -0.1383 | 0.0296 | 3.52E-06 |
| genus | DefluviitaleaceaeUCG011 | rs55658617 | C | T | 0.1744 | 0.0362 | 2.15E-06 |
| genus | DefluviitaleaceaeUCG011 | rs9608282 | G | T | 0.1429 | 0.0300 | 2.52E-06 |
| genus | DefluviitaleaceaeUCG011 | rs28696126 | T | A | -0.1065 | 0.0238 | 6.63E-06 |
| genus | DefluviitaleaceaeUCG011 | rs1582238 | C | T | 0.0805 | 0.0167 | 1.57E-06 |
| genus | DefluviitaleaceaeUCG011 | rs4344384 | T | G | 0.0716 | 0.0156 | 4.83E-06 |
| genus | DefluviitaleaceaeUCG011 | rs2892880 | A | G | 0.0818 | 0.0182 | 6.83E-06 |
| genus | Desulfovibrio | rs11160353 | A | T | -0.0692 | 0.0147 | 2.70E-06 |
| genus | Desulfovibrio | rs2853179 | T | C | 0.0812 | 0.0174 | 2.42E-06 |
| genus | Desulfovibrio | rs16863365 | G | A | 0.1094 | 0.0227 | 1.79E-06 |
| genus | Desulfovibrio | rs12031543 | C | T | -0.1272 | 0.0282 | 6.55E-06 |
| genus | Desulfovibrio | rs13066142 | A | G | 0.1191 | 0.0251 | 3.79E-06 |
| genus | Desulfovibrio | rs2590913 | A | G | 0.1545 | 0.0339 | 6.65E-06 |
| genus | Desulfovibrio | rs2032031 | G | A | -0.0655 | 0.0149 | 9.14E-06 |
| genus | Desulfovibrio | rs72647089 | G | T | -0.1066 | 0.0239 | 8.30E-06 |
| genus | Desulfovibrio | rs7729080 | A | C | -0.0703 | 0.0158 | 9.96E-06 |
| genus | Desulfovibrio | rs4797774 | A | G | 0.2126 | 0.0470 | 5.64E-06 |
| genus | Desulfovibrio | rs6580353 | C | T | 0.0771 | 0.0170 | 4.94E-06 |
| genus | Dialister | rs11166701 | A | G | -0.0655 | 0.0132 | 5.51E-07 |
| genus | Dialister | rs517089 | A | T | 0.0762 | 0.0170 | 5.14E-06 |
| genus | Dialister | rs4747450 | A | C | 0.0669 | 0.0148 | 5.84E-06 |
| genus | Dialister | rs2314294 | C | T | 0.0866 | 0.0194 | 8.08E-06 |
| genus | Dialister | rs4753063 | A | G | -0.0596 | 0.0130 | 4.86E-06 |
| genus | Dialister | rs2435610 | C | A | 0.0647 | 0.0143 | 5.93E-06 |
| genus | Dialister | rs10938938 | A | G | -0.0774 | 0.0171 | 7.37E-06 |
| genus | Dialister | rs10138457 | C | T | -0.1131 | 0.0262 | 7.88E-06 |
| genus | Dialister | rs11071887 | C | T | 0.0662 | 0.0146 | 5.91E-06 |
| genus | Dialister | rs764177 | A | C | -0.0601 | 0.0135 | 9.61E-06 |
| genus | Dialister | rs76680460 | A | G | -0.1613 | 0.0364 | 8.19E-06 |
| genus | Dialister | rs75416973 | G | A | 0.0727 | 0.0165 | 9.46E-06 |
| genus | Dorea | rs13279148 | A | G | 0.0715 | 0.0151 | 2.25E-06 |
| genus | Dorea | rs62503162 | G | A | -0.0974 | 0.0194 | 7.47E-07 |
| genus | Dorea | rs12216169 | A | T | 0.0882 | 0.0194 | 5.33E-06 |
| genus | Dorea | rs73729431 | T | C | -0.1375 | 0.0300 | 3.17E-06 |
| genus | Dorea | rs4793307 | T | C | 0.0574 | 0.0122 | 4.01E-06 |
| genus | Dorea | rs11150408 | G | T | 0.0488 | 0.0109 | 7.06E-06 |
| genus | Dorea | rs345219 | G | T | -0.0497 | 0.0113 | 8.80E-06 |
| genus | Dorea | rs62583469 | A | T | -0.0634 | 0.0142 | 5.78E-06 |
| genus | Dorea | rs3005511 | G | A | 0.0516 | 0.0113 | 5.29E-06 |
| genus | Dorea | rs1899291 | T | C | 0.0697 | 0.0150 | 4.57E-06 |
| genus | Dorea | rs3752849 | A | G | 0.1638 | 0.0366 | 7.68E-06 |
| genus | Dorea | rs12537781 | C | T | -0.0555 | 0.0125 | 9.15E-06 |
| genus | Eggerthella | rs3851328 | G | T | -0.1078 | 0.0237 | 4.18E-06 |
| genus | Eggerthella | rs2240838 | G | A | 0.0981 | 0.0198 | 7.36E-07 |
| genus | Eggerthella | rs2223081 | A | G | 0.1026 | 0.0221 | 3.89E-06 |
| genus | Eggerthella | rs112205261 | C | T | -0.1886 | 0.0404 | 3.35E-06 |
| genus | Eggerthella | rs4985746 | A | G | 0.1105 | 0.0248 | 5.71E-06 |
| genus | Eggerthella | rs1784446 | A | G | 0.0908 | 0.0198 | 5.23E-06 |
| genus | Eggerthella | rs76663501 | T | C | 0.1753 | 0.0379 | 4.83E-06 |
| genus | Eggerthella | rs6430926 | T | C | 0.0880 | 0.0197 | 8.37E-06 |
| genus | Eggerthella | rs2877457 | A | G | -0.0935 | 0.0210 | 9.03E-06 |
| genus | Eggerthella | rs13070736 | C | A | -0.1213 | 0.0272 | 7.62E-06 |
| genus | Eggerthella | rs67490567 | C | T | 0.1085 | 0.0245 | 8.94E-06 |
| genus | Eisenbergiella | rs3812426 | A | G | 0.1064 | 0.0224 | 2.72E-06 |
| genus | Eisenbergiella | rs1508033 | C | A | 0.0915 | 0.0196 | 3.23E-06 |
| genus | Eisenbergiella | rs12278566 | A | T | -0.1211 | 0.0252 | 1.65E-06 |
| genus | Eisenbergiella | rs2683098 | T | C | 0.1073 | 0.0225 | 2.24E-06 |
| genus | Eisenbergiella | rs11079158 | C | T | 0.1006 | 0.0225 | 7.35E-06 |
| genus | Eisenbergiella | rs13258851 | G | A | 0.1370 | 0.0302 | 7.75E-06 |
| genus | Eisenbergiella | rs12710729 | A | C | 0.0893 | 0.0199 | 9.84E-06 |
| genus | Eisenbergiella | rs12257723 | C | A | -0.0953 | 0.0212 | 8.85E-06 |
| genus | Eisenbergiella | rs4462860 | A | G | 0.0939 | 0.0201 | 4.16E-06 |
| genus | Eisenbergiella | rs11027642 | T | C | 0.1290 | 0.0285 | 4.92E-06 |
| genus | Eisenbergiella | rs11938607 | C | T | 0.0978 | 0.0217 | 8.22E-06 |
| genus | Eisenbergiella | rs1553971 | G | T | 0.1210 | 0.0263 | 5.27E-06 |
| genus | Enterorhabdus | rs11098863 | A | T | -0.0966 | 0.0163 | 3.06E-09 |
| genus | Enterorhabdus | rs7923280 | T | A | 0.0860 | 0.0169 | 5.24E-07 |
| genus | Enterorhabdus | rs114731706 | G | T | 0.1823 | 0.0382 | 2.17E-06 |
| genus | Enterorhabdus | rs3017103 | G | A | 0.0981 | 0.0209 | 2.94E-06 |
| genus | Enterorhabdus | rs73331712 | C | T | 0.2620 | 0.0551 | 4.85E-06 |
| genus | Enterorhabdus | rs77655283 | A | G | 0.1330 | 0.0298 | 5.88E-06 |
| genus | Enterorhabdus | rs9470637 | T | A | -0.0756 | 0.0166 | 5.70E-06 |
| genus | Enterorhabdus | rs424715 | C | T | 0.0819 | 0.0175 | 4.41E-06 |
| genus | Enterorhabdus | rs10098492 | C | T | 0.1323 | 0.0294 | 6.41E-06 |
| genus | Enterorhabdus | rs2051957 | T | C | 0.0843 | 0.0190 | 8.90E-06 |
| genus | Erysipelatoclostridium | rs7221249 | G | A | 0.0840 | 0.0143 | 4.31E-09 |
| genus | Erysipelatoclostridium | rs710230 | C | T | 0.1434 | 0.0282 | 6.33E-07 |
| genus | Erysipelatoclostridium | rs4697572 | G | A | -0.0811 | 0.0163 | 7.59E-07 |
| genus | Erysipelatoclostridium | rs58236560 | T | G | -0.1112 | 0.0234 | 2.16E-06 |
| genus | Erysipelatoclostridium | rs622418 | G | A | -0.0668 | 0.0143 | 3.68E-06 |
| genus | Erysipelatoclostridium | rs17804233 | C | T | -0.0663 | 0.0144 | 4.59E-06 |
| genus | Erysipelatoclostridium | rs1434153 | A | G | -0.0685 | 0.0152 | 6.85E-06 |
| genus | Erysipelatoclostridium | rs340991 | G | A | -0.0740 | 0.0159 | 3.75E-06 |
| genus | Erysipelatoclostridium | rs6474512 | C | A | 0.0670 | 0.0143 | 3.02E-06 |
| genus | Erysipelatoclostridium | rs9590927 | A | G | -0.0646 | 0.0143 | 6.39E-06 |
| genus | Erysipelatoclostridium | rs61806970 | T | C | 0.1425 | 0.0321 | 9.09E-06 |
| genus | Erysipelatoclostridium | rs45480394 | G | T | -0.0687 | 0.0152 | 7.66E-06 |
| genus | Erysipelatoclostridium | rs2901723 | A | C | 0.0641 | 0.0144 | 8.79E-06 |
| genus | Erysipelatoclostridium | rs34528142 | G | C | -0.0876 | 0.0196 | 6.13E-06 |
| genus | Erysipelatoclostridium | rs16936671 | T | C | -0.0968 | 0.0218 | 6.04E-06 |
| genus | Erysipelatoclostridium | rs3804326 | G | A | 0.1415 | 0.0336 | 9.85E-06 |
| genus | ErysipelotrichaceaeUCG003 | rs28568391 | G | A | -0.0584 | 0.0119 | 6.42E-07 |
| genus | ErysipelotrichaceaeUCG003 | rs11994308 | T | C | 0.1154 | 0.0243 | 1.33E-06 |
| genus | ErysipelotrichaceaeUCG003 | rs76502207 | C | T | 0.1448 | 0.0290 | 6.41E-07 |
| genus | ErysipelotrichaceaeUCG003 | rs2302840 | C | T | -0.0872 | 0.0177 | 1.29E-06 |
| genus | ErysipelotrichaceaeUCG003 | rs10164067 | G | T | -0.1033 | 0.0212 | 1.13E-06 |
| genus | ErysipelotrichaceaeUCG003 | rs8053479 | G | A | -0.0838 | 0.0187 | 5.83E-06 |
| genus | ErysipelotrichaceaeUCG003 | rs17798136 | A | G | 0.1588 | 0.0348 | 3.24E-06 |
| genus | ErysipelotrichaceaeUCG003 | rs11666127 | G | A | -0.0719 | 0.0161 | 7.90E-06 |
| genus | ErysipelotrichaceaeUCG003 | rs62403464 | C | T | -0.0732 | 0.0157 | 3.44E-06 |
| genus | ErysipelotrichaceaeUCG003 | rs59068084 | G | T | 0.0565 | 0.0120 | 3.12E-06 |
| genus | ErysipelotrichaceaeUCG003 | rs73074432 | T | C | 0.0722 | 0.0164 | 9.99E-06 |
| genus | ErysipelotrichaceaeUCG003 | rs214586 | C | T | -0.1065 | 0.0240 | 8.50E-06 |
| genus | ErysipelotrichaceaeUCG003 | rs4758231 | T | G | -0.0552 | 0.0122 | 6.55E-06 |
| genus | ErysipelotrichaceaeUCG003 | rs74988980 | A | G | -0.1331 | 0.0349 | 8.64E-06 |
| genus | ErysipelotrichaceaeUCG003 | rs75949021 | C | T | -0.1697 | 0.0374 | 3.58E-06 |
| genus | ErysipelotrichaceaeUCG003 | rs6875357 | T | C | 0.1657 | 0.0354 | 6.70E-06 |
| genus | ErysipelotrichaceaeUCG003 | rs79396538 | G | C | 0.0848 | 0.0191 | 8.63E-06 |
| genus | ErysipelotrichaceaeUCG003 | rs12251396 | G | A | -0.0705 | 0.0159 | 9.52E-06 |
| genus | Escherichia | rs2267739 | C | G | 0.1155 | 0.0238 | 1.42E-06 |
| genus | Escherichia | rs1154904 | G | A | -0.0613 | 0.0131 | 3.04E-06 |
| genus | Escherichia | rs73208162 | G | A | -0.1193 | 0.0248 | 2.19E-06 |
| genus | Escherichia | rs113513883 | G | A | 0.1723 | 0.0380 | 5.28E-06 |
| genus | Escherichia | rs7502686 | C | G | -0.1364 | 0.0303 | 5.90E-06 |
| genus | Escherichia | rs57024273 | C | T | 0.0626 | 0.0140 | 9.70E-06 |
| genus | Escherichia | rs113127095 | G | A | 0.1510 | 0.0323 | 3.33E-06 |
| genus | Escherichia | rs117092367 | T | A | 0.1174 | 0.0265 | 9.65E-06 |
| genus | Escherichia | rs112767262 | C | T | 0.0733 | 0.0164 | 8.21E-06 |
| genus | Escherichia | rs11706043 | A | T | 0.0757 | 0.0164 | 5.87E-06 |
| genus | Escherichia | rs592299 | C | T | -0.0592 | 0.0129 | 4.77E-06 |
| genus | Escherichia | rs4731451 | A | G | -0.0610 | 0.0135 | 7.47E-06 |
| genus | Escherichia | rs35555519 | G | C | 0.1020 | 0.0223 | 4.92E-06 |
| genus | Escherichia | rs118526 | A | C | -0.0594 | 0.0136 | 8.00E-06 |
| genus | Escherichia | rs2798105 | G | A | -0.1009 | 0.0222 | 8.25E-06 |
| genus | Faecalibacterium | rs12320842 | G | C | 0.0948 | 0.0164 | 7.57E-09 |
| genus | Faecalibacterium | rs6910935 | G | A | 0.1349 | 0.0277 | 1.38E-06 |
| genus | Faecalibacterium | rs1271565 | T | C | -0.0576 | 0.0120 | 1.30E-06 |
| genus | Faecalibacterium | rs75499067 | T | C | 0.2276 | 0.0466 | 1.76E-06 |
| genus | Faecalibacterium | rs9536330 | C | T | -0.0483 | 0.0108 | 5.33E-06 |
| genus | Faecalibacterium | rs28376661 | G | C | 0.0505 | 0.0109 | 3.66E-06 |
| genus | Faecalibacterium | rs10927394 | T | G | -0.2323 | 0.0512 | 7.02E-06 |
| genus | Faecalibacterium | rs114946999 | T | C | -0.0862 | 0.0190 | 5.70E-06 |
| genus | Faecalibacterium | rs79656633 | C | T | 0.1456 | 0.0323 | 8.14E-06 |
| genus | Faecalibacterium | rs61875484 | G | C | 0.0818 | 0.0184 | 9.18E-06 |
| genus | Faecalibacterium | rs12753492 | C | A | 0.0641 | 0.0150 | 8.80E-06 |
| genus | Faecalibacterium | rs2835874 | C | T | -0.0866 | 0.0196 | 7.54E-06 |
| genus | Faecalibacterium | rs11776390 | C | T | -0.0784 | 0.0172 | 6.40E-06 |
| genus | FamilyXIIIAD3011group | rs9852893 | G | C | 0.0658 | 0.0129 | 3.88E-07 |
| genus | FamilyXIIIAD3011group | rs62200412 | T | C | -0.0801 | 0.0164 | 5.80E-07 |
| genus | FamilyXIIIAD3011group | rs16840310 | G | A | -0.0608 | 0.0122 | 6.75E-07 |
| genus | FamilyXIIIAD3011group | rs72730932 | A | C | -0.0900 | 0.0177 | 6.89E-07 |
| genus | FamilyXIIIAD3011group | rs16940167 | T | C | 0.0733 | 0.0160 | 3.91E-06 |
| genus | FamilyXIIIAD3011group | rs17156849 | A | G | -0.1129 | 0.0245 | 4.19E-06 |
| genus | FamilyXIIIAD3011group | rs62029761 | G | A | 0.1288 | 0.0276 | 3.89E-06 |
| genus | FamilyXIIIAD3011group | rs11736617 | A | G | -0.0759 | 0.0172 | 9.02E-06 |
| genus | FamilyXIIIAD3011group | rs12812672 | C | T | -0.0961 | 0.0208 | 2.56E-06 |
| genus | FamilyXIIIAD3011group | rs9276029 | G | A | -0.0811 | 0.0186 | 8.93E-06 |
| genus | FamilyXIIIAD3011group | rs12911842 | T | A | -0.0812 | 0.0183 | 6.91E-06 |
| genus | FamilyXIIIAD3011group | rs149302 | C | T | -0.0646 | 0.0143 | 7.48E-06 |
| genus | FamilyXIIIAD3011group | rs9837139 | G | A | 0.1075 | 0.0240 | 8.71E-06 |
| genus | FamilyXIIIAD3011group | rs739451 | T | C | 0.0650 | 0.0148 | 7.88E-06 |
| genus | FamilyXIIIAD3011group | rs11126423 | T | C | 0.0904 | 0.0196 | 5.91E-06 |
| genus | FamilyXIIIUCG001 | rs1426266 | C | T | -0.0666 | 0.0137 | 1.25E-06 |
| genus | FamilyXIIIUCG001 | rs12049454 | C | T | -0.0647 | 0.0134 | 1.17E-06 |
| genus | FamilyXIIIUCG001 | rs3842897 | A | G | -0.1126 | 0.0243 | 5.20E-06 |
| genus | FamilyXIIIUCG001 | rs116979587 | A | T | -0.1217 | 0.0261 | 3.05E-06 |
| genus | FamilyXIIIUCG001 | rs62414802 | T | C | -0.0612 | 0.0135 | 4.29E-06 |
| genus | FamilyXIIIUCG001 | rs2276529 | G | C | -0.0759 | 0.0165 | 5.36E-06 |
| genus | FamilyXIIIUCG001 | rs112362903 | G | A | -0.1490 | 0.0333 | 7.88E-06 |
| genus | FamilyXIIIUCG001 | rs8076666 | G | A | 0.0887 | 0.0198 | 8.02E-06 |
| genus | FamilyXIIIUCG001 | rs7119679 | A | G | -0.0809 | 0.0175 | 3.52E-06 |
| genus | FamilyXIIIUCG001 | rs76463770 | G | A | 0.1931 | 0.0420 | 3.77E-06 |
| genus | Flavonifractor | rs12030302 | G | A | -0.0692 | 0.0137 | 5.61E-07 |
| genus | Flavonifractor | rs806808 | C | T | 0.0667 | 0.0137 | 1.18E-06 |
| genus | Flavonifractor | rs34066017 | G | A | 0.0764 | 0.0160 | 1.52E-06 |
| genus | Flavonifractor | rs11811696 | C | T | -0.1161 | 0.0241 | 2.07E-06 |
| genus | Flavonifractor | rs11642826 | C | G | 0.1469 | 0.0325 | 6.65E-06 |
| genus | Flavonifractor | rs114873521 | T | C | -0.1301 | 0.0294 | 7.13E-06 |
| genus | Flavonifractor | rs6761463 | C | G | -0.0834 | 0.0185 | 8.11E-06 |
| genus | Flavonifractor | rs798674 | C | G | 0.0637 | 0.0142 | 7.02E-06 |
| genus | Flavonifractor | rs12038887 | G | C | 0.0943 | 0.0212 | 9.37E-06 |
| genus | Fusicatenibacter | rs4378146 | C | A | -0.0617 | 0.0125 | 7.20E-07 |
| genus | Fusicatenibacter | rs62353480 | G | A | -0.0701 | 0.0146 | 1.57E-06 |
| genus | Fusicatenibacter | rs704418 | C | T | 0.0739 | 0.0151 | 7.77E-07 |
| genus | Fusicatenibacter | rs2132128 | A | G | -0.0772 | 0.0160 | 1.08E-06 |
| genus | Fusicatenibacter | rs206581 | G | A | -0.0568 | 0.0128 | 8.96E-06 |
| genus | Fusicatenibacter | rs62187631 | C | T | -0.0711 | 0.0159 | 4.55E-06 |
| genus | Fusicatenibacter | rs2025938 | A | G | -0.0967 | 0.0205 | 2.99E-06 |
| genus | Fusicatenibacter | rs7069626 | C | T | 0.0509 | 0.0111 | 5.42E-06 |
| genus | Fusicatenibacter | rs3303 | C | T | -0.0954 | 0.0204 | 3.94E-06 |
| genus | Fusicatenibacter | rs2039204 | A | T | -0.0497 | 0.0108 | 3.94E-06 |
| genus | Fusicatenibacter | rs8028026 | G | A | -0.0792 | 0.0181 | 8.06E-06 |
| genus | Fusicatenibacter | rs1864685 | C | A | -0.0495 | 0.0108 | 4.96E-06 |
| genus | Fusicatenibacter | rs792108 | C | T | -0.0508 | 0.0114 | 8.50E-06 |
| genus | Fusicatenibacter | rs60254196 | G | A | -0.0492 | 0.0109 | 5.47E-06 |
| genus | Fusicatenibacter | rs9905659 | A | G | -0.0616 | 0.0137 | 7.31E-06 |
| genus | Fusicatenibacter | rs8063430 | C | T | -0.1040 | 0.0222 | 4.93E-06 |
| genus | Fusicatenibacter | rs6515626 | A | G | 0.1416 | 0.0314 | 7.29E-06 |
| genus | Fusicatenibacter | rs10439674 | G | A | -0.0572 | 0.0130 | 7.68E-06 |
| genus | Fusicatenibacter | rs167879 | T | C | -0.0660 | 0.0149 | 5.87E-06 |
| genus | Fusicatenibacter | rs73103914 | G | A | -0.0597 | 0.0134 | 8.30E-06 |
| genus | Gordonibacter | rs76287110 | T | A | -0.2430 | 0.0466 | 1.67E-07 |
| genus | Gordonibacter | rs7294633 | T | C | 0.1287 | 0.0250 | 3.44E-07 |
| genus | Gordonibacter | rs7220558 | T | A | 0.1169 | 0.0235 | 6.71E-07 |
| genus | Gordonibacter | rs71545975 | G | A | -0.1540 | 0.0339 | 7.04E-06 |
| genus | Gordonibacter | rs35042269 | A | C | -0.1803 | 0.0403 | 8.11E-06 |
| genus | Gordonibacter | rs72714787 | A | C | 0.1814 | 0.0377 | 1.43E-06 |
| genus | Gordonibacter | rs322296 | A | G | 0.1787 | 0.0377 | 4.02E-06 |
| genus | Gordonibacter | rs72939513 | G | A | -0.2140 | 0.0491 | 7.98E-06 |
| genus | Gordonibacter | rs3765837 | G | T | -0.1907 | 0.0434 | 7.17E-06 |
| genus | Gordonibacter | rs13412653 | C | A | 0.1076 | 0.0239 | 8.61E-06 |
| genus | Gordonibacter | rs117347059 | C | G | -0.1283 | 0.0285 | 9.17E-06 |
| genus | Gordonibacter | rs16955299 | A | G | -0.1964 | 0.0434 | 6.37E-06 |
| genus | Gordonibacter | rs4596722 | G | A | 0.1029 | 0.0232 | 9.06E-06 |
| genus | Gordonibacter | rs768830 | A | G | 0.1499 | 0.0333 | 7.76E-06 |
| genus | Gordonibacter | rs61934597 | T | C | -0.1725 | 0.0389 | 8.37E-06 |
| genus | Haemophilus | rs12191680 | G | C | 0.1065 | 0.0200 | 1.47E-07 |
| genus | Haemophilus | rs9382510 | T | C | -0.0935 | 0.0173 | 7.12E-08 |
| genus | Haemophilus | rs9574096 | T | A | -0.0736 | 0.0155 | 2.18E-06 |
| genus | Haemophilus | rs76022354 | T | C | 0.2446 | 0.0506 | 1.83E-06 |
| genus | Haemophilus | rs111582866 | A | G | -0.1243 | 0.0260 | 1.27E-06 |
| genus | Haemophilus | rs9895850 | C | T | -0.1930 | 0.0417 | 2.14E-06 |
| genus | Haemophilus | rs35509 | A | G | 0.1282 | 0.0269 | 2.01E-06 |
| genus | Haemophilus | rs78909003 | C | T | -0.2463 | 0.0504 | 1.67E-06 |
| genus | Haemophilus | rs9328464 | C | T | 0.0723 | 0.0149 | 1.42E-06 |
| genus | Haemophilus | rs4822728 | C | T | 0.0706 | 0.0151 | 3.48E-06 |
| genus | Haemophilus | rs10840326 | G | C | -0.0677 | 0.0151 | 7.37E-06 |
| genus | Haemophilus | rs12876183 | A | T | 0.0749 | 0.0167 | 9.62E-06 |
| genus | Haemophilus | rs10781340 | A | G | 0.0949 | 0.0203 | 4.32E-06 |
| genus | Haemophilus | rs56310940 | C | G | -0.1084 | 0.0247 | 7.23E-06 |
| genus | Holdemanella | rs761624 | G | C | 0.0960 | 0.0180 | 1.38E-07 |
| genus | Holdemanella | rs607782 | C | T | -0.0854 | 0.0173 | 7.19E-07 |
| genus | Holdemanella | rs75764681 | C | T | -0.2831 | 0.0599 | 1.94E-06 |
| genus | Holdemanella | rs73011279 | C | T | -0.0962 | 0.0199 | 1.36E-06 |
| genus | Holdemanella | rs4541991 | C | T | -0.0927 | 0.0194 | 2.10E-06 |
| genus | Holdemanella | rs1830029 | G | C | -0.0954 | 0.0211 | 5.35E-06 |
| genus | Holdemanella | rs12513188 | A | G | 0.0904 | 0.0195 | 4.65E-06 |
| genus | Holdemanella | rs12415649 | C | G | 0.0839 | 0.0191 | 7.88E-06 |
| genus | Holdemanella | rs1926302 | A | G | -0.1080 | 0.0231 | 7.50E-06 |
| genus | Holdemanella | rs17586763 | C | T | -0.2273 | 0.0510 | 7.72E-06 |
| genus | Holdemanella | rs8113760 | A | G | 0.0790 | 0.0173 | 4.62E-06 |
| genus | Holdemanella | rs34187114 | A | C | -0.1045 | 0.0226 | 5.13E-06 |
| genus | Holdemanella | rs35228298 | A | G | 0.0935 | 0.0203 | 7.30E-06 |
| genus | Holdemanella | rs62113381 | C | T | -0.1054 | 0.0232 | 5.54E-06 |
| genus | Holdemania | rs1867876 | C | T | 0.0843 | 0.0162 | 2.74E-07 |
| genus | Holdemania | rs9500080 | T | C | 0.0927 | 0.0179 | 4.09E-07 |
| genus | Holdemania | rs6133067 | C | T | 0.0911 | 0.0179 | 5.17E-07 |
| genus | Holdemania | rs150096134 | A | T | 0.1621 | 0.0332 | 2.38E-06 |
| genus | Holdemania | rs41438744 | G | C | -0.1249 | 0.0270 | 2.44E-06 |
| genus | Holdemania | rs77293403 | G | A | 0.1646 | 0.0342 | 1.77E-06 |
| genus | Holdemania | rs80149660 | T | C | -0.2330 | 0.0519 | 6.04E-06 |
| genus | Holdemania | rs116500994 | T | G | -0.1376 | 0.0293 | 2.34E-06 |
| genus | Holdemania | rs55888180 | G | C | 0.1287 | 0.0283 | 5.89E-06 |
| genus | Holdemania | rs4146507 | T | C | 0.0795 | 0.0177 | 7.23E-06 |
| genus | Holdemania | rs111745969 | G | A | 0.1207 | 0.0266 | 3.71E-06 |
| genus | Holdemania | rs113593397 | G | A | -0.1289 | 0.0283 | 9.36E-06 |
| genus | Holdemania | rs10885477 | C | T | -0.1351 | 0.0302 | 8.60E-06 |
| genus | Holdemania | rs9529719 | C | T | 0.0740 | 0.0160 | 5.97E-06 |
| genus | Holdemania | rs967319 | C | T | 0.0789 | 0.0177 | 8.38E-06 |
| genus | Holdemania | rs12701617 | G | A | -0.0661 | 0.0149 | 9.52E-06 |
| genus | Holdemania | rs73139538 | A | G | -0.1486 | 0.0327 | 7.77E-06 |
| genus | Holdemania | rs11080063 | A | G | -0.0665 | 0.0150 | 6.67E-06 |
| genus | Howardella | rs901099 | G | T | -0.1272 | 0.0251 | 6.53E-07 |
| genus | Howardella | rs1484873 | G | A | -0.2278 | 0.0463 | 2.56E-06 |
| genus | Howardella | rs17167098 | A | G | -0.1694 | 0.0352 | 1.12E-06 |
| genus | Howardella | rs609430 | G | T | -0.1120 | 0.0239 | 3.34E-06 |
| genus | Howardella | rs36081916 | C | T | -0.1812 | 0.0403 | 4.70E-06 |
| genus | Howardella | rs12452946 | G | A | -0.1058 | 0.0229 | 3.80E-06 |
| genus | Howardella | rs61771805 | T | A | -0.1368 | 0.0297 | 4.03E-06 |
| genus | Howardella | rs672217 | A | G | 0.1641 | 0.0350 | 3.52E-06 |
| genus | Howardella | rs3791893 | G | A | 0.1470 | 0.0340 | 9.50E-06 |
| genus | Howardella | rs10048062 | T | C | -0.1474 | 0.0337 | 8.59E-06 |
| genus | Howardella | rs2154047 | A | C | -0.1926 | 0.0420 | 9.97E-06 |
| genus | Hungatella | rs13128780 | C | T | -0.1497 | 0.0313 | 1.75E-06 |
| genus | Hungatella | rs72759041 | T | G | -0.1260 | 0.0282 | 3.86E-06 |
| genus | Hungatella | rs10044993 | A | C | 0.1395 | 0.0317 | 8.07E-06 |
| genus | Hungatella | rs13249325 | G | T | -0.1000 | 0.0226 | 9.69E-06 |
| genus | Hungatella | rs17092615 | A | G | 0.1522 | 0.0338 | 7.38E-06 |
| genus | Intestinibacter | rs10805326 | A | G | 0.0775 | 0.0140 | 3.55E-08 |
| genus | Intestinibacter | rs4327025 | A | G | -0.0810 | 0.0154 | 1.64E-07 |
| genus | Intestinibacter | rs478972 | C | T | -0.1427 | 0.0297 | 1.82E-06 |
| genus | Intestinibacter | rs6875660 | T | C | 0.0890 | 0.0194 | 3.06E-06 |
| genus | Intestinibacter | rs16938435 | C | T | -0.1122 | 0.0235 | 1.80E-06 |
| genus | Intestinibacter | rs6062862 | G | A | 0.0925 | 0.0205 | 6.68E-06 |
| genus | Intestinibacter | rs118030283 | A | G | -0.1518 | 0.0324 | 2.67E-06 |
| genus | Intestinibacter | rs11109097 | T | C | 0.0624 | 0.0139 | 5.49E-06 |
| genus | Intestinibacter | rs9348442 | T | C | 0.0991 | 0.0222 | 6.26E-06 |
| genus | Intestinibacter | rs447950 | G | A | 0.0628 | 0.0137 | 5.64E-06 |
| genus | Intestinibacter | rs62430350 | C | T | 0.1511 | 0.0351 | 6.84E-06 |
| genus | Intestinibacter | rs2702387 | G | A | 0.0609 | 0.0132 | 4.26E-06 |
| genus | Intestinibacter | rs68093214 | T | C | 0.0662 | 0.0150 | 9.26E-06 |
| genus | Intestinibacter | rs2098844 | T | C | -0.0575 | 0.0128 | 6.79E-06 |
| genus | Intestinibacter | rs893394 | A | G | 0.0583 | 0.0131 | 7.85E-06 |
| genus | Intestinimonas | rs11258178 | G | A | 0.0661 | 0.0134 | 6.98E-07 |
| genus | Intestinimonas | rs12226153 | G | A | -0.1511 | 0.0307 | 5.12E-07 |
| genus | Intestinimonas | rs4784055 | C | T | -0.1753 | 0.0386 | 8.72E-07 |
| genus | Intestinimonas | rs716604 | G | A | 0.0818 | 0.0166 | 8.57E-07 |
| genus | Intestinimonas | rs2930225 | T | G | 0.0730 | 0.0153 | 1.35E-06 |
| genus | Intestinimonas | rs62240188 | A | G | 0.1301 | 0.0267 | 2.20E-06 |
| genus | Intestinimonas | rs2731794 | T | C | 0.1206 | 0.0258 | 1.92E-06 |
| genus | Intestinimonas | rs7170984 | C | T | -0.0658 | 0.0141 | 2.98E-06 |
| genus | Intestinimonas | rs10262702 | C | T | 0.0918 | 0.0195 | 2.06E-06 |
| genus | Intestinimonas | rs12566247 | A | T | 0.0637 | 0.0135 | 2.19E-06 |
| genus | Intestinimonas | rs1859797 | A | G | 0.0604 | 0.0132 | 4.12E-06 |
| genus | Intestinimonas | rs11928843 | A | G | -0.0607 | 0.0132 | 4.21E-06 |
| genus | Intestinimonas | rs17067892 | T | C | 0.1072 | 0.0250 | 6.38E-06 |
| genus | Intestinimonas | rs72982915 | T | C | 0.1832 | 0.0403 | 4.91E-06 |
| genus | Intestinimonas | rs994794 | C | G | -0.1419 | 0.0315 | 7.31E-06 |
| genus | Intestinimonas | rs4113676 | C | A | -0.2185 | 0.0490 | 7.42E-06 |
| genus | Intestinimonas | rs62427239 | A | C | 0.1627 | 0.0368 | 9.41E-06 |
| genus | Intestinimonas | rs2276760 | G | A | -0.0685 | 0.0153 | 7.84E-06 |
| genus | Intestinimonas | rs6934519 | T | C | 0.0692 | 0.0151 | 8.57E-06 |
| genus | Intestinimonas | rs1000888 | G | C | -0.0585 | 0.0132 | 9.71E-06 |
| genus | Lachnoclostridium | rs6112314 | C | A | -0.0562 | 0.0108 | 2.43E-07 |
| genus | Lachnoclostridium | rs62285313 | G | A | 0.0864 | 0.0182 | 1.58E-06 |
| genus | Lachnoclostridium | rs78068103 | G | A | 0.0886 | 0.0194 | 3.67E-06 |
| genus | Lachnoclostridium | rs615997 | C | T | 0.0512 | 0.0106 | 2.03E-06 |
| genus | Lachnoclostridium | rs61915992 | T | A | 0.0804 | 0.0172 | 2.67E-06 |
| genus | Lachnoclostridium | rs789029 | T | C | -0.0641 | 0.0138 | 3.75E-06 |
| genus | Lachnoclostridium | rs12566975 | C | T | -0.0468 | 0.0106 | 9.57E-06 |
| genus | Lachnoclostridium | rs4738679 | A | G | -0.0520 | 0.0114 | 4.42E-06 |
| genus | Lachnoclostridium | rs1031599 | T | G | -0.0786 | 0.0176 | 6.31E-06 |
| genus | Lachnoclostridium | rs72829893 | T | G | 0.1175 | 0.0268 | 5.58E-06 |
| genus | Lachnoclostridium | rs3821998 | A | C | -0.0864 | 0.0193 | 6.72E-06 |
| genus | Lachnoclostridium | rs2385421 | G | A | 0.0746 | 0.0181 | 7.14E-06 |
| genus | Lachnoclostridium | rs1528479 | A | G | -0.0498 | 0.0112 | 9.64E-06 |
| genus | Lachnoclostridium | rs1997204 | C | T | -0.1081 | 0.0242 | 5.97E-06 |
| genus | Lachnoclostridium | rs62028349 | C | G | 0.0470 | 0.0106 | 9.17E-06 |
| genus | Lachnospira | rs13157098 | G | A | -0.0768 | 0.0155 | 5.99E-07 |
| genus | Lachnospira | rs4923324 | A | G | -0.0617 | 0.0133 | 2.44E-06 |
| genus | Lachnospira | rs56791201 | C | T | 0.0518 | 0.0111 | 2.93E-06 |
| genus | Lachnospira | rs2326833 | G | C | -0.0785 | 0.0171 | 4.60E-06 |
| genus | Lachnospira | rs4686798 | C | T | 0.0532 | 0.0114 | 2.74E-06 |
| genus | Lachnospira | rs159484 | A | G | 0.0795 | 0.0177 | 6.68E-06 |
| genus | Lachnospira | rs2520509 | G | A | 0.0519 | 0.0116 | 7.42E-06 |
| genus | LachnospiraceaeFCS020group | rs7249113 | A | G | 0.0679 | 0.0133 | 3.72E-07 |
| genus | LachnospiraceaeFCS020group | rs12078956 | G | C | 0.1061 | 0.0223 | 2.15E-06 |
| genus | LachnospiraceaeFCS020group | rs369444 | G | C | 0.1255 | 0.0259 | 3.15E-06 |
| genus | LachnospiraceaeFCS020group | rs72793667 | G | A | -0.1169 | 0.0247 | 1.63E-06 |
| genus | LachnospiraceaeFCS020group | rs1363769 | C | T | -0.2006 | 0.0449 | 1.58E-06 |
| genus | LachnospiraceaeFCS020group | rs10093861 | A | G | -0.0569 | 0.0121 | 3.06E-06 |
| genus | LachnospiraceaeFCS020group | rs9788306 | T | C | -0.0628 | 0.0131 | 1.39E-06 |
| genus | LachnospiraceaeFCS020group | rs62140927 | T | C | 0.0614 | 0.0131 | 2.38E-06 |
| genus | LachnospiraceaeFCS020group | rs113859143 | C | G | -0.1089 | 0.0242 | 2.55E-06 |
| genus | LachnospiraceaeFCS020group | rs9919338 | C | G | -0.0553 | 0.0121 | 4.91E-06 |
| genus | LachnospiraceaeFCS020group | rs35035870 | C | T | -0.1906 | 0.0414 | 2.62E-06 |
| genus | LachnospiraceaeFCS020group | rs2862811 | C | T | 0.0565 | 0.0122 | 3.92E-06 |
| genus | LachnospiraceaeFCS020group | rs1254846 | A | G | 0.1060 | 0.0233 | 5.60E-06 |
| genus | LachnospiraceaeFCS020group | rs4452603 | G | T | 0.0604 | 0.0136 | 8.98E-06 |
| genus | LachnospiraceaeFCS020group | rs2322265 | T | C | -0.0666 | 0.0142 | 5.21E-06 |
| genus | LachnospiraceaeFCS020group | rs3999074 | T | G | -0.0551 | 0.0122 | 6.55E-06 |
| genus | LachnospiraceaeFCS020group | rs9308097 | G | A | 0.0554 | 0.0124 | 7.47E-06 |
| genus | LachnospiraceaeNC2004group | rs6116753 | A | G | 0.0995 | 0.0209 | 2.92E-06 |
| genus | LachnospiraceaeNC2004group | rs3756315 | G | A | -0.0883 | 0.0188 | 3.33E-06 |
| genus | LachnospiraceaeNC2004group | rs12127733 | A | G | 0.1152 | 0.0246 | 3.11E-06 |
| genus | LachnospiraceaeNC2004group | rs17067076 | A | G | -0.1546 | 0.0352 | 5.61E-06 |
| genus | LachnospiraceaeNC2004group | rs1331592 | G | C | 0.0949 | 0.0208 | 5.34E-06 |
| genus | LachnospiraceaeNC2004group | rs1928659 | C | T | 0.1025 | 0.0226 | 6.17E-06 |
| genus | LachnospiraceaeNC2004group | rs1929743 | C | T | 0.0837 | 0.0190 | 9.06E-06 |
| genus | LachnospiraceaeNC2004group | rs12863463 | A | G | -0.1564 | 0.0345 | 6.04E-06 |
| genus | LachnospiraceaeNC2004group | rs117467633 | C | T | -0.1697 | 0.0383 | 9.13E-06 |
| genus | LachnospiraceaeNC2004group | rs12208226 | A | C | -0.1547 | 0.0340 | 9.75E-06 |
| genus | LachnospiraceaeND3007group | rs9932954 | G | A | -0.0562 | 0.0116 | 1.25E-06 |
| genus | LachnospiraceaeND3007group | rs13110238 | G | C | -0.0637 | 0.0142 | 4.48E-06 |
| genus | LachnospiraceaeND3007group | rs72776675 | C | T | -0.0647 | 0.0148 | 8.72E-06 |
| genus | LachnospiraceaeND3007group | rs2861203 | A | G | 0.0572 | 0.0127 | 7.37E-06 |
| genus | LachnospiraceaeNK4A136group | rs12362320 | C | G | 0.0573 | 0.0115 | 8.04E-07 |
| genus | LachnospiraceaeNK4A136group | rs954878 | G | A | -0.0521 | 0.0109 | 1.78E-06 |
| genus | LachnospiraceaeNK4A136group | rs7832116 | G | A | -0.0715 | 0.0152 | 3.57E-06 |
| genus | LachnospiraceaeNK4A136group | rs7616165 | T | G | -0.2305 | 0.0483 | 2.77E-06 |
| genus | LachnospiraceaeNK4A136group | rs76193507 | G | A | -0.2297 | 0.0500 | 2.93E-06 |
| genus | LachnospiraceaeNK4A136group | rs11263806 | G | A | -0.0525 | 0.0117 | 5.07E-06 |
| genus | LachnospiraceaeNK4A136group | rs73044693 | G | A | -0.1076 | 0.0230 | 3.57E-06 |
| genus | LachnospiraceaeNK4A136group | rs160061 | G | A | 0.0514 | 0.0108 | 2.12E-06 |
| genus | LachnospiraceaeNK4A136group | rs7073658 | G | T | -0.0500 | 0.0110 | 5.27E-06 |
| genus | LachnospiraceaeNK4A136group | rs68104925 | C | T | -0.0549 | 0.0115 | 2.37E-06 |
| genus | LachnospiraceaeNK4A136group | rs12611395 | G | A | -0.0903 | 0.0200 | 5.83E-06 |
| genus | LachnospiraceaeNK4A136group | rs2880566 | C | T | 0.0600 | 0.0135 | 5.61E-06 |
| genus | LachnospiraceaeNK4A136group | rs28540839 | C | A | 0.0508 | 0.0111 | 9.34E-06 |
| genus | LachnospiraceaeNK4A136group | rs59805249 | C | T | 0.0936 | 0.0208 | 9.45E-06 |
| genus | LachnospiraceaeNK4A136group | rs10952110 | T | G | 0.0488 | 0.0110 | 9.08E-06 |
| genus | LachnospiraceaeNK4A136group | rs4955932 | C | T | -0.0492 | 0.0109 | 7.05E-06 |
| genus | LachnospiraceaeUCG001 | rs437876 | C | T | 0.0785 | 0.0145 | 7.17E-08 |
| genus | LachnospiraceaeUCG001 | rs985416 | T | C | 0.0970 | 0.0182 | 1.46E-07 |
| genus | LachnospiraceaeUCG001 | rs2050911 | A | G | 0.0751 | 0.0154 | 1.11E-06 |
| genus | LachnospiraceaeUCG001 | rs10815577 | G | C | -0.0684 | 0.0144 | 1.72E-06 |
| genus | LachnospiraceaeUCG001 | rs573933 | C | T | -0.1079 | 0.0232 | 3.11E-06 |
| genus | LachnospiraceaeUCG001 | rs78848836 | G | A | -0.1189 | 0.0260 | 3.38E-06 |
| genus | LachnospiraceaeUCG001 | rs9403580 | T | C | 0.1078 | 0.0230 | 3.47E-06 |
| genus | LachnospiraceaeUCG001 | rs7213933 | A | T | -0.0816 | 0.0184 | 9.02E-06 |
| genus | LachnospiraceaeUCG001 | rs8104225 | G | A | 0.0892 | 0.0198 | 8.04E-06 |
| genus | LachnospiraceaeUCG001 | rs74034332 | A | G | 0.1680 | 0.0383 | 3.33E-06 |
| genus | LachnospiraceaeUCG001 | rs62496417 | G | T | -0.0749 | 0.0166 | 5.88E-06 |
| genus | LachnospiraceaeUCG001 | rs2371284 | C | T | -0.0762 | 0.0170 | 7.56E-06 |
| genus | LachnospiraceaeUCG001 | rs12131224 | T | C | 0.1171 | 0.0259 | 7.40E-06 |
| genus | LachnospiraceaeUCG001 | rs4981345 | C | T | -0.0682 | 0.0150 | 6.09E-06 |
| genus | LachnospiraceaeUCG001 | rs79476906 | A | T | -0.0871 | 0.0197 | 8.27E-06 |
| genus | LachnospiraceaeUCG001 | rs7341608 | C | T | -0.0785 | 0.0178 | 9.48E-06 |
| genus | LachnospiraceaeUCG004 | rs12747809 | A | G | -0.0622 | 0.0126 | 8.65E-07 |
| genus | LachnospiraceaeUCG004 | rs2882478 | A | G | -0.0577 | 0.0118 | 1.21E-06 |
| genus | LachnospiraceaeUCG004 | rs12673420 | A | G | 0.0554 | 0.0118 | 2.98E-06 |
| genus | LachnospiraceaeUCG004 | rs2444793 | T | C | -0.0542 | 0.0118 | 4.77E-06 |
| genus | LachnospiraceaeUCG004 | rs2726805 | G | A | 0.0548 | 0.0121 | 6.30E-06 |
| genus | LachnospiraceaeUCG004 | rs11128180 | G | A | 0.0648 | 0.0140 | 4.52E-06 |
| genus | LachnospiraceaeUCG004 | rs12202257 | A | G | -0.0551 | 0.0124 | 7.03E-06 |
| genus | LachnospiraceaeUCG004 | rs12894272 | G | A | 0.0580 | 0.0125 | 4.34E-06 |
| genus | LachnospiraceaeUCG004 | rs233486 | G | A | -0.0799 | 0.0178 | 6.28E-06 |
| genus | LachnospiraceaeUCG004 | rs35182105 | G | A | -0.1097 | 0.0242 | 4.87E-06 |
| genus | LachnospiraceaeUCG004 | rs6656451 | T | C | -0.0544 | 0.0119 | 5.57E-06 |
| genus | LachnospiraceaeUCG004 | rs12072562 | C | T | 0.1331 | 0.0304 | 7.07E-06 |
| genus | LachnospiraceaeUCG004 | rs2706242 | C | G | -0.0901 | 0.0200 | 9.84E-06 |
| genus | LachnospiraceaeUCG004 | rs62256516 | A | G | 0.0592 | 0.0132 | 9.85E-06 |
| genus | LachnospiraceaeUCG004 | rs7629954 | G | A | 0.1084 | 0.0238 | 5.77E-06 |
| genus | LachnospiraceaeUCG008 | rs10793103 | T | C | 0.0974 | 0.0181 | 9.35E-08 |
| genus | LachnospiraceaeUCG008 | rs9873555 | C | G | -0.1212 | 0.0233 | 2.41E-07 |
| genus | LachnospiraceaeUCG008 | rs13024781 | C | T | -0.0799 | 0.0169 | 2.29E-06 |
| genus | LachnospiraceaeUCG008 | rs10741777 | C | T | -0.0974 | 0.0195 | 7.69E-07 |
| genus | LachnospiraceaeUCG008 | rs955844 | C | A | 0.1121 | 0.0228 | 1.81E-06 |
| genus | LachnospiraceaeUCG008 | rs67078837 | C | T | -0.0846 | 0.0171 | 7.68E-07 |
| genus | LachnospiraceaeUCG008 | rs62277846 | T | C | 0.1023 | 0.0212 | 1.59E-06 |
| genus | LachnospiraceaeUCG008 | rs10801803 | A | G | -0.1170 | 0.0243 | 1.40E-06 |
| genus | LachnospiraceaeUCG008 | rs61944774 | G | A | 0.1798 | 0.0394 | 6.34E-06 |
| genus | LachnospiraceaeUCG008 | rs75356640 | A | G | 0.1365 | 0.0303 | 9.83E-06 |
| genus | LachnospiraceaeUCG008 | rs57254474 | A | G | 0.0887 | 0.0199 | 6.92E-06 |
| genus | LachnospiraceaeUCG008 | rs57091572 | G | A | -0.1104 | 0.0236 | 2.86E-06 |
| genus | LachnospiraceaeUCG010 | rs11192447 | G | A | 0.1266 | 0.0243 | 4.69E-07 |
| genus | LachnospiraceaeUCG010 | rs9981767 | C | A | 0.0655 | 0.0132 | 9.96E-07 |
| genus | LachnospiraceaeUCG010 | rs74315802 | T | G | 0.0867 | 0.0183 | 3.19E-06 |
| genus | LachnospiraceaeUCG010 | rs12346653 | T | C | 0.0658 | 0.0140 | 2.70E-06 |
| genus | LachnospiraceaeUCG010 | rs10414815 | C | T | 0.1045 | 0.0230 | 4.24E-06 |
| genus | LachnospiraceaeUCG010 | rs72761829 | T | A | 0.1119 | 0.0239 | 2.58E-06 |
| genus | LachnospiraceaeUCG010 | rs72894957 | A | G | 0.2223 | 0.0486 | 5.68E-06 |
| genus | LachnospiraceaeUCG010 | rs336138 | T | G | 0.0780 | 0.0172 | 7.48E-06 |
| genus | LachnospiraceaeUCG010 | rs4576377 | C | A | -0.0572 | 0.0127 | 7.63E-06 |
| genus | LachnospiraceaeUCG010 | rs2833528 | T | C | -0.0562 | 0.0128 | 9.92E-06 |
| genus | LachnospiraceaeUCG010 | rs2153460 | T | A | -0.0684 | 0.0157 | 9.17E-06 |
| genus | LachnospiraceaeUCG010 | rs17730011 | A | G | -0.0702 | 0.0157 | 7.85E-06 |
| genus | Lactobacillus | rs921925 | C | A | 0.0985 | 0.0203 | 9.72E-07 |
| genus | Lactobacillus | rs16861661 | A | G | -0.1831 | 0.0381 | 1.28E-06 |
| genus | Lactobacillus | rs768253 | G | T | -0.0792 | 0.0172 | 4.25E-06 |
| genus | Lactobacillus | rs11674854 | T | C | -0.0853 | 0.0177 | 1.59E-06 |
| genus | Lactobacillus | rs328312 | A | T | 0.0815 | 0.0169 | 1.41E-06 |
| genus | Lactobacillus | rs6092149 | T | A | -0.0801 | 0.0171 | 3.29E-06 |
| genus | Lactobacillus | rs75127669 | A | C | 0.1398 | 0.0310 | 6.83E-06 |
| genus | Lactobacillus | rs77478751 | G | A | -0.2199 | 0.0476 | 7.33E-06 |
| genus | Lactobacillus | rs1530559 | A | G | 0.0804 | 0.0178 | 4.93E-06 |
| genus | Lactobacillus | rs62314653 | A | C | 0.1877 | 0.0395 | 2.24E-06 |
| genus | Lactobacillus | rs7399658 | A | G | -0.1071 | 0.0222 | 3.12E-06 |
| genus | Lactobacillus | rs12693845 | T | C | -0.0805 | 0.0177 | 8.96E-06 |
| genus | Lactococcus | rs34757988 | C | G | 0.1223 | 0.0229 | 8.95E-08 |
| genus | Lactococcus | rs757872 | C | G | 0.1408 | 0.0276 | 4.37E-07 |
| genus | Lactococcus | rs123059 | C | T | -0.1367 | 0.0275 | 1.27E-06 |
| genus | Lactococcus | rs4766997 | T | C | 0.1146 | 0.0238 | 2.06E-06 |
| genus | Lactococcus | rs10417872 | G | T | 0.1183 | 0.0245 | 1.29E-06 |
| genus | Lactococcus | rs6674304 | T | C | 0.2008 | 0.0442 | 6.18E-06 |
| genus | Lactococcus | rs7992246 | C | T | 0.1042 | 0.0231 | 4.45E-06 |
| genus | Lactococcus | rs55910161 | T | C | 0.1464 | 0.0307 | 2.36E-06 |
| genus | Lactococcus | rs12621813 | A | G | 0.1084 | 0.0240 | 6.61E-06 |
| genus | Lactococcus | rs2293361 | T | C | -0.1992 | 0.0431 | 1.40E-06 |
| genus | Lactococcus | rs17168302 | A | G | 0.1919 | 0.0425 | 6.29E-06 |
| genus | Marvinbryantia | rs61884471 | A | G | 0.1244 | 0.0248 | 1.01E-06 |
| genus | Marvinbryantia | rs2724813 | G | A | -0.0841 | 0.0168 | 6.28E-07 |
| genus | Marvinbryantia | rs1187983 | T | C | -0.0935 | 0.0193 | 2.02E-06 |
| genus | Marvinbryantia | rs2842896 | T | C | -0.0649 | 0.0131 | 7.25E-07 |
| genus | Marvinbryantia | rs11645029 | C | G | -0.0606 | 0.0132 | 4.15E-06 |
| genus | Marvinbryantia | rs12963345 | C | G | -0.0597 | 0.0132 | 6.60E-06 |
| genus | Marvinbryantia | rs2863363 | G | A | 0.0635 | 0.0136 | 3.11E-06 |
| genus | Marvinbryantia | rs72948274 | C | A | -0.1264 | 0.0272 | 3.26E-06 |
| genus | Marvinbryantia | rs146541147 | A | G | 0.1188 | 0.0268 | 6.86E-06 |
| genus | Marvinbryantia | rs11620597 | C | T | 0.1195 | 0.0272 | 7.80E-06 |
| genus | Marvinbryantia | rs8006832 | T | G | -0.0952 | 0.0217 | 6.58E-06 |
| genus | Marvinbryantia | rs3125832 | C | A | 0.0679 | 0.0150 | 5.03E-06 |
| genus | Methanobrevibacter | rs76029318 | C | T | 0.2228 | 0.0454 | 1.08E-06 |
| genus | Methanobrevibacter | rs10202904 | G | T | -0.1128 | 0.0239 | 3.09E-06 |
| genus | Methanobrevibacter | rs894996 | A | C | 0.2142 | 0.0456 | 3.82E-06 |
| genus | Methanobrevibacter | rs11018665 | T | A | 0.1130 | 0.0254 | 7.03E-06 |
| genus | Methanobrevibacter | rs1334944 | C | T | 0.1152 | 0.0255 | 7.61E-06 |
| genus | Methanobrevibacter | rs6776814 | C | T | -0.1890 | 0.0420 | 8.05E-06 |
| genus | Methanobrevibacter | rs4779844 | C | G | 0.1096 | 0.0248 | 9.28E-06 |
| genus | Methanobrevibacter | rs4802933 | G | A | -0.1356 | 0.0308 | 9.74E-06 |
| genus | Odoribacter | rs503751 | G | C | 0.0618 | 0.0119 | 2.08E-07 |
| genus | Odoribacter | rs77779484 | A | G | -0.1335 | 0.0269 | 6.56E-07 |
| genus | Odoribacter | rs10423795 | T | C | 0.0551 | 0.0121 | 6.58E-06 |
| genus | Odoribacter | rs6856150 | A | G | 0.0882 | 0.0194 | 6.06E-06 |
| genus | Odoribacter | rs10093869 | G | A | -0.0578 | 0.0125 | 3.67E-06 |
| genus | Odoribacter | rs4793970 | G | A | -0.0576 | 0.0129 | 6.03E-06 |
| genus | Odoribacter | rs74553962 | G | T | 0.1214 | 0.0264 | 9.49E-06 |
| genus | Odoribacter | rs28417404 | G | A | -0.0727 | 0.0161 | 3.68E-06 |
| genus | Odoribacter | rs16918425 | T | A | 0.1000 | 0.0224 | 8.85E-06 |
| genus | Olsenella | rs1035588 | G | A | -0.1081 | 0.0237 | 4.86E-06 |
| genus | Olsenella | rs62112538 | T | C | -0.1994 | 0.0407 | 1.19E-06 |
| genus | Olsenella | rs35225860 | G | A | -0.2236 | 0.0482 | 3.87E-06 |
| genus | Olsenella | rs72691585 | A | C | -0.2491 | 0.0521 | 2.95E-06 |
| genus | Olsenella | rs9460691 | A | C | 0.1200 | 0.0269 | 7.28E-06 |
| genus | Olsenella | rs17148768 | A | G | 0.1404 | 0.0296 | 2.20E-06 |
| genus | Olsenella | rs61090148 | G | A | -0.1048 | 0.0231 | 6.44E-06 |
| genus | Olsenella | rs7540303 | T | C | 0.1080 | 0.0236 | 5.32E-06 |
| genus | Olsenella | rs2759329 | A | G | -0.1111 | 0.0237 | 3.43E-06 |
| genus | Olsenella | rs6046522 | T | C | 0.1229 | 0.0270 | 4.48E-06 |
| genus | Olsenella | rs8066522 | A | G | -0.1065 | 0.0240 | 9.70E-06 |
| genus | Oscillibacter | rs234108 | G | A | 0.0750 | 0.0153 | 9.16E-07 |
| genus | Oscillibacter | rs36095275 | T | C | -0.0752 | 0.0157 | 1.40E-06 |
| genus | Oscillibacter | rs11627628 | C | T | 0.1440 | 0.0290 | 1.01E-06 |
| genus | Oscillibacter | rs9393920 | G | A | -0.0745 | 0.0151 | 9.92E-07 |
| genus | Oscillibacter | rs133832 | C | A | -0.0796 | 0.0162 | 1.15E-06 |
| genus | Oscillibacter | rs12649930 | G | T | 0.1216 | 0.0260 | 4.09E-06 |
| genus | Oscillibacter | rs75453768 | T | G | 0.1221 | 0.0269 | 5.35E-06 |
| genus | Oscillibacter | rs16866406 | G | A | 0.0989 | 0.0209 | 3.08E-06 |
| genus | Oscillibacter | rs761240 | G | T | -0.1766 | 0.0389 | 2.04E-06 |
| genus | Oscillibacter | rs4506202 | G | A | -0.0711 | 0.0152 | 3.21E-06 |
| genus | Oscillibacter | rs61883564 | G | A | -0.1014 | 0.0221 | 3.39E-06 |
| genus | Oscillibacter | rs16934185 | G | A | -0.1296 | 0.0282 | 4.38E-06 |
| genus | Oscillibacter | rs12417956 | G | C | 0.0785 | 0.0174 | 6.03E-06 |
| genus | Oscillibacter | rs6901560 | G | C | 0.0855 | 0.0187 | 6.21E-06 |
| genus | Oscillibacter | rs62206502 | A | C | -0.0681 | 0.0151 | 6.60E-06 |
| genus | Oscillibacter | rs137917150 | A | T | -0.1750 | 0.0388 | 4.62E-06 |
| genus | Oscillibacter | rs11990279 | C | T | -0.0825 | 0.0180 | 4.94E-06 |
| genus | Oscillospira | rs12206468 | A | G | -0.1330 | 0.0270 | 1.04E-06 |
| genus | Oscillospira | rs73038677 | A | T | -0.0832 | 0.0169 | 1.09E-06 |
| genus | Oscillospira | rs1954532 | C | T | -0.0826 | 0.0175 | 2.27E-06 |
| genus | Oscillospira | rs28889936 | C | A | 0.1141 | 0.0253 | 3.37E-06 |
| genus | Oscillospira | rs751183 | C | T | -0.0774 | 0.0172 | 6.85E-06 |
| genus | Oscillospira | rs8076323 | G | A | 0.0715 | 0.0157 | 5.61E-06 |
| genus | Oscillospira | rs72866977 | C | A | -0.1306 | 0.0282 | 5.63E-06 |
| genus | Oscillospira | rs62422654 | T | C | 0.0898 | 0.0198 | 6.47E-06 |
| genus | Oscillospira | rs12925026 | C | T | 0.1356 | 0.0307 | 9.31E-06 |
| genus | Oxalobacter | rs4428215 | A | G | 0.1303 | 0.0242 | 7.51E-08 |
| genus | Oxalobacter | rs736744 | T | C | 0.1179 | 0.0211 | 2.57E-08 |
| genus | Oxalobacter | rs6000536 | T | C | -0.1310 | 0.0254 | 2.06E-07 |
| genus | Oxalobacter | rs36057338 | T | G | 0.2078 | 0.0421 | 8.80E-07 |
| genus | Oxalobacter | rs6071435 | A | T | -0.1055 | 0.0215 | 1.07E-06 |
| genus | Oxalobacter | rs12002250 | C | A | 0.2171 | 0.0466 | 1.42E-06 |
| genus | Oxalobacter | rs1569853 | C | T | -0.1381 | 0.0297 | 3.65E-06 |
| genus | Oxalobacter | rs11108500 | G | A | -0.1991 | 0.0427 | 3.74E-06 |
| genus | Oxalobacter | rs10464997 | A | G | 0.1377 | 0.0295 | 3.30E-06 |
| genus | Oxalobacter | rs111966731 | C | T | 0.2131 | 0.0472 | 7.30E-06 |
| genus | Oxalobacter | rs6993398 | A | G | 0.1272 | 0.0279 | 7.13E-06 |
| genus | Oxalobacter | rs3862635 | T | C | -0.1721 | 0.0394 | 9.19E-06 |
| genus | Parabacteroides | rs60884758 | T | C | -0.0703 | 0.0142 | 5.71E-07 |
| genus | Parabacteroides | rs3860755 | C | G | 0.0559 | 0.0117 | 1.71E-06 |
| genus | Parabacteroides | rs4236095 | A | G | 0.0762 | 0.0157 | 1.93E-06 |
| genus | Parabacteroides | rs115602804 | A | G | 0.1031 | 0.0223 | 1.93E-06 |
| genus | Parabacteroides | rs114567323 | C | T | 0.1865 | 0.0405 | 5.65E-06 |
| genus | Parabacteroides | rs7298818 | T | C | 0.0889 | 0.0201 | 8.54E-06 |
| genus | Parabacteroides | rs6657302 | C | T | -0.1045 | 0.0226 | 9.76E-06 |
| genus | Parabacteroides | rs11965579 | C | G | 0.1628 | 0.0382 | 8.87E-06 |
| genus | Parabacteroides | rs17141986 | C | G | 0.0500 | 0.0111 | 7.27E-06 |
| genus | Parabacteroides | rs72893646 | T | A | -0.0716 | 0.0157 | 8.83E-06 |
| genus | Paraprevotella | rs2081023 | G | A | -0.1226 | 0.0237 | 2.64E-07 |
| genus | Paraprevotella | rs9900242 | G | A | -0.0853 | 0.0175 | 1.14E-06 |
| genus | Paraprevotella | rs9602779 | C | A | -0.1067 | 0.0220 | 6.93E-07 |
| genus | Paraprevotella | rs140997932 | C | T | -0.1624 | 0.0354 | 2.11E-06 |
| genus | Paraprevotella | rs145020347 | G | A | -0.1247 | 0.0262 | 4.03E-06 |
| genus | Paraprevotella | rs4767113 | T | C | 0.0882 | 0.0184 | 2.14E-06 |
| genus | Paraprevotella | rs3008582 | C | T | 0.1057 | 0.0227 | 4.36E-06 |
| genus | Paraprevotella | rs4756632 | T | G | -0.1389 | 0.0290 | 3.82E-06 |
| genus | Paraprevotella | rs10842464 | C | T | -0.0758 | 0.0173 | 6.60E-06 |
| genus | Paraprevotella | rs3801748 | A | G | 0.0780 | 0.0172 | 5.20E-06 |
| genus | Paraprevotella | rs7240324 | G | T | -0.1023 | 0.0227 | 5.96E-06 |
| genus | Paraprevotella | rs17109926 | G | A | -0.0988 | 0.0216 | 6.75E-06 |
| genus | Paraprevotella | rs17785622 | G | A | 0.2481 | 0.0524 | 1.93E-06 |
| genus | Parasutterella | rs2387977 | C | T | -0.0682 | 0.0135 | 5.38E-07 |
| genus | Parasutterella | rs7572229 | A | G | 0.0663 | 0.0133 | 6.32E-07 |
| genus | Parasutterella | rs35414597 | A | T | -0.0685 | 0.0142 | 1.51E-06 |
| genus | Parasutterella | rs6828768 | T | C | 0.0637 | 0.0133 | 1.78E-06 |
| genus | Parasutterella | rs10899911 | G | A | -0.0717 | 0.0148 | 1.15E-06 |
| genus | Parasutterella | rs7303158 | T | C | 0.0647 | 0.0134 | 1.33E-06 |
| genus | Parasutterella | rs78383039 | C | T | -0.1463 | 0.0297 | 1.57E-06 |
| genus | Parasutterella | rs35055552 | C | T | 0.1096 | 0.0235 | 3.35E-06 |
| genus | Parasutterella | rs2090816 | C | A | 0.0841 | 0.0177 | 2.90E-06 |
| genus | Parasutterella | rs1403396 | T | A | -0.0757 | 0.0159 | 2.76E-06 |
| genus | Parasutterella | rs55877868 | C | A | -0.1045 | 0.0228 | 2.87E-06 |
| genus | Parasutterella | rs8039785 | G | T | 0.0618 | 0.0133 | 3.62E-06 |
| genus | Parasutterella | rs823424 | A | G | -0.0713 | 0.0157 | 4.95E-06 |
| genus | Parasutterella | rs7311004 | C | T | -0.0618 | 0.0136 | 5.92E-06 |
| genus | Parasutterella | rs62273907 | G | A | 0.2295 | 0.0502 | 5.88E-06 |
| genus | Parasutterella | rs6809952 | A | G | -0.0685 | 0.0151 | 8.13E-06 |
| genus | Parasutterella | rs11715853 | A | G | -0.0663 | 0.0146 | 6.23E-06 |
| genus | Peptococcus | rs75754569 | G | C | 0.1814 | 0.0319 | 1.10E-08 |
| genus | Peptococcus | rs77681628 | T | C | 0.2003 | 0.0387 | 2.69E-07 |
| genus | Peptococcus | rs10031059 | C | T | -0.1212 | 0.0226 | 1.24E-07 |
| genus | Peptococcus | rs6918730 | A | G | 0.1353 | 0.0290 | 1.15E-06 |
| genus | Peptococcus | rs413827 | A | G | 0.1102 | 0.0238 | 3.30E-06 |
| genus | Peptococcus | rs2054133 | A | G | 0.0895 | 0.0188 | 2.14E-06 |
| genus | Peptococcus | rs11001941 | A | G | -0.1956 | 0.0392 | 1.33E-06 |
| genus | Peptococcus | rs7033353 | G | T | 0.0902 | 0.0190 | 2.22E-06 |
| genus | Peptococcus | rs5770862 | C | T | 0.1620 | 0.0357 | 3.22E-06 |
| genus | Peptococcus | rs34282744 | C | G | 0.1918 | 0.0400 | 1.84E-06 |
| genus | Peptococcus | rs72850165 | C | T | -0.1343 | 0.0300 | 5.74E-06 |
| genus | Peptococcus | rs77614201 | C | T | -0.1759 | 0.0388 | 5.68E-06 |
| genus | Peptococcus | rs11030569 | T | A | -0.1740 | 0.0374 | 3.13E-06 |
| genus | Peptococcus | rs7766680 | C | G | 0.0977 | 0.0214 | 3.51E-06 |
| genus | Peptococcus | rs74592222 | A | G | 0.1380 | 0.0303 | 8.55E-06 |
| genus | Peptococcus | rs36121075 | G | A | -0.1407 | 0.0306 | 6.99E-06 |
| genus | Peptococcus | rs12069354 | T | C | 0.1676 | 0.0380 | 9.28E-06 |
| genus | Phascolarctobacterium | rs75882962 | C | T | 0.0969 | 0.0191 | 3.19E-07 |
| genus | Phascolarctobacterium | rs56157888 | C | A | 0.0955 | 0.0194 | 1.09E-06 |
| genus | Phascolarctobacterium | rs56069061 | A | G | -0.1113 | 0.0231 | 1.87E-06 |
| genus | Phascolarctobacterium | rs74540770 | A | G | -0.1210 | 0.0259 | 3.60E-06 |
| genus | Phascolarctobacterium | rs76124218 | G | C | -0.1594 | 0.0345 | 2.67E-06 |
| genus | Phascolarctobacterium | rs6427992 | C | G | -0.0652 | 0.0138 | 2.09E-06 |
| genus | Phascolarctobacterium | rs12618201 | G | A | 0.0642 | 0.0138 | 3.38E-06 |
| genus | Phascolarctobacterium | rs74847270 | G | A | -0.1049 | 0.0231 | 5.73E-06 |
| genus | Phascolarctobacterium | rs1264476 | G | T | 0.0767 | 0.0166 | 4.30E-06 |
| genus | Phascolarctobacterium | rs28525131 | A | G | -0.1187 | 0.0269 | 8.23E-06 |
| genus | Phascolarctobacterium | rs7982713 | A | G | 0.0727 | 0.0163 | 9.72E-06 |
| genus | Phascolarctobacterium | rs11929846 | C | T | -0.0697 | 0.0158 | 8.88E-06 |
| genus | Phascolarctobacterium | rs130483 | G | A | 0.0655 | 0.0144 | 6.79E-06 |
| genus | Prevotella7 | rs430270 | C | A | 0.1392 | 0.0297 | 2.87E-06 |
| genus | Prevotella7 | rs57404562 | A | C | 0.1555 | 0.0316 | 6.22E-07 |
| genus | Prevotella7 | rs9959718 | A | G | 0.1330 | 0.0275 | 1.90E-06 |
| genus | Prevotella7 | rs9608249 | G | A | -0.1582 | 0.0336 | 2.07E-06 |
| genus | Prevotella7 | rs2240542 | T | C | 0.1208 | 0.0262 | 4.84E-06 |
| genus | Prevotella7 | rs2918132 | T | C | -0.1146 | 0.0255 | 6.42E-06 |
| genus | Prevotella7 | rs16937247 | C | G | 0.1461 | 0.0352 | 9.64E-06 |
| genus | Prevotella7 | rs118038478 | G | A | 0.2057 | 0.0469 | 7.85E-06 |
| genus | Prevotella7 | rs12124567 | G | A | -0.1213 | 0.0275 | 9.49E-06 |
| genus | Prevotella7 | rs12195431 | C | T | 0.1965 | 0.0442 | 8.73E-06 |
| genus | Prevotella7 | rs9426434 | C | T | -0.1237 | 0.0279 | 9.72E-06 |
| genus | Prevotella7 | rs79263163 | C | A | -0.1440 | 0.0315 | 7.51E-06 |
| genus | Prevotella9 | rs111509883 | C | T | 0.1711 | 0.0348 | 1.24E-06 |
| genus | Prevotella9 | rs2683313 | G | A | -0.0725 | 0.0152 | 1.69E-06 |
| genus | Prevotella9 | rs117271932 | G | A | 0.2081 | 0.0440 | 2.82E-06 |
| genus | Prevotella9 | rs10512344 | G | C | 0.2474 | 0.0544 | 3.19E-06 |
| genus | Prevotella9 | rs9428102 | G | A | -0.0779 | 0.0176 | 4.62E-06 |
| genus | Prevotella9 | rs746764 | C | T | -0.0916 | 0.0193 | 2.04E-06 |
| genus | Prevotella9 | rs16966465 | C | G | 0.0743 | 0.0165 | 9.33E-06 |
| genus | Prevotella9 | rs2104588 | C | T | 0.1056 | 0.0238 | 8.13E-06 |
| genus | Prevotella9 | rs11199734 | T | A | 0.0770 | 0.0169 | 7.00E-06 |
| genus | Prevotella9 | rs7232121 | C | G | 0.0671 | 0.0144 | 3.76E-06 |
| genus | Prevotella9 | rs11685699 | T | C | -0.1414 | 0.0296 | 2.03E-06 |
| genus | Prevotella9 | rs9613013 | A | G | 0.0918 | 0.0203 | 6.10E-06 |
| genus | Prevotella9 | rs2495052 | G | A | 0.0839 | 0.0189 | 8.97E-06 |
| genus | Prevotella9 | rs72815774 | C | T | -0.1762 | 0.0393 | 8.78E-06 |
| genus | Prevotella9 | rs4968431 | T | G | 0.0640 | 0.0144 | 8.58E-06 |
| genus | Prevotella9 | rs4821647 | C | G | 0.0637 | 0.0144 | 9.95E-06 |
| genus | Prevotella9 | rs7976209 | C | T | -0.0871 | 0.0198 | 7.28E-06 |
| genus | Prevotella9 | rs12648235 | C | T | 0.0786 | 0.0178 | 7.39E-06 |
| genus | Prevotella9 | rs1304512 | A | G | 0.0762 | 0.0166 | 5.29E-06 |
| genus | Prevotella9 | rs7237249 | T | C | -0.0824 | 0.0182 | 8.93E-06 |
| genus | RikenellaceaeRC9gutgroup | rs2900503 | T | G | -0.1723 | 0.0327 | 1.55E-07 |
| genus | RikenellaceaeRC9gutgroup | rs17582787 | G | A | -0.1577 | 0.0340 | 3.55E-06 |
| genus | RikenellaceaeRC9gutgroup | rs7113155 | C | G | 0.1144 | 0.0248 | 5.26E-06 |
| genus | RikenellaceaeRC9gutgroup | rs4270579 | A | G | -0.1180 | 0.0271 | 5.46E-06 |
| genus | RikenellaceaeRC9gutgroup | rs2998141 | C | T | -0.1363 | 0.0293 | 4.42E-06 |
| genus | RikenellaceaeRC9gutgroup | rs4717843 | T | G | -0.1194 | 0.0261 | 4.72E-06 |
| genus | RikenellaceaeRC9gutgroup | rs9887954 | A | G | -0.1148 | 0.0249 | 4.81E-06 |
| genus | RikenellaceaeRC9gutgroup | rs7193937 | C | G | 0.1242 | 0.0277 | 6.19E-06 |
| genus | RikenellaceaeRC9gutgroup | rs80309088 | A | G | 0.1740 | 0.0383 | 4.56E-06 |
| genus | RikenellaceaeRC9gutgroup | rs12501673 | G | A | 0.1164 | 0.0262 | 6.29E-06 |
| genus | RikenellaceaeRC9gutgroup | rs17032291 | C | T | -0.1696 | 0.0368 | 6.61E-06 |
| genus | RikenellaceaeRC9gutgroup | rs2074881 | C | T | -0.1422 | 0.0324 | 9.45E-06 |
| genus | RikenellaceaeRC9gutgroup | rs7712231 | G | A | 0.1563 | 0.0350 | 7.97E-06 |
| genus | Romboutsia | rs61841503 | A | G | 0.0929 | 0.0171 | 4.00E-08 |
| genus | Romboutsia | rs10279978 | G | A | -0.0622 | 0.0128 | 1.17E-06 |
| genus | Romboutsia | rs16843578 | T | C | -0.0875 | 0.0197 | 5.08E-06 |
| genus | Romboutsia | rs77702691 | G | A | -0.0944 | 0.0209 | 7.37E-06 |
| genus | Romboutsia | rs75987356 | A | G | -0.1296 | 0.0280 | 6.71E-06 |
| genus | Romboutsia | rs75200530 | G | T | -0.1906 | 0.0421 | 5.07E-06 |
| genus | Romboutsia | rs9567264 | T | C | 0.0580 | 0.0127 | 5.76E-06 |
| genus | Romboutsia | rs34302036 | G | A | 0.0550 | 0.0121 | 5.88E-06 |
| genus | Romboutsia | rs7109293 | G | A | 0.0919 | 0.0206 | 6.98E-06 |
| genus | Romboutsia | rs10091895 | C | T | -0.0650 | 0.0146 | 6.62E-06 |
| genus | Romboutsia | rs114398731 | C | G | -0.1310 | 0.0294 | 7.96E-06 |
| genus | Romboutsia | rs28603357 | C | T | -0.2149 | 0.0475 | 8.52E-06 |
| genus | Romboutsia | rs11221428 | C | T | -0.0727 | 0.0158 | 6.49E-06 |
| genus | Romboutsia | rs9389266 | G | T | 0.0723 | 0.0162 | 9.38E-06 |
| genus | Romboutsia | rs62504452 | G | A | -0.0710 | 0.0157 | 4.66E-06 |
| genus | Roseburia | rs2034589 | C | G | 0.0630 | 0.0123 | 5.01E-07 |
| genus | Roseburia | rs116270582 | A | T | -0.1538 | 0.0329 | 1.20E-06 |
| genus | Roseburia | rs2160994 | C | T | 0.0551 | 0.0112 | 9.70E-07 |
| genus | Roseburia | rs16910295 | C | T | -0.0980 | 0.0210 | 2.91E-06 |
| genus | Roseburia | rs12740451 | C | T | 0.0698 | 0.0154 | 7.34E-06 |
| genus | Roseburia | rs6445851 | A | G | -0.0497 | 0.0108 | 3.53E-06 |
| genus | Roseburia | rs9300744 | T | C | -0.0588 | 0.0126 | 4.75E-06 |
| genus | Roseburia | rs2943022 | C | T | 0.0494 | 0.0107 | 4.11E-06 |
| genus | Roseburia | rs6930661 | T | C | -0.0962 | 0.0205 | 2.48E-06 |
| genus | Roseburia | rs4748237 | C | G | 0.0488 | 0.0106 | 4.67E-06 |
| genus | Roseburia | rs147990086 | G | A | -0.0579 | 0.0132 | 8.93E-06 |
| genus | Roseburia | rs329182 | C | T | 0.0690 | 0.0153 | 5.90E-06 |
| genus | Roseburia | rs302266 | C | T | -0.0777 | 0.0173 | 8.13E-06 |
| genus | Roseburia | rs75326254 | T | C | -0.1046 | 0.0231 | 7.50E-06 |
| genus | Roseburia | rs55858165 | C | A | 0.1793 | 0.0405 | 9.99E-06 |
| genus | Roseburia | rs57466170 | T | C | 0.0741 | 0.0172 | 8.30E-06 |
| genus | Roseburia | rs28040 | C | G | 0.0566 | 0.0127 | 9.26E-06 |
| genus | Roseburia | rs78753150 | C | A | 0.0969 | 0.0214 | 9.98E-06 |
| genus | Ruminiclostridium5 | rs243585 | G | C | -0.0586 | 0.0121 | 1.33E-06 |
| genus | Ruminiclostridium5 | rs2286384 | C | G | -0.0519 | 0.0107 | 1.44E-06 |
| genus | Ruminiclostridium5 | rs79968837 | G | A | -0.0950 | 0.0194 | 1.15E-06 |
| genus | Ruminiclostridium5 | rs2482038 | A | C | 0.0519 | 0.0109 | 1.70E-06 |
| genus | Ruminiclostridium5 | rs113753996 | C | T | 0.0821 | 0.0174 | 3.99E-06 |
| genus | Ruminiclostridium5 | rs1492620 | C | T | -0.0831 | 0.0180 | 3.53E-06 |
| genus | Ruminiclostridium5 | rs2791343 | C | T | 0.0517 | 0.0113 | 5.54E-06 |
| genus | Ruminiclostridium5 | rs10827477 | G | A | -0.0547 | 0.0115 | 2.19E-06 |
| genus | Ruminiclostridium5 | rs6121460 | A | G | 0.0933 | 0.0199 | 2.64E-06 |
| genus | Ruminiclostridium5 | rs2801960 | G | C | 0.0521 | 0.0115 | 6.21E-06 |
| genus | Ruminiclostridium5 | rs8053158 | G | A | -0.0741 | 0.0159 | 5.90E-06 |
| genus | Ruminiclostridium5 | rs1223978 | C | T | 0.0484 | 0.0108 | 8.16E-06 |
| genus | Ruminiclostridium5 | rs73002572 | C | G | 0.1815 | 0.0414 | 8.82E-06 |
| genus | Ruminiclostridium5 | rs2833828 | A | G | 0.0490 | 0.0109 | 6.82E-06 |
| genus | Ruminiclostridium5 | rs4955951 | G | A | -0.0714 | 0.0166 | 9.96E-06 |
| genus | Ruminiclostridium6 | rs1756364 | T | G | 0.0999 | 0.0197 | 2.54E-07 |
| genus | Ruminiclostridium6 | rs71414120 | G | T | 0.2009 | 0.0406 | 1.08E-06 |
| genus | Ruminiclostridium6 | rs61060922 | G | T | 0.1591 | 0.0322 | 1.09E-06 |
| genus | Ruminiclostridium6 | rs79968172 | A | G | 0.1161 | 0.0243 | 1.66E-06 |
| genus | Ruminiclostridium6 | rs77193512 | G | A | 0.0737 | 0.0153 | 1.30E-06 |
| genus | Ruminiclostridium6 | rs11992182 | C | A | 0.0625 | 0.0138 | 4.65E-06 |
| genus | Ruminiclostridium6 | rs72991535 | G | T | 0.1356 | 0.0295 | 4.95E-06 |
| genus | Ruminiclostridium6 | rs663262 | C | T | -0.1350 | 0.0311 | 3.39E-06 |
| genus | Ruminiclostridium6 | rs9555756 | C | A | -0.0804 | 0.0177 | 7.10E-06 |
| genus | Ruminiclostridium6 | rs792058 | A | G | 0.0554 | 0.0125 | 8.58E-06 |
| genus | Ruminiclostridium6 | rs10829821 | C | T | -0.0976 | 0.0216 | 3.47E-06 |
| genus | Ruminiclostridium6 | rs67479537 | C | T | 0.1190 | 0.0265 | 9.30E-06 |
| genus | Ruminiclostridium6 | rs1871858 | G | C | -0.1054 | 0.0237 | 9.12E-06 |
| genus | Ruminiclostridium6 | rs116969552 | G | A | -0.1668 | 0.0377 | 9.16E-06 |
| genus | Ruminiclostridium6 | rs2548459 | T | C | 0.0555 | 0.0123 | 6.40E-06 |
| genus | Ruminiclostridium6 | rs73176030 | C | T | 0.0587 | 0.0132 | 7.29E-06 |
| genus | Ruminiclostridium6 | rs35362464 | A | C | 0.0720 | 0.0165 | 8.99E-06 |
| genus | Ruminiclostridium9 | rs57665991 | G | C | -0.0642 | 0.0123 | 2.07E-07 |
| genus | Ruminiclostridium9 | rs12040548 | T | G | 0.0570 | 0.0122 | 3.15E-06 |
| genus | Ruminiclostridium9 | rs115044523 | A | G | -0.0980 | 0.0203 | 2.37E-06 |
| genus | Ruminiclostridium9 | rs12419854 | A | T | -0.0728 | 0.0156 | 3.18E-06 |
| genus | Ruminiclostridium9 | rs6082461 | C | A | 0.0586 | 0.0131 | 4.87E-06 |
| genus | Ruminiclostridium9 | rs113048721 | G | C | 0.0601 | 0.0133 | 4.10E-06 |
| genus | Ruminiclostridium9 | rs918449 | G | A | -0.0951 | 0.0197 | 2.56E-06 |
| genus | Ruminiclostridium9 | rs73592673 | T | A | -0.0816 | 0.0170 | 2.14E-06 |
| genus | Ruminiclostridium9 | rs79082720 | G | C | 0.0929 | 0.0205 | 6.47E-06 |
| genus | Ruminiclostridium9 | rs74303178 | C | T | 0.0533 | 0.0119 | 7.92E-06 |
| genus | Ruminiclostridium9 | rs13033315 | A | T | 0.0511 | 0.0111 | 5.68E-06 |
| genus | Ruminiclostridium9 | rs9809789 | T | C | -0.0717 | 0.0160 | 8.72E-06 |
| genus | Ruminiclostridium9 | rs9522712 | C | T | 0.0700 | 0.0155 | 4.66E-06 |
| genus | Ruminiclostridium9 | rs78191726 | C | T | 0.0945 | 0.0210 | 7.58E-06 |
| genus | Ruminiclostridium9 | rs7137760 | T | C | 0.0508 | 0.0112 | 7.07E-06 |
| genus | RuminococcaceaeNK4A214group | rs5994253 | G | A | -0.0811 | 0.0158 | 2.35E-07 |
| genus | RuminococcaceaeNK4A214group | rs136761 | A | G | -0.0588 | 0.0119 | 8.15E-07 |
| genus | RuminococcaceaeNK4A214group | rs11586410 | A | G | -0.0863 | 0.0170 | 3.66E-07 |
| genus | RuminococcaceaeNK4A214group | rs34576931 | C | G | -0.0873 | 0.0195 | 4.72E-06 |
| genus | RuminococcaceaeNK4A214group | rs10178772 | T | G | 0.0714 | 0.0162 | 7.72E-06 |
| genus | RuminococcaceaeNK4A214group | rs114244418 | G | C | -0.1753 | 0.0373 | 3.59E-06 |
| genus | RuminococcaceaeNK4A214group | rs4814689 | T | C | -0.1083 | 0.0231 | 4.55E-06 |
| genus | RuminococcaceaeNK4A214group | rs73158814 | G | C | -0.1093 | 0.0227 | 2.20E-06 |
| genus | RuminococcaceaeNK4A214group | rs12731 | G | A | -0.0528 | 0.0115 | 4.87E-06 |
| genus | RuminococcaceaeNK4A214group | rs7573569 | C | T | 0.1077 | 0.0234 | 3.23E-06 |
| genus | RuminococcaceaeNK4A214group | rs147475196 | G | A | -0.1338 | 0.0295 | 4.72E-06 |
| genus | RuminococcaceaeNK4A214group | rs62027366 | C | T | 0.0615 | 0.0138 | 6.58E-06 |
| genus | RuminococcaceaeNK4A214group | rs12642039 | C | T | -0.0553 | 0.0119 | 3.43E-06 |
| genus | RuminococcaceaeNK4A214group | rs35559912 | C | T | -0.0925 | 0.0204 | 4.89E-06 |
| genus | RuminococcaceaeNK4A214group | rs13087692 | G | T | 0.0575 | 0.0126 | 8.69E-06 |
| genus | RuminococcaceaeNK4A214group | rs6681678 | T | C | -0.1002 | 0.0240 | 9.05E-06 |
| genus | RuminococcaceaeNK4A214group | rs11241747 | T | C | 0.0534 | 0.0120 | 6.59E-06 |
| genus | RuminococcaceaeUCG002 | rs77564310 | C | A | -0.0713 | 0.0141 | 3.29E-07 |
| genus | RuminococcaceaeUCG002 | rs55793120 | C | T | 0.1374 | 0.0274 | 4.81E-07 |
| genus | RuminococcaceaeUCG002 | rs10927423 | A | C | -0.0714 | 0.0148 | 8.50E-07 |
| genus | RuminococcaceaeUCG002 | rs67746927 | G | C | -0.0542 | 0.0110 | 9.17E-07 |
| genus | RuminococcaceaeUCG002 | rs116974815 | A | C | -0.1897 | 0.0397 | 2.03E-06 |
| genus | RuminococcaceaeUCG002 | rs7155595 | A | C | 0.0570 | 0.0117 | 1.15E-06 |
| genus | RuminococcaceaeUCG002 | rs7120052 | C | A | 0.0625 | 0.0136 | 1.97E-06 |
| genus | RuminococcaceaeUCG002 | rs10916131 | T | C | -0.0693 | 0.0147 | 2.87E-06 |
| genus | RuminococcaceaeUCG002 | rs79016051 | T | C | -0.0888 | 0.0189 | 2.34E-06 |
| genus | RuminococcaceaeUCG002 | rs12463378 | G | A | -0.0522 | 0.0112 | 2.96E-06 |
| genus | RuminococcaceaeUCG002 | rs11750293 | T | G | -0.0578 | 0.0121 | 1.76E-06 |
| genus | RuminococcaceaeUCG002 | rs2265670 | G | C | -0.0512 | 0.0109 | 2.99E-06 |
| genus | RuminococcaceaeUCG002 | rs15256 | T | C | 0.0732 | 0.0168 | 9.46E-06 |
| genus | RuminococcaceaeUCG002 | rs882348 | G | A | -0.0800 | 0.0179 | 5.45E-06 |
| genus | RuminococcaceaeUCG002 | rs72874194 | C | G | -0.0770 | 0.0167 | 3.47E-06 |
| genus | RuminococcaceaeUCG002 | rs6793778 | T | C | -0.0559 | 0.0125 | 9.81E-06 |
| genus | RuminococcaceaeUCG002 | rs7342369 | A | C | -0.0528 | 0.0116 | 5.66E-06 |
| genus | RuminococcaceaeUCG002 | rs10964441 | A | G | -0.1491 | 0.0345 | 7.45E-06 |
| genus | RuminococcaceaeUCG002 | rs11607472 | G | A | -0.0780 | 0.0176 | 7.19E-06 |
| genus | RuminococcaceaeUCG002 | rs7249614 | G | A | -0.0493 | 0.0111 | 9.07E-06 |
| genus | RuminococcaceaeUCG002 | rs76847269 | G | A | 0.1635 | 0.0356 | 5.17E-06 |
| genus | RuminococcaceaeUCG002 | rs6542556 | G | A | 0.0510 | 0.0114 | 7.86E-06 |
| genus | RuminococcaceaeUCG002 | rs57079348 | G | T | -0.0766 | 0.0173 | 7.22E-06 |
| genus | RuminococcaceaeUCG002 | rs56030423 | A | G | -0.0983 | 0.0216 | 6.30E-06 |
| genus | RuminococcaceaeUCG002 | rs362417 | C | G | -0.0549 | 0.0121 | 7.80E-06 |
| genus | RuminococcaceaeUCG002 | rs113147300 | G | A | -0.0758 | 0.0165 | 7.69E-06 |
| genus | RuminococcaceaeUCG003 | rs73341549 | C | T | -0.1699 | 0.0319 | 1.51E-07 |
| genus | RuminococcaceaeUCG003 | rs6759615 | G | A | 0.1025 | 0.0200 | 7.86E-07 |
| genus | RuminococcaceaeUCG003 | rs646327 | A | G | 0.0587 | 0.0118 | 7.83E-07 |
| genus | RuminococcaceaeUCG003 | rs11613919 | T | G | 0.0728 | 0.0156 | 1.63E-06 |
| genus | RuminococcaceaeUCG003 | rs11243416 | C | T | -0.0926 | 0.0191 | 1.67E-06 |
| genus | RuminococcaceaeUCG003 | rs16959793 | C | A | -0.0625 | 0.0131 | 2.22E-06 |
| genus | RuminococcaceaeUCG003 | rs4452755 | C | A | -0.0634 | 0.0135 | 3.29E-06 |
| genus | RuminococcaceaeUCG003 | rs10490280 | T | C | -0.0672 | 0.0143 | 4.16E-06 |
| genus | RuminococcaceaeUCG003 | rs4532474 | A | G | 0.0769 | 0.0170 | 4.82E-06 |
| genus | RuminococcaceaeUCG003 | rs4629039 | A | T | 0.0554 | 0.0123 | 6.54E-06 |
| genus | RuminococcaceaeUCG003 | rs2523124 | C | T | -0.0547 | 0.0121 | 5.78E-06 |
| genus | RuminococcaceaeUCG003 | rs3013089 | A | G | -0.0551 | 0.0120 | 4.38E-06 |
| genus | RuminococcaceaeUCG003 | rs78720113 | G | A | -0.1153 | 0.0250 | 7.59E-06 |
| genus | RuminococcaceaeUCG003 | rs139730 | C | G | -0.0578 | 0.0131 | 9.72E-06 |
| genus | RuminococcaceaeUCG004 | rs6769553 | G | A | 0.0850 | 0.0157 | 7.91E-08 |
| genus | RuminococcaceaeUCG004 | rs12125734 | T | G | 0.1340 | 0.0257 | 2.09E-07 |
| genus | RuminococcaceaeUCG004 | rs511258 | A | G | -0.0757 | 0.0162 | 4.52E-06 |
| genus | RuminococcaceaeUCG004 | rs2248146 | C | T | 0.0689 | 0.0154 | 8.20E-06 |
| genus | RuminococcaceaeUCG004 | rs10976229 | G | T | 0.0960 | 0.0215 | 7.04E-06 |
| genus | RuminococcaceaeUCG004 | rs7569771 | G | A | -0.0759 | 0.0170 | 8.12E-06 |
| genus | RuminococcaceaeUCG004 | rs872501 | A | G | 0.1161 | 0.0259 | 5.81E-06 |
| genus | RuminococcaceaeUCG004 | rs550351 | C | A | 0.0786 | 0.0180 | 9.43E-06 |
| genus | RuminococcaceaeUCG004 | rs3800154 | C | A | -0.0798 | 0.0178 | 6.12E-06 |
| genus | RuminococcaceaeUCG004 | rs11961899 | A | G | -0.0708 | 0.0161 | 9.18E-06 |
| genus | RuminococcaceaeUCG004 | rs9818949 | T | G | 0.0860 | 0.0189 | 5.39E-06 |
| genus | RuminococcaceaeUCG004 | rs7123615 | G | C | -0.0786 | 0.0180 | 7.09E-06 |
| genus | RuminococcaceaeUCG005 | rs10950694 | C | T | 0.0578 | 0.0114 | 4.30E-07 |
| genus | RuminococcaceaeUCG005 | rs34781347 | A | G | 0.1887 | 0.0386 | 6.05E-07 |
| genus | RuminococcaceaeUCG005 | rs12288512 | G | A | 0.0666 | 0.0144 | 3.10E-06 |
| genus | RuminococcaceaeUCG005 | rs60081663 | G | C | 0.1581 | 0.0320 | 9.28E-07 |
| genus | RuminococcaceaeUCG005 | rs114279581 | G | A | -0.1466 | 0.0316 | 3.22E-06 |
| genus | RuminococcaceaeUCG005 | rs12458218 | C | T | 0.0677 | 0.0145 | 2.41E-06 |
| genus | RuminococcaceaeUCG005 | rs35166120 | G | C | -0.0686 | 0.0146 | 3.75E-06 |
| genus | RuminococcaceaeUCG005 | rs7555878 | G | A | 0.0587 | 0.0125 | 2.81E-06 |
| genus | RuminococcaceaeUCG005 | rs394449 | T | A | 0.0693 | 0.0149 | 2.60E-06 |
| genus | RuminococcaceaeUCG005 | rs10873449 | C | T | 0.0655 | 0.0144 | 4.11E-06 |
| genus | RuminococcaceaeUCG005 | rs2893871 | A | G | -0.0736 | 0.0155 | 3.54E-06 |
| genus | RuminococcaceaeUCG005 | rs72776570 | A | C | 0.0871 | 0.0197 | 5.36E-06 |
| genus | RuminococcaceaeUCG005 | rs898577 | C | T | -0.1230 | 0.0287 | 7.46E-06 |
| genus | RuminococcaceaeUCG005 | rs7449320 | A | C | 0.0599 | 0.0131 | 4.81E-06 |
| genus | RuminococcaceaeUCG005 | rs10937802 | A | G | 0.0756 | 0.0168 | 8.17E-06 |
| genus | RuminococcaceaeUCG005 | rs7586445 | A | G | 0.0782 | 0.0176 | 8.81E-06 |
| genus | RuminococcaceaeUCG005 | rs55793120 | C | T | 0.1215 | 0.0280 | 7.37E-06 |
| genus | RuminococcaceaeUCG009 | rs8009993 | C | G | -0.1359 | 0.0245 | 4.42E-08 |
| genus | RuminococcaceaeUCG009 | rs1550196 | A | G | 0.1308 | 0.0262 | 1.13E-06 |
| genus | RuminococcaceaeUCG009 | rs4708333 | G | T | -0.0840 | 0.0175 | 1.56E-06 |
| genus | RuminococcaceaeUCG009 | rs61779334 | C | G | -0.1381 | 0.0292 | 1.94E-06 |
| genus | RuminococcaceaeUCG009 | rs4079028 | T | C | 0.0916 | 0.0199 | 3.28E-06 |
| genus | RuminococcaceaeUCG009 | rs2058609 | G | A | 0.0816 | 0.0175 | 3.12E-06 |
| genus | RuminococcaceaeUCG009 | rs12508214 | T | C | -0.0775 | 0.0169 | 4.75E-06 |
| genus | RuminococcaceaeUCG009 | rs9558661 | C | T | -0.0898 | 0.0201 | 7.01E-06 |
| genus | RuminococcaceaeUCG009 | rs758191 | G | T | 0.1770 | 0.0375 | 9.01E-06 |
| genus | RuminococcaceaeUCG009 | rs2192926 | G | A | -0.0890 | 0.0193 | 4.88E-06 |
| genus | RuminococcaceaeUCG009 | rs113006825 | C | T | -0.0929 | 0.0208 | 7.98E-06 |
| genus | RuminococcaceaeUCG009 | rs138460696 | G | A | 0.1393 | 0.0316 | 9.81E-06 |
| genus | RuminococcaceaeUCG009 | rs6952765 | A | G | 0.0732 | 0.0167 | 8.13E-06 |
| genus | RuminococcaceaeUCG009 | rs78410648 | G | A | 0.1210 | 0.0277 | 9.67E-06 |
| genus | RuminococcaceaeUCG010 | rs35506912 | C | G | -0.0694 | 0.0148 | 3.21E-06 |
| genus | RuminococcaceaeUCG010 | rs682403 | G | A | -0.0588 | 0.0125 | 2.37E-06 |
| genus | RuminococcaceaeUCG010 | rs6958419 | T | C | -0.0586 | 0.0125 | 2.84E-06 |
| genus | RuminococcaceaeUCG010 | rs2820282 | C | A | -0.0592 | 0.0126 | 2.85E-06 |
| genus | RuminococcaceaeUCG010 | rs12597105 | A | G | 0.0671 | 0.0144 | 4.87E-06 |
| genus | RuminococcaceaeUCG010 | rs7935775 | T | A | -0.0631 | 0.0138 | 4.99E-06 |
| genus | RuminococcaceaeUCG010 | rs73218807 | A | G | -0.1662 | 0.0368 | 6.43E-06 |
| genus | RuminococcaceaeUCG010 | rs7441445 | T | C | -0.0569 | 0.0127 | 6.80E-06 |
| genus | RuminococcaceaeUCG011 | rs1416041 | C | A | -0.1823 | 0.0340 | 7.04E-08 |
| genus | RuminococcaceaeUCG011 | rs12724320 | T | C | -0.1209 | 0.0249 | 1.52E-06 |
| genus | RuminococcaceaeUCG011 | rs79113084 | T | C | -0.1522 | 0.0318 | 2.06E-06 |
| genus | RuminococcaceaeUCG011 | rs9729514 | G | A | 0.1849 | 0.0395 | 2.37E-06 |
| genus | RuminococcaceaeUCG011 | rs2729556 | T | C | -0.1091 | 0.0234 | 3.19E-06 |
| genus | RuminococcaceaeUCG011 | rs10274562 | T | C | 0.1109 | 0.0245 | 6.50E-06 |
| genus | RuminococcaceaeUCG011 | rs4490371 | C | T | -0.1118 | 0.0249 | 7.75E-06 |
| genus | RuminococcaceaeUCG011 | rs12636310 | A | G | 0.1327 | 0.0282 | 2.81E-06 |
| genus | RuminococcaceaeUCG013 | rs12781711 | T | C | -0.0656 | 0.0117 | 2.55E-08 |
| genus | RuminococcaceaeUCG013 | rs12189346 | A | G | 0.0685 | 0.0146 | 1.68E-06 |
| genus | RuminococcaceaeUCG013 | rs75088940 | C | T | -0.0943 | 0.0201 | 2.55E-06 |
| genus | RuminococcaceaeUCG013 | rs16918863 | C | A | 0.1115 | 0.0240 | 4.16E-06 |
| genus | RuminococcaceaeUCG013 | rs76973485 | T | G | 0.1950 | 0.0418 | 3.35E-06 |
| genus | RuminococcaceaeUCG013 | rs12485353 | A | G | -0.0608 | 0.0131 | 4.19E-06 |
| genus | RuminococcaceaeUCG013 | rs9565219 | A | T | -0.0525 | 0.0118 | 8.73E-06 |
| genus | RuminococcaceaeUCG013 | rs1729063 | C | G | -0.0533 | 0.0121 | 9.64E-06 |
| genus | RuminococcaceaeUCG013 | rs7784330 | A | G | -0.0498 | 0.0112 | 8.16E-06 |
| genus | RuminococcaceaeUCG013 | rs11581881 | T | C | 0.0661 | 0.0145 | 4.73E-06 |
| genus | RuminococcaceaeUCG013 | rs2428106 | G | C | -0.0491 | 0.0110 | 8.38E-06 |
| genus | RuminococcaceaeUCG013 | rs2730183 | A | G | -0.0489 | 0.0110 | 8.44E-06 |
| genus | RuminococcaceaeUCG013 | rs9313055 | C | T | 0.1051 | 0.0234 | 9.55E-06 |
| genus | RuminococcaceaeUCG013 | rs12336782 | C | T | -0.0856 | 0.0189 | 8.60E-06 |
| genus | RuminococcaceaeUCG013 | rs4385846 | T | G | 0.0598 | 0.0132 | 6.46E-06 |
| genus | RuminococcaceaeUCG014 | rs115777838 | C | T | -0.1883 | 0.0387 | 4.62E-07 |
| genus | RuminococcaceaeUCG014 | rs72809222 | C | T | 0.0672 | 0.0140 | 2.41E-06 |
| genus | RuminococcaceaeUCG014 | rs12638134 | G | T | 0.0583 | 0.0120 | 1.21E-06 |
| genus | RuminococcaceaeUCG014 | rs56105232 | A | G | 0.1393 | 0.0299 | 2.91E-06 |
| genus | RuminococcaceaeUCG014 | rs995642 | T | C | 0.0600 | 0.0126 | 1.90E-06 |
| genus | RuminococcaceaeUCG014 | rs10941294 | T | C | -0.1221 | 0.0260 | 2.40E-06 |
| genus | RuminococcaceaeUCG014 | rs79640386 | A | T | -0.1109 | 0.0248 | 8.74E-06 |
| genus | RuminococcaceaeUCG014 | rs439810 | C | G | -0.0577 | 0.0127 | 7.04E-06 |
| genus | RuminococcaceaeUCG014 | rs10495392 | T | C | -0.0825 | 0.0187 | 9.96E-06 |
| genus | RuminococcaceaeUCG014 | rs62478832 | A | T | -0.0581 | 0.0129 | 6.04E-06 |
| genus | RuminococcaceaeUCG014 | rs61898819 | T | A | 0.0608 | 0.0139 | 9.92E-06 |
| genus | RuminococcaceaeUCG014 | rs853612 | G | A | -0.0528 | 0.0119 | 9.75E-06 |
| genus | RuminococcaceaeUCG014 | rs73186226 | A | G | -0.0994 | 0.0217 | 6.72E-06 |
| genus | RuminococcaceaeUCG014 | rs34402072 | T | C | -0.0688 | 0.0156 | 9.80E-06 |
| genus | RuminococcaceaeUCG014 | rs74060145 | G | C | -0.1158 | 0.0254 | 8.71E-06 |
| genus | RuminococcaceaeUCG014 | rs77627087 | G | C | 0.0676 | 0.0150 | 7.43E-06 |
| genus | RuminococcaceaeUCG014 | rs17296933 | G | C | -0.0830 | 0.0185 | 7.34E-06 |
| genus | RuminococcaceaeUCG014 | rs10791168 | G | A | -0.0665 | 0.0150 | 9.76E-06 |
| genus | Ruminococcus1 | rs10769159 | C | G | -0.0640 | 0.0110 | 5.29E-09 |
| genus | Ruminococcus1 | rs7117576 | G | A | 0.0830 | 0.0171 | 6.48E-07 |
| genus | Ruminococcus1 | rs17781867 | T | C | 0.0999 | 0.0212 | 1.96E-06 |
| genus | Ruminococcus1 | rs7583465 | T | C | 0.0528 | 0.0113 | 2.56E-06 |
| genus | Ruminococcus1 | rs78613526 | A | G | 0.1675 | 0.0368 | 5.11E-06 |
| genus | Ruminococcus1 | rs78572139 | A | G | 0.1250 | 0.0279 | 5.23E-06 |
| genus | Ruminococcus1 | rs3000856 | A | T | -0.0710 | 0.0163 | 9.28E-06 |
| genus | Ruminococcus1 | rs6493760 | T | C | 0.0535 | 0.0116 | 3.38E-06 |
| genus | Ruminococcus1 | rs11783695 | T | G | -0.0734 | 0.0161 | 4.73E-06 |
| genus | Ruminococcus1 | rs10995816 | G | C | -0.0762 | 0.0175 | 8.38E-06 |
| genus | Ruminococcus1 | rs10167839 | G | A | 0.0520 | 0.0116 | 8.09E-06 |
| genus | Ruminococcus1 | rs3819978 | T | C | -0.1150 | 0.0260 | 8.74E-06 |
| genus | Ruminococcus1 | rs4849717 | A | T | 0.1328 | 0.0300 | 8.83E-06 |
| genus | Ruminococcus1 | rs6105066 | C | T | -0.0607 | 0.0134 | 5.06E-06 |
| genus | Ruminococcus2 | rs78120384 | G | A | -0.1928 | 0.0392 | 3.31E-07 |
| genus | Ruminococcus2 | rs12986628 | T | C | 0.0666 | 0.0140 | 2.14E-06 |
| genus | Ruminococcus2 | rs7635831 | A | G | 0.0618 | 0.0129 | 1.98E-06 |
| genus | Ruminococcus2 | rs2997412 | G | A | -0.0568 | 0.0122 | 4.22E-06 |
| genus | Ruminococcus2 | rs2368224 | G | T | 0.1996 | 0.0438 | 3.63E-06 |
| genus | Ruminococcus2 | rs4400279 | G | A | 0.0546 | 0.0120 | 5.80E-06 |
| genus | Ruminococcus2 | rs58681734 | G | A | 0.0724 | 0.0161 | 4.18E-06 |
| genus | Ruminococcus2 | rs1819812 | T | G | 0.0842 | 0.0185 | 5.28E-06 |
| genus | Ruminococcus2 | rs12406309 | C | A | -0.0632 | 0.0142 | 9.79E-06 |
| genus | Ruminococcus2 | rs2846589 | T | G | 0.0522 | 0.0116 | 7.59E-06 |
| genus | Ruminococcus2 | rs55707116 | A | C | 0.0866 | 0.0189 | 8.01E-06 |
| genus | Ruminococcus2 | rs75140805 | G | T | 0.0837 | 0.0176 | 3.95E-06 |
| genus | Ruminococcus2 | rs4799823 | T | C | 0.0837 | 0.0182 | 5.40E-06 |
| genus | Ruminococcus2 | rs61791565 | C | T | -0.0524 | 0.0117 | 6.79E-06 |
| genus | Ruminococcus2 | rs7693984 | A | G | -0.1028 | 0.0235 | 9.42E-06 |
| genus | Sellimonas | rs13417181 | C | T | 0.1665 | 0.0338 | 7.62E-07 |
| genus | Sellimonas | rs2371572 | C | A | 0.1273 | 0.0251 | 4.46E-07 |
| genus | Sellimonas | rs113379006 | C | T | -0.1628 | 0.0357 | 7.21E-06 |
| genus | Sellimonas | rs2187447 | C | A | 0.2435 | 0.0528 | 3.98E-06 |
| genus | Sellimonas | rs2016057 | C | A | -0.1259 | 0.0256 | 1.03E-06 |
| genus | Sellimonas | rs72553859 | C | G | -0.1505 | 0.0333 | 5.38E-06 |
| genus | Sellimonas | rs56203279 | C | T | -0.1240 | 0.0269 | 3.72E-06 |
| genus | Sellimonas | rs553697 | C | T | -0.1538 | 0.0339 | 6.13E-06 |
| genus | Sellimonas | rs6433790 | T | C | -0.1385 | 0.0302 | 3.98E-06 |
| genus | Sellimonas | rs7968030 | T | A | -0.1271 | 0.0281 | 5.56E-06 |
| genus | Sellimonas | rs17657328 | T | C | 0.1146 | 0.0254 | 8.25E-06 |
| genus | Sellimonas | rs41816 | G | A | 0.1322 | 0.0291 | 8.39E-06 |
| genus | Senegalimassilia | rs11787826 | A | C | 0.0813 | 0.0171 | 2.63E-06 |
| genus | Senegalimassilia | rs72887800 | A | T | -0.0822 | 0.0176 | 2.42E-06 |
| genus | Senegalimassilia | rs7225245 | A | G | 0.0792 | 0.0170 | 4.18E-06 |
| genus | Senegalimassilia | rs57512504 | A | T | 0.0819 | 0.0172 | 2.03E-06 |
| genus | Senegalimassilia | rs10036909 | T | C | 0.1855 | 0.0401 | 8.05E-06 |
| genus | Senegalimassilia | rs13383270 | C | G | 0.0775 | 0.0171 | 6.04E-06 |
| genus | Senegalimassilia | rs1990708 | C | A | -0.1096 | 0.0248 | 8.91E-06 |
| genus | Senegalimassilia | rs2017373 | T | C | 0.0782 | 0.0177 | 9.50E-06 |
| genus | Slackia | rs8901 | T | C | 0.0935 | 0.0187 | 6.07E-07 |
| genus | Slackia | rs4492265 | G | A | -0.0906 | 0.0192 | 2.41E-06 |
| genus | Slackia | rs16894137 | T | C | -0.1228 | 0.0263 | 2.71E-06 |
| genus | Slackia | rs112764253 | A | T | 0.1947 | 0.0412 | 3.40E-06 |
| genus | Slackia | rs12440440 | G | A | 0.0902 | 0.0191 | 2.63E-06 |
| genus | Slackia | rs35156985 | C | T | -0.1557 | 0.0348 | 8.06E-06 |
| genus | Slackia | rs10409783 | G | A | 0.0951 | 0.0211 | 7.70E-06 |
| genus | Slackia | rs13339230 | G | C | 0.1471 | 0.0331 | 7.42E-06 |
| genus | Slackia | rs58767323 | C | G | -0.1028 | 0.0227 | 4.60E-06 |
| genus | Streptococcus | rs11110281 | C | T | -0.1375 | 0.0227 | 2.58E-09 |
| genus | Streptococcus | rs4968759 | G | A | -0.0515 | 0.0112 | 3.78E-06 |
| genus | Streptococcus | rs11764382 | G | A | -0.0695 | 0.0144 | 1.29E-06 |
| genus | Streptococcus | rs72739637 | G | A | 0.0960 | 0.0193 | 1.03E-06 |
| genus | Streptococcus | rs1918540 | A | G | 0.0596 | 0.0128 | 2.44E-06 |
| genus | Streptococcus | rs17708276 | G | A | -0.0794 | 0.0171 | 3.04E-06 |
| genus | Streptococcus | rs77558518 | G | A | -0.1040 | 0.0230 | 4.71E-06 |
| genus | Streptococcus | rs11720390 | A | G | 0.1070 | 0.0228 | 3.59E-06 |
| genus | Streptococcus | rs395407 | C | G | -0.0793 | 0.0174 | 4.37E-06 |
| genus | Streptococcus | rs10448310 | G | A | -0.0518 | 0.0111 | 3.31E-06 |
| genus | Streptococcus | rs7916711 | G | A | 0.1029 | 0.0217 | 2.72E-06 |
| genus | Streptococcus | rs71481756 | G | T | 0.0931 | 0.0208 | 6.51E-06 |
| genus | Streptococcus | rs2370083 | T | G | -0.0817 | 0.0186 | 9.75E-06 |
| genus | Streptococcus | rs10028567 | T | C | -0.0921 | 0.0192 | 7.30E-06 |
| genus | Streptococcus | rs6806351 | C | T | -0.0634 | 0.0137 | 4.94E-06 |
| genus | Streptococcus | rs57646748 | A | G | -0.0908 | 0.0200 | 5.48E-06 |
| genus | Streptococcus | rs9903102 | A | C | -0.0709 | 0.0155 | 4.18E-06 |
| genus | Streptococcus | rs6563952 | C | G | 0.0827 | 0.0180 | 5.82E-06 |
| genus | Subdoligranulum | rs12638227 | C | G | -0.0557 | 0.0109 | 2.48E-07 |
| genus | Subdoligranulum | rs10065321 | C | T | -0.0513 | 0.0108 | 2.10E-06 |
| genus | Subdoligranulum | rs4347804 | G | A | 0.1661 | 0.0357 | 2.18E-06 |
| genus | Subdoligranulum | rs6555306 | C | T | -0.0741 | 0.0155 | 2.81E-06 |
| genus | Subdoligranulum | rs3761728 | G | T | -0.0543 | 0.0119 | 3.87E-06 |
| genus | Subdoligranulum | rs2114677 | T | C | -0.1042 | 0.0231 | 2.72E-06 |
| genus | Subdoligranulum | rs75158211 | C | T | -0.0723 | 0.0159 | 7.52E-06 |
| genus | Subdoligranulum | rs2171249 | T | C | 0.1067 | 0.0233 | 4.51E-06 |
| genus | Subdoligranulum | rs35940633 | A | G | -0.0511 | 0.0110 | 4.22E-06 |
| genus | Subdoligranulum | rs76528319 | T | G | -0.1433 | 0.0311 | 7.41E-06 |
| genus | Subdoligranulum | rs1667315 | A | G | 0.0485 | 0.0107 | 6.72E-06 |
| genus | Subdoligranulum | rs76664262 | A | T | 0.0834 | 0.0185 | 4.87E-06 |
| genus | Subdoligranulum | rs10497836 | T | C | -0.0524 | 0.0119 | 8.38E-06 |
| genus | Subdoligranulum | rs16962433 | T | A | 0.0856 | 0.0189 | 7.65E-06 |
| genus | Sutterella | rs13173038 | G | A | -0.0718 | 0.0152 | 2.73E-06 |
| genus | Sutterella | rs2321387 | A | G | -0.0593 | 0.0125 | 1.87E-06 |
| genus | Sutterella | rs7499539 | G | A | 0.0617 | 0.0131 | 2.36E-06 |
| genus | Sutterella | rs62501473 | A | G | 0.0694 | 0.0149 | 5.52E-06 |
| genus | Sutterella | rs143438747 | C | T | -0.1458 | 0.0307 | 3.28E-06 |
| genus | Sutterella | rs9350083 | G | T | -0.0593 | 0.0134 | 8.23E-06 |
| genus | Sutterella | rs7638039 | C | T | 0.0646 | 0.0144 | 8.66E-06 |
| genus | Sutterella | rs11591622 | G | T | -0.0688 | 0.0151 | 6.50E-06 |
| genus | Sutterella | rs2613606 | T | C | -0.0557 | 0.0124 | 7.20E-06 |
| genus | Sutterella | rs1145877 | G | A | -0.0735 | 0.0162 | 7.20E-06 |
| genus | Sutterella | rs607327 | T | C | 0.0578 | 0.0129 | 6.63E-06 |
| genus | Sutterella | rs2050185 | A | G | 0.0575 | 0.0129 | 7.97E-06 |
| genus | Terrisporobacter | rs1883097 | T | C | 0.2264 | 0.0455 | 4.16E-07 |
| genus | Terrisporobacter | rs2569953 | C | A | -0.0776 | 0.0175 | 8.95E-06 |
| genus | Terrisporobacter | rs7184125 | C | T | 0.0913 | 0.0205 | 8.48E-06 |
| genus | Terrisporobacter | rs2872237 | A | C | -0.0815 | 0.0176 | 3.97E-06 |
| genus | Terrisporobacter | rs58405430 | T | G | 0.1346 | 0.0301 | 7.94E-06 |
| genus | Terrisporobacter | rs7034891 | C | G | -0.0799 | 0.0174 | 4.54E-06 |
| genus | Turicibacter | rs149744580 | G | A | 0.1699 | 0.0315 | 7.01E-08 |
| genus | Turicibacter | rs4869133 | A | G | 0.1312 | 0.0272 | 2.55E-06 |
| genus | Turicibacter | rs12603364 | C | T | 0.1109 | 0.0226 | 8.67E-07 |
| genus | Turicibacter | rs11054680 | C | T | -0.1048 | 0.0227 | 2.31E-06 |
| genus | Turicibacter | rs55756211 | C | T | -0.1151 | 0.0241 | 2.81E-06 |
| genus | Turicibacter | rs61265175 | C | G | -0.0859 | 0.0186 | 4.14E-06 |
| genus | Turicibacter | rs7199484 | A | G | -0.0731 | 0.0160 | 5.77E-06 |
| genus | Turicibacter | rs11649454 | C | G | 0.0951 | 0.0203 | 3.27E-06 |
| genus | Turicibacter | rs2834977 | C | T | -0.0960 | 0.0208 | 3.96E-06 |
| genus | Turicibacter | rs2952020 | A | G | -0.0759 | 0.0166 | 5.63E-06 |
| genus | Turicibacter | rs11666533 | T | C | -0.1117 | 0.0248 | 7.37E-06 |
| genus | Turicibacter | rs4247078 | G | C | 0.0710 | 0.0155 | 5.46E-06 |
| genus | Turicibacter | rs2221441 | C | G | 0.0710 | 0.0153 | 3.46E-06 |
| genus | Turicibacter | rs3734633 | A | G | -0.1210 | 0.0268 | 5.32E-06 |
| genus | Tyzzerella3 | rs67476743 | G | T | 0.1322 | 0.0222 | 3.74E-09 |
| genus | Tyzzerella3 | rs17706273 | C | T | -0.1404 | 0.0275 | 5.88E-07 |
| genus | Tyzzerella3 | rs7561370 | C | T | 0.1313 | 0.0286 | 1.52E-06 |
| genus | Tyzzerella3 | rs55799124 | G | A | -0.1144 | 0.0239 | 1.34E-06 |
| genus | Tyzzerella3 | rs17809157 | T | A | -0.1638 | 0.0336 | 1.54E-06 |
| genus | Tyzzerella3 | rs7019909 | C | T | 0.1442 | 0.0302 | 1.76E-06 |
| genus | Tyzzerella3 | rs75091807 | T | G | -0.1850 | 0.0383 | 1.71E-06 |
| genus | Tyzzerella3 | rs4904512 | C | T | -0.1172 | 0.0250 | 3.09E-06 |
| genus | Tyzzerella3 | rs7333521 | C | T | -0.2072 | 0.0453 | 4.88E-06 |
| genus | Tyzzerella3 | rs112102233 | G | A | -0.2163 | 0.0478 | 6.18E-06 |
| genus | Tyzzerella3 | rs1232220 | T | G | -0.1439 | 0.0318 | 7.91E-06 |
| genus | Tyzzerella3 | rs191093 | A | G | 0.1590 | 0.0353 | 6.76E-06 |
| genus | Tyzzerella3 | rs10898797 | T | C | 0.1224 | 0.0275 | 8.85E-06 |
| genus | Tyzzerella3 | rs6920448 | T | C | -0.1411 | 0.0305 | 4.15E-06 |
| genus | Veillonella | rs2013594 | C | T | -0.0721 | 0.0155 | 3.42E-06 |
| genus | Veillonella | rs12679709 | G | C | -0.0793 | 0.0165 | 1.78E-06 |
| genus | Veillonella | rs742016 | G | A | -0.0689 | 0.0150 | 4.66E-06 |
| genus | Veillonella | rs1882878 | G | A | -0.0769 | 0.0164 | 2.98E-06 |
| genus | Veillonella | rs7645873 | T | A | 0.0761 | 0.0164 | 3.12E-06 |
| genus | Veillonella | rs55807413 | G | A | 0.1073 | 0.0238 | 5.51E-06 |
| genus | Veillonella | rs62376424 | T | C | -0.0762 | 0.0163 | 3.65E-06 |
| genus | Veillonella | rs7359080 | A | C | -0.1354 | 0.0304 | 7.40E-06 |
| genus | Veillonella | rs11141494 | A | G | -0.0783 | 0.0174 | 9.75E-06 |
| genus | Veillonella | rs6656807 | G | A | 0.0703 | 0.0154 | 5.50E-06 |
| genus | Veillonella | rs11614532 | C | G | 0.0745 | 0.0165 | 7.13E-06 |
| genus | Victivallis | rs56349194 | G | A | -0.1585 | 0.0315 | 6.26E-07 |
| genus | Victivallis | rs592514 | A | T | -0.1814 | 0.0392 | 2.60E-06 |
| genus | Victivallis | rs911666 | C | T | -0.1186 | 0.0263 | 7.65E-06 |
| genus | Victivallis | rs7374366 | A | G | -0.1823 | 0.0405 | 7.23E-06 |
| genus | Victivallis | rs4764863 | A | G | 0.1216 | 0.0246 | 8.22E-07 |
| genus | Victivallis | rs12512543 | C | A | -0.1780 | 0.0374 | 2.54E-06 |
| genus | Victivallis | rs342302 | G | A | -0.1528 | 0.0352 | 8.16E-06 |
| genus | Victivallis | rs4895919 | C | T | -0.1169 | 0.0248 | 2.75E-06 |
| genus | Victivallis | rs11899949 | A | G | 0.1306 | 0.0276 | 2.77E-06 |
| genus | Victivallis | rs6445926 | C | G | 0.1170 | 0.0250 | 2.96E-06 |
| genus | Victivallis | rs173120 | C | T | 0.1338 | 0.0290 | 7.65E-06 |
| genus | Victivallis | rs2546432 | C | T | -0.1108 | 0.0250 | 9.93E-06 |
| genus | Victivallis | rs1882775 | G | A | -0.1383 | 0.0313 | 8.73E-06 |
| order | Actinomycetales | rs35011108 | G | A | 0.2415 | 0.0504 | 1.88E-06 |
| order | Actinomycetales | rs2889192 | T | G | 0.0884 | 0.0195 | 3.97E-06 |
| order | Actinomycetales | rs34583783 | T | G | 0.1237 | 0.0264 | 5.54E-06 |
| order | Actinomycetales | rs4073240 | A | G | 0.0750 | 0.0165 | 5.68E-06 |
| order | Actinomycetales | rs58484246 | C | T | 0.0758 | 0.0169 | 6.67E-06 |
| order | Bacillales | rs74887130 | G | C | -0.1922 | 0.0359 | 1.16E-07 |
| order | Bacillales | rs4617108 | G | A | 0.2489 | 0.0526 | 1.98E-06 |
| order | Bacillales | rs62640857 | G | A | 0.1482 | 0.0326 | 4.49E-06 |
| order | Bacillales | rs1287018 | A | G | 0.1411 | 0.0319 | 9.87E-06 |
| order | Bacillales | rs10410917 | C | T | 0.1147 | 0.0250 | 5.57E-06 |
| order | Bacillales | rs74420793 | G | A | -0.1644 | 0.0354 | 3.07E-06 |
| order | Bacillales | rs11034576 | G | A | 0.2058 | 0.0453 | 8.86E-06 |
| order | Bacillales | rs10233278 | C | T | -0.1163 | 0.0249 | 3.51E-06 |
| order | Bacillales | rs11207728 | A | G | -0.1446 | 0.0317 | 5.73E-06 |
| order | Bacillales | rs11844714 | G | A | -0.1432 | 0.0320 | 5.06E-06 |
| order | Bacillales | rs12522021 | A | T | 0.1743 | 0.0401 | 7.60E-06 |
| order | Bacteroidales | rs55773148 | A | G | -0.1215 | 0.0237 | 3.90E-07 |
| order | Bacteroidales | rs7631304 | A | G | -0.0646 | 0.0133 | 8.37E-07 |
| order | Bacteroidales | rs73975615 | A | G | -0.2070 | 0.0443 | 1.22E-06 |
| order | Bacteroidales | rs111845179 | C | T | 0.1026 | 0.0214 | 9.24E-07 |
| order | Bacteroidales | rs2032750 | C | T | -0.0508 | 0.0107 | 1.92E-06 |
| order | Bacteroidales | rs7546249 | A | T | -0.0567 | 0.0118 | 1.55E-06 |
| order | Bacteroidales | rs929878 | T | C | -0.0549 | 0.0122 | 4.73E-06 |
| order | Bacteroidales | rs13291169 | G | C | 0.0690 | 0.0149 | 3.75E-06 |
| order | Bacteroidales | rs17343978 | C | A | -0.0552 | 0.0120 | 8.36E-06 |
| order | Bacteroidales | rs72706335 | C | T | -0.2224 | 0.0493 | 7.66E-06 |
| order | Bacteroidales | rs11146701 | G | A | 0.0474 | 0.0105 | 7.08E-06 |
| order | Bacteroidales | rs62531359 | G | T | 0.0656 | 0.0150 | 9.09E-06 |
| order | Bacteroidales | rs4146051 | G | A | -0.1074 | 0.0246 | 8.76E-06 |
| order | Bacteroidales | rs79585701 | C | A | 0.0647 | 0.0150 | 9.99E-06 |
| order | Bacteroidales | rs4916508 | A | G | -0.0467 | 0.0105 | 8.47E-06 |
| order | Bacteroidales | rs62575403 | T | C | 0.1401 | 0.0311 | 7.06E-06 |
| order | Bifidobacteriales | rs182549 | T | C | 0.1171 | 0.0127 | 5.94E-20 |
| order | Bifidobacteriales | rs7322849 | C | T | 0.1107 | 0.0201 | 1.74E-08 |
| order | Bifidobacteriales | rs1961273 | T | C | 0.0688 | 0.0132 | 1.70E-07 |
| order | Bifidobacteriales | rs76671854 | G | C | -0.0893 | 0.0183 | 1.04E-06 |
| order | Bifidobacteriales | rs4957061 | C | T | 0.0570 | 0.0117 | 1.15E-06 |
| order | Bifidobacteriales | rs10841473 | C | G | -0.0613 | 0.0129 | 2.28E-06 |
| order | Bifidobacteriales | rs4567981 | A | T | 0.0578 | 0.0117 | 8.88E-07 |
| order | Bifidobacteriales | rs13020688 | A | G | 0.0584 | 0.0122 | 1.57E-06 |
| order | Bifidobacteriales | rs677208 | C | G | 0.0729 | 0.0165 | 6.00E-06 |
| order | Bifidobacteriales | rs12446429 | C | T | 0.0810 | 0.0191 | 8.53E-06 |
| order | Bifidobacteriales | rs11655079 | C | T | -0.0586 | 0.0130 | 5.92E-06 |
| order | Bifidobacteriales | rs540489 | G | T | -0.0633 | 0.0138 | 5.37E-06 |
| order | Bifidobacteriales | rs857444 | T | C | 0.0554 | 0.0121 | 3.82E-06 |
| order | Bifidobacteriales | rs10857328 | A | T | 0.0562 | 0.0126 | 7.24E-06 |
| order | Bifidobacteriales | rs10831953 | A | G | 0.0538 | 0.0124 | 9.95E-06 |
| order | Bifidobacteriales | rs7174549 | T | C | 0.0552 | 0.0125 | 6.87E-06 |
| order | Bifidobacteriales | rs73797465 | G | T | -0.0943 | 0.0208 | 4.85E-06 |
| order | Bifidobacteriales | rs55888705 | G | A | 0.0537 | 0.0121 | 8.66E-06 |
| order | Bifidobacteriales | rs6899771 | G | A | -0.0914 | 0.0203 | 7.28E-06 |
| order | Bifidobacteriales | rs7588568 | C | A | -0.0607 | 0.0131 | 3.73E-06 |
| order | Burkholderiales | rs62191117 | G | A | 0.0680 | 0.0132 | 2.79E-07 |
| order | Burkholderiales | rs6087811 | G | T | -0.1016 | 0.0199 | 2.88E-07 |
| order | Burkholderiales | rs4033856 | T | C | 0.0833 | 0.0167 | 5.67E-07 |
| order | Burkholderiales | rs2367850 | C | G | 0.0633 | 0.0131 | 1.24E-06 |
| order | Burkholderiales | rs62395635 | C | T | 0.1099 | 0.0236 | 2.90E-06 |
| order | Burkholderiales | rs2321387 | A | G | -0.0509 | 0.0110 | 3.26E-06 |
| order | Burkholderiales | rs7638039 | C | T | 0.0581 | 0.0127 | 4.84E-06 |
| order | Burkholderiales | rs2613606 | T | C | -0.0500 | 0.0109 | 4.13E-06 |
| order | Burkholderiales | rs72747231 | G | C | -0.1337 | 0.0291 | 3.76E-06 |
| order | Burkholderiales | rs1928341 | G | A | 0.0508 | 0.0111 | 4.52E-06 |
| order | Burkholderiales | rs9964679 | G | A | 0.0525 | 0.0115 | 6.06E-06 |
| order | Burkholderiales | rs1511453 | G | A | 0.0911 | 0.0199 | 8.00E-06 |
| order | Burkholderiales | rs75242906 | T | C | -0.1210 | 0.0281 | 9.75E-06 |
| order | Clostridiales | rs6815608 | C | T | 0.1041 | 0.0211 | 3.72E-07 |
| order | Clostridiales | rs112334273 | A | G | 0.0639 | 0.0127 | 4.07E-07 |
| order | Clostridiales | rs6814436 | C | T | 0.0742 | 0.0151 | 9.06E-07 |
| order | Clostridiales | rs72915163 | C | T | -0.0580 | 0.0121 | 1.39E-06 |
| order | Clostridiales | rs2273429 | G | A | -0.0726 | 0.0153 | 4.17E-06 |
| order | Clostridiales | rs10774377 | G | A | 0.0523 | 0.0114 | 3.81E-06 |
| order | Clostridiales | rs13179700 | C | T | 0.0511 | 0.0110 | 3.52E-06 |
| order | Clostridiales | rs6934062 | C | G | 0.0522 | 0.0116 | 7.09E-06 |
| order | Clostridiales | rs10209007 | C | G | 0.0795 | 0.0182 | 9.60E-06 |
| order | Clostridiales | rs1842454 | A | G | -0.0544 | 0.0127 | 9.92E-06 |
| order | Clostridiales | rs76767978 | A | G | 0.0700 | 0.0156 | 8.18E-06 |
| order | Clostridiales | rs13105690 | C | T | -0.0527 | 0.0118 | 9.37E-06 |
| order | Clostridiales | rs7269302 | T | A | -0.0965 | 0.0212 | 8.84E-06 |
| order | Clostridiales | rs72738886 | C | T | 0.0865 | 0.0190 | 8.42E-06 |
| order | Clostridiales | rs76860606 | G | C | 0.0941 | 0.0225 | 8.58E-06 |
| order | Clostridiales | rs290772 | A | G | 0.0843 | 0.0196 | 1.00E-05 |
| order | Clostridiales | rs6442336 | T | C | -0.0548 | 0.0124 | 9.63E-06 |
| order | Coriobacteriales | rs719099 | G | A | 0.0778 | 0.0156 | 5.43E-07 |
| order | Coriobacteriales | rs240104 | C | T | -0.0603 | 0.0127 | 1.52E-06 |
| order | Coriobacteriales | rs11250875 | C | T | 0.0607 | 0.0131 | 4.83E-06 |
| order | Coriobacteriales | rs34739816 | T | G | 0.0965 | 0.0208 | 3.88E-06 |
| order | Coriobacteriales | rs76779974 | G | C | 0.0775 | 0.0172 | 5.63E-06 |
| order | Coriobacteriales | rs80046645 | G | C | 0.2558 | 0.0562 | 4.66E-06 |
| order | Coriobacteriales | rs45480394 | G | T | -0.0504 | 0.0113 | 9.71E-06 |
| order | Coriobacteriales | rs1816223 | G | A | -0.0586 | 0.0129 | 4.84E-06 |
| order | Coriobacteriales | rs7898585 | C | T | 0.0732 | 0.0164 | 5.25E-06 |
| order | Coriobacteriales | rs67561917 | G | A | -0.0714 | 0.0154 | 5.39E-06 |
| order | Coriobacteriales | rs3025411 | G | A | 0.0927 | 0.0209 | 8.27E-06 |
| order | Coriobacteriales | rs11656361 | C | A | 0.0773 | 0.0176 | 8.02E-06 |
| order | Coriobacteriales | rs1397793 | A | G | -0.0499 | 0.0112 | 9.77E-06 |
| order | Coriobacteriales | rs11073596 | G | T | 0.0510 | 0.0114 | 8.14E-06 |
| order | Coriobacteriales | rs2442778 | A | G | -0.1164 | 0.0259 | 9.03E-06 |
| order | Coriobacteriales | rs7540303 | T | C | 0.0483 | 0.0109 | 9.62E-06 |
| order | Coriobacteriales | rs62448869 | A | T | -0.0486 | 0.0109 | 7.87E-06 |
| order | Coriobacteriales | rs13307134 | T | C | 0.0566 | 0.0126 | 7.80E-06 |
| order | Coriobacteriales | rs4880783 | T | G | 0.0565 | 0.0125 | 5.90E-06 |
| order | Coriobacteriales | rs12974142 | A | G | 0.0790 | 0.0177 | 8.51E-06 |
| order | Coriobacteriales | rs8010111 | A | G | -0.1034 | 0.0229 | 6.90E-06 |
| order | Desulfovibrionales | rs6058181 | T | C | 0.0837 | 0.0166 | 2.53E-07 |
| order | Desulfovibrionales | rs4506934 | T | C | -0.0953 | 0.0201 | 2.43E-06 |
| order | Desulfovibrionales | rs11599763 | C | T | -0.0555 | 0.0117 | 2.61E-06 |
| order | Desulfovibrionales | rs2838334 | A | G | 0.0569 | 0.0124 | 4.17E-06 |
| order | Desulfovibrionales | rs9928243 | A | C | -0.0545 | 0.0118 | 3.97E-06 |
| order | Desulfovibrionales | rs112381107 | T | C | 0.2101 | 0.0457 | 3.22E-06 |
| order | Desulfovibrionales | rs16851319 | C | G | -0.0729 | 0.0151 | 2.70E-06 |
| order | Desulfovibrionales | rs17791387 | G | A | -0.0728 | 0.0154 | 2.25E-06 |
| order | Desulfovibrionales | rs186073 | C | T | 0.0529 | 0.0119 | 8.74E-06 |
| order | Desulfovibrionales | rs2692012 | G | A | 0.1122 | 0.0254 | 2.27E-06 |
| order | Desulfovibrionales | rs62020470 | G | A | -0.0574 | 0.0129 | 7.51E-06 |
| order | Desulfovibrionales | rs3935584 | T | C | -0.0524 | 0.0116 | 7.20E-06 |
| order | Desulfovibrionales | rs72647048 | C | T | -0.0772 | 0.0171 | 9.00E-06 |
| order | Enterobacteriales | rs78143293 | G | A | -0.0849 | 0.0170 | 1.20E-06 |
| order | Enterobacteriales | rs111229068 | T | A | 0.1106 | 0.0242 | 3.65E-06 |
| order | Enterobacteriales | rs62210023 | G | A | 0.0607 | 0.0130 | 3.13E-06 |
| order | Enterobacteriales | rs504442 | G | T | 0.0842 | 0.0189 | 5.17E-06 |
| order | Enterobacteriales | rs80319214 | G | C | 0.0990 | 0.0216 | 6.95E-06 |
| order | Enterobacteriales | rs11026530 | C | T | 0.0822 | 0.0186 | 9.43E-06 |
| order | Enterobacteriales | rs2374342 | A | C | 0.0583 | 0.0126 | 4.52E-06 |
| order | Enterobacteriales | rs79757635 | A | C | 0.0759 | 0.0171 | 9.32E-06 |
| order | Enterobacteriales | rs35673018 | A | G | 0.0900 | 0.0203 | 7.63E-06 |
| order | Enterobacteriales | rs61973590 | G | C | -0.0605 | 0.0134 | 8.54E-06 |
| order | Enterobacteriales | rs4792380 | T | A | 0.1156 | 0.0258 | 9.49E-06 |
| order | Erysipelotrichales | rs62504403 | T | C | 0.0681 | 0.0128 | 1.12E-07 |
| order | Erysipelotrichales | rs7234058 | C | T | -0.0946 | 0.0194 | 9.12E-07 |
| order | Erysipelotrichales | rs17530232 | G | A | 0.1030 | 0.0225 | 2.79E-06 |
| order | Erysipelotrichales | rs2300774 | A | G | 0.0524 | 0.0107 | 8.95E-07 |
| order | Erysipelotrichales | rs35161940 | C | T | -0.0806 | 0.0168 | 1.85E-06 |
| order | Erysipelotrichales | rs10781552 | T | C | -0.0552 | 0.0116 | 2.33E-06 |
| order | Erysipelotrichales | rs8003149 | T | C | 0.0539 | 0.0117 | 4.08E-06 |
| order | Erysipelotrichales | rs4078432 | T | C | -0.0609 | 0.0134 | 4.23E-06 |
| order | Erysipelotrichales | rs1074800 | G | A | 0.0492 | 0.0109 | 6.15E-06 |
| order | Erysipelotrichales | rs56970041 | G | T | 0.0724 | 0.0164 | 5.40E-06 |
| order | Erysipelotrichales | rs7826267 | G | T | -0.0839 | 0.0199 | 9.28E-06 |
| order | Erysipelotrichales | rs1884466 | T | C | -0.0475 | 0.0107 | 9.53E-06 |
| order | Erysipelotrichales | rs290833 | G | T | -0.0497 | 0.0111 | 8.03E-06 |
| order | Gastranaerophilales | rs9864379 | C | T | -0.1605 | 0.0293 | 4.66E-08 |
| order | Gastranaerophilales | rs11150282 | C | T | 0.0982 | 0.0197 | 7.36E-07 |
| order | Gastranaerophilales | rs16851659 | C | G | -0.0895 | 0.0187 | 1.27E-06 |
| order | Gastranaerophilales | rs4129395 | A | G | 0.0904 | 0.0185 | 1.22E-06 |
| order | Gastranaerophilales | rs1221147 | A | T | 0.1263 | 0.0279 | 4.86E-06 |
| order | Gastranaerophilales | rs79790072 | C | T | 0.2261 | 0.0488 | 3.54E-06 |
| order | Gastranaerophilales | rs73074665 | T | A | 0.1646 | 0.0356 | 3.62E-06 |
| order | Gastranaerophilales | rs113884518 | C | T | -0.2059 | 0.0455 | 7.74E-06 |
| order | Gastranaerophilales | rs789069 | C | A | -0.1041 | 0.0234 | 6.50E-06 |
| order | Gastranaerophilales | rs367480 | A | G | -0.0842 | 0.0186 | 7.52E-06 |
| order | Gastranaerophilales | rs8028558 | G | A | 0.0835 | 0.0189 | 9.78E-06 |
| order | Gastranaerophilales | rs28678345 | C | T | 0.2131 | 0.0471 | 8.06E-06 |
| order | Lactobacillales | rs2952251 | G | A | -0.0629 | 0.0124 | 3.36E-07 |
| order | Lactobacillales | rs35344081 | A | G | 0.0642 | 0.0127 | 4.16E-07 |
| order | Lactobacillales | rs76717940 | A | T | 0.1609 | 0.0327 | 6.55E-07 |
| order | Lactobacillales | rs77558518 | G | A | -0.1065 | 0.0223 | 1.67E-06 |
| order | Lactobacillales | rs11110282 | G | A | -0.1024 | 0.0218 | 3.96E-06 |
| order | Lactobacillales | rs9581006 | T | C | 0.2258 | 0.0469 | 1.77E-06 |
| order | Lactobacillales | rs4028634 | C | T | 0.0533 | 0.0110 | 1.35E-06 |
| order | Lactobacillales | rs74352383 | A | T | -0.1034 | 0.0218 | 1.88E-06 |
| order | Lactobacillales | rs78938557 | C | T | 0.1055 | 0.0234 | 2.31E-06 |
| order | Lactobacillales | rs57872228 | T | C | -0.0688 | 0.0147 | 2.58E-06 |
| order | Lactobacillales | rs1962325 | G | C | 0.0547 | 0.0115 | 1.96E-06 |
| order | Lactobacillales | rs11730038 | A | G | -0.0605 | 0.0129 | 5.10E-06 |
| order | Lactobacillales | rs2370083 | T | G | -0.0805 | 0.0181 | 8.33E-06 |
| order | Lactobacillales | rs12797734 | C | T | 0.0571 | 0.0127 | 7.77E-06 |
| order | Lactobacillales | rs34989881 | G | A | 0.1135 | 0.0246 | 4.09E-06 |
| order | Lactobacillales | rs11627423 | A | C | 0.0499 | 0.0110 | 5.09E-06 |
| order | Lactobacillales | rs111552159 | G | C | 0.1289 | 0.0287 | 8.31E-06 |
| order | Lactobacillales | rs1595463 | A | C | 0.0480 | 0.0109 | 7.44E-06 |
| order | Lactobacillales | rs74663707 | T | C | 0.0983 | 0.0225 | 8.40E-06 |
| order | Methanobacteriales | rs10202904 | G | T | -0.1218 | 0.0235 | 3.01E-07 |
| order | Methanobacteriales | rs73457410 | G | A | 0.2153 | 0.0437 | 1.41E-06 |
| order | Methanobacteriales | rs6776814 | C | T | -0.1996 | 0.0412 | 1.63E-06 |
| order | Methanobacteriales | rs894996 | A | C | 0.2170 | 0.0449 | 1.88E-06 |
| order | Methanobacteriales | rs75208022 | T | C | -0.2272 | 0.0488 | 5.92E-06 |
| order | Methanobacteriales | rs12825290 | G | C | -0.2168 | 0.0493 | 6.08E-06 |
| order | Methanobacteriales | rs56131665 | A | G | 0.1787 | 0.0393 | 6.18E-06 |
| order | Methanobacteriales | rs11018665 | T | A | 0.1115 | 0.0251 | 6.52E-06 |
| order | Methanobacteriales | rs4257531 | A | G | 0.1645 | 0.0365 | 7.44E-06 |
| order | Methanobacteriales | rs6508769 | C | T | 0.1535 | 0.0345 | 8.23E-06 |
| order | Methanobacteriales | rs73068003 | T | G | -0.1581 | 0.0352 | 8.45E-06 |
| order | Methanobacteriales | rs10424197 | A | G | -0.1113 | 0.0248 | 9.28E-06 |
| order | MollicutesRF9 | rs74603314 | C | T | 0.2308 | 0.0490 | 2.28E-06 |
| order | MollicutesRF9 | rs76373661 | A | G | 0.0908 | 0.0203 | 5.16E-06 |
| order | MollicutesRF9 | rs17235252 | C | T | -0.1220 | 0.0255 | 2.16E-06 |
| order | MollicutesRF9 | rs638542 | A | G | -0.0706 | 0.0157 | 5.17E-06 |
| order | MollicutesRF9 | rs62188991 | C | G | -0.1109 | 0.0241 | 5.27E-06 |
| order | MollicutesRF9 | rs949341 | A | G | 0.0657 | 0.0147 | 7.73E-06 |
| order | MollicutesRF9 | rs13100746 | T | C | 0.0639 | 0.0143 | 7.29E-06 |
| order | MollicutesRF9 | rs515984 | C | T | -0.0875 | 0.0191 | 6.61E-06 |
| order | MollicutesRF9 | rs7706512 | A | G | 0.0657 | 0.0139 | 2.27E-06 |
| order | MollicutesRF9 | rs11779863 | A | G | -0.0773 | 0.0172 | 6.69E-06 |
| order | MollicutesRF9 | rs7801843 | G | A | -0.0869 | 0.0195 | 9.47E-06 |
| order | MollicutesRF9 | rs10071529 | C | G | 0.1251 | 0.0279 | 8.64E-06 |
| order | MollicutesRF9 | rs739151 | G | C | 0.0653 | 0.0140 | 3.09E-06 |
| order | MollicutesRF9 | rs12566890 | G | T | -0.1031 | 0.0242 | 8.11E-06 |
| order | MollicutesRF9 | rs3932485 | T | C | 0.0626 | 0.0142 | 9.93E-06 |
| order | MollicutesRF9 | rs7853673 | A | G | -0.0624 | 0.0140 | 6.73E-06 |
| order | NB1n | rs60583455 | C | T | 0.1089 | 0.0212 | 2.60E-07 |
| order | NB1n | rs2172426 | T | C | -0.1021 | 0.0199 | 3.17E-07 |
| order | NB1n | rs11251024 | A | G | 0.1042 | 0.0207 | 6.63E-07 |
| order | NB1n | rs13219468 | C | G | 0.1153 | 0.0237 | 1.41E-06 |
| order | NB1n | rs267959 | G | A | 0.0989 | 0.0210 | 2.62E-06 |
| order | NB1n | rs11606187 | G | A | -0.1545 | 0.0326 | 3.31E-06 |
| order | NB1n | rs7911787 | T | G | -0.2231 | 0.0470 | 3.39E-06 |
| order | NB1n | rs72671304 | C | T | 0.1723 | 0.0370 | 3.80E-06 |
| order | NB1n | rs13385922 | C | T | 0.0929 | 0.0201 | 3.97E-06 |
| order | NB1n | rs4383094 | C | T | 0.1492 | 0.0321 | 4.28E-06 |
| order | NB1n | rs55921101 | T | A | -0.1092 | 0.0236 | 4.32E-06 |
| order | NB1n | rs9542068 | C | T | 0.0991 | 0.0218 | 6.52E-06 |
| order | NB1n | rs2930903 | G | C | 0.0908 | 0.0209 | 6.84E-06 |
| order | NB1n | rs60775321 | C | T | -0.0962 | 0.0214 | 7.10E-06 |
| order | NB1n | rs8126061 | C | T | -0.1591 | 0.0352 | 7.36E-06 |
| order | NB1n | rs166849 | A | G | 0.0911 | 0.0202 | 7.74E-06 |
| order | NB1n | rs80270230 | C | G | 0.1761 | 0.0393 | 8.41E-06 |
| order | Pasteurellales | rs12191680 | G | C | 0.1022 | 0.0197 | 3.05E-07 |
| order | Pasteurellales | rs9382510 | T | C | -0.0882 | 0.0170 | 2.48E-07 |
| order | Pasteurellales | rs10840326 | G | C | -0.0718 | 0.0149 | 1.44E-06 |
| order | Pasteurellales | rs76022354 | T | C | 0.2429 | 0.0500 | 1.83E-06 |
| order | Pasteurellales | rs72756943 | A | G | 0.1399 | 0.0303 | 3.35E-06 |
| order | Pasteurellales | rs78909003 | C | T | -0.2412 | 0.0498 | 2.05E-06 |
| order | Pasteurellales | rs6092684 | T | A | 0.0685 | 0.0147 | 3.44E-06 |
| order | Pasteurellales | rs4822728 | C | T | 0.0685 | 0.0149 | 4.72E-06 |
| order | Pasteurellales | rs35510 | G | A | 0.1227 | 0.0265 | 4.02E-06 |
| order | Pasteurellales | rs10965428 | A | C | -0.1199 | 0.0258 | 4.29E-06 |
| order | Pasteurellales | rs16970009 | G | A | 0.1874 | 0.0430 | 7.32E-06 |
| order | Pasteurellales | rs12050685 | G | A | -0.0671 | 0.0152 | 9.19E-06 |
| order | Pasteurellales | rs73139353 | C | A | -0.2226 | 0.0485 | 8.71E-06 |
| order | Pasteurellales | rs6972479 | G | A | -0.0782 | 0.0175 | 7.75E-06 |
| order | Pasteurellales | rs9895850 | C | T | -0.1764 | 0.0410 | 9.08E-06 |
| order | Pasteurellales | rs111582866 | A | G | -0.1140 | 0.0256 | 7.07E-06 |
| order | Pasteurellales | rs62568866 | A | T | -0.1180 | 0.0259 | 7.23E-06 |
| order | Pasteurellales | rs731534 | G | C | -0.0987 | 0.0216 | 9.46E-06 |
| order | Pasteurellales | rs9938097 | C | T | -0.0710 | 0.0158 | 8.23E-06 |
| order | Rhodospirillales | rs9813022 | G | A | -0.0831 | 0.0163 | 3.07E-07 |
| order | Rhodospirillales | rs11630875 | C | T | 0.0945 | 0.0202 | 3.70E-06 |
| order | Rhodospirillales | rs76784716 | G | A | 0.1360 | 0.0284 | 1.31E-06 |
| order | Rhodospirillales | rs3754624 | T | C | 0.0941 | 0.0198 | 2.68E-06 |
| order | Rhodospirillales | rs1549633 | C | A | 0.0997 | 0.0217 | 3.88E-06 |
| order | Rhodospirillales | rs7001029 | T | C | 0.1207 | 0.0261 | 2.83E-06 |
| order | Rhodospirillales | rs1035406 | A | G | -0.1147 | 0.0248 | 4.07E-06 |
| order | Rhodospirillales | rs4278423 | C | T | 0.1052 | 0.0234 | 3.98E-06 |
| order | Rhodospirillales | rs61933850 | A | G | 0.1647 | 0.0360 | 7.00E-06 |
| order | Rhodospirillales | rs77304857 | A | C | -0.0997 | 0.0222 | 6.02E-06 |
| order | Rhodospirillales | rs55876211 | T | C | -0.0865 | 0.0195 | 7.87E-06 |
| order | Rhodospirillales | rs11591293 | T | G | 0.0723 | 0.0158 | 4.69E-06 |
| order | Rhodospirillales | rs4822789 | C | G | 0.0728 | 0.0160 | 7.33E-06 |
| order | Rhodospirillales | rs13336560 | T | C | -0.0695 | 0.0157 | 9.75E-06 |
| order | Rhodospirillales | rs3730086 | G | A | 0.0800 | 0.0179 | 7.98E-06 |
| order | Selenomonadales | rs1643968 | C | T | -0.0565 | 0.0112 | 4.15E-07 |
| order | Selenomonadales | rs60274479 | C | T | -0.0660 | 0.0134 | 1.16E-06 |
| order | Selenomonadales | rs13086907 | A | G | 0.0625 | 0.0132 | 1.95E-06 |
| order | Selenomonadales | rs73232831 | A | G | -0.1518 | 0.0315 | 1.87E-06 |
| order | Selenomonadales | rs71405394 | A | G | -0.1142 | 0.0240 | 2.17E-06 |
| order | Selenomonadales | rs61249479 | C | A | 0.0777 | 0.0169 | 2.95E-06 |
| order | Selenomonadales | rs1447205 | C | G | -0.0502 | 0.0108 | 2.93E-06 |
| order | Selenomonadales | rs4722181 | G | T | 0.0501 | 0.0106 | 2.00E-06 |
| order | Selenomonadales | rs4463806 | C | T | -0.0544 | 0.0129 | 7.81E-06 |
| order | Selenomonadales | rs9423647 | A | G | 0.0478 | 0.0105 | 6.06E-06 |
| order | Selenomonadales | rs2834062 | G | A | 0.0489 | 0.0109 | 8.44E-06 |
| order | Selenomonadales | rs1649999 | G | A | 0.0749 | 0.0166 | 7.58E-06 |
| order | Selenomonadales | rs1135612 | A | G | 0.0530 | 0.0119 | 9.26E-06 |
| order | Verrucomicrobiales | rs11729256 | C | T | 0.0750 | 0.0150 | 6.73E-07 |
| order | Verrucomicrobiales | rs4936098 | G | A | 0.0649 | 0.0136 | 1.12E-06 |
| order | Verrucomicrobiales | rs9349825 | G | A | -0.0704 | 0.0147 | 2.54E-06 |
| order | Verrucomicrobiales | rs74542928 | C | T | 0.1122 | 0.0236 | 1.63E-06 |
| order | Verrucomicrobiales | rs3995795 | T | C | 0.0641 | 0.0141 | 5.19E-06 |
| order | Verrucomicrobiales | rs2602429 | T | C | 0.0747 | 0.0156 | 2.58E-06 |
| order | Verrucomicrobiales | rs4242783 | A | G | 0.0689 | 0.0148 | 2.64E-06 |
| order | Verrucomicrobiales | rs11184341 | C | G | 0.0655 | 0.0142 | 4.13E-06 |
| order | Verrucomicrobiales | rs61779207 | A | G | -0.0758 | 0.0168 | 6.72E-06 |
| order | Verrucomicrobiales | rs117107102 | G | A | 0.2047 | 0.0432 | 2.92E-06 |
| order | Verrucomicrobiales | rs12908520 | A | G | 0.0619 | 0.0131 | 2.17E-06 |
| order | Verrucomicrobiales | rs111862613 | C | T | 0.0907 | 0.0197 | 3.74E-06 |
| order | Verrucomicrobiales | rs941682 | A | G | -0.0631 | 0.0144 | 9.61E-06 |
| order | Victivallales | rs2825714 | G | A | -0.1374 | 0.0289 | 1.72E-06 |
| order | Victivallales | rs17114848 | A | G | 0.1524 | 0.0324 | 4.06E-06 |
| order | Victivallales | rs77599476 | G | A | 0.2303 | 0.0480 | 1.86E-06 |
| order | Victivallales | rs11770843 | T | C | 0.1094 | 0.0235 | 1.91E-06 |
| order | Victivallales | rs72640280 | G | A | 0.2202 | 0.0486 | 5.18E-06 |
| order | Victivallales | rs62570196 | T | C | -0.2164 | 0.0440 | 1.08E-06 |
| order | Victivallales | rs2731834 | G | C | 0.1094 | 0.0237 | 4.24E-06 |
| order | Victivallales | rs2031282 | G | A | 0.1224 | 0.0270 | 4.38E-06 |
| order | Victivallales | rs1002941 | A | G | 0.1050 | 0.0233 | 8.15E-06 |
| order | Victivallales | rs73113483 | A | T | -0.1312 | 0.0289 | 8.66E-06 |
| phylum | Actinobacteria | rs7570971 | C | A | 0.0867 | 0.0114 | 1.41E-14 |
| phylum | Actinobacteria | rs4429415 | T | C | 0.0582 | 0.0111 | 2.05E-07 |
| phylum | Actinobacteria | rs10841473 | C | G | -0.0597 | 0.0118 | 4.47E-07 |
| phylum | Actinobacteria | rs74037001 | A | G | -0.0819 | 0.0165 | 6.71E-07 |
| phylum | Actinobacteria | rs55888705 | G | A | 0.0534 | 0.0110 | 1.31E-06 |
| phylum | Actinobacteria | rs62448869 | A | T | -0.0525 | 0.0108 | 1.14E-06 |
| phylum | Actinobacteria | rs8047955 | G | A | 0.0521 | 0.0111 | 2.66E-06 |
| phylum | Actinobacteria | rs34284163 | T | A | -0.0603 | 0.0137 | 8.82E-06 |
| phylum | Actinobacteria | rs9833771 | C | T | 0.0490 | 0.0107 | 4.07E-06 |
| phylum | Actinobacteria | rs1397793 | A | G | -0.0521 | 0.0112 | 3.74E-06 |
| phylum | Actinobacteria | rs6496870 | C | T | 0.0511 | 0.0114 | 4.62E-06 |
| phylum | Actinobacteria | rs857444 | T | C | 0.0507 | 0.0110 | 3.80E-06 |
| phylum | Actinobacteria | rs12528285 | T | C | 0.0809 | 0.0181 | 5.69E-06 |
| phylum | Actinobacteria | rs80124826 | C | T | -0.1242 | 0.0279 | 8.75E-06 |
| phylum | Actinobacteria | rs13192624 | C | T | -0.0523 | 0.0118 | 9.33E-06 |
| phylum | Actinobacteria | rs75211493 | A | G | 0.0841 | 0.0185 | 9.27E-06 |
| phylum | Actinobacteria | rs6743026 | C | T | 0.0589 | 0.0135 | 9.88E-06 |
| phylum | Actinobacteria | rs11766971 | C | T | 0.0476 | 0.0106 | 9.40E-06 |
| phylum | Bacteroidetes | rs73846128 | G | A | -0.0664 | 0.0133 | 4.78E-07 |
| phylum | Bacteroidetes | rs73512608 | A | G | -0.1231 | 0.0237 | 2.54E-07 |
| phylum | Bacteroidetes | rs111845179 | C | T | 0.1042 | 0.0214 | 6.47E-07 |
| phylum | Bacteroidetes | rs73975615 | A | G | -0.2072 | 0.0443 | 1.20E-06 |
| phylum | Bacteroidetes | rs2032750 | C | T | -0.0511 | 0.0107 | 1.71E-06 |
| phylum | Bacteroidetes | rs7546249 | A | T | -0.0569 | 0.0118 | 1.42E-06 |
| phylum | Bacteroidetes | rs13291169 | G | C | 0.0701 | 0.0149 | 2.61E-06 |
| phylum | Bacteroidetes | rs17343978 | C | A | -0.0556 | 0.0120 | 7.22E-06 |
| phylum | Bacteroidetes | rs929878 | T | C | -0.0540 | 0.0122 | 6.51E-06 |
| phylum | Bacteroidetes | rs72706335 | C | T | -0.2232 | 0.0493 | 7.13E-06 |
| phylum | Bacteroidetes | rs7999780 | A | G | 0.0544 | 0.0124 | 9.47E-06 |
| phylum | Bacteroidetes | rs6586324 | C | T | 0.0478 | 0.0105 | 7.37E-06 |
| phylum | Bacteroidetes | rs62531359 | G | T | 0.0658 | 0.0150 | 8.42E-06 |
| phylum | Bacteroidetes | rs62575403 | T | C | 0.1454 | 0.0311 | 2.96E-06 |
| phylum | Cyanobacteria | rs789068 | A | G | -0.1114 | 0.0213 | 1.57E-07 |
| phylum | Cyanobacteria | rs9864379 | C | T | -0.1389 | 0.0268 | 2.03E-07 |
| phylum | Cyanobacteria | rs76531781 | C | T | -0.2319 | 0.0493 | 2.87E-06 |
| phylum | Cyanobacteria | rs7148504 | T | G | 0.0800 | 0.0177 | 6.62E-06 |
| phylum | Cyanobacteria | rs584122 | T | C | -0.1518 | 0.0327 | 4.23E-06 |
| phylum | Cyanobacteria | rs2314810 | G | C | -0.2183 | 0.0465 | 4.25E-06 |
| phylum | Cyanobacteria | rs2553290 | T | A | 0.0962 | 0.0203 | 3.61E-06 |
| phylum | Cyanobacteria | rs12555298 | A | G | 0.0974 | 0.0219 | 8.09E-06 |
| phylum | Cyanobacteria | rs2585223 | C | T | 0.1114 | 0.0248 | 8.86E-06 |
| phylum | Cyanobacteria | rs61972390 | C | T | 0.1073 | 0.0242 | 9.11E-06 |
| phylum | Euryarchaeota | rs10202904 | G | T | -0.1160 | 0.0230 | 6.19E-07 |
| phylum | Euryarchaeota | rs76029318 | C | T | 0.2149 | 0.0438 | 1.05E-06 |
| phylum | Euryarchaeota | rs73031978 | G | C | -0.1975 | 0.0414 | 2.74E-06 |
| phylum | Euryarchaeota | rs34928225 | C | T | 0.1998 | 0.0425 | 4.33E-06 |
| phylum | Euryarchaeota | rs7635189 | A | G | 0.1200 | 0.0259 | 4.64E-06 |
| phylum | Euryarchaeota | rs77658038 | C | A | -0.1602 | 0.0341 | 4.75E-06 |
| phylum | Euryarchaeota | rs894996 | A | C | 0.2036 | 0.0440 | 5.12E-06 |
| phylum | Euryarchaeota | rs56131665 | A | G | 0.1768 | 0.0386 | 5.30E-06 |
| phylum | Euryarchaeota | rs45498998 | A | G | -0.1319 | 0.0292 | 5.32E-06 |
| phylum | Euryarchaeota | rs7015093 | A | G | -0.1181 | 0.0264 | 7.20E-06 |
| phylum | Euryarchaeota | rs11022995 | A | G | -0.1037 | 0.0229 | 7.73E-06 |
| phylum | Euryarchaeota | rs6508769 | C | T | 0.1505 | 0.0337 | 8.12E-06 |
| phylum | Euryarchaeota | rs6064552 | C | T | -0.1236 | 0.0276 | 9.34E-06 |
| phylum | Firmicutes | rs3792064 | A | G | 0.0897 | 0.0183 | 6.75E-07 |
| phylum | Firmicutes | rs2009919 | C | T | 0.0536 | 0.0106 | 4.95E-07 |
| phylum | Firmicutes | rs112334273 | A | G | 0.0627 | 0.0127 | 9.26E-07 |
| phylum | Firmicutes | rs13421739 | G | C | 0.0944 | 0.0180 | 1.95E-06 |
| phylum | Firmicutes | rs7975768 | G | C | 0.0513 | 0.0109 | 2.82E-06 |
| phylum | Firmicutes | rs2332027 | A | G | -0.0483 | 0.0105 | 4.05E-06 |
| phylum | Firmicutes | rs2273429 | G | A | -0.0702 | 0.0153 | 9.26E-06 |
| phylum | Firmicutes | rs7247191 | C | T | -0.0713 | 0.0157 | 4.73E-06 |
| phylum | Firmicutes | rs72771021 | T | C | -0.1414 | 0.0308 | 5.12E-06 |
| phylum | Firmicutes | rs3852931 | T | C | -0.0482 | 0.0105 | 4.53E-06 |
| phylum | Firmicutes | rs2547978 | G | A | 0.0466 | 0.0105 | 8.57E-06 |
| phylum | Firmicutes | rs36111799 | T | C | -0.0481 | 0.0105 | 5.17E-06 |
| phylum | Firmicutes | rs12554342 | C | G | -0.0526 | 0.0117 | 6.81E-06 |
| phylum | Firmicutes | rs72738886 | C | T | 0.0865 | 0.0190 | 7.68E-06 |
| phylum | Firmicutes | rs4750583 | G | A | 0.0616 | 0.0138 | 5.79E-06 |
| phylum | Firmicutes | rs6815608 | C | T | 0.0936 | 0.0211 | 7.24E-06 |
| phylum | Firmicutes | rs8085381 | A | G | 0.0647 | 0.0146 | 8.67E-06 |
| phylum | Firmicutes | rs56199908 | C | T | -0.1862 | 0.0411 | 8.67E-06 |
| phylum | Firmicutes | rs7573799 | G | C | -0.0698 | 0.0154 | 8.05E-06 |
| phylum | Firmicutes | rs6814436 | C | T | 0.0680 | 0.0151 | 6.80E-06 |
| phylum | Lentisphaerae | rs2825714 | G | A | -0.1382 | 0.0289 | 1.50E-06 |
| phylum | Lentisphaerae | rs11770843 | T | C | 0.1119 | 0.0235 | 1.14E-06 |
| phylum | Lentisphaerae | rs77599476 | G | A | 0.2300 | 0.0480 | 1.90E-06 |
| phylum | Lentisphaerae | rs62570196 | T | C | -0.2172 | 0.0440 | 9.64E-07 |
| phylum | Lentisphaerae | rs72640280 | G | A | 0.2202 | 0.0486 | 5.19E-06 |
| phylum | Lentisphaerae | rs17114848 | A | G | 0.1490 | 0.0324 | 6.77E-06 |
| phylum | Lentisphaerae | rs1002941 | A | G | 0.1077 | 0.0233 | 4.31E-06 |
| phylum | Lentisphaerae | rs2731834 | G | C | 0.1099 | 0.0237 | 3.80E-06 |
| phylum | Lentisphaerae | rs60995569 | G | T | -0.1606 | 0.0337 | 9.19E-06 |
| phylum | Lentisphaerae | rs2031282 | G | A | 0.1204 | 0.0270 | 5.86E-06 |
| phylum | Lentisphaerae | rs73113483 | A | T | -0.1317 | 0.0289 | 8.79E-06 |
| phylum | Proteobacteria | rs922773 | T | C | -0.0804 | 0.0158 | 3.68E-07 |
| phylum | Proteobacteria | rs2532663 | A | G | -0.1256 | 0.0258 | 7.47E-07 |
| phylum | Proteobacteria | rs12150865 | T | C | 0.0512 | 0.0106 | 1.54E-06 |
| phylum | Proteobacteria | rs2347697 | T | G | 0.0502 | 0.0109 | 4.27E-06 |
| phylum | Proteobacteria | rs312757 | G | C | -0.0517 | 0.0113 | 4.84E-06 |
| phylum | Proteobacteria | rs12467198 | T | C | 0.0498 | 0.0111 | 6.31E-06 |
| phylum | Proteobacteria | rs10750258 | C | A | -0.0491 | 0.0108 | 8.72E-06 |
| phylum | Proteobacteria | rs6707783 | T | C | 0.0850 | 0.0188 | 8.09E-06 |
| phylum | Proteobacteria | rs72771021 | T | C | 0.1417 | 0.0309 | 7.18E-06 |
| phylum | Proteobacteria | rs3890996 | G | T | -0.0475 | 0.0106 | 6.95E-06 |
| phylum | Proteobacteria | rs74757828 | T | A | 0.0951 | 0.0217 | 9.25E-06 |
| phylum | Proteobacteria | rs4340090 | T | C | -0.0668 | 0.0153 | 9.99E-06 |
| phylum | Proteobacteria | rs11715072 | A | G | -0.0519 | 0.0115 | 6.90E-06 |
| phylum | Proteobacteria | rs11126162 | C | T | -0.0771 | 0.0187 | 9.26E-06 |
| phylum | Tenericutes | rs74603314 | C | T | 0.2216 | 0.0463 | 1.56E-06 |
| phylum | Tenericutes | rs10108398 | A | G | 0.0769 | 0.0154 | 1.09E-06 |
| phylum | Tenericutes | rs11890098 | G | A | 0.0744 | 0.0153 | 9.57E-07 |
| phylum | Tenericutes | rs72901605 | C | T | -0.0842 | 0.0178 | 3.26E-06 |
| phylum | Tenericutes | rs3768491 | G | A | -0.0681 | 0.0149 | 4.23E-06 |
| phylum | Tenericutes | rs17214486 | A | C | 0.0610 | 0.0136 | 6.61E-06 |
| phylum | Tenericutes | rs12566890 | G | T | -0.1011 | 0.0231 | 3.65E-06 |
| phylum | Tenericutes | rs78169027 | G | A | -0.1083 | 0.0237 | 5.88E-06 |
| phylum | Tenericutes | rs4885016 | C | T | -0.0820 | 0.0182 | 7.27E-06 |
| phylum | Tenericutes | rs28537087 | A | G | 0.0821 | 0.0188 | 8.07E-06 |
| phylum | Tenericutes | rs2464826 | C | A | 0.0944 | 0.0212 | 8.40E-06 |
| phylum | Tenericutes | rs6043847 | C | T | -0.1149 | 0.0249 | 4.55E-06 |
| phylum | Verrucomicrobia | rs74542928 | C | T | 0.1160 | 0.0231 | 4.08E-07 |
| phylum | Verrucomicrobia | rs11252894 | C | A | 0.0784 | 0.0163 | 1.11E-06 |
| phylum | Verrucomicrobia | rs2602429 | T | C | 0.0764 | 0.0153 | 8.71E-07 |
| phylum | Verrucomicrobia | rs11729256 | C | T | 0.0697 | 0.0147 | 2.23E-06 |
| phylum | Verrucomicrobia | rs45598138 | A | C | -0.1439 | 0.0305 | 2.19E-06 |
| phylum | Verrucomicrobia | rs61779207 | A | G | -0.0755 | 0.0164 | 5.28E-06 |
| phylum | Verrucomicrobia | rs76430504 | C | T | -0.1175 | 0.0255 | 3.50E-06 |
| phylum | Verrucomicrobia | rs9349825 | G | A | -0.0660 | 0.0144 | 6.27E-06 |
| phylum | Verrucomicrobia | rs117107102 | G | A | 0.2043 | 0.0428 | 2.68E-06 |
| phylum | Verrucomicrobia | rs3995795 | T | C | 0.0611 | 0.0139 | 9.72E-06 |
| phylum | Verrucomicrobia | rs12908520 | A | G | 0.0595 | 0.0128 | 3.40E-06 |
| phylum | Verrucomicrobia | rs12512971 | C | A | 0.1711 | 0.0398 | 9.81E-06 |
|  |  |  |  |  |  |  |  |

SNP, single nucleotide polymorphism

**Table S2** MR results of causal links between gut microbiome and PTSD in Freeze 2 datasets in MR analysis

| **outcome** | **Classification** | **Traits** | **method** | **b** | **se** | **pval** | **estimate** |
| --- | --- | --- | --- | --- | --- | --- | --- |
| PTSD | class | Actinobacteria | MR Egger | -0.3012 | 0.2059 | 0.1656 | 0.74(0.49-1.11) |
| PTSD | class | Actinobacteria | Weighted median | -0.1040 | 0.0907 | 0.2516 | 0.90(0.75-1.08) |
| PTSD | class | Actinobacteria | IVW | -0.0201 | 0.0674 | 0.7659 | 0.98(0.86-1.12) |
| PTSD | class | Actinobacteria | Simple mode | -0.1063 | 0.1612 | 0.5199 | 0.90(0.66-1.23) |
| PTSD | class | Actinobacteria | Weighted mode | -0.1307 | 0.1394 | 0.3631 | 0.88(0.67-1.15) |
| PTSD | class | Alphaproteobacteria | MR Egger | -0.4215 | 0.3238 | 0.2497 | 0.66(0.35-1.24) |
| PTSD | class | Alphaproteobacteria | Weighted median | 0.0626 | 0.1093 | 0.5670 | 1.06(0.86-1.32) |
| PTSD | class | Alphaproteobacteria | IVW | 0.0136 | 0.0852 | 0.8727 | 1.01(0.86-1.20) |
| PTSD | class | Alphaproteobacteria | Simple mode | 0.1093 | 0.1580 | 0.5149 | 1.12(0.82-1.52) |
| PTSD | class | Alphaproteobacteria | Weighted mode | 0.0991 | 0.1547 | 0.5453 | 1.10(0.82-1.50) |
| PTSD | class | Bacilli | MR Egger | 0.1084 | 0.1829 | 0.5619 | 1.11(0.78-1.60) |
| PTSD | class | Bacilli | Weighted median | 0.1047 | 0.0964 | 0.2770 | 1.11(0.92-1.34) |
| PTSD | class | Bacilli | IVW | 0.0648 | 0.0676 | 0.3372 | 1.07(0.93-1.22) |
| PTSD | class | Bacilli | Simple mode | 0.2014 | 0.1648 | 0.2384 | 1.22(0.89-1.69) |
| PTSD | class | Bacilli | Weighted mode | 0.1733 | 0.1424 | 0.2404 | 1.19(0.90-1.57) |
| PTSD | class | Bacteroidia | MR Egger | -0.0502 | 0.1701 | 0.7736 | 0.95(0.68-1.33) |
| PTSD | class | Bacteroidia | Weighted median | -0.0117 | 0.1083 | 0.9140 | 0.99(0.80-1.22) |
| PTSD | class | Bacteroidia | IVW | -0.0406 | 0.0779 | 0.6024 | 0.96(0.82-1.12) |
| PTSD | class | Bacteroidia | Simple mode | 0.1425 | 0.1873 | 0.4615 | 1.15(0.80-1.66) |
| PTSD | class | Bacteroidia | Weighted mode | 0.0968 | 0.1384 | 0.4976 | 1.10(0.84-1.44) |
| PTSD | class | Betaproteobacteria | MR Egger | -0.3069 | 0.3091 | 0.3442 | 0.74(0.40-1.35) |
| PTSD | class | Betaproteobacteria | Weighted median | 0.0516 | 0.1172 | 0.6596 | 1.05(0.84-1.32) |
| PTSD | class | Betaproteobacteria | IVW | 0.0751 | 0.0854 | 0.3789 | 1.08(0.91-1.27) |
| PTSD | class | Betaproteobacteria | Simple mode | -0.1293 | 0.2141 | 0.5581 | 0.88(0.58-1.34) |
| PTSD | class | Betaproteobacteria | Weighted mode | -0.1187 | 0.2189 | 0.5983 | 0.89(0.58-1.36) |
| PTSD | class | Clostridia | MR Egger | -0.0921 | 0.4773 | 0.8509 | 0.91(0.36-2.32) |
| PTSD | class | Clostridia | Weighted median | -0.1776 | 0.1165 | 0.1273 | 0.84(0.67-1.05) |
| PTSD | class | Clostridia | IVW | -0.1691 | 0.0900 | 0.0604 | 0.84(0.71-1.01) |
| PTSD | class | Clostridia | Simple mode | -0.1927 | 0.1982 | 0.3519 | 0.82(0.56-1.22) |
| PTSD | class | Clostridia | Weighted mode | -0.1863 | 0.1905 | 0.3492 | 0.83(0.57-1.21) |
| PTSD | class | Coriobacteriia | MR Egger | -0.3376 | 0.3237 | 0.3125 | 0.71(0.38-1.35) |
| PTSD | class | Coriobacteriia | Weighted median | 0.0324 | 0.1040 | 0.7552 | 1.03(0.84-1.27) |
| PTSD | class | Coriobacteriia | IVW | 0.0484 | 0.0802 | 0.5467 | 1.05(0.90-1.23) |
| PTSD | class | Coriobacteriia | Simple mode | -0.0261 | 0.1907 | 0.8927 | 0.97(0.67-1.42) |
| PTSD | class | Coriobacteriia | Weighted mode | -0.0261 | 0.1813 | 0.8872 | 0.97(0.68-1.39) |
| PTSD | class | Deltaproteobacteria | MR Egger | 0.0366 | 0.2092 | 0.8642 | 1.04(0.69-1.56) |
| PTSD | class | Deltaproteobacteria | Weighted median | 0.0296 | 0.0974 | 0.7609 | 1.03(0.85-1.25) |
| PTSD | class | Deltaproteobacteria | IVW | 0.0265 | 0.0739 | 0.7203 | 1.03(0.89-1.19) |
| PTSD | class | Deltaproteobacteria | Simple mode | 0.0060 | 0.1383 | 0.9659 | 1.01(0.77-1.32) |
| PTSD | class | Deltaproteobacteria | Weighted mode | 0.0242 | 0.1310 | 0.8563 | 1.02(0.79-1.32) |
| PTSD | class | Erysipelotrichia | MR Egger | 0.2322 | 0.3705 | 0.5437 | 1.26(0.61-2.61) |
| PTSD | class | Erysipelotrichia | Weighted median | -0.0190 | 0.1161 | 0.8701 | 0.98(0.78-1.23) |
| PTSD | class | Erysipelotrichia | IVW | -0.0054 | 0.0849 | 0.9494 | 0.99(0.84-1.17) |
| PTSD | class | Erysipelotrichia | Simple mode | 0.0108 | 0.2045 | 0.9586 | 1.01(0.68-1.51) |
| PTSD | class | Erysipelotrichia | Weighted mode | 0.0197 | 0.1988 | 0.9227 | 1.02(0.69-1.51) |
| PTSD | class | Gammaproteobacteria | MR Egger | 0.5704 | 0.4454 | 0.2566 | 1.77(0.74-4.24) |
| PTSD | class | Gammaproteobacteria | Weighted median | 0.0488 | 0.1490 | 0.7431 | 1.05(0.78-1.41) |
| PTSD | class | Gammaproteobacteria | IVW | 0.0694 | 0.1341 | 0.6046 | 1.07(0.82-1.39) |
| PTSD | class | Gammaproteobacteria | Simple mode | 0.3107 | 0.2696 | 0.2929 | 1.36(0.80-2.31) |
| PTSD | class | Gammaproteobacteria | Weighted mode | 0.3393 | 0.2934 | 0.2914 | 1.40(0.79-2.49) |
| PTSD | class | Lentisphaeria | MR Egger | 0.0833 | 0.2179 | 0.7155 | 1.09(0.71-1.67) |
| PTSD | class | Lentisphaeria | Weighted median | -0.0309 | 0.0742 | 0.6773 | 0.97(0.84-1.12) |
| PTSD | class | Lentisphaeria | IVW | -0.0617 | 0.0601 | 0.3047 | 0.94(0.84-1.06) |
| PTSD | class | Lentisphaeria | Simple mode | -0.0430 | 0.1282 | 0.7473 | 0.96(0.75-1.23) |
| PTSD | class | Lentisphaeria | Weighted mode | -0.0277 | 0.1163 | 0.8184 | 0.97(0.77-1.22) |
| PTSD | class | Melainabacteria | MR Egger | -0.0439 | 0.1509 | 0.7784 | 0.96(0.71-1.29) |
| PTSD | class | Melainabacteria | Weighted median | -0.0135 | 0.0697 | 0.8459 | 0.99(0.86-1.13) |
| PTSD | class | Melainabacteria | IVW | -0.0062 | 0.0532 | 0.9073 | 0.99(0.90-1.10) |
| PTSD | class | Melainabacteria | Simple mode | -0.0263 | 0.1137 | 0.8225 | 0.97(0.78-1.22) |
| PTSD | class | Melainabacteria | Weighted mode | -0.0298 | 0.1289 | 0.8223 | 0.97(0.75-1.25) |
| PTSD | class | Methanobacteria | MR Egger | 0.0287 | 0.1777 | 0.8758 | 1.03(0.73-1.46) |
| PTSD | class | Methanobacteria | Weighted median | -0.0120 | 0.0536 | 0.8227 | 0.99(0.89-1.10) |
| PTSD | class | Methanobacteria | IVW | -0.0224 | 0.0423 | 0.5964 | 0.98(0.90-1.06) |
| PTSD | class | Methanobacteria | Simple mode | -0.0155 | 0.0917 | 0.8694 | 0.98(0.82-1.18) |
| PTSD | class | Methanobacteria | Weighted mode | -0.0117 | 0.0849 | 0.8938 | 0.99(0.84-1.17) |
| PTSD | class | Mollicutes | MR Egger | 0.1802 | 0.2165 | 0.4248 | 1.20(0.78-1.83) |
| PTSD | class | Mollicutes | Weighted median | 0.0842 | 0.0905 | 0.3522 | 1.09(0.91-1.30) |
| PTSD | class | Mollicutes | IVW | -0.0177 | 0.0669 | 0.7907 | 0.98(0.86-1.12) |
| PTSD | class | Mollicutes | Simple mode | 0.1318 | 0.1628 | 0.4356 | 1.14(0.83-1.57) |
| PTSD | class | Mollicutes | Weighted mode | 0.1106 | 0.1433 | 0.4565 | 1.12(0.84-1.48) |
| PTSD | class | Negativicutes | MR Egger | 0.0108 | 0.2793 | 0.9700 | 1.01(0.58-1.75) |
| PTSD | class | Negativicutes | Weighted median | -0.0518 | 0.1159 | 0.6548 | 0.95(0.76-1.19) |
| PTSD | class | Negativicutes | IVW | -0.0600 | 0.0858 | 0.4846 | 0.94(0.80-1.11) |
| PTSD | class | Negativicutes | Simple mode | -0.0205 | 0.1915 | 0.9168 | 0.98(0.67-1.43) |
| PTSD | class | Negativicutes | Weighted mode | -0.0515 | 0.1772 | 0.7766 | 0.95(0.67-1.34) |
| PTSD | class | Verrucomicrobiae | MR Egger | 0.1818 | 0.2580 | 0.4972 | 1.20(0.72-1.99) |
| PTSD | class | Verrucomicrobiae | Weighted median | 0.1165 | 0.1033 | 0.2592 | 1.12(0.92-1.38) |
| PTSD | class | Verrucomicrobiae | IVW | 0.0826 | 0.0707 | 0.2430 | 1.09(0.95-1.25) |
| PTSD | class | Verrucomicrobiae | Simple mode | 0.2243 | 0.1894 | 0.2614 | 1.25(0.86-1.81) |
| PTSD | class | Verrucomicrobiae | Weighted mode | 0.2509 | 0.1812 | 0.1937 | 1.29(0.90-1.83) |
| PTSD | family | Acidaminococcaceae | MR Egger | 0.1599 | 0.2789 | 0.5912 | 1.17(0.68-2.03) |
| PTSD | family | Acidaminococcaceae | Weighted median | 0.1087 | 0.1183 | 0.3583 | 1.11(0.88-1.41) |
| PTSD | family | Acidaminococcaceae | IVW | 0.0433 | 0.0922 | 0.6384 | 1.04(0.87-1.25) |
| PTSD | family | Acidaminococcaceae | Simple mode | 0.1354 | 0.1649 | 0.4428 | 1.15(0.83-1.58) |
| PTSD | family | Acidaminococcaceae | Weighted mode | 0.1421 | 0.1664 | 0.4259 | 1.15(0.83-1.60) |
| PTSD | family | Actinomycetaceae | MR Egger | 0.0411 | 0.2323 | 0.8707 | 1.04(0.66-1.64) |
| PTSD | family | Actinomycetaceae | Weighted median | 0.0230 | 0.1123 | 0.8377 | 1.02(0.82-1.28) |
| PTSD | family | Actinomycetaceae | IVW | 0.0202 | 0.0838 | 0.8098 | 1.02(0.87-1.20) |
| PTSD | family | Actinomycetaceae | Simple mode | 0.0325 | 0.1544 | 0.8437 | 1.03(0.76-1.40) |
| PTSD | family | Actinomycetaceae | Weighted mode | 0.0242 | 0.1411 | 0.8723 | 1.02(0.78-1.35) |
| PTSD | family | Alcaligenaceae | MR Egger | 0.3402 | 0.3996 | 0.4113 | 1.41(0.64-3.08) |
| PTSD | family | Alcaligenaceae | Weighted median | 0.0172 | 0.1115 | 0.8773 | 1.02(0.82-1.27) |
| PTSD | family | Alcaligenaceae | IVW | 0.0880 | 0.0858 | 0.3050 | 1.09(0.92-1.29) |
| PTSD | family | Alcaligenaceae | Simple mode | -0.0448 | 0.1987 | 0.8251 | 0.96(0.65-1.41) |
| PTSD | family | Alcaligenaceae | Weighted mode | -0.0448 | 0.2062 | 0.8313 | 0.96(0.64-1.43) |
| PTSD | family | Bacteroidaceae | MR Egger | 0.2136 | 0.6319 | 0.7468 | 1.24(0.36-4.27) |
| PTSD | family | Bacteroidaceae | Weighted median | 0.1530 | 0.1471 | 0.2984 | 1.17(0.87-1.55) |
| PTSD | family | Bacteroidaceae | IVW | 0.0684 | 0.1084 | 0.5284 | 1.07(0.87-1.32) |
| PTSD | family | Bacteroidaceae | Simple mode | 0.2550 | 0.2499 | 0.3415 | 1.29(0.79-2.11) |
| PTSD | family | Bacteroidaceae | Weighted mode | 0.2705 | 0.2206 | 0.2598 | 1.31(0.85-2.02) |
| PTSD | family | BacteroidalesS24 | MR Egger | -0.0287 | 0.2939 | 0.9250 | 0.97(0.55-1.73) |
| PTSD | family | BacteroidalesS24 | Weighted median | -0.0148 | 0.0810 | 0.8546 | 0.99(0.84-1.15) |
| PTSD | family | BacteroidalesS24 | IVW | 0.0033 | 0.0653 | 0.9598 | 1.00(0.88-1.14) |
| PTSD | family | BacteroidalesS24 | Simple mode | -0.0388 | 0.1186 | 0.7523 | 0.96(0.76-1.21) |
| PTSD | family | BacteroidalesS24 | Weighted mode | -0.0388 | 0.1268 | 0.7677 | 0.96(0.75-1.23) |
| PTSD | family | Bifidobacteriaceae | MR Egger | -0.0159 | 0.2868 | 0.9566 | 0.98(0.56-1.73) |
| PTSD | family | Bifidobacteriaceae | Weighted median | -0.1320 | 0.1007 | 0.1897 | 0.88(0.72-1.07) |
| PTSD | family | Bifidobacteriaceae | IVW | -0.0589 | 0.0745 | 0.4291 | 0.94(0.81-1.09) |
| PTSD | family | Bifidobacteriaceae | Simple mode | 0.2168 | 0.2078 | 0.3144 | 1.24(0.83-1.87) |
| PTSD | family | Bifidobacteriaceae | Weighted mode | -0.2179 | 0.1329 | 0.1235 | 0.80(0.62-1.04) |
| PTSD | family | Christensenellaceae | MR Egger | -0.0419 | 0.1662 | 0.8068 | 0.96(0.69-1.33) |
| PTSD | family | Christensenellaceae | Weighted median | 0.0192 | 0.1032 | 0.8523 | 1.02(0.83-1.25) |
| PTSD | family | Christensenellaceae | IVW | 0.0081 | 0.0780 | 0.9169 | 1.01(0.87-1.17) |
| PTSD | family | Christensenellaceae | Simple mode | 0.0081 | 0.1555 | 0.9597 | 1.01(0.74-1.37) |
| PTSD | family | Christensenellaceae | Weighted mode | 0.0254 | 0.1426 | 0.8623 | 1.03(0.78-1.36) |
| PTSD | family | Clostridiaceae1 | MR Egger | -0.1746 | 0.2442 | 0.4950 | 0.84(0.52-1.36) |
| PTSD | family | Clostridiaceae1 | Weighted median | -0.0539 | 0.1120 | 0.6303 | 0.95(0.76-1.18) |
| PTSD | family | Clostridiaceae1 | IVW | -0.0552 | 0.0834 | 0.5082 | 0.95(0.80-1.11) |
| PTSD | family | Clostridiaceae1 | Simple mode | -0.0909 | 0.1877 | 0.6397 | 0.91(0.63-1.32) |
| PTSD | family | Clostridiaceae1 | Weighted mode | -0.0703 | 0.1699 | 0.6887 | 0.93(0.67-1.30) |
| PTSD | family | ClostridialesvadinBB60group | MR Egger | 0.1272 | 0.1656 | 0.4560 | 1.14(0.82-1.57) |
| PTSD | family | ClostridialesvadinBB60group | Weighted median | 0.0357 | 0.0804 | 0.6569 | 1.04(0.89-1.21) |
| PTSD | family | ClostridialesvadinBB60group | IVW | -0.0068 | 0.0577 | 0.9059 | 0.99(0.89-1.11) |
| PTSD | family | ClostridialesvadinBB60group | Simple mode | 0.0945 | 0.1344 | 0.4934 | 1.10(0.84-1.43) |
| PTSD | family | ClostridialesvadinBB60group | Weighted mode | 0.0770 | 0.1308 | 0.5651 | 1.08(0.84-1.40) |
| PTSD | family | Coriobacteriaceae | MR Egger | -0.3376 | 0.3237 | 0.3125 | 0.71(0.38-1.35) |
| PTSD | family | Coriobacteriaceae | Weighted median | 0.0324 | 0.0994 | 0.7441 | 1.03(0.85-1.26) |
| PTSD | family | Coriobacteriaceae | IVW | 0.0484 | 0.0802 | 0.5467 | 1.05(0.90-1.23) |
| PTSD | family | Coriobacteriaceae | Simple mode | -0.0261 | 0.1798 | 0.8862 | 0.97(0.68-1.39) |
| PTSD | family | Coriobacteriaceae | Weighted mode | -0.0261 | 0.1769 | 0.8844 | 0.97(0.69-1.38) |
| PTSD | family | Defluviitaleaceae | MR Egger | 0.1167 | 0.2057 | 0.5845 | 1.12(0.75-1.68) |
| PTSD | family | Defluviitaleaceae | Weighted median | 0.0858 | 0.0819 | 0.2949 | 1.09(0.93-1.28) |
| PTSD | family | Defluviitaleaceae | IVW | 0.0812 | 0.0620 | 0.1903 | 1.08(0.96-1.22) |
| PTSD | family | Defluviitaleaceae | Simple mode | 0.0642 | 0.1292 | 0.6298 | 1.07(0.83-1.37) |
| PTSD | family | Defluviitaleaceae | Weighted mode | 0.0642 | 0.1216 | 0.6087 | 1.07(0.84-1.35) |
| PTSD | family | Desulfovibrionaceae | MR Egger | 0.0187 | 0.2082 | 0.9305 | 1.02(0.68-1.53) |
| PTSD | family | Desulfovibrionaceae | Weighted median | 0.0304 | 0.1058 | 0.7740 | 1.03(0.84-1.27) |
| PTSD | family | Desulfovibrionaceae | IVW | 0.0399 | 0.0820 | 0.6270 | 1.04(0.89-1.22) |
| PTSD | family | Desulfovibrionaceae | Simple mode | 0.0311 | 0.1622 | 0.8520 | 1.03(0.75-1.42) |
| PTSD | family | Desulfovibrionaceae | Weighted mode | 0.0386 | 0.1365 | 0.7836 | 1.04(0.80-1.36) |
| PTSD | family | Enterobacteriaceae | MR Egger | 0.3596 | 0.6915 | 0.6252 | 1.43(0.37-5.56) |
| PTSD | family | Enterobacteriaceae | Weighted median | 0.1940 | 0.1444 | 0.1791 | 1.21(0.91-1.61) |
| PTSD | family | Enterobacteriaceae | IVW | 0.0626 | 0.1079 | 0.5618 | 1.06(0.86-1.32) |
| PTSD | family | Enterobacteriaceae | Simple mode | 0.2373 | 0.2333 | 0.3484 | 1.27(0.80-2.00) |
| PTSD | family | Enterobacteriaceae | Weighted mode | 0.2408 | 0.2246 | 0.3250 | 1.27(0.82-1.98) |
| PTSD | family | Erysipelotrichaceae | MR Egger | 0.2322 | 0.3705 | 0.5437 | 1.26(0.61-2.61) |
| PTSD | family | Erysipelotrichaceae | Weighted median | -0.0190 | 0.1127 | 0.8662 | 0.98(0.79-1.22) |
| PTSD | family | Erysipelotrichaceae | IVW | -0.0054 | 0.0849 | 0.9494 | 0.99(0.84-1.17) |
| PTSD | family | Erysipelotrichaceae | Simple mode | 0.0108 | 0.1937 | 0.9562 | 1.01(0.69-1.48) |
| PTSD | family | Erysipelotrichaceae | Weighted mode | 0.0197 | 0.2062 | 0.9255 | 1.02(0.68-1.53) |
| PTSD | family | FamilyXI | MR Egger | -0.2087 | 0.3124 | 0.5289 | 0.81(0.44-1.50) |
| PTSD | family | FamilyXI | Weighted median | -0.0225 | 0.0583 | 0.6994 | 0.98(0.87-1.10) |
| PTSD | family | FamilyXI | IVW | 0.0214 | 0.0470 | 0.6489 | 1.02(0.93-1.12) |
| PTSD | family | FamilyXI | Simple mode | -0.0711 | 0.0996 | 0.4985 | 0.93(0.77-1.13) |
| PTSD | family | FamilyXI | Weighted mode | -0.0678 | 0.0877 | 0.4648 | 0.93(0.79-1.11) |
| PTSD | family | FamilyXIII | MR Egger | -0.0321 | 0.2933 | 0.9156 | 0.97(0.54-1.72) |
| PTSD | family | FamilyXIII | Weighted median | -0.1343 | 0.1168 | 0.2505 | 0.87(0.70-1.10) |
| PTSD | family | FamilyXIII | IVW | -0.1335 | 0.0876 | 0.1274 | 0.88(0.74-1.04) |
| PTSD | family | FamilyXIII | Simple mode | -0.0898 | 0.1769 | 0.6237 | 0.91(0.65-1.29) |
| PTSD | family | FamilyXIII | Weighted mode | -0.1102 | 0.1795 | 0.5543 | 0.90(0.63-1.27) |
| PTSD | family | Lachnospiraceae | MR Egger | 0.1840 | 0.1719 | 0.3014 | 1.20(0.86-1.68) |
| PTSD | family | Lachnospiraceae | Weighted median | 0.1320 | 0.0903 | 0.1439 | 1.14(0.96-1.36) |
| PTSD | family | Lachnospiraceae | IVW | 0.0853 | 0.0690 | 0.2167 | 1.09(0.95-1.25) |
| PTSD | family | Lachnospiraceae | Simple mode | 0.1503 | 0.1401 | 0.2994 | 1.16(0.88-1.53) |
| PTSD | family | Lachnospiraceae | Weighted mode | 0.1563 | 0.1303 | 0.2476 | 1.17(0.91-1.51) |
| PTSD | family | Lactobacillaceae | MR Egger | 0.0197 | 0.1792 | 0.9151 | 1.02(0.72-1.45) |
| PTSD | family | Lactobacillaceae | Weighted median | 0.0378 | 0.0796 | 0.6351 | 1.04(0.89-1.21) |
| PTSD | family | Lactobacillaceae | IVW | 0.0464 | 0.0625 | 0.4581 | 1.05(0.93-1.18) |
| PTSD | family | Lactobacillaceae | Simple mode | 0.0335 | 0.1320 | 0.8057 | 1.03(0.80-1.34) |
| PTSD | family | Lactobacillaceae | Weighted mode | 0.0360 | 0.1300 | 0.7881 | 1.04(0.80-1.34) |
| PTSD | family | Methanobacteriaceae | MR Egger | 0.0287 | 0.1777 | 0.8758 | 1.03(0.73-1.46) |
| PTSD | family | Methanobacteriaceae | Weighted median | -0.0120 | 0.0540 | 0.8240 | 0.99(0.89-1.10) |
| PTSD | family | Methanobacteriaceae | IVW | -0.0224 | 0.0423 | 0.5964 | 0.98(0.90-1.06) |
| PTSD | family | Methanobacteriaceae | Simple mode | -0.0155 | 0.0910 | 0.8683 | 0.98(0.82-1.18) |
| PTSD | family | Methanobacteriaceae | Weighted mode | -0.0117 | 0.0906 | 0.9005 | 0.99(0.83-1.18) |
| PTSD | family | Oxalobacteraceae | MR Egger | -0.1651 | 0.1713 | 0.3542 | 0.85(0.61-1.19) |
| PTSD | family | Oxalobacteraceae | Weighted median | -0.0205 | 0.0567 | 0.7176 | 0.98(0.88-1.09) |
| PTSD | family | Oxalobacteraceae | IVW | 0.0068 | 0.0428 | 0.8745 | 1.01(0.93-1.09) |
| PTSD | family | Oxalobacteraceae | Simple mode | -0.0173 | 0.0962 | 0.8601 | 0.98(0.81-1.19) |
| PTSD | family | Oxalobacteraceae | Weighted mode | -0.0189 | 0.0971 | 0.8489 | 0.98(0.81-1.19) |
| PTSD | family | Pasteurellaceae | MR Egger | -0.0910 | 0.1169 | 0.4513 | 0.91(0.73-1.15) |
| PTSD | family | Pasteurellaceae | Weighted median | -0.0225 | 0.0716 | 0.7535 | 0.98(0.85-1.13) |
| PTSD | family | Pasteurellaceae | IVW | -0.0461 | 0.0513 | 0.3690 | 0.95(0.86-1.06) |
| PTSD | family | Pasteurellaceae | Simple mode | -0.0019 | 0.1220 | 0.9876 | 1.00(0.79-1.27) |
| PTSD | family | Pasteurellaceae | Weighted mode | -0.0114 | 0.1093 | 0.9182 | 0.99(0.80-1.22) |
| PTSD | family | Peptococcaceae | MR Egger | -0.0047 | 0.1594 | 0.9773 | 1.00(0.73-1.36) |
| PTSD | family | Peptococcaceae | Weighted median | -0.0733 | 0.0883 | 0.4063 | 0.93(0.78-1.10) |
| PTSD | family | Peptococcaceae | IVW | -0.0555 | 0.0654 | 0.3964 | 0.95(0.83-1.08) |
| PTSD | family | Peptococcaceae | Simple mode | -0.0479 | 0.1325 | 0.7259 | 0.95(0.74-1.24) |
| PTSD | family | Peptococcaceae | Weighted mode | -0.0662 | 0.1147 | 0.5782 | 0.94(0.75-1.17) |
| PTSD | family | Peptostreptococcaceae | MR Egger | -0.0648 | 0.1901 | 0.7392 | 0.94(0.65-1.36) |
| PTSD | family | Peptostreptococcaceae | Weighted median | -0.0352 | 0.0934 | 0.7066 | 0.97(0.80-1.16) |
| PTSD | family | Peptostreptococcaceae | IVW | 0.0002 | 0.0817 | 0.9978 | 1.00(0.85-1.17) |
| PTSD | family | Peptostreptococcaceae | Simple mode | -0.0278 | 0.1433 | 0.8490 | 0.97(0.73-1.29) |
| PTSD | family | Peptostreptococcaceae | Weighted mode | -0.0487 | 0.1168 | 0.6837 | 0.95(0.76-1.20) |
| PTSD | family | Porphyromonadaceae | MR Egger | 0.1929 | 0.4040 | 0.6458 | 1.21(0.55-2.68) |
| PTSD | family | Porphyromonadaceae | Weighted median | -0.0292 | 0.1436 | 0.8387 | 0.97(0.73-1.29) |
| PTSD | family | Porphyromonadaceae | IVW | -0.2109 | 0.1065 | 0.0476 | 0.81(0.66-1.00) |
| PTSD | family | Porphyromonadaceae | Simple mode | 0.0129 | 0.2139 | 0.9531 | 1.01(0.67-1.54) |
| PTSD | family | Porphyromonadaceae | Weighted mode | 0.0097 | 0.1832 | 0.9589 | 1.01(0.71-1.45) |
| PTSD | family | Prevotellaceae | MR Egger | 0.0503 | 0.2397 | 0.8367 | 1.05(0.66-1.68) |
| PTSD | family | Prevotellaceae | Weighted median | -0.0577 | 0.0933 | 0.5366 | 0.94(0.79-1.13) |
| PTSD | family | Prevotellaceae | IVW | -0.0363 | 0.0668 | 0.5865 | 0.96(0.85-1.10) |
| PTSD | family | Prevotellaceae | Simple mode | -0.1859 | 0.1663 | 0.2814 | 0.83(0.60-1.15) |
| PTSD | family | Prevotellaceae | Weighted mode | -0.1369 | 0.1498 | 0.3750 | 0.87(0.65-1.17) |
| PTSD | family | Rhodospirillaceae | MR Egger | -0.1735 | 0.3397 | 0.6189 | 0.84(0.43-1.64) |
| PTSD | family | Rhodospirillaceae | Weighted median | -0.0149 | 0.0801 | 0.8525 | 0.99(0.84-1.15) |
| PTSD | family | Rhodospirillaceae | IVW | -0.0360 | 0.0571 | 0.5285 | 0.96(0.86-1.08) |
| PTSD | family | Rhodospirillaceae | Simple mode | 0.0587 | 0.1371 | 0.6753 | 1.06(0.81-1.39) |
| PTSD | family | Rhodospirillaceae | Weighted mode | 0.0793 | 0.1383 | 0.5760 | 1.08(0.83-1.42) |
| PTSD | family | Rikenellaceae | MR Egger | -0.1487 | 0.2017 | 0.4698 | 0.86(0.58-1.28) |
| PTSD | family | Rikenellaceae | Weighted median | -0.0330 | 0.0868 | 0.7041 | 0.97(0.82-1.15) |
| PTSD | family | Rikenellaceae | IVW | 0.0117 | 0.0639 | 0.8542 | 1.01(0.89-1.15) |
| PTSD | family | Rikenellaceae | Simple mode | -0.0674 | 0.1474 | 0.6524 | 0.93(0.70-1.25) |
| PTSD | family | Rikenellaceae | Weighted mode | -0.0546 | 0.1404 | 0.7013 | 0.95(0.72-1.25) |
| PTSD | family | Ruminococcaceae | MR Egger | -0.0034 | 0.1820 | 0.9856 | 1.00(0.70-1.42) |
| PTSD | family | Ruminococcaceae | Weighted median | 0.0691 | 0.1128 | 0.5398 | 1.07(0.86-1.34) |
| PTSD | family | Ruminococcaceae | IVW | -0.0180 | 0.0844 | 0.8313 | 0.98(0.83-1.16) |
| PTSD | family | Ruminococcaceae | Simple mode | 0.0975 | 0.1830 | 0.6072 | 1.10(0.77-1.58) |
| PTSD | family | Ruminococcaceae | Weighted mode | 0.1053 | 0.1530 | 0.5086 | 1.11(0.82-1.50) |
| PTSD | family | Streptococcaceae | MR Egger | 0.4274 | 0.2802 | 0.1495 | 1.53(0.89-2.66) |
| PTSD | family | Streptococcaceae | Weighted median | -0.0189 | 0.0963 | 0.8446 | 0.98(0.81-1.19) |
| PTSD | family | Streptococcaceae | IVW | 0.0318 | 0.0720 | 0.6586 | 1.03(0.90-1.19) |
| PTSD | family | Streptococcaceae | Simple mode | -0.0650 | 0.1716 | 0.7102 | 0.94(0.67-1.31) |
| PTSD | family | Streptococcaceae | Weighted mode | -0.0880 | 0.1497 | 0.5656 | 0.92(0.68-1.23) |
| PTSD | family | Veillonellaceae | MR Egger | -0.1363 | 0.1239 | 0.2867 | 0.87(0.68-1.11) |
| PTSD | family | Veillonellaceae | Weighted median | -0.0389 | 0.0851 | 0.6476 | 0.96(0.81-1.14) |
| PTSD | family | Veillonellaceae | IVW | -0.1183 | 0.0571 | 0.0382 | 0.89(0.79-0.99) |
| PTSD | family | Veillonellaceae | Simple mode | -0.0116 | 0.1417 | 0.9355 | 0.99(0.75-1.30) |
| PTSD | family | Veillonellaceae | Weighted mode | 0.0036 | 0.1488 | 0.9807 | 1.00(0.75-1.34) |
| PTSD | family | Verrucomicrobiaceae | MR Egger | 0.1825 | 0.2581 | 0.4956 | 1.20(0.72-1.99) |
| PTSD | family | Verrucomicrobiaceae | Weighted median | 0.1166 | 0.0952 | 0.2210 | 1.12(0.93-1.35) |
| PTSD | family | Verrucomicrobiaceae | IVW | 0.0826 | 0.0707 | 0.2427 | 1.09(0.95-1.25) |
| PTSD | family | Verrucomicrobiaceae | Simple mode | 0.2242 | 0.1815 | 0.2424 | 1.25(0.88-1.79) |
| PTSD | family | Verrucomicrobiaceae | Weighted mode | 0.2508 | 0.1856 | 0.2036 | 1.29(0.89-1.85) |
| PTSD | family | Victivallaceae | MR Egger | -0.1779 | 0.2234 | 0.4442 | 0.84(0.54-1.30) |
| PTSD | family | Victivallaceae | Weighted median | -0.0468 | 0.0574 | 0.4155 | 0.95(0.85-1.07) |
| PTSD | family | Victivallaceae | IVW | -0.0263 | 0.0481 | 0.5841 | 0.97(0.89-1.07) |
| PTSD | family | Victivallaceae | Simple mode | -0.0613 | 0.0935 | 0.5254 | 0.94(0.78-1.13) |
| PTSD | family | Victivallaceae | Weighted mode | -0.0699 | 0.0842 | 0.4243 | 0.93(0.79-1.10) |
| PTSD | genus | Clostridiuminnocuumgroup | MR Egger | 0.1328 | 0.4930 | 0.7955 | 1.14(0.43-3.00) |
| PTSD | genus | Clostridiuminnocuumgroup | Weighted median | 0.0657 | 0.0763 | 0.3892 | 1.07(0.92-1.24) |
| PTSD | genus | Clostridiuminnocuumgroup | IVW | -0.0035 | 0.0929 | 0.9703 | 1.00(0.83-1.20) |
| PTSD | genus | Clostridiuminnocuumgroup | Simple mode | 0.0704 | 0.0979 | 0.4924 | 1.07(0.89-1.30) |
| PTSD | genus | Clostridiuminnocuumgroup | Weighted mode | 0.0674 | 0.0931 | 0.4902 | 1.07(0.89-1.28) |
| PTSD | genus | Eubacteriumbrachygroup | MR Egger | 0.3433 | 0.2224 | 0.1666 | 1.41(0.91-2.18) |
| PTSD | genus | Eubacteriumbrachygroup | Weighted median | -0.0739 | 0.0699 | 0.2903 | 0.93(0.81-1.07) |
| PTSD | genus | Eubacteriumbrachygroup | IVW | -0.0394 | 0.0496 | 0.4273 | 0.96(0.87-1.06) |
| PTSD | genus | Eubacteriumbrachygroup | Simple mode | -0.1229 | 0.1018 | 0.2620 | 0.88(0.72-1.08) |
| PTSD | genus | Eubacteriumbrachygroup | Weighted mode | -0.1181 | 0.1015 | 0.2780 | 0.89(0.73-1.08) |
| PTSD | genus | Eubacteriumcoprostanoligenesgroup | MR Egger | -0.2292 | 0.3198 | 0.4885 | 0.80(0.42-1.49) |
| PTSD | genus | Eubacteriumcoprostanoligenesgroup | Weighted median | 0.0405 | 0.1102 | 0.7133 | 1.04(0.84-1.29) |
| PTSD | genus | Eubacteriumcoprostanoligenesgroup | IVW | 0.0684 | 0.0819 | 0.4038 | 1.07(0.91-1.26) |
| PTSD | genus | Eubacteriumcoprostanoligenesgroup | Simple mode | 0.1229 | 0.1850 | 0.5190 | 1.13(0.79-1.62) |
| PTSD | genus | Eubacteriumcoprostanoligenesgroup | Weighted mode | 0.1427 | 0.1815 | 0.4468 | 1.15(0.81-1.65) |
| PTSD | genus | Eubacteriumeligensgroup | MR Egger | -0.4784 | 0.4244 | 0.3108 | 0.62(0.27-1.42) |
| PTSD | genus | Eubacteriumeligensgroup | Weighted median | 0.0361 | 0.1471 | 0.8059 | 1.04(0.78-1.38) |
| PTSD | genus | Eubacteriumeligensgroup | IVW | 0.0262 | 0.1053 | 0.8033 | 1.03(0.84-1.26) |
| PTSD | genus | Eubacteriumeligensgroup | Simple mode | -0.1731 | 0.2325 | 0.4847 | 0.84(0.53-1.33) |
| PTSD | genus | Eubacteriumeligensgroup | Weighted mode | -0.1392 | 0.2269 | 0.5621 | 0.87(0.56-1.36) |
| PTSD | genus | Eubacteriumfissicatenagroup | MR Egger | 0.1628 | 0.2559 | 0.5449 | 1.18(0.71-1.94) |
| PTSD | genus | Eubacteriumfissicatenagroup | Weighted median | -0.0500 | 0.0627 | 0.4255 | 0.95(0.84-1.08) |
| PTSD | genus | Eubacteriumfissicatenagroup | IVW | -0.0314 | 0.0478 | 0.5111 | 0.97(0.88-1.06) |
| PTSD | genus | Eubacteriumfissicatenagroup | Simple mode | -0.0706 | 0.0882 | 0.4469 | 0.93(0.78-1.11) |
| PTSD | genus | Eubacteriumfissicatenagroup | Weighted mode | -0.0622 | 0.0923 | 0.5192 | 0.94(0.78-1.13) |
| PTSD | genus | Eubacteriumhalliigroup | MR Egger | 0.3483 | 0.1710 | 0.0611 | 1.42(1.01-1.98) |
| PTSD | genus | Eubacteriumhalliigroup | Weighted median | 0.0760 | 0.1026 | 0.4591 | 1.08(0.88-1.32) |
| PTSD | genus | Eubacteriumhalliigroup | IVW | 0.0611 | 0.0861 | 0.4781 | 1.06(0.90-1.26) |
| PTSD | genus | Eubacteriumhalliigroup | Simple mode | 0.3107 | 0.2086 | 0.1571 | 1.36(0.91-2.05) |
| PTSD | genus | Eubacteriumhalliigroup | Weighted mode | 0.2977 | 0.2059 | 0.1689 | 1.35(0.90-2.02) |
| PTSD | genus | Eubacteriumnodatumgroup | MR Egger | -0.1143 | 0.2112 | 0.6014 | 0.89(0.59-1.35) |
| PTSD | genus | Eubacteriumnodatumgroup | Weighted median | -0.0624 | 0.0543 | 0.2506 | 0.94(0.84-1.05) |
| PTSD | genus | Eubacteriumnodatumgroup | IVW | -0.0128 | 0.0461 | 0.7804 | 0.99(0.90-1.08) |
| PTSD | genus | Eubacteriumnodatumgroup | Simple mode | -0.0761 | 0.0965 | 0.4487 | 0.93(0.77-1.12) |
| PTSD | genus | Eubacteriumnodatumgroup | Weighted mode | -0.0800 | 0.0912 | 0.4015 | 0.92(0.77-1.10) |
| PTSD | genus | Eubacteriumoxidoreducensgroup | MR Egger | 0.2139 | 0.2746 | 0.4929 | 1.24(0.72-2.12) |
| PTSD | genus | Eubacteriumoxidoreducensgroup | Weighted median | 0.1201 | 0.0955 | 0.2083 | 1.13(0.94-1.36) |
| PTSD | genus | Eubacteriumoxidoreducensgroup | IVW | 0.0789 | 0.0734 | 0.2825 | 1.08(0.94-1.25) |
| PTSD | genus | Eubacteriumoxidoreducensgroup | Simple mode | 0.1228 | 0.1279 | 0.3912 | 1.13(0.88-1.45) |
| PTSD | genus | Eubacteriumoxidoreducensgroup | Weighted mode | 0.1228 | 0.1344 | 0.4127 | 1.13(0.87-1.47) |
| PTSD | genus | Eubacteriumrectalegroup | MR Egger | -0.1262 | 0.4748 | 0.7980 | 0.88(0.35-2.24) |
| PTSD | genus | Eubacteriumrectalegroup | Weighted median | 0.0119 | 0.1472 | 0.9354 | 1.01(0.76-1.35) |
| PTSD | genus | Eubacteriumrectalegroup | IVW | -0.0261 | 0.1218 | 0.8306 | 0.97(0.77-1.24) |
| PTSD | genus | Eubacteriumrectalegroup | Simple mode | -0.0393 | 0.2414 | 0.8748 | 0.96(0.60-1.54) |
| PTSD | genus | Eubacteriumrectalegroup | Weighted mode | -0.0258 | 0.2320 | 0.9142 | 0.97(0.62-1.54) |
| PTSD | genus | Eubacteriumruminantiumgroup | MR Egger | 0.1759 | 0.1746 | 0.3285 | 1.19(0.85-1.68) |
| PTSD | genus | Eubacteriumruminantiumgroup | Weighted median | 0.0232 | 0.0666 | 0.7276 | 1.02(0.90-1.17) |
| PTSD | genus | Eubacteriumruminantiumgroup | IVW | -0.0290 | 0.0476 | 0.5421 | 0.97(0.88-1.07) |
| PTSD | genus | Eubacteriumruminantiumgroup | Simple mode | 0.0584 | 0.1241 | 0.6441 | 1.06(0.83-1.35) |
| PTSD | genus | Eubacteriumruminantiumgroup | Weighted mode | 0.0559 | 0.1189 | 0.6442 | 1.06(0.84-1.33) |
| PTSD | genus | Eubacteriumventriosumgroup | MR Egger | -0.1108 | 0.3641 | 0.7658 | 0.90(0.44-1.83) |
| PTSD | genus | Eubacteriumventriosumgroup | Weighted median | 0.1377 | 0.1106 | 0.2133 | 1.15(0.92-1.43) |
| PTSD | genus | Eubacteriumventriosumgroup | IVW | 0.0456 | 0.0777 | 0.5571 | 1.05(0.90-1.22) |
| PTSD | genus | Eubacteriumventriosumgroup | Simple mode | 0.2278 | 0.2066 | 0.2887 | 1.26(0.84-1.88) |
| PTSD | genus | Eubacteriumventriosumgroup | Weighted mode | 0.2349 | 0.2107 | 0.2838 | 1.26(0.84-1.91) |
| PTSD | genus | Eubacteriumxylanophilumgroup | MR Egger | 0.4881 | 0.3845 | 0.2448 | 1.63(0.77-3.46) |
| PTSD | genus | Eubacteriumxylanophilumgroup | Weighted median | -0.0143 | 0.1192 | 0.9049 | 0.99(0.78-1.25) |
| PTSD | genus | Eubacteriumxylanophilumgroup | IVW | 0.0592 | 0.1254 | 0.6368 | 1.06(0.83-1.36) |
| PTSD | genus | Eubacteriumxylanophilumgroup | Simple mode | -0.0535 | 0.1701 | 0.7610 | 0.95(0.68-1.32) |
| PTSD | genus | Eubacteriumxylanophilumgroup | Weighted mode | -0.0535 | 0.1454 | 0.7222 | 0.95(0.71-1.26) |
| PTSD | genus | Ruminococcusgauvreauiigroup | MR Egger | 0.0846 | 0.3444 | 0.8109 | 1.09(0.55-2.14) |
| PTSD | genus | Ruminococcusgauvreauiigroup | Weighted median | -0.0043 | 0.1055 | 0.9675 | 1.00(0.81-1.22) |
| PTSD | genus | Ruminococcusgauvreauiigroup | IVW | 0.0452 | 0.0755 | 0.5490 | 1.05(0.90-1.21) |
| PTSD | genus | Ruminococcusgauvreauiigroup | Simple mode | -0.0756 | 0.2035 | 0.7173 | 0.93(0.62-1.38) |
| PTSD | genus | Ruminococcusgauvreauiigroup | Weighted mode | -0.0819 | 0.1900 | 0.6749 | 0.92(0.63-1.34) |
| PTSD | genus | Ruminococcusgnavusgroup | MR Egger | -0.0574 | 0.2365 | 0.8131 | 0.94(0.59-1.50) |
| PTSD | genus | Ruminococcusgnavusgroup | Weighted median | -0.0521 | 0.0666 | 0.4345 | 0.95(0.83-1.08) |
| PTSD | genus | Ruminococcusgnavusgroup | IVW | -0.0221 | 0.0519 | 0.6701 | 0.98(0.88-1.08) |
| PTSD | genus | Ruminococcusgnavusgroup | Simple mode | -0.0587 | 0.1083 | 0.5990 | 0.94(0.76-1.17) |
| PTSD | genus | Ruminococcusgnavusgroup | Weighted mode | -0.0694 | 0.1070 | 0.5299 | 0.93(0.76-1.15) |
| PTSD | genus | Ruminococcustorquesgroup | MR Egger | -0.1678 | 0.2407 | 0.5053 | 0.85(0.53-1.36) |
| PTSD | genus | Ruminococcustorquesgroup | Weighted median | -0.1624 | 0.1181 | 0.1693 | 0.85(0.67-1.07) |
| PTSD | genus | Ruminococcustorquesgroup | IVW | -0.1368 | 0.0885 | 0.1222 | 0.87(0.73-1.04) |
| PTSD | genus | Ruminococcustorquesgroup | Simple mode | -0.1714 | 0.1818 | 0.3705 | 0.84(0.59-1.20) |
| PTSD | genus | Ruminococcustorquesgroup | Weighted mode | -0.1714 | 0.1624 | 0.3190 | 0.84(0.61-1.16) |
| PTSD | genus | Actinomyces | MR Egger | -0.1631 | 0.2719 | 0.5748 | 0.85(0.50-1.45) |
| PTSD | genus | Actinomyces | Weighted median | 0.0113 | 0.1031 | 0.9127 | 1.01(0.83-1.24) |
| PTSD | genus | Actinomyces | IVW | 0.0238 | 0.0973 | 0.8071 | 1.02(0.85-1.24) |
| PTSD | genus | Actinomyces | Simple mode | 0.1898 | 0.1854 | 0.3455 | 1.21(0.84-1.74) |
| PTSD | genus | Actinomyces | Weighted mode | 0.0026 | 0.1673 | 0.9882 | 1.00(0.72-1.39) |
| PTSD | genus | Adlercreutzia | MR Egger | -0.0296 | 0.3377 | 0.9329 | 0.97(0.50-1.88) |
| PTSD | genus | Adlercreutzia | Weighted median | 0.0306 | 0.0878 | 0.7276 | 1.03(0.87-1.22) |
| PTSD | genus | Adlercreutzia | IVW | 0.0387 | 0.0744 | 0.6035 | 1.04(0.90-1.20) |
| PTSD | genus | Adlercreutzia | Simple mode | 0.0212 | 0.1317 | 0.8765 | 1.02(0.79-1.32) |
| PTSD | genus | Adlercreutzia | Weighted mode | 0.0181 | 0.1387 | 0.8997 | 1.02(0.78-1.34) |
| PTSD | genus | Akkermansia | MR Egger | 0.1797 | 0.2577 | 0.5015 | 1.20(0.72-1.98) |
| PTSD | genus | Akkermansia | Weighted median | 0.1159 | 0.1000 | 0.2464 | 1.12(0.92-1.37) |
| PTSD | genus | Akkermansia | IVW | 0.0825 | 0.0707 | 0.2437 | 1.09(0.95-1.25) |
| PTSD | genus | Akkermansia | Simple mode | 0.2244 | 0.1930 | 0.2696 | 1.25(0.86-1.83) |
| PTSD | genus | Akkermansia | Weighted mode | 0.2510 | 0.1832 | 0.1980 | 1.29(0.90-1.84) |
| PTSD | genus | Alistipes | MR Egger | 0.0451 | 0.4680 | 0.9249 | 1.05(0.42-2.62) |
| PTSD | genus | Alistipes | Weighted median | -0.0244 | 0.1163 | 0.8338 | 0.98(0.78-1.23) |
| PTSD | genus | Alistipes | IVW | 0.0936 | 0.0919 | 0.3084 | 1.10(0.92-1.31) |
| PTSD | genus | Alistipes | Simple mode | -0.0932 | 0.2058 | 0.6581 | 0.91(0.61-1.36) |
| PTSD | genus | Alistipes | Weighted mode | -0.0890 | 0.1785 | 0.6262 | 0.91(0.64-1.30) |
| PTSD | genus | Allisonella | MR Egger | -0.3341 | 0.2983 | 0.3054 | 0.72(0.40-1.28) |
| PTSD | genus | Allisonella | Weighted median | -0.0005 | 0.0574 | 0.9937 | 1.00(0.89-1.12) |
| PTSD | genus | Allisonella | IVW | -0.0065 | 0.0445 | 0.8844 | 0.99(0.91-1.08) |
| PTSD | genus | Allisonella | Simple mode | 0.0185 | 0.0876 | 0.8388 | 1.02(0.86-1.21) |
| PTSD | genus | Allisonella | Weighted mode | -0.0035 | 0.0856 | 0.9684 | 1.00(0.84-1.18) |
| PTSD | genus | Alloprevotella | MR Egger | 0.1329 | 0.5640 | 0.8252 | 1.14(0.38-3.45) |
| PTSD | genus | Alloprevotella | Weighted median | 0.0480 | 0.0666 | 0.4711 | 1.05(0.92-1.20) |
| PTSD | genus | Alloprevotella | IVW | 0.0575 | 0.0543 | 0.2900 | 1.06(0.95-1.18) |
| PTSD | genus | Alloprevotella | Simple mode | -0.0070 | 0.1085 | 0.9510 | 0.99(0.80-1.23) |
| PTSD | genus | Alloprevotella | Weighted mode | -0.0053 | 0.1074 | 0.9624 | 0.99(0.81-1.23) |
| PTSD | genus | Anaerofilum | MR Egger | -0.0991 | 0.2718 | 0.7238 | 0.91(0.53-1.54) |
| PTSD | genus | Anaerofilum | Weighted median | -0.0474 | 0.0668 | 0.4775 | 0.95(0.84-1.09) |
| PTSD | genus | Anaerofilum | IVW | -0.0141 | 0.0504 | 0.7801 | 0.99(0.89-1.09) |
| PTSD | genus | Anaerofilum | Simple mode | -0.0809 | 0.1116 | 0.4847 | 0.92(0.74-1.15) |
| PTSD | genus | Anaerofilum | Weighted mode | -0.0809 | 0.1053 | 0.4600 | 0.92(0.75-1.13) |
| PTSD | genus | Anaerostipes | MR Egger | 0.2770 | 0.3028 | 0.3800 | 1.32(0.73-2.39) |
| PTSD | genus | Anaerostipes | Weighted median | -0.0844 | 0.1120 | 0.4510 | 0.92(0.74-1.14) |
| PTSD | genus | Anaerostipes | IVW | -0.0943 | 0.0890 | 0.2891 | 0.91(0.76-1.08) |
| PTSD | genus | Anaerostipes | Simple mode | -0.0650 | 0.1804 | 0.7248 | 0.94(0.66-1.33) |
| PTSD | genus | Anaerostipes | Weighted mode | -0.0650 | 0.1516 | 0.6755 | 0.94(0.70-1.26) |
| PTSD | genus | Anaerotruncus | MR Egger | 0.0760 | 0.2889 | 0.7974 | 1.08(0.61-1.90) |
| PTSD | genus | Anaerotruncus | Weighted median | -0.1451 | 0.1115 | 0.1933 | 0.86(0.70-1.08) |
| PTSD | genus | Anaerotruncus | IVW | -0.0042 | 0.0871 | 0.9618 | 1.00(0.84-1.18) |
| PTSD | genus | Anaerotruncus | Simple mode | -0.2347 | 0.1923 | 0.2458 | 0.79(0.54-1.15) |
| PTSD | genus | Anaerotruncus | Weighted mode | -0.2347 | 0.1736 | 0.2014 | 0.79(0.56-1.11) |
| PTSD | genus | Bacteroides | MR Egger | 0.2136 | 0.6319 | 0.7468 | 1.24(0.36-4.27) |
| PTSD | genus | Bacteroides | Weighted median | 0.1530 | 0.1438 | 0.2875 | 1.17(0.88-1.54) |
| PTSD | genus | Bacteroides | IVW | 0.0684 | 0.1084 | 0.5284 | 1.07(0.87-1.32) |
| PTSD | genus | Bacteroides | Simple mode | 0.2550 | 0.2444 | 0.3314 | 1.29(0.80-2.08) |
| PTSD | genus | Bacteroides | Weighted mode | 0.2705 | 0.2250 | 0.2683 | 1.31(0.84-2.04) |
| PTSD | genus | Barnesiella | MR Egger | 0.0173 | 0.3299 | 0.9591 | 1.02(0.53-1.94) |
| PTSD | genus | Barnesiella | Weighted median | -0.0548 | 0.1150 | 0.6338 | 0.95(0.76-1.19) |
| PTSD | genus | Barnesiella | IVW | -0.1348 | 0.0966 | 0.1629 | 0.87(0.72-1.06) |
| PTSD | genus | Barnesiella | Simple mode | 0.1052 | 0.2008 | 0.6092 | 1.11(0.75-1.65) |
| PTSD | genus | Barnesiella | Weighted mode | 0.0813 | 0.1739 | 0.6480 | 1.08(0.77-1.53) |
| PTSD | genus | Bifidobacterium | MR Egger | 0.0123 | 0.1964 | 0.9509 | 1.01(0.69-1.49) |
| PTSD | genus | Bifidobacterium | Weighted median | -0.0203 | 0.0953 | 0.8315 | 0.98(0.81-1.18) |
| PTSD | genus | Bifidobacterium | IVW | -0.0562 | 0.0685 | 0.4118 | 0.95(0.83-1.08) |
| PTSD | genus | Bifidobacterium | Simple mode | 0.1513 | 0.1820 | 0.4196 | 1.16(0.81-1.66) |
| PTSD | genus | Bifidobacterium | Weighted mode | 0.0972 | 0.1370 | 0.4895 | 1.10(0.84-1.44) |
| PTSD | genus | Bilophila | MR Egger | -0.0945 | 0.3403 | 0.7865 | 0.91(0.47-1.77) |
| PTSD | genus | Bilophila | Weighted median | -0.0487 | 0.0958 | 0.6110 | 0.95(0.79-1.15) |
| PTSD | genus | Bilophila | IVW | 0.0092 | 0.0735 | 0.9004 | 1.01(0.87-1.17) |
| PTSD | genus | Bilophila | Simple mode | -0.0823 | 0.1551 | 0.6053 | 0.92(0.68-1.25) |
| PTSD | genus | Bilophila | Weighted mode | -0.0717 | 0.1494 | 0.6398 | 0.93(0.69-1.25) |
| PTSD | genus | Blautia | MR Egger | -0.0096 | 0.1668 | 0.9552 | 0.99(0.71-1.37) |
| PTSD | genus | Blautia | Weighted median | -0.0710 | 0.1006 | 0.4801 | 0.93(0.76-1.13) |
| PTSD | genus | Blautia | IVW | -0.0765 | 0.0758 | 0.3131 | 0.93(0.80-1.07) |
| PTSD | genus | Blautia | Simple mode | -0.0484 | 0.1509 | 0.7540 | 0.95(0.71-1.28) |
| PTSD | genus | Blautia | Weighted mode | -0.0612 | 0.1310 | 0.6487 | 0.94(0.73-1.22) |
| PTSD | genus | Butyricicoccus | MR Egger | 0.0432 | 0.1489 | 0.7814 | 1.04(0.78-1.40) |
| PTSD | genus | Butyricicoccus | Weighted median | -0.0122 | 0.1034 | 0.9065 | 0.99(0.81-1.21) |
| PTSD | genus | Butyricicoccus | IVW | 0.0311 | 0.0817 | 0.7038 | 1.03(0.88-1.21) |
| PTSD | genus | Butyricicoccus | Simple mode | -0.0882 | 0.1689 | 0.6176 | 0.92(0.66-1.27) |
| PTSD | genus | Butyricicoccus | Weighted mode | -0.0529 | 0.1268 | 0.6893 | 0.95(0.74-1.22) |
| PTSD | genus | Butyricimonas | MR Egger | 0.0171 | 0.2117 | 0.9372 | 1.02(0.67-1.54) |
| PTSD | genus | Butyricimonas | Weighted median | -0.0188 | 0.0859 | 0.8264 | 0.98(0.83-1.16) |
| PTSD | genus | Butyricimonas | IVW | -0.0265 | 0.0635 | 0.6763 | 0.97(0.86-1.10) |
| PTSD | genus | Butyricimonas | Simple mode | -0.0016 | 0.1441 | 0.9915 | 1.00(0.75-1.32) |
| PTSD | genus | Butyricimonas | Weighted mode | -0.0233 | 0.1383 | 0.8690 | 0.98(0.75-1.28) |
| PTSD | genus | Butyrivibrio | MR Egger | -0.1882 | 0.2136 | 0.3943 | 0.83(0.55-1.26) |
| PTSD | genus | Butyrivibrio | Weighted median | -0.0089 | 0.0526 | 0.8656 | 0.99(0.89-1.10) |
| PTSD | genus | Butyrivibrio | IVW | 0.0061 | 0.0462 | 0.8956 | 1.01(0.92-1.10) |
| PTSD | genus | Butyrivibrio | Simple mode | -0.0272 | 0.1110 | 0.8103 | 0.97(0.78-1.21) |
| PTSD | genus | Butyrivibrio | Weighted mode | -0.0067 | 0.1082 | 0.9514 | 0.99(0.80-1.23) |
| PTSD | genus | CandidatusSoleaferrea | MR Egger | 0.1802 | 0.7273 | 0.8115 | 1.20(0.29-4.98) |
| PTSD | genus | CandidatusSoleaferrea | Weighted median | -0.0419 | 0.0881 | 0.6342 | 0.96(0.81-1.14) |
| PTSD | genus | CandidatusSoleaferrea | IVW | 0.0048 | 0.0630 | 0.9392 | 1.00(0.89-1.14) |
| PTSD | genus | CandidatusSoleaferrea | Simple mode | -0.1395 | 0.1586 | 0.4047 | 0.87(0.64-1.19) |
| PTSD | genus | CandidatusSoleaferrea | Weighted mode | -0.1372 | 0.1685 | 0.4390 | 0.87(0.63-1.21) |
| PTSD | genus | Catenibacterium | MR Egger | -0.5050 | 0.6218 | 0.4761 | 0.60(0.18-2.04) |
| PTSD | genus | Catenibacterium | Weighted median | -0.1131 | 0.0801 | 0.1580 | 0.89(0.76-1.04) |
| PTSD | genus | Catenibacterium | IVW | -0.1215 | 0.0630 | 0.0539 | 0.89(0.78-1.00) |
| PTSD | genus | Catenibacterium | Simple mode | -0.1185 | 0.1003 | 0.3032 | 0.89(0.73-1.08) |
| PTSD | genus | Catenibacterium | Weighted mode | -0.1174 | 0.1036 | 0.3202 | 0.89(0.73-1.09) |
| PTSD | genus | ChristensenellaceaeR | MR Egger | -0.1372 | 0.2758 | 0.6322 | 0.87(0.51-1.50) |
| PTSD | genus | ChristensenellaceaeR | Weighted median | 0.0438 | 0.1243 | 0.7247 | 1.04(0.82-1.33) |
| PTSD | genus | ChristensenellaceaeR | IVW | 0.0540 | 0.0936 | 0.5643 | 1.06(0.88-1.27) |
| PTSD | genus | ChristensenellaceaeR | Simple mode | 0.0528 | 0.1947 | 0.7925 | 1.05(0.72-1.54) |
| PTSD | genus | ChristensenellaceaeR | Weighted mode | 0.0499 | 0.1870 | 0.7955 | 1.05(0.73-1.52) |
| PTSD | genus | Clostridiumsensustricto1 | MR Egger | -0.0448 | 0.1843 | 0.8175 | 0.96(0.67-1.37) |
| PTSD | genus | Clostridiumsensustricto1 | Weighted median | -0.0196 | 0.1034 | 0.8499 | 0.98(0.80-1.20) |
| PTSD | genus | Clostridiumsensustricto1 | IVW | 0.0165 | 0.0844 | 0.8450 | 1.02(0.86-1.20) |
| PTSD | genus | Clostridiumsensustricto1 | Simple mode | -0.0554 | 0.1480 | 0.7210 | 0.95(0.71-1.26) |
| PTSD | genus | Clostridiumsensustricto1 | Weighted mode | -0.0446 | 0.1335 | 0.7497 | 0.96(0.74-1.24) |
| PTSD | genus | Collinsella | MR Egger | 0.1609 | 0.3364 | 0.6470 | 1.17(0.61-2.27) |
| PTSD | genus | Collinsella | Weighted median | 0.0428 | 0.1227 | 0.7272 | 1.04(0.82-1.33) |
| PTSD | genus | Collinsella | IVW | 0.0532 | 0.0907 | 0.5570 | 1.05(0.88-1.26) |
| PTSD | genus | Collinsella | Simple mode | -0.0188 | 0.2080 | 0.9301 | 0.98(0.65-1.48) |
| PTSD | genus | Collinsella | Weighted mode | -0.0255 | 0.1889 | 0.8959 | 0.97(0.67-1.41) |
| PTSD | genus | Coprobacter | MR Egger | -0.2973 | 0.2439 | 0.2538 | 0.74(0.46-1.20) |
| PTSD | genus | Coprobacter | Weighted median | -0.1010 | 0.0769 | 0.1894 | 0.90(0.78-1.05) |
| PTSD | genus | Coprobacter | IVW | -0.0603 | 0.0719 | 0.4015 | 0.94(0.82-1.08) |
| PTSD | genus | Coprobacter | Simple mode | -0.1322 | 0.1240 | 0.3113 | 0.88(0.69-1.12) |
| PTSD | genus | Coprobacter | Weighted mode | -0.1473 | 0.1125 | 0.2194 | 0.86(0.69-1.08) |
| PTSD | genus | Coprococcus1 | MR Egger | -0.1096 | 0.2217 | 0.6317 | 0.90(0.58-1.38) |
| PTSD | genus | Coprococcus1 | Weighted median | 0.1137 | 0.1137 | 0.3172 | 1.12(0.90-1.40) |
| PTSD | genus | Coprococcus1 | IVW | 0.0452 | 0.0873 | 0.6042 | 1.05(0.88-1.24) |
| PTSD | genus | Coprococcus1 | Simple mode | 0.1782 | 0.2051 | 0.4036 | 1.20(0.80-1.79) |
| PTSD | genus | Coprococcus1 | Weighted mode | 0.1659 | 0.1947 | 0.4125 | 1.18(0.81-1.73) |
| PTSD | genus | Coprococcus2 | MR Egger | -0.5482 | 0.4885 | 0.2988 | 0.58(0.22-1.51) |
| PTSD | genus | Coprococcus2 | Weighted median | 0.1687 | 0.1074 | 0.1164 | 1.18(0.96-1.46) |
| PTSD | genus | Coprococcus2 | IVW | 0.0941 | 0.0824 | 0.2534 | 1.10(0.93-1.29) |
| PTSD | genus | Coprococcus2 | Simple mode | 0.1992 | 0.1772 | 0.2936 | 1.22(0.86-1.73) |
| PTSD | genus | Coprococcus2 | Weighted mode | 0.2060 | 0.1822 | 0.2911 | 1.23(0.86-1.76) |
| PTSD | genus | Coprococcus3 | MR Egger | 0.2330 | 0.3596 | 0.5351 | 1.26(0.62-2.55) |
| PTSD | genus | Coprococcus3 | Weighted median | 0.0943 | 0.1210 | 0.4356 | 1.10(0.87-1.39) |
| PTSD | genus | Coprococcus3 | IVW | 0.0635 | 0.0897 | 0.4789 | 1.07(0.89-1.27) |
| PTSD | genus | Coprococcus3 | Simple mode | 0.1581 | 0.1834 | 0.4111 | 1.17(0.82-1.68) |
| PTSD | genus | Coprococcus3 | Weighted mode | 0.1133 | 0.1829 | 0.5509 | 1.12(0.78-1.60) |
| PTSD | genus | DefluviitaleaceaeUCG011 | MR Egger | 0.2236 | 0.2304 | 0.3643 | 1.25(0.80-1.96) |
| PTSD | genus | DefluviitaleaceaeUCG011 | Weighted median | 0.0847 | 0.0863 | 0.3264 | 1.09(0.92-1.29) |
| PTSD | genus | DefluviitaleaceaeUCG011 | IVW | 0.0819 | 0.0667 | 0.2195 | 1.09(0.95-1.24) |
| PTSD | genus | DefluviitaleaceaeUCG011 | Simple mode | 0.0861 | 0.1295 | 0.5247 | 1.09(0.85-1.40) |
| PTSD | genus | DefluviitaleaceaeUCG011 | Weighted mode | 0.0824 | 0.1382 | 0.5675 | 1.09(0.83-1.42) |
| PTSD | genus | Desulfovibrio | MR Egger | -0.1528 | 0.2237 | 0.5139 | 0.86(0.55-1.33) |
| PTSD | genus | Desulfovibrio | Weighted median | 0.0109 | 0.0927 | 0.9063 | 1.01(0.84-1.21) |
| PTSD | genus | Desulfovibrio | IVW | 0.0137 | 0.0704 | 0.8461 | 1.01(0.88-1.16) |
| PTSD | genus | Desulfovibrio | Simple mode | -0.0887 | 0.1748 | 0.6241 | 0.92(0.65-1.29) |
| PTSD | genus | Desulfovibrio | Weighted mode | -0.1061 | 0.1665 | 0.5397 | 0.90(0.65-1.25) |
| PTSD | genus | Dialister | MR Egger | 0.0520 | 0.3572 | 0.8875 | 1.05(0.52-2.12) |
| PTSD | genus | Dialister | Weighted median | -0.0834 | 0.1055 | 0.4296 | 0.92(0.75-1.13) |
| PTSD | genus | Dialister | IVW | -0.0866 | 0.0856 | 0.3114 | 0.92(0.78-1.08) |
| PTSD | genus | Dialister | Simple mode | -0.2554 | 0.1931 | 0.2154 | 0.77(0.53-1.13) |
| PTSD | genus | Dialister | Weighted mode | -0.1642 | 0.1744 | 0.3689 | 0.85(0.60-1.19) |
| PTSD | genus | Dorea | MR Egger | 0.0559 | 0.2476 | 0.8270 | 1.06(0.65-1.72) |
| PTSD | genus | Dorea | Weighted median | -0.2678 | 0.1218 | 0.0279 | 0.77(0.60-0.97) |
| PTSD | genus | Dorea | IVW | -0.2389 | 0.0902 | 0.0081 | 0.79(0.66-0.94) |
| PTSD | genus | Dorea | Simple mode | -0.2855 | 0.2098 | 0.2067 | 0.75(0.50-1.13) |
| PTSD | genus | Dorea | Weighted mode | -0.2797 | 0.2143 | 0.2243 | 0.76(0.50-1.15) |
| PTSD | genus | Eggerthella | MR Egger | -0.3624 | 0.2978 | 0.2546 | 0.70(0.39-1.25) |
| PTSD | genus | Eggerthella | Weighted median | -0.0564 | 0.0732 | 0.4408 | 0.95(0.82-1.09) |
| PTSD | genus | Eggerthella | IVW | 0.0062 | 0.0640 | 0.9222 | 1.01(0.89-1.14) |
| PTSD | genus | Eggerthella | Simple mode | -0.0728 | 0.1132 | 0.5350 | 0.93(0.74-1.16) |
| PTSD | genus | Eggerthella | Weighted mode | -0.0789 | 0.1052 | 0.4704 | 0.92(0.75-1.14) |
| PTSD | genus | Eisenbergiella | MR Egger | -0.4390 | 0.4056 | 0.3073 | 0.64(0.29-1.43) |
| PTSD | genus | Eisenbergiella | Weighted median | 0.0290 | 0.0714 | 0.6849 | 1.03(0.89-1.18) |
| PTSD | genus | Eisenbergiella | IVW | 0.0298 | 0.0520 | 0.5667 | 1.03(0.93-1.14) |
| PTSD | genus | Eisenbergiella | Simple mode | 0.0665 | 0.1198 | 0.5909 | 1.07(0.85-1.35) |
| PTSD | genus | Eisenbergiella | Weighted mode | 0.0573 | 0.1212 | 0.6468 | 1.06(0.84-1.34) |
| PTSD | genus | Enterorhabdus | MR Egger | 0.0762 | 0.1987 | 0.7208 | 1.08(0.73-1.59) |
| PTSD | genus | Enterorhabdus | Weighted median | 0.0429 | 0.0948 | 0.6513 | 1.04(0.87-1.26) |
| PTSD | genus | Enterorhabdus | IVW | 0.0554 | 0.0744 | 0.4561 | 1.06(0.91-1.22) |
| PTSD | genus | Enterorhabdus | Simple mode | 0.1450 | 0.1459 | 0.3659 | 1.16(0.87-1.54) |
| PTSD | genus | Enterorhabdus | Weighted mode | 0.0023 | 0.1260 | 0.9861 | 1.00(0.78-1.28) |
| PTSD | genus | Erysipelatoclostridium | MR Egger | 0.2659 | 0.2215 | 0.2512 | 1.30(0.85-2.01) |
| PTSD | genus | Erysipelatoclostridium | Weighted median | 0.0386 | 0.0812 | 0.6346 | 1.04(0.89-1.22) |
| PTSD | genus | Erysipelatoclostridium | IVW | 0.0600 | 0.0565 | 0.2881 | 1.06(0.95-1.19) |
| PTSD | genus | Erysipelatoclostridium | Simple mode | 0.0365 | 0.1352 | 0.7914 | 1.04(0.80-1.35) |
| PTSD | genus | Erysipelatoclostridium | Weighted mode | 0.0365 | 0.1322 | 0.7868 | 1.04(0.80-1.34) |
| PTSD | genus | ErysipelotrichaceaeUCG003 | MR Egger | 0.1538 | 0.2048 | 0.4642 | 1.17(0.78-1.74) |
| PTSD | genus | ErysipelotrichaceaeUCG003 | Weighted median | 0.1544 | 0.0872 | 0.0768 | 1.17(0.98-1.38) |
| PTSD | genus | ErysipelotrichaceaeUCG003 | IVW | 0.0632 | 0.0728 | 0.3858 | 1.07(0.92-1.23) |
| PTSD | genus | ErysipelotrichaceaeUCG003 | Simple mode | 0.1652 | 0.1391 | 0.2523 | 1.18(0.90-1.55) |
| PTSD | genus | ErysipelotrichaceaeUCG003 | Weighted mode | 0.1684 | 0.1271 | 0.2036 | 1.18(0.92-1.52) |
| PTSD | genus | Escherichia | MR Egger | -0.0397 | 0.2263 | 0.8650 | 0.96(0.62-1.50) |
| PTSD | genus | Escherichia | Weighted median | -0.0871 | 0.1005 | 0.3861 | 0.92(0.75-1.12) |
| PTSD | genus | Escherichia | IVW | -0.0815 | 0.0775 | 0.2929 | 0.92(0.79-1.07) |
| PTSD | genus | Escherichia | Simple mode | -0.0930 | 0.1503 | 0.5513 | 0.91(0.68-1.22) |
| PTSD | genus | Escherichia | Weighted mode | -0.0991 | 0.1504 | 0.5263 | 0.91(0.67-1.22) |
| PTSD | genus | Faecalibacterium | MR Egger | 0.0779 | 0.1525 | 0.6234 | 1.08(0.80-1.46) |
| PTSD | genus | Faecalibacterium | Weighted median | -0.0171 | 0.1036 | 0.8689 | 0.98(0.80-1.20) |
| PTSD | genus | Faecalibacterium | IVW | -0.0819 | 0.0779 | 0.2931 | 0.92(0.79-1.07) |
| PTSD | genus | Faecalibacterium | Simple mode | -0.0254 | 0.1415 | 0.8617 | 0.97(0.74-1.29) |
| PTSD | genus | Faecalibacterium | Weighted mode | -0.0134 | 0.1196 | 0.9129 | 0.99(0.78-1.25) |
| PTSD | genus | FamilyXIIIAD3011group | MR Egger | 0.1587 | 0.4392 | 0.7247 | 1.17(0.50-2.77) |
| PTSD | genus | FamilyXIIIAD3011group | Weighted median | 0.0336 | 0.1102 | 0.7607 | 1.03(0.83-1.28) |
| PTSD | genus | FamilyXIIIAD3011group | IVW | -0.0046 | 0.0838 | 0.9560 | 1.00(0.84-1.17) |
| PTSD | genus | FamilyXIIIAD3011group | Simple mode | -0.2497 | 0.2112 | 0.2600 | 0.78(0.52-1.18) |
| PTSD | genus | FamilyXIIIAD3011group | Weighted mode | -0.2531 | 0.2091 | 0.2493 | 0.78(0.52-1.17) |
| PTSD | genus | FamilyXIIIUCG001 | MR Egger | -0.4049 | 0.2810 | 0.1997 | 0.67(0.38-1.16) |
| PTSD | genus | FamilyXIIIUCG001 | Weighted median | -0.1464 | 0.1303 | 0.2611 | 0.86(0.67-1.12) |
| PTSD | genus | FamilyXIIIUCG001 | IVW | 0.0044 | 0.1129 | 0.9692 | 1.00(0.80-1.25) |
| PTSD | genus | FamilyXIIIUCG001 | Simple mode | -0.1842 | 0.2156 | 0.4213 | 0.83(0.55-1.27) |
| PTSD | genus | FamilyXIIIUCG001 | Weighted mode | -0.2112 | 0.1800 | 0.2791 | 0.81(0.57-1.15) |
| PTSD | genus | Flavonifractor | MR Egger | -0.0355 | 0.4388 | 0.9406 | 0.97(0.41-2.28) |
| PTSD | genus | Flavonifractor | Weighted median | 0.1231 | 0.1250 | 0.3248 | 1.13(0.89-1.45) |
| PTSD | genus | Flavonifractor | IVW | 0.1091 | 0.1020 | 0.2845 | 1.12(0.91-1.36) |
| PTSD | genus | Flavonifractor | Simple mode | 0.1693 | 0.1679 | 0.3702 | 1.18(0.85-1.65) |
| PTSD | genus | Flavonifractor | Weighted mode | 0.1576 | 0.1742 | 0.4170 | 1.17(0.83-1.65) |
| PTSD | genus | Fusicatenibacter | MR Egger | 0.1332 | 0.2732 | 0.6320 | 1.14(0.67-1.95) |
| PTSD | genus | Fusicatenibacter | Weighted median | -0.1500 | 0.0923 | 0.1041 | 0.86(0.72-1.03) |
| PTSD | genus | Fusicatenibacter | IVW | -0.1169 | 0.0686 | 0.0884 | 0.89(0.78-1.02) |
| PTSD | genus | Fusicatenibacter | Simple mode | -0.1647 | 0.1791 | 0.3700 | 0.85(0.60-1.20) |
| PTSD | genus | Fusicatenibacter | Weighted mode | -0.1647 | 0.1702 | 0.3460 | 0.85(0.61-1.18) |
| PTSD | genus | Gordonibacter | MR Egger | -0.3328 | 0.1715 | 0.0811 | 0.72(0.51-1.00) |
| PTSD | genus | Gordonibacter | Weighted median | -0.1003 | 0.0565 | 0.0759 | 0.90(0.81-1.01) |
| PTSD | genus | Gordonibacter | IVW | -0.0890 | 0.0398 | 0.0252 | 0.91(0.85-0.99) |
| PTSD | genus | Gordonibacter | Simple mode | -0.1068 | 0.0904 | 0.2622 | 0.90(0.75-1.07) |
| PTSD | genus | Gordonibacter | Weighted mode | -0.1055 | 0.0903 | 0.2676 | 0.90(0.75-1.07) |
| PTSD | genus | Haemophilus | MR Egger | -0.1848 | 0.1503 | 0.2586 | 0.83(0.62-1.12) |
| PTSD | genus | Haemophilus | Weighted median | -0.0528 | 0.0854 | 0.5364 | 0.95(0.80-1.12) |
| PTSD | genus | Haemophilus | IVW | -0.0668 | 0.0639 | 0.2961 | 0.94(0.83-1.06) |
| PTSD | genus | Haemophilus | Simple mode | -0.0621 | 0.1316 | 0.6496 | 0.94(0.73-1.22) |
| PTSD | genus | Haemophilus | Weighted mode | -0.0551 | 0.1142 | 0.6422 | 0.95(0.76-1.18) |
| PTSD | genus | Holdemanella | MR Egger | -0.0571 | 0.1663 | 0.7393 | 0.94(0.68-1.31) |
| PTSD | genus | Holdemanella | Weighted median | -0.0114 | 0.0749 | 0.8788 | 0.99(0.85-1.14) |
| PTSD | genus | Holdemanella | IVW | -0.0016 | 0.0563 | 0.9777 | 1.00(0.89-1.11) |
| PTSD | genus | Holdemanella | Simple mode | -0.0338 | 0.1157 | 0.7762 | 0.97(0.77-1.21) |
| PTSD | genus | Holdemanella | Weighted mode | -0.0268 | 0.1031 | 0.7998 | 0.97(0.80-1.19) |
| PTSD | genus | Holdemania | MR Egger | 0.0641 | 0.1522 | 0.6804 | 1.07(0.79-1.44) |
| PTSD | genus | Holdemania | Weighted median | 0.0596 | 0.0677 | 0.3784 | 1.06(0.93-1.21) |
| PTSD | genus | Holdemania | IVW | 0.0549 | 0.0528 | 0.2987 | 1.06(0.95-1.17) |
| PTSD | genus | Holdemania | Simple mode | 0.0369 | 0.1165 | 0.7564 | 1.04(0.83-1.30) |
| PTSD | genus | Holdemania | Weighted mode | 0.0764 | 0.1154 | 0.5188 | 1.08(0.86-1.35) |
| PTSD | genus | Howardella | MR Egger | -0.0250 | 0.1978 | 0.9025 | 0.98(0.66-1.44) |
| PTSD | genus | Howardella | Weighted median | 0.0826 | 0.0617 | 0.1803 | 1.09(0.96-1.23) |
| PTSD | genus | Howardella | IVW | 0.0592 | 0.0443 | 0.1814 | 1.06(0.97-1.16) |
| PTSD | genus | Howardella | Simple mode | 0.0816 | 0.0891 | 0.3839 | 1.08(0.91-1.29) |
| PTSD | genus | Howardella | Weighted mode | 0.0776 | 0.0921 | 0.4213 | 1.08(0.90-1.29) |
| PTSD | genus | Hungatella | MR Egger | 0.2258 | 0.4159 | 0.6248 | 1.25(0.55-2.83) |
| PTSD | genus | Hungatella | Weighted median | 0.0938 | 0.0836 | 0.2616 | 1.10(0.93-1.29) |
| PTSD | genus | Hungatella | IVW | 0.1062 | 0.0677 | 0.1170 | 1.11(0.97-1.27) |
| PTSD | genus | Hungatella | Simple mode | 0.1058 | 0.1078 | 0.3820 | 1.11(0.90-1.37) |
| PTSD | genus | Hungatella | Weighted mode | 0.1033 | 0.1114 | 0.4062 | 1.11(0.89-1.38) |
| PTSD | genus | Intestinibacter | MR Egger | -0.0976 | 0.2211 | 0.6662 | 0.91(0.59-1.40) |
| PTSD | genus | Intestinibacter | Weighted median | 0.0721 | 0.0927 | 0.4365 | 1.07(0.90-1.29) |
| PTSD | genus | Intestinibacter | IVW | 0.1076 | 0.0669 | 0.1078 | 1.11(0.98-1.27) |
| PTSD | genus | Intestinibacter | Simple mode | 0.0242 | 0.1564 | 0.8793 | 1.02(0.75-1.39) |
| PTSD | genus | Intestinibacter | Weighted mode | 0.0084 | 0.1472 | 0.9551 | 1.01(0.76-1.35) |
| PTSD | genus | Intestinimonas | MR Egger | -0.0036 | 0.1526 | 0.9815 | 1.00(0.74-1.34) |
| PTSD | genus | Intestinimonas | Weighted median | -0.0383 | 0.0781 | 0.6238 | 0.96(0.83-1.12) |
| PTSD | genus | Intestinimonas | IVW | -0.0494 | 0.0585 | 0.3985 | 0.95(0.85-1.07) |
| PTSD | genus | Intestinimonas | Simple mode | -0.0415 | 0.1328 | 0.7584 | 0.96(0.74-1.24) |
| PTSD | genus | Intestinimonas | Weighted mode | -0.0340 | 0.1185 | 0.7781 | 0.97(0.77-1.22) |
| PTSD | genus | Lachnoclostridium | MR Egger | -0.1286 | 0.3511 | 0.7212 | 0.88(0.44-1.75) |
| PTSD | genus | Lachnoclostridium | Weighted median | 0.0331 | 0.1250 | 0.7914 | 1.03(0.81-1.32) |
| PTSD | genus | Lachnoclostridium | IVW | 0.1013 | 0.0958 | 0.2899 | 1.11(0.92-1.34) |
| PTSD | genus | Lachnoclostridium | Simple mode | -0.0704 | 0.2214 | 0.7559 | 0.93(0.60-1.44) |
| PTSD | genus | Lachnoclostridium | Weighted mode | -0.0506 | 0.2375 | 0.8349 | 0.95(0.60-1.51) |
| PTSD | genus | Lachnospira | MR Egger | 0.2567 | 0.7150 | 0.7378 | 1.29(0.32-5.25) |
| PTSD | genus | Lachnospira | Weighted median | -0.2355 | 0.1595 | 0.1400 | 0.79(0.58-1.08) |
| PTSD | genus | Lachnospira | IVW | -0.1763 | 0.1190 | 0.1384 | 0.84(0.66-1.06) |
| PTSD | genus | Lachnospira | Simple mode | -0.3256 | 0.2609 | 0.2673 | 0.72(0.43-1.20) |
| PTSD | genus | Lachnospira | Weighted mode | -0.3320 | 0.2460 | 0.2351 | 0.72(0.44-1.16) |
| PTSD | genus | LachnospiraceaeFCS020group | MR Egger | 0.0898 | 0.1831 | 0.6333 | 1.09(0.76-1.57) |
| PTSD | genus | LachnospiraceaeFCS020group | Weighted median | 0.0145 | 0.0925 | 0.8756 | 1.01(0.85-1.22) |
| PTSD | genus | LachnospiraceaeFCS020group | IVW | 0.0293 | 0.0689 | 0.6702 | 1.03(0.90-1.18) |
| PTSD | genus | LachnospiraceaeFCS020group | Simple mode | 0.0497 | 0.1582 | 0.7590 | 1.05(0.77-1.43) |
| PTSD | genus | LachnospiraceaeFCS020group | Weighted mode | 0.0187 | 0.1406 | 0.8964 | 1.02(0.77-1.34) |
| PTSD | genus | LachnospiraceaeNC2004group | MR Egger | -0.0383 | 0.2487 | 0.8820 | 0.96(0.59-1.57) |
| PTSD | genus | LachnospiraceaeNC2004group | Weighted median | 0.0407 | 0.0758 | 0.5913 | 1.04(0.90-1.21) |
| PTSD | genus | LachnospiraceaeNC2004group | IVW | 0.0259 | 0.0598 | 0.6646 | 1.03(0.91-1.15) |
| PTSD | genus | LachnospiraceaeNC2004group | Simple mode | 0.0565 | 0.1281 | 0.6707 | 1.06(0.82-1.36) |
| PTSD | genus | LachnospiraceaeNC2004group | Weighted mode | 0.0400 | 0.1229 | 0.7532 | 1.04(0.82-1.32) |
| PTSD | genus | LachnospiraceaeND3007group | MR Egger | -3.8255 | 2.8694 | 0.4097 | 0.02(0.00-6.04) |
| PTSD | genus | LachnospiraceaeND3007group | Weighted median | 0.1215 | 0.2214 | 0.5833 | 1.13(0.73-1.74) |
| PTSD | genus | LachnospiraceaeND3007group | IVW | 0.1216 | 0.2105 | 0.5634 | 1.13(0.75-1.71) |
| PTSD | genus | LachnospiraceaeND3007group | Simple mode | 0.1916 | 0.2951 | 0.5827 | 1.21(0.68-2.16) |
| PTSD | genus | LachnospiraceaeND3007group | Weighted mode | 0.1455 | 0.3070 | 0.6823 | 1.16(0.63-2.11) |
| PTSD | genus | LachnospiraceaeNK4A136group | MR Egger | -0.2420 | 0.1666 | 0.1702 | 0.79(0.57-1.09) |
| PTSD | genus | LachnospiraceaeNK4A136group | Weighted median | -0.1875 | 0.0989 | 0.0579 | 0.83(0.68-1.01) |
| PTSD | genus | LachnospiraceaeNK4A136group | IVW | -0.1085 | 0.0800 | 0.1748 | 0.90(0.77-1.05) |
| PTSD | genus | LachnospiraceaeNK4A136group | Simple mode | -0.1216 | 0.1685 | 0.4825 | 0.89(0.64-1.23) |
| PTSD | genus | LachnospiraceaeNK4A136group | Weighted mode | -0.1881 | 0.1114 | 0.1133 | 0.83(0.67-1.03) |
| PTSD | genus | LachnospiraceaeUCG001 | MR Egger | 0.1738 | 0.2977 | 0.5723 | 1.19(0.66-2.13) |
| PTSD | genus | LachnospiraceaeUCG001 | Weighted median | -0.0679 | 0.0877 | 0.4388 | 0.93(0.79-1.11) |
| PTSD | genus | LachnospiraceaeUCG001 | IVW | -0.0888 | 0.0646 | 0.1695 | 0.92(0.81-1.04) |
| PTSD | genus | LachnospiraceaeUCG001 | Simple mode | -0.1135 | 0.1501 | 0.4652 | 0.89(0.67-1.20) |
| PTSD | genus | LachnospiraceaeUCG001 | Weighted mode | -0.0809 | 0.1465 | 0.5918 | 0.92(0.69-1.23) |
| PTSD | genus | LachnospiraceaeUCG004 | MR Egger | -0.1284 | 0.3689 | 0.7339 | 0.88(0.43-1.81) |
| PTSD | genus | LachnospiraceaeUCG004 | Weighted median | 0.1520 | 0.1051 | 0.1482 | 1.16(0.95-1.43) |
| PTSD | genus | LachnospiraceaeUCG004 | IVW | 0.1633 | 0.0906 | 0.0716 | 1.18(0.99-1.41) |
| PTSD | genus | LachnospiraceaeUCG004 | Simple mode | 0.2916 | 0.2155 | 0.1991 | 1.34(0.88-2.04) |
| PTSD | genus | LachnospiraceaeUCG004 | Weighted mode | 0.2342 | 0.2014 | 0.2657 | 1.26(0.85-1.88) |
| PTSD | genus | LachnospiraceaeUCG008 | MR Egger | 0.2442 | 0.2942 | 0.4279 | 1.28(0.72-2.27) |
| PTSD | genus | LachnospiraceaeUCG008 | Weighted median | 0.0039 | 0.0709 | 0.9561 | 1.00(0.87-1.15) |
| PTSD | genus | LachnospiraceaeUCG008 | IVW | 0.0239 | 0.0556 | 0.6671 | 1.02(0.92-1.14) |
| PTSD | genus | LachnospiraceaeUCG008 | Simple mode | -0.0297 | 0.1165 | 0.8038 | 0.97(0.77-1.22) |
| PTSD | genus | LachnospiraceaeUCG008 | Weighted mode | -0.0217 | 0.1162 | 0.8556 | 0.98(0.78-1.23) |
| PTSD | genus | LachnospiraceaeUCG010 | MR Egger | 0.0839 | 0.2714 | 0.7652 | 1.09(0.64-1.85) |
| PTSD | genus | LachnospiraceaeUCG010 | Weighted median | -0.0146 | 0.1006 | 0.8850 | 0.99(0.81-1.20) |
| PTSD | genus | LachnospiraceaeUCG010 | IVW | -0.0104 | 0.0983 | 0.9155 | 0.99(0.82-1.20) |
| PTSD | genus | LachnospiraceaeUCG010 | Simple mode | -0.0652 | 0.1453 | 0.6643 | 0.94(0.70-1.25) |
| PTSD | genus | LachnospiraceaeUCG010 | Weighted mode | -0.0422 | 0.1412 | 0.7719 | 0.96(0.73-1.26) |
| PTSD | genus | Lactobacillus | MR Egger | -0.1508 | 0.1879 | 0.4453 | 0.86(0.60-1.24) |
| PTSD | genus | Lactobacillus | Weighted median | -0.0354 | 0.0779 | 0.6494 | 0.97(0.83-1.12) |
| PTSD | genus | Lactobacillus | IVW | 0.0137 | 0.0616 | 0.8235 | 1.01(0.90-1.14) |
| PTSD | genus | Lactobacillus | Simple mode | -0.0841 | 0.1254 | 0.5192 | 0.92(0.72-1.18) |
| PTSD | genus | Lactobacillus | Weighted mode | -0.0771 | 0.1227 | 0.5457 | 0.93(0.73-1.18) |
| PTSD | genus | Lactococcus | MR Egger | 0.0837 | 0.2560 | 0.7532 | 1.09(0.66-1.80) |
| PTSD | genus | Lactococcus | Weighted median | 0.0101 | 0.0687 | 0.8833 | 1.01(0.88-1.16) |
| PTSD | genus | Lactococcus | IVW | 0.0126 | 0.0547 | 0.8180 | 1.01(0.91-1.13) |
| PTSD | genus | Lactococcus | Simple mode | -0.0168 | 0.1098 | 0.8821 | 0.98(0.79-1.22) |
| PTSD | genus | Lactococcus | Weighted mode | -0.0134 | 0.1041 | 0.9008 | 0.99(0.80-1.21) |
| PTSD | genus | Marvinbryantia | MR Egger | 0.6246 | 0.2997 | 0.0707 | 1.87(1.04-3.36) |
| PTSD | genus | Marvinbryantia | Weighted median | -0.0572 | 0.1024 | 0.5767 | 0.94(0.77-1.15) |
| PTSD | genus | Marvinbryantia | IVW | 0.0327 | 0.0888 | 0.7131 | 1.03(0.87-1.23) |
| PTSD | genus | Marvinbryantia | Simple mode | -0.1256 | 0.1600 | 0.4527 | 0.88(0.64-1.21) |
| PTSD | genus | Marvinbryantia | Weighted mode | -0.1256 | 0.1661 | 0.4690 | 0.88(0.64-1.22) |
| PTSD | genus | Methanobrevibacter | MR Egger | -0.0662 | 0.2031 | 0.7608 | 0.94(0.63-1.39) |
| PTSD | genus | Methanobrevibacter | Weighted median | 0.0574 | 0.0648 | 0.3754 | 1.06(0.93-1.20) |
| PTSD | genus | Methanobrevibacter | IVW | 0.0498 | 0.0554 | 0.3686 | 1.05(0.94-1.17) |
| PTSD | genus | Methanobrevibacter | Simple mode | 0.1048 | 0.0968 | 0.3284 | 1.11(0.92-1.34) |
| PTSD | genus | Methanobrevibacter | Weighted mode | 0.1019 | 0.1024 | 0.3654 | 1.11(0.91-1.35) |
| PTSD | genus | Odoribacter | MR Egger | -0.0275 | 0.3335 | 0.9376 | 0.97(0.51-1.87) |
| PTSD | genus | Odoribacter | Weighted median | 0.0023 | 0.1246 | 0.9855 | 1.00(0.79-1.28) |
| PTSD | genus | Odoribacter | IVW | 0.0075 | 0.1027 | 0.9420 | 1.01(0.82-1.23) |
| PTSD | genus | Odoribacter | Simple mode | -0.0508 | 0.1846 | 0.7926 | 0.95(0.66-1.36) |
| PTSD | genus | Odoribacter | Weighted mode | -0.0620 | 0.1756 | 0.7361 | 0.94(0.67-1.33) |
| PTSD | genus | Olsenella | MR Egger | -0.0589 | 0.1786 | 0.7493 | 0.94(0.66-1.34) |
| PTSD | genus | Olsenella | Weighted median | 0.0378 | 0.0588 | 0.5198 | 1.04(0.93-1.17) |
| PTSD | genus | Olsenella | IVW | 0.0686 | 0.0452 | 0.1291 | 1.07(0.98-1.17) |
| PTSD | genus | Olsenella | Simple mode | 0.0043 | 0.1022 | 0.9676 | 1.00(0.82-1.23) |
| PTSD | genus | Olsenella | Weighted mode | -0.0070 | 0.0912 | 0.9400 | 0.99(0.83-1.19) |
| PTSD | genus | Oscillibacter | MR Egger | -0.1741 | 0.2093 | 0.4217 | 0.84(0.56-1.27) |
| PTSD | genus | Oscillibacter | Weighted median | -0.0635 | 0.0761 | 0.4042 | 0.94(0.81-1.09) |
| PTSD | genus | Oscillibacter | IVW | -0.0193 | 0.0554 | 0.7277 | 0.98(0.88-1.09) |
| PTSD | genus | Oscillibacter | Simple mode | -0.0977 | 0.1282 | 0.4596 | 0.91(0.71-1.17) |
| PTSD | genus | Oscillibacter | Weighted mode | -0.1000 | 0.1256 | 0.4402 | 0.90(0.71-1.16) |
| PTSD | genus | Oscillospira | MR Egger | -0.3708 | 0.3559 | 0.3377 | 0.69(0.34-1.39) |
| PTSD | genus | Oscillospira | Weighted median | -0.0025 | 0.1068 | 0.9815 | 1.00(0.81-1.23) |
| PTSD | genus | Oscillospira | IVW | 0.0436 | 0.0900 | 0.6279 | 1.04(0.88-1.25) |
| PTSD | genus | Oscillospira | Simple mode | -0.0061 | 0.1653 | 0.9714 | 0.99(0.72-1.37) |
| PTSD | genus | Oscillospira | Weighted mode | -0.0123 | 0.1447 | 0.9344 | 0.99(0.74-1.31) |
| PTSD | genus | Oxalobacter | MR Egger | -0.0460 | 0.2264 | 0.8434 | 0.96(0.61-1.49) |
| PTSD | genus | Oxalobacter | Weighted median | -0.0215 | 0.0636 | 0.7358 | 0.98(0.86-1.11) |
| PTSD | genus | Oxalobacter | IVW | -0.0303 | 0.0463 | 0.5138 | 0.97(0.89-1.06) |
| PTSD | genus | Oxalobacter | Simple mode | -0.0053 | 0.1012 | 0.9590 | 0.99(0.82-1.21) |
| PTSD | genus | Oxalobacter | Weighted mode | -0.0113 | 0.0927 | 0.9056 | 0.99(0.82-1.19) |
| PTSD | genus | Parabacteroides | MR Egger | 0.2376 | 0.3347 | 0.5169 | 1.27(0.66-2.44) |
| PTSD | genus | Parabacteroides | Weighted median | 0.0923 | 0.1296 | 0.4764 | 1.10(0.85-1.41) |
| PTSD | genus | Parabacteroides | IVW | 0.0768 | 0.1084 | 0.4788 | 1.08(0.87-1.34) |
| PTSD | genus | Parabacteroides | Simple mode | 0.0951 | 0.1857 | 0.6304 | 1.10(0.76-1.58) |
| PTSD | genus | Parabacteroides | Weighted mode | 0.1302 | 0.1761 | 0.4929 | 1.14(0.81-1.61) |
| PTSD | genus | Paraprevotella | MR Egger | 0.2131 | 0.1748 | 0.2484 | 1.24(0.88-1.74) |
| PTSD | genus | Paraprevotella | Weighted median | 0.1133 | 0.0701 | 0.1059 | 1.12(0.98-1.28) |
| PTSD | genus | Paraprevotella | IVW | 0.0848 | 0.0510 | 0.0964 | 1.09(0.98-1.20) |
| PTSD | genus | Paraprevotella | Simple mode | 0.1632 | 0.1153 | 0.1824 | 1.18(0.94-1.48) |
| PTSD | genus | Paraprevotella | Weighted mode | 0.1488 | 0.1118 | 0.2080 | 1.16(0.93-1.44) |
| PTSD | genus | Parasutterella | MR Egger | 0.0218 | 0.1774 | 0.9039 | 1.02(0.72-1.45) |
| PTSD | genus | Parasutterella | Weighted median | -0.0883 | 0.0790 | 0.2637 | 0.92(0.78-1.07) |
| PTSD | genus | Parasutterella | IVW | -0.0778 | 0.0589 | 0.1868 | 0.93(0.82-1.04) |
| PTSD | genus | Parasutterella | Simple mode | -0.1473 | 0.1384 | 0.3053 | 0.86(0.66-1.13) |
| PTSD | genus | Parasutterella | Weighted mode | -0.0889 | 0.1406 | 0.5373 | 0.91(0.69-1.21) |
| PTSD | genus | Peptococcus | MR Egger | -0.0661 | 0.2735 | 0.8140 | 0.94(0.55-1.60) |
| PTSD | genus | Peptococcus | Weighted median | -0.0097 | 0.0746 | 0.8971 | 0.99(0.86-1.15) |
| PTSD | genus | Peptococcus | IVW | 0.0414 | 0.0686 | 0.5463 | 1.04(0.91-1.19) |
| PTSD | genus | Peptococcus | Simple mode | -0.0308 | 0.1425 | 0.8331 | 0.97(0.73-1.28) |
| PTSD | genus | Peptococcus | Weighted mode | -0.0308 | 0.1479 | 0.8391 | 0.97(0.73-1.30) |
| PTSD | genus | Phascolarctobacterium | MR Egger | 0.1767 | 0.3777 | 0.6511 | 1.19(0.57-2.50) |
| PTSD | genus | Phascolarctobacterium | Weighted median | 0.0615 | 0.1021 | 0.5469 | 1.06(0.87-1.30) |
| PTSD | genus | Phascolarctobacterium | IVW | 0.1708 | 0.0799 | 0.0324 | 1.19(1.01-1.39) |
| PTSD | genus | Phascolarctobacterium | Simple mode | -0.0103 | 0.1961 | 0.9590 | 0.99(0.67-1.45) |
| PTSD | genus | Phascolarctobacterium | Weighted mode | -0.0103 | 0.1793 | 0.9552 | 0.99(0.70-1.41) |
| PTSD | genus | Prevotella7 | MR Egger | -0.2487 | 0.2985 | 0.4264 | 0.78(0.43-1.40) |
| PTSD | genus | Prevotella7 | Weighted median | 0.0347 | 0.0622 | 0.5775 | 1.04(0.92-1.17) |
| PTSD | genus | Prevotella7 | IVW | -0.0385 | 0.0476 | 0.4185 | 0.96(0.88-1.06) |
| PTSD | genus | Prevotella7 | Simple mode | 0.0533 | 0.1059 | 0.6260 | 1.05(0.86-1.30) |
| PTSD | genus | Prevotella7 | Weighted mode | 0.0550 | 0.1095 | 0.6261 | 1.06(0.85-1.31) |
| PTSD | genus | Prevotella9 | MR Egger | 0.0025 | 0.1841 | 0.9894 | 1.00(0.70-1.44) |
| PTSD | genus | Prevotella9 | Weighted median | 0.0898 | 0.0813 | 0.2694 | 1.09(0.93-1.28) |
| PTSD | genus | Prevotella9 | IVW | 0.1064 | 0.0585 | 0.0689 | 1.11(0.99-1.25) |
| PTSD | genus | Prevotella9 | Simple mode | 0.0725 | 0.1445 | 0.6238 | 1.08(0.81-1.43) |
| PTSD | genus | Prevotella9 | Weighted mode | 0.0935 | 0.1269 | 0.4735 | 1.10(0.86-1.41) |
| PTSD | genus | RikenellaceaeRC9gutgroup | MR Egger | 0.0453 | 0.2497 | 0.8601 | 1.05(0.64-1.71) |
| PTSD | genus | RikenellaceaeRC9gutgroup | Weighted median | -0.0166 | 0.0533 | 0.7547 | 0.98(0.89-1.09) |
| PTSD | genus | RikenellaceaeRC9gutgroup | IVW | -0.0425 | 0.0403 | 0.2906 | 0.96(0.89-1.04) |
| PTSD | genus | RikenellaceaeRC9gutgroup | Simple mode | 0.0100 | 0.0842 | 0.9077 | 1.01(0.86-1.19) |
| PTSD | genus | RikenellaceaeRC9gutgroup | Weighted mode | 0.0164 | 0.0795 | 0.8404 | 1.02(0.87-1.19) |
| PTSD | genus | Romboutsia | MR Egger | -0.2566 | 0.1713 | 0.1601 | 0.77(0.55-1.08) |
| PTSD | genus | Romboutsia | Weighted median | -0.0715 | 0.0917 | 0.4351 | 0.93(0.78-1.11) |
| PTSD | genus | Romboutsia | IVW | -0.0445 | 0.0662 | 0.5014 | 0.96(0.84-1.09) |
| PTSD | genus | Romboutsia | Simple mode | -0.1222 | 0.1611 | 0.4617 | 0.88(0.65-1.21) |
| PTSD | genus | Romboutsia | Weighted mode | -0.1272 | 0.1821 | 0.4971 | 0.88(0.62-1.26) |
| PTSD | genus | Roseburia | MR Egger | 0.2232 | 0.2357 | 0.3622 | 1.25(0.79-1.98) |
| PTSD | genus | Roseburia | Weighted median | 0.0469 | 0.1076 | 0.6631 | 1.05(0.85-1.29) |
| PTSD | genus | Roseburia | IVW | -0.0133 | 0.0809 | 0.8693 | 0.99(0.84-1.16) |
| PTSD | genus | Roseburia | Simple mode | 0.1048 | 0.2146 | 0.6335 | 1.11(0.73-1.69) |
| PTSD | genus | Roseburia | Weighted mode | 0.1372 | 0.1760 | 0.4496 | 1.15(0.81-1.62) |
| PTSD | genus | Ruminiclostridium5 | MR Egger | -0.3479 | 0.3818 | 0.3859 | 0.71(0.33-1.49) |
| PTSD | genus | Ruminiclostridium5 | Weighted median | 0.0564 | 0.1290 | 0.6619 | 1.06(0.82-1.36) |
| PTSD | genus | Ruminiclostridium5 | IVW | 0.0001 | 0.0926 | 0.9994 | 1.00(0.83-1.20) |
| PTSD | genus | Ruminiclostridium5 | Simple mode | 0.1198 | 0.2215 | 0.6003 | 1.13(0.73-1.74) |
| PTSD | genus | Ruminiclostridium5 | Weighted mode | 0.1107 | 0.2077 | 0.6057 | 1.12(0.74-1.68) |
| PTSD | genus | Ruminiclostridium6 | MR Egger | 0.1392 | 0.1652 | 0.4136 | 1.15(0.83-1.59) |
| PTSD | genus | Ruminiclostridium6 | Weighted median | 0.0876 | 0.0889 | 0.3245 | 1.09(0.92-1.30) |
| PTSD | genus | Ruminiclostridium6 | IVW | 0.0962 | 0.0630 | 0.1265 | 1.10(0.97-1.25) |
| PTSD | genus | Ruminiclostridium6 | Simple mode | -0.0761 | 0.1424 | 0.6007 | 0.93(0.70-1.22) |
| PTSD | genus | Ruminiclostridium6 | Weighted mode | 0.0007 | 0.1350 | 0.9960 | 1.00(0.77-1.30) |
| PTSD | genus | Ruminiclostridium9 | MR Egger | 0.3395 | 0.4236 | 0.4492 | 1.40(0.61-3.22) |
| PTSD | genus | Ruminiclostridium9 | Weighted median | 0.0714 | 0.1400 | 0.6104 | 1.07(0.82-1.41) |
| PTSD | genus | Ruminiclostridium9 | IVW | 0.1075 | 0.0980 | 0.2722 | 1.11(0.92-1.35) |
| PTSD | genus | Ruminiclostridium9 | Simple mode | -0.1221 | 0.2199 | 0.5940 | 0.89(0.58-1.36) |
| PTSD | genus | Ruminiclostridium9 | Weighted mode | -0.1152 | 0.2282 | 0.6274 | 0.89(0.57-1.39) |
| PTSD | genus | RuminococcaceaeNK4A214group | MR Egger | -0.0518 | 0.2962 | 0.8641 | 0.95(0.53-1.70) |
| PTSD | genus | RuminococcaceaeNK4A214group | Weighted median | -0.0601 | 0.1065 | 0.5729 | 0.94(0.76-1.16) |
| PTSD | genus | RuminococcaceaeNK4A214group | IVW | -0.0092 | 0.0806 | 0.9094 | 0.99(0.85-1.16) |
| PTSD | genus | RuminococcaceaeNK4A214group | Simple mode | -0.0938 | 0.2011 | 0.6485 | 0.91(0.61-1.35) |
| PTSD | genus | RuminococcaceaeNK4A214group | Weighted mode | -0.0976 | 0.2137 | 0.6555 | 0.91(0.60-1.38) |
| PTSD | genus | RuminococcaceaeUCG002 | MR Egger | -0.1485 | 0.1638 | 0.3753 | 0.86(0.63-1.19) |
| PTSD | genus | RuminococcaceaeUCG002 | Weighted median | -0.0191 | 0.0810 | 0.8139 | 0.98(0.84-1.15) |
| PTSD | genus | RuminococcaceaeUCG002 | IVW | -0.0520 | 0.0589 | 0.3774 | 0.95(0.85-1.07) |
| PTSD | genus | RuminococcaceaeUCG002 | Simple mode | 0.0579 | 0.1565 | 0.7151 | 1.06(0.78-1.44) |
| PTSD | genus | RuminococcaceaeUCG002 | Weighted mode | -0.0184 | 0.1422 | 0.8984 | 0.98(0.74-1.30) |
| PTSD | genus | RuminococcaceaeUCG003 | MR Egger | 0.2231 | 0.2497 | 0.3926 | 1.25(0.77-2.04) |
| PTSD | genus | RuminococcaceaeUCG003 | Weighted median | -0.1035 | 0.1035 | 0.3176 | 0.90(0.74-1.10) |
| PTSD | genus | RuminococcaceaeUCG003 | IVW | -0.0476 | 0.0753 | 0.5270 | 0.95(0.82-1.11) |
| PTSD | genus | RuminococcaceaeUCG003 | Simple mode | -0.1881 | 0.1770 | 0.3105 | 0.83(0.59-1.17) |
| PTSD | genus | RuminococcaceaeUCG003 | Weighted mode | -0.1857 | 0.1942 | 0.3595 | 0.83(0.57-1.22) |
| PTSD | genus | RuminococcaceaeUCG004 | MR Egger | 0.4332 | 0.3763 | 0.2794 | 1.54(0.74-3.22) |
| PTSD | genus | RuminococcaceaeUCG004 | Weighted median | 0.2143 | 0.0916 | 0.0194 | 1.24(1.04-1.48) |
| PTSD | genus | RuminococcaceaeUCG004 | IVW | 0.2033 | 0.0669 | 0.0024 | 1.23(1.07-1.40) |
| PTSD | genus | RuminococcaceaeUCG004 | Simple mode | 0.3572 | 0.1739 | 0.0670 | 1.43(1.02-2.01) |
| PTSD | genus | RuminococcaceaeUCG004 | Weighted mode | 0.3643 | 0.1724 | 0.0607 | 1.44(1.03-2.02) |
| PTSD | genus | RuminococcaceaeUCG005 | MR Egger | -0.0707 | 0.2048 | 0.7360 | 0.93(0.62-1.39) |
| PTSD | genus | RuminococcaceaeUCG005 | Weighted median | 0.0607 | 0.0941 | 0.5188 | 1.06(0.88-1.28) |
| PTSD | genus | RuminococcaceaeUCG005 | IVW | 0.0510 | 0.0714 | 0.4748 | 1.05(0.91-1.21) |
| PTSD | genus | RuminococcaceaeUCG005 | Simple mode | 0.0871 | 0.1418 | 0.5496 | 1.09(0.83-1.44) |
| PTSD | genus | RuminococcaceaeUCG005 | Weighted mode | 0.0621 | 0.1318 | 0.6454 | 1.06(0.82-1.38) |
| PTSD | genus | RuminococcaceaeUCG009 | MR Egger | 0.1606 | 0.2856 | 0.5875 | 1.17(0.67-2.06) |
| PTSD | genus | RuminococcaceaeUCG009 | Weighted median | 0.0754 | 0.0854 | 0.3772 | 1.08(0.91-1.27) |
| PTSD | genus | RuminococcaceaeUCG009 | IVW | 0.0436 | 0.0659 | 0.5082 | 1.04(0.92-1.19) |
| PTSD | genus | RuminococcaceaeUCG009 | Simple mode | 0.1709 | 0.1438 | 0.2621 | 1.19(0.89-1.57) |
| PTSD | genus | RuminococcaceaeUCG009 | Weighted mode | 0.1526 | 0.1542 | 0.3456 | 1.16(0.86-1.58) |
| PTSD | genus | RuminococcaceaeUCG010 | MR Egger | 0.0877 | 0.5228 | 0.8749 | 1.09(0.39-3.04) |
| PTSD | genus | RuminococcaceaeUCG010 | Weighted median | 0.0445 | 0.1298 | 0.7317 | 1.05(0.81-1.35) |
| PTSD | genus | RuminococcaceaeUCG010 | IVW | -0.0474 | 0.1342 | 0.7239 | 0.95(0.73-1.24) |
| PTSD | genus | RuminococcaceaeUCG010 | Simple mode | 0.0464 | 0.1659 | 0.7910 | 1.05(0.76-1.45) |
| PTSD | genus | RuminococcaceaeUCG010 | Weighted mode | 0.0464 | 0.1602 | 0.7838 | 1.05(0.77-1.43) |
| PTSD | genus | RuminococcaceaeUCG011 | MR Egger | 0.1456 | 0.4016 | 0.7293 | 1.16(0.53-2.54) |
| PTSD | genus | RuminococcaceaeUCG011 | Weighted median | 0.0085 | 0.0725 | 0.9063 | 1.01(0.87-1.16) |
| PTSD | genus | RuminococcaceaeUCG011 | IVW | 0.0362 | 0.0728 | 0.6191 | 1.04(0.90-1.20) |
| PTSD | genus | RuminococcaceaeUCG011 | Simple mode | -0.0356 | 0.1237 | 0.7819 | 0.97(0.76-1.23) |
| PTSD | genus | RuminococcaceaeUCG011 | Weighted mode | -0.0164 | 0.1111 | 0.8867 | 0.98(0.79-1.22) |
| PTSD | genus | RuminococcaceaeUCG013 | MR Egger | 0.0359 | 0.2156 | 0.8711 | 1.04(0.68-1.58) |
| PTSD | genus | RuminococcaceaeUCG013 | Weighted median | 0.0344 | 0.1041 | 0.7410 | 1.04(0.84-1.27) |
| PTSD | genus | RuminococcaceaeUCG013 | IVW | 0.0121 | 0.0789 | 0.8783 | 1.01(0.87-1.18) |
| PTSD | genus | RuminococcaceaeUCG013 | Simple mode | 0.0342 | 0.1554 | 0.8296 | 1.03(0.76-1.40) |
| PTSD | genus | RuminococcaceaeUCG013 | Weighted mode | 0.0342 | 0.1467 | 0.8197 | 1.03(0.78-1.38) |
| PTSD | genus | RuminococcaceaeUCG014 | MR Egger | -0.1189 | 0.2722 | 0.6725 | 0.89(0.52-1.51) |
| PTSD | genus | RuminococcaceaeUCG014 | Weighted median | -0.2811 | 0.1077 | 0.0091 | 0.75(0.61-0.93) |
| PTSD | genus | RuminococcaceaeUCG014 | IVW | -0.1592 | 0.0937 | 0.0896 | 0.85(0.71-1.02) |
| PTSD | genus | RuminococcaceaeUCG014 | Simple mode | -0.3195 | 0.1626 | 0.0778 | 0.73(0.53-1.00) |
| PTSD | genus | RuminococcaceaeUCG014 | Weighted mode | -0.3162 | 0.1654 | 0.0850 | 0.73(0.53-1.01) |
| PTSD | genus | Ruminococcus1 | MR Egger | 0.1417 | 0.2293 | 0.5538 | 1.15(0.74-1.81) |
| PTSD | genus | Ruminococcus1 | Weighted median | 0.1072 | 0.1201 | 0.3718 | 1.11(0.88-1.41) |
| PTSD | genus | Ruminococcus1 | IVW | 0.0549 | 0.0867 | 0.5265 | 1.06(0.89-1.25) |
| PTSD | genus | Ruminococcus1 | Simple mode | 0.1590 | 0.1872 | 0.4177 | 1.17(0.81-1.69) |
| PTSD | genus | Ruminococcus1 | Weighted mode | 0.1234 | 0.1699 | 0.4861 | 1.13(0.81-1.58) |
| PTSD | genus | Ruminococcus2 | MR Egger | 0.1318 | 0.2236 | 0.5656 | 1.14(0.74-1.77) |
| PTSD | genus | Ruminococcus2 | Weighted median | 0.0012 | 0.1040 | 0.9910 | 1.00(0.82-1.23) |
| PTSD | genus | Ruminococcus2 | IVW | 0.0206 | 0.0809 | 0.7985 | 1.02(0.87-1.20) |
| PTSD | genus | Ruminococcus2 | Simple mode | -0.0948 | 0.2083 | 0.6559 | 0.91(0.60-1.37) |
| PTSD | genus | Ruminococcus2 | Weighted mode | 0.0183 | 0.1744 | 0.9177 | 1.02(0.72-1.43) |
| PTSD | genus | Sellimonas | MR Egger | -0.1076 | 0.2617 | 0.6934 | 0.90(0.54-1.50) |
| PTSD | genus | Sellimonas | Weighted median | -0.0618 | 0.0595 | 0.2987 | 0.94(0.84-1.06) |
| PTSD | genus | Sellimonas | IVW | -0.0965 | 0.0437 | 0.0272 | 0.91(0.83-0.99) |
| PTSD | genus | Sellimonas | Simple mode | -0.0443 | 0.0993 | 0.6669 | 0.96(0.79-1.16) |
| PTSD | genus | Sellimonas | Weighted mode | -0.0398 | 0.0894 | 0.6679 | 0.96(0.81-1.14) |
| PTSD | genus | Senegalimassilia | MR Egger | 0.1993 | 0.3739 | 0.6310 | 1.22(0.59-2.54) |
| PTSD | genus | Senegalimassilia | Weighted median | -0.0162 | 0.1107 | 0.8840 | 0.98(0.79-1.22) |
| PTSD | genus | Senegalimassilia | IVW | -0.1102 | 0.1059 | 0.2982 | 0.90(0.73-1.10) |
| PTSD | genus | Senegalimassilia | Simple mode | -0.0109 | 0.1239 | 0.9344 | 0.99(0.78-1.26) |
| PTSD | genus | Senegalimassilia | Weighted mode | -0.0050 | 0.1258 | 0.9704 | 1.00(0.78-1.27) |
| PTSD | genus | Slackia | MR Egger | 0.1800 | 0.4215 | 0.6913 | 1.20(0.52-2.73) |
| PTSD | genus | Slackia | Weighted median | -0.0283 | 0.0914 | 0.7571 | 0.97(0.81-1.16) |
| PTSD | genus | Slackia | IVW | -0.0248 | 0.0727 | 0.7332 | 0.98(0.85-1.12) |
| PTSD | genus | Slackia | Simple mode | -0.0231 | 0.1311 | 0.8669 | 0.98(0.76-1.26) |
| PTSD | genus | Slackia | Weighted mode | -0.0278 | 0.1136 | 0.8161 | 0.97(0.78-1.22) |
| PTSD | genus | Streptococcus | MR Egger | 0.0857 | 0.2562 | 0.7430 | 1.09(0.66-1.80) |
| PTSD | genus | Streptococcus | Weighted median | -0.0858 | 0.0964 | 0.3736 | 0.92(0.76-1.11) |
| PTSD | genus | Streptococcus | IVW | -0.0494 | 0.0710 | 0.4862 | 0.95(0.83-1.09) |
| PTSD | genus | Streptococcus | Simple mode | -0.1334 | 0.1623 | 0.4240 | 0.88(0.64-1.20) |
| PTSD | genus | Streptococcus | Weighted mode | -0.1236 | 0.1486 | 0.4184 | 0.88(0.66-1.18) |
| PTSD | genus | Subdoligranulum | MR Egger | -0.2850 | 0.2290 | 0.2447 | 0.75(0.48-1.18) |
| PTSD | genus | Subdoligranulum | Weighted median | -0.0848 | 0.1200 | 0.4794 | 0.92(0.73-1.16) |
| PTSD | genus | Subdoligranulum | IVW | -0.0745 | 0.0866 | 0.3900 | 0.93(0.78-1.10) |
| PTSD | genus | Subdoligranulum | Simple mode | 0.0617 | 0.2132 | 0.7781 | 1.06(0.70-1.62) |
| PTSD | genus | Subdoligranulum | Weighted mode | -0.2389 | 0.1943 | 0.2469 | 0.79(0.54-1.15) |
| PTSD | genus | Sutterella | MR Egger | -0.0685 | 0.4645 | 0.8858 | 0.93(0.38-2.32) |
| PTSD | genus | Sutterella | Weighted median | 0.1058 | 0.1099 | 0.3355 | 1.11(0.90-1.38) |
| PTSD | genus | Sutterella | IVW | 0.0823 | 0.0897 | 0.3587 | 1.09(0.91-1.29) |
| PTSD | genus | Sutterella | Simple mode | 0.1385 | 0.1848 | 0.4692 | 1.15(0.80-1.65) |
| PTSD | genus | Sutterella | Weighted mode | 0.1142 | 0.1777 | 0.5336 | 1.12(0.79-1.59) |
| PTSD | genus | Terrisporobacter | MR Egger | 0.2195 | 0.2132 | 0.3790 | 1.25(0.82-1.89) |
| PTSD | genus | Terrisporobacter | Weighted median | 0.0043 | 0.0960 | 0.9639 | 1.00(0.83-1.21) |
| PTSD | genus | Terrisporobacter | IVW | -0.0223 | 0.0785 | 0.7763 | 0.98(0.84-1.14) |
| PTSD | genus | Terrisporobacter | Simple mode | 0.0342 | 0.1318 | 0.8080 | 1.03(0.80-1.34) |
| PTSD | genus | Terrisporobacter | Weighted mode | 0.0524 | 0.1259 | 0.6987 | 1.05(0.82-1.35) |
| PTSD | genus | Turicibacter | MR Egger | 0.3765 | 0.2831 | 0.2201 | 1.46(0.84-2.54) |
| PTSD | genus | Turicibacter | Weighted median | -0.0536 | 0.0893 | 0.5486 | 0.95(0.80-1.13) |
| PTSD | genus | Turicibacter | IVW | -0.0804 | 0.0673 | 0.2322 | 0.92(0.81-1.05) |
| PTSD | genus | Turicibacter | Simple mode | -0.0211 | 0.1549 | 0.8946 | 0.98(0.72-1.33) |
| PTSD | genus | Turicibacter | Weighted mode | -0.0211 | 0.1649 | 0.9010 | 0.98(0.71-1.35) |
| PTSD | genus | Tyzzerella3 | MR Egger | -0.2459 | 0.2416 | 0.3306 | 0.78(0.49-1.26) |
| PTSD | genus | Tyzzerella3 | Weighted median | -0.0628 | 0.0584 | 0.2820 | 0.94(0.84-1.05) |
| PTSD | genus | Tyzzerella3 | IVW | -0.0615 | 0.0446 | 0.1676 | 0.94(0.86-1.03) |
| PTSD | genus | Tyzzerella3 | Simple mode | -0.0797 | 0.0962 | 0.4234 | 0.92(0.76-1.11) |
| PTSD | genus | Tyzzerella3 | Weighted mode | -0.0832 | 0.0932 | 0.3896 | 0.92(0.77-1.10) |
| PTSD | genus | Veillonella | MR Egger | -0.7989 | 0.5825 | 0.2193 | 0.45(0.14-1.41) |
| PTSD | genus | Veillonella | Weighted median | 0.0831 | 0.1153 | 0.4711 | 1.09(0.87-1.36) |
| PTSD | genus | Veillonella | IVW | 0.0867 | 0.0975 | 0.3739 | 1.09(0.90-1.32) |
| PTSD | genus | Veillonella | Simple mode | 0.0742 | 0.1685 | 0.6728 | 1.08(0.77-1.50) |
| PTSD | genus | Veillonella | Weighted mode | 0.0742 | 0.1567 | 0.6500 | 1.08(0.79-1.46) |
| PTSD | genus | Victivallis | MR Egger | -0.3483 | 0.2610 | 0.2148 | 0.71(0.42-1.18) |
| PTSD | genus | Victivallis | Weighted median | -0.0309 | 0.0496 | 0.5326 | 0.97(0.88-1.07) |
| PTSD | genus | Victivallis | IVW | -0.0293 | 0.0397 | 0.4612 | 0.97(0.90-1.05) |
| PTSD | genus | Victivallis | Simple mode | -0.0283 | 0.0799 | 0.7306 | 0.97(0.83-1.14) |
| PTSD | genus | Victivallis | Weighted mode | -0.0319 | 0.0749 | 0.6787 | 0.97(0.84-1.12) |
| PTSD | order | Actinomycetales | MR Egger | 0.0389 | 0.2327 | 0.8779 | 1.04(0.66-1.64) |
| PTSD | order | Actinomycetales | Weighted median | 0.0224 | 0.1055 | 0.8321 | 1.02(0.83-1.26) |
| PTSD | order | Actinomycetales | IVW | 0.0199 | 0.0839 | 0.8130 | 1.02(0.87-1.20) |
| PTSD | order | Actinomycetales | Simple mode | 0.0313 | 0.1455 | 0.8403 | 1.03(0.78-1.37) |
| PTSD | order | Actinomycetales | Weighted mode | 0.0230 | 0.1367 | 0.8747 | 1.02(0.78-1.34) |
| PTSD | order | Bacillales | MR Egger | 0.1540 | 0.2176 | 0.5021 | 1.17(0.76-1.79) |
| PTSD | order | Bacillales | Weighted median | -0.0431 | 0.0642 | 0.5016 | 0.96(0.84-1.09) |
| PTSD | order | Bacillales | IVW | -0.0246 | 0.0455 | 0.5886 | 0.98(0.89-1.07) |
| PTSD | order | Bacillales | Simple mode | -0.0793 | 0.1070 | 0.4799 | 0.92(0.75-1.14) |
| PTSD | order | Bacillales | Weighted mode | -0.0707 | 0.1019 | 0.5078 | 0.93(0.76-1.14) |
| PTSD | order | Bacteroidales | MR Egger | -0.0502 | 0.1701 | 0.7736 | 0.95(0.68-1.33) |
| PTSD | order | Bacteroidales | Weighted median | -0.0117 | 0.1043 | 0.9107 | 0.99(0.81-1.21) |
| PTSD | order | Bacteroidales | IVW | -0.0406 | 0.0779 | 0.6024 | 0.96(0.82-1.12) |
| PTSD | order | Bacteroidales | Simple mode | 0.1425 | 0.1781 | 0.4393 | 1.15(0.81-1.63) |
| PTSD | order | Bacteroidales | Weighted mode | 0.0968 | 0.1414 | 0.5066 | 1.10(0.83-1.45) |
| PTSD | order | Bifidobacteriales | MR Egger | -0.0159 | 0.2868 | 0.9566 | 0.98(0.56-1.73) |
| PTSD | order | Bifidobacteriales | Weighted median | -0.1320 | 0.0980 | 0.1781 | 0.88(0.72-1.06) |
| PTSD | order | Bifidobacteriales | IVW | -0.0589 | 0.0745 | 0.4291 | 0.94(0.81-1.09) |
| PTSD | order | Bifidobacteriales | Simple mode | 0.2168 | 0.2071 | 0.3129 | 1.24(0.83-1.86) |
| PTSD | order | Bifidobacteriales | Weighted mode | -0.2179 | 0.1396 | 0.1409 | 0.80(0.61-1.06) |
| PTSD | order | Burkholderiales | MR Egger | -0.3590 | 0.3022 | 0.2652 | 0.70(0.39-1.26) |
| PTSD | order | Burkholderiales | Weighted median | 0.0586 | 0.1208 | 0.6278 | 1.06(0.84-1.34) |
| PTSD | order | Burkholderiales | IVW | 0.1041 | 0.0891 | 0.2428 | 1.11(0.93-1.32) |
| PTSD | order | Burkholderiales | Simple mode | -0.0737 | 0.2188 | 0.7434 | 0.93(0.61-1.43) |
| PTSD | order | Burkholderiales | Weighted mode | -0.0590 | 0.2181 | 0.7925 | 0.94(0.61-1.45) |
| PTSD | order | Clostridiales | MR Egger | -0.2283 | 0.4355 | 0.6105 | 0.80(0.34-1.87) |
| PTSD | order | Clostridiales | Weighted median | -0.1844 | 0.1211 | 0.1278 | 0.83(0.66-1.05) |
| PTSD | order | Clostridiales | IVW | -0.2086 | 0.0867 | 0.0162 | 0.81(0.68-0.96) |
| PTSD | order | Clostridiales | Simple mode | -0.1904 | 0.2147 | 0.3926 | 0.83(0.54-1.26) |
| PTSD | order | Clostridiales | Weighted mode | -0.1870 | 0.1983 | 0.3641 | 0.83(0.56-1.22) |
| PTSD | order | Coriobacteriales | MR Egger | -0.3376 | 0.3237 | 0.3125 | 0.71(0.38-1.35) |
| PTSD | order | Coriobacteriales | Weighted median | 0.0324 | 0.1018 | 0.7501 | 1.03(0.85-1.26) |
| PTSD | order | Coriobacteriales | IVW | 0.0484 | 0.0802 | 0.5467 | 1.05(0.90-1.23) |
| PTSD | order | Coriobacteriales | Simple mode | -0.0261 | 0.1790 | 0.8857 | 0.97(0.69-1.38) |
| PTSD | order | Coriobacteriales | Weighted mode | -0.0261 | 0.1801 | 0.8864 | 0.97(0.68-1.39) |
| PTSD | order | Desulfovibrionales | MR Egger | 0.0426 | 0.2043 | 0.8391 | 1.04(0.70-1.56) |
| PTSD | order | Desulfovibrionales | Weighted median | 0.0125 | 0.1011 | 0.9019 | 1.01(0.83-1.23) |
| PTSD | order | Desulfovibrionales | IVW | 0.0240 | 0.0762 | 0.7525 | 1.02(0.88-1.19) |
| PTSD | order | Desulfovibrionales | Simple mode | 0.0054 | 0.1510 | 0.9721 | 1.01(0.75-1.35) |
| PTSD | order | Desulfovibrionales | Weighted mode | 0.0216 | 0.1314 | 0.8724 | 1.02(0.79-1.32) |
| PTSD | order | Enterobacteriales | MR Egger | 0.3596 | 0.6915 | 0.6252 | 1.43(0.37-5.56) |
| PTSD | order | Enterobacteriales | Weighted median | 0.1940 | 0.1462 | 0.1846 | 1.21(0.91-1.62) |
| PTSD | order | Enterobacteriales | IVW | 0.0626 | 0.1079 | 0.5618 | 1.06(0.86-1.32) |
| PTSD | order | Enterobacteriales | Simple mode | 0.2373 | 0.2263 | 0.3349 | 1.27(0.81-1.98) |
| PTSD | order | Enterobacteriales | Weighted mode | 0.2408 | 0.2355 | 0.3461 | 1.27(0.80-2.02) |
| PTSD | order | Erysipelotrichales | MR Egger | 0.2322 | 0.3705 | 0.5437 | 1.26(0.61-2.61) |
| PTSD | order | Erysipelotrichales | Weighted median | -0.0190 | 0.1168 | 0.8709 | 0.98(0.78-1.23) |
| PTSD | order | Erysipelotrichales | IVW | -0.0054 | 0.0849 | 0.9494 | 0.99(0.84-1.17) |
| PTSD | order | Erysipelotrichales | Simple mode | 0.0108 | 0.1998 | 0.9576 | 1.01(0.68-1.50) |
| PTSD | order | Erysipelotrichales | Weighted mode | 0.0197 | 0.2166 | 0.9290 | 1.02(0.67-1.56) |
| PTSD | order | Gastranaerophilales | MR Egger | -0.0576 | 0.1609 | 0.7311 | 0.94(0.69-1.29) |
| PTSD | order | Gastranaerophilales | Weighted median | -0.0165 | 0.0807 | 0.8383 | 0.98(0.84-1.15) |
| PTSD | order | Gastranaerophilales | IVW | -0.0001 | 0.0552 | 0.9993 | 1.00(0.90-1.11) |
| PTSD | order | Gastranaerophilales | Simple mode | -0.0096 | 0.1221 | 0.9393 | 0.99(0.78-1.26) |
| PTSD | order | Gastranaerophilales | Weighted mode | -0.0036 | 0.1265 | 0.9780 | 1.00(0.78-1.28) |
| PTSD | order | Lactobacillales | MR Egger | 0.0710 | 0.1865 | 0.7095 | 1.07(0.74-1.55) |
| PTSD | order | Lactobacillales | Weighted median | 0.0972 | 0.1000 | 0.3313 | 1.10(0.91-1.34) |
| PTSD | order | Lactobacillales | IVW | 0.0686 | 0.0728 | 0.3467 | 1.07(0.93-1.24) |
| PTSD | order | Lactobacillales | Simple mode | 0.1830 | 0.1731 | 0.3083 | 1.20(0.86-1.69) |
| PTSD | order | Lactobacillales | Weighted mode | 0.1479 | 0.1455 | 0.3265 | 1.16(0.87-1.54) |
| PTSD | order | Methanobacteriales | MR Egger | 0.0287 | 0.1777 | 0.8758 | 1.03(0.73-1.46) |
| PTSD | order | Methanobacteriales | Weighted median | -0.0120 | 0.0556 | 0.8290 | 0.99(0.89-1.10) |
| PTSD | order | Methanobacteriales | IVW | -0.0224 | 0.0423 | 0.5964 | 0.98(0.90-1.06) |
| PTSD | order | Methanobacteriales | Simple mode | -0.0155 | 0.0836 | 0.8569 | 0.98(0.84-1.16) |
| PTSD | order | Methanobacteriales | Weighted mode | -0.0117 | 0.0834 | 0.8920 | 0.99(0.84-1.16) |
| PTSD | order | MollicutesRF9 | MR Egger | 0.1523 | 0.1907 | 0.4414 | 1.16(0.80-1.69) |
| PTSD | order | MollicutesRF9 | Weighted median | 0.0265 | 0.0841 | 0.7527 | 1.03(0.87-1.21) |
| PTSD | order | MollicutesRF9 | IVW | 0.0186 | 0.0622 | 0.7653 | 1.02(0.90-1.15) |
| PTSD | order | MollicutesRF9 | Simple mode | 0.0561 | 0.1287 | 0.6706 | 1.06(0.82-1.36) |
| PTSD | order | MollicutesRF9 | Weighted mode | 0.0589 | 0.1225 | 0.6394 | 1.06(0.83-1.35) |
| PTSD | order | NB1n | MR Egger | 0.0682 | 0.1810 | 0.7135 | 1.07(0.75-1.53) |
| PTSD | order | NB1n | Weighted median | -0.0564 | 0.0617 | 0.3609 | 0.95(0.84-1.07) |
| PTSD | order | NB1n | IVW | -0.0368 | 0.0443 | 0.4069 | 0.96(0.88-1.05) |
| PTSD | order | NB1n | Simple mode | -0.0236 | 0.1105 | 0.8344 | 0.98(0.79-1.21) |
| PTSD | order | NB1n | Weighted mode | -0.0501 | 0.1124 | 0.6634 | 0.95(0.76-1.19) |
| PTSD | order | Pasteurellales | MR Egger | -0.0910 | 0.1169 | 0.4513 | 0.91(0.73-1.15) |
| PTSD | order | Pasteurellales | Weighted median | -0.0225 | 0.0717 | 0.7538 | 0.98(0.85-1.13) |
| PTSD | order | Pasteurellales | IVW | -0.0461 | 0.0513 | 0.3690 | 0.95(0.86-1.06) |
| PTSD | order | Pasteurellales | Simple mode | -0.0019 | 0.1211 | 0.9875 | 1.00(0.79-1.27) |
| PTSD | order | Pasteurellales | Weighted mode | -0.0114 | 0.1158 | 0.9228 | 0.99(0.79-1.24) |
| PTSD | order | Rhodospirillales | MR Egger | -0.1902 | 0.3354 | 0.5822 | 0.83(0.43-1.60) |
| PTSD | order | Rhodospirillales | Weighted median | -0.0216 | 0.0786 | 0.7839 | 0.98(0.84-1.14) |
| PTSD | order | Rhodospirillales | IVW | -0.0477 | 0.0595 | 0.4229 | 0.95(0.85-1.07) |
| PTSD | order | Rhodospirillales | Simple mode | -0.1395 | 0.1428 | 0.3476 | 0.87(0.66-1.15) |
| PTSD | order | Rhodospirillales | Weighted mode | -0.1545 | 0.1400 | 0.2915 | 0.86(0.65-1.13) |
| PTSD | order | Selenomonadales | MR Egger | 0.0108 | 0.2793 | 0.9700 | 1.01(0.58-1.75) |
| PTSD | order | Selenomonadales | Weighted median | -0.0518 | 0.1089 | 0.6341 | 0.95(0.77-1.18) |
| PTSD | order | Selenomonadales | IVW | -0.0600 | 0.0858 | 0.4846 | 0.94(0.80-1.11) |
| PTSD | order | Selenomonadales | Simple mode | -0.0205 | 0.2002 | 0.9204 | 0.98(0.66-1.45) |
| PTSD | order | Selenomonadales | Weighted mode | -0.0515 | 0.1703 | 0.7679 | 0.95(0.68-1.33) |
| PTSD | order | Verrucomicrobiales | MR Egger | 0.1818 | 0.2580 | 0.4972 | 1.20(0.72-1.99) |
| PTSD | order | Verrucomicrobiales | Weighted median | 0.1165 | 0.0957 | 0.2233 | 1.12(0.93-1.36) |
| PTSD | order | Verrucomicrobiales | IVW | 0.0826 | 0.0707 | 0.2430 | 1.09(0.95-1.25) |
| PTSD | order | Verrucomicrobiales | Simple mode | 0.2243 | 0.1756 | 0.2279 | 1.25(0.89-1.77) |
| PTSD | order | Verrucomicrobiales | Weighted mode | 0.2509 | 0.1796 | 0.1901 | 1.29(0.90-1.83) |
| PTSD | order | Victivallales | MR Egger | 0.0833 | 0.2179 | 0.7155 | 1.09(0.71-1.67) |
| PTSD | order | Victivallales | Weighted median | -0.0309 | 0.0737 | 0.6750 | 0.97(0.84-1.12) |
| PTSD | order | Victivallales | IVW | -0.0617 | 0.0601 | 0.3047 | 0.94(0.84-1.06) |
| PTSD | order | Victivallales | Simple mode | -0.0430 | 0.1276 | 0.7461 | 0.96(0.75-1.23) |
| PTSD | order | Victivallales | Weighted mode | -0.0277 | 0.1218 | 0.8264 | 0.97(0.77-1.23) |
| PTSD | phylum | Actinobacteria | MR Egger | -0.0400 | 0.3581 | 0.9128 | 0.96(0.48-1.94) |
| PTSD | phylum | Actinobacteria | Weighted median | 0.1154 | 0.1119 | 0.3022 | 1.12(0.90-1.40) |
| PTSD | phylum | Actinobacteria | IVW | 0.0285 | 0.0856 | 0.7392 | 1.03(0.87-1.22) |
| PTSD | phylum | Actinobacteria | Simple mode | 0.2416 | 0.2100 | 0.2694 | 1.27(0.84-1.92) |
| PTSD | phylum | Actinobacteria | Weighted mode | 0.2416 | 0.2408 | 0.3328 | 1.27(0.79-2.04) |
| PTSD | phylum | Bacteroidetes | MR Egger | -0.0708 | 0.1701 | 0.6861 | 0.93(0.67-1.30) |
| PTSD | phylum | Bacteroidetes | Weighted median | -0.0336 | 0.1041 | 0.7469 | 0.97(0.79-1.19) |
| PTSD | phylum | Bacteroidetes | IVW | -0.0019 | 0.0798 | 0.9812 | 1.00(0.85-1.17) |
| PTSD | phylum | Bacteroidetes | Simple mode | 0.1509 | 0.1856 | 0.4334 | 1.16(0.81-1.67) |
| PTSD | phylum | Bacteroidetes | Weighted mode | -0.0736 | 0.1473 | 0.6272 | 0.93(0.70-1.24) |
| PTSD | phylum | Cyanobacteria | MR Egger | 0.1694 | 0.2206 | 0.4717 | 1.18(0.77-1.83) |
| PTSD | phylum | Cyanobacteria | Weighted median | 0.0118 | 0.0891 | 0.8949 | 1.01(0.85-1.20) |
| PTSD | phylum | Cyanobacteria | IVW | 0.0130 | 0.0652 | 0.8422 | 1.01(0.89-1.15) |
| PTSD | phylum | Cyanobacteria | Simple mode | -0.0026 | 0.1393 | 0.9856 | 1.00(0.76-1.31) |
| PTSD | phylum | Cyanobacteria | Weighted mode | -0.0071 | 0.1300 | 0.9578 | 0.99(0.77-1.28) |
| PTSD | phylum | Euryarchaeota | MR Egger | 0.0896 | 0.1804 | 0.6300 | 1.09(0.77-1.56) |
| PTSD | phylum | Euryarchaeota | Weighted median | 0.0108 | 0.0514 | 0.8342 | 1.01(0.91-1.12) |
| PTSD | phylum | Euryarchaeota | IVW | 0.0278 | 0.0408 | 0.4964 | 1.03(0.95-1.11) |
| PTSD | phylum | Euryarchaeota | Simple mode | -0.0187 | 0.0899 | 0.8389 | 0.98(0.82-1.17) |
| PTSD | phylum | Euryarchaeota | Weighted mode | -0.0111 | 0.0906 | 0.9046 | 0.99(0.83-1.18) |
| PTSD | phylum | Firmicutes | MR Egger | -0.1344 | 0.2047 | 0.5230 | 0.87(0.59-1.31) |
| PTSD | phylum | Firmicutes | Weighted median | 0.0660 | 0.1070 | 0.5375 | 1.07(0.87-1.32) |
| PTSD | phylum | Firmicutes | IVW | -0.0230 | 0.0755 | 0.7605 | 0.98(0.84-1.13) |
| PTSD | phylum | Firmicutes | Simple mode | 0.1272 | 0.1929 | 0.5206 | 1.14(0.78-1.66) |
| PTSD | phylum | Firmicutes | Weighted mode | 0.1118 | 0.1674 | 0.5154 | 1.12(0.81-1.55) |
| PTSD | phylum | Lentisphaerae | MR Egger | 0.0950 | 0.2034 | 0.6546 | 1.10(0.74-1.64) |
| PTSD | phylum | Lentisphaerae | Weighted median | -0.0752 | 0.0708 | 0.2878 | 0.93(0.81-1.07) |
| PTSD | phylum | Lentisphaerae | IVW | -0.0643 | 0.0537 | 0.2314 | 0.94(0.84-1.04) |
| PTSD | phylum | Lentisphaerae | Simple mode | -0.0990 | 0.1162 | 0.4191 | 0.91(0.72-1.14) |
| PTSD | phylum | Lentisphaerae | Weighted mode | -0.0632 | 0.1227 | 0.6205 | 0.94(0.74-1.19) |
| PTSD | phylum | Proteobacteria | MR Egger | -0.2392 | 0.3592 | 0.5221 | 0.79(0.39-1.59) |
| PTSD | phylum | Proteobacteria | Weighted median | -0.1406 | 0.1285 | 0.2739 | 0.87(0.68-1.12) |
| PTSD | phylum | Proteobacteria | IVW | -0.0152 | 0.1142 | 0.8944 | 0.98(0.79-1.23) |
| PTSD | phylum | Proteobacteria | Simple mode | -0.1605 | 0.2163 | 0.4752 | 0.85(0.56-1.30) |
| PTSD | phylum | Proteobacteria | Weighted mode | -0.1686 | 0.2133 | 0.4474 | 0.84(0.56-1.28) |
| PTSD | phylum | Tenericutes | MR Egger | 0.1802 | 0.2165 | 0.4248 | 1.20(0.78-1.83) |
| PTSD | phylum | Tenericutes | Weighted median | 0.0842 | 0.0917 | 0.3584 | 1.09(0.91-1.30) |
| PTSD | phylum | Tenericutes | IVW | -0.0177 | 0.0669 | 0.7907 | 0.98(0.86-1.12) |
| PTSD | phylum | Tenericutes | Simple mode | 0.1318 | 0.1546 | 0.4121 | 1.14(0.84-1.54) |
| PTSD | phylum | Tenericutes | Weighted mode | 0.1106 | 0.1453 | 0.4626 | 1.12(0.84-1.48) |
| PTSD | phylum | Verrucomicrobia | MR Egger | -0.3638 | 0.2736 | 0.2132 | 0.70(0.41-1.19) |
| PTSD | phylum | Verrucomicrobia | Weighted median | -0.1032 | 0.1044 | 0.3229 | 0.90(0.73-1.11) |
| PTSD | phylum | Verrucomicrobia | IVW | -0.0714 | 0.0989 | 0.4708 | 0.93(0.77-1.13) |
| PTSD | phylum | Verrucomicrobia | Simple mode | -0.1946 | 0.2172 | 0.3894 | 0.82(0.54-1.26) |
| PTSD | phylum | Verrucomicrobia | Weighted mode | -0.1946 | 0.2223 | 0.4001 | 0.82(0.53-1.27) |

SNP, single nucleotide polymorphism; OR, odds ratio; CI, confidential interval, MR, mendelian randomization; PTSD, post-traumatic stress disorder

**Table S3** The MR-Egger and Cochran’s Q test of gut microbiome on PTSD in Freeze 2 datasets in the MR analysis

| Exposure | Outcome | Methods | *P*-Egger intercept | Egger_intercept | Cochran’s | Cochran’s *P* |
| --- | --- | --- | --- | --- | --- | --- |
|  | **family** |  |  |  |  |  |
| PTSD | Porphyromonadaceae | IVW |  |  | 10.34 | 0.3233 |
|  |  | MR-Egger | 0.3305 | -0.0258 | 9.12 | 0.3322 |
| PTSD | Veillonellaceae | IVW |  |  | 14.77 | 0.6774 |
|  |  | MR-Egger | 0.8718 | 0.0015 | 14.75 | 0.6136 |
|  | **genus** |  |  |  |  |  |
| PTSD | Dorea | IVW |  |  | 6.97 | 0.6398 |
|  |  | MR-Egger | 0.2369 | -0.0218 | 5.34 | 0.7208 |
| PTSD | Gordonibacter | IVW |  |  | 8.27 | 0.6889 |
|  |  | MR-Egger | 0.1747 | 0.0379 | 6.14 | 0.8037 |
| PTSD | Phascolarctobacterium | IVW |  |  | 12.21 | 0.2710 |
|  |  | MR-Egger | 0.9877 | -0.0005 | 12.21 | 0.2015 |
| PTSD | RuminococcaceaeUCG004 | IVW |  |  | 9.16 | 0.5171 |
|  |  | MR-Egger | 0.5502 | -0.0195 | 8.77 | 0.4584 |
| PTSD | Sellimonas | IVW |  |  | 8.47 | 0.3893 |
|  |  | MR-Egger | 0.9669 | 0.0015 | 8.46 | 0.2935 |
|  | **order** |  |  |  |  |  |
| PTSD | Clostridiales | IVW |  |  | 9.28 | 0.6789 |
|  |  | MR-Egger | 0.9640 | 0.0013 | 9.28 | 0.5964 |

MR, mendelian randomization; OR, odds ratio; CI; confidential interval; PTSD, post-traumatic stress disorder

**Table S4** MR results of causal links between gut microbiome and PTSD in FinnGen datasets in MR analysis

| **outcome** | **Classification** | **Traits** | **method** | **b** | **se** | **pval** | **estimate** |
| --- | --- | --- | --- | --- | --- | --- | --- |
| PTSD | class | Actinobacteria | MR Egger | -0.2876 | 0.4412 | 0.5258 | 0.75(0.32-1.78) |
| PTSD | class | Actinobacteria | Weighted median | 0.1149 | 0.2051 | 0.5754 | 1.12(0.75-1.68) |
| PTSD | class | Actinobacteria | IVW | -0.0545 | 0.1483 | 0.7131 | 0.95(0.71-1.27) |
| PTSD | class | Actinobacteria | Simple mode | 0.0149 | 0.3364 | 0.9653 | 1.02(0.52-1.96) |
| PTSD | class | Actinobacteria | Weighted mode | 0.0852 | 0.2643 | 0.7520 | 1.09(0.65-1.83) |
| PTSD | class | Alphaproteobacteria | MR Egger | 0.5910 | 0.9265 | 0.5583 | 1.81(0.29-11.10) |
| PTSD | class | Alphaproteobacteria | Weighted median | 0.0497 | 0.2684 | 0.8532 | 1.05(0.62-1.78) |
| PTSD | class | Alphaproteobacteria | IVW | -0.0136 | 0.2501 | 0.9565 | 0.99(0.60-1.61) |
| PTSD | class | Alphaproteobacteria | Simple mode | -0.5373 | 0.5058 | 0.3367 | 0.58(0.22-1.57) |
| PTSD | class | Alphaproteobacteria | Weighted mode | 0.3277 | 0.3864 | 0.4351 | 1.39(0.65-2.96) |
| PTSD | class | Bacilli | MR Egger | 0.2654 | 0.4034 | 0.5200 | 1.30(0.59-2.87) |
| PTSD | class | Bacilli | Weighted median | 0.1358 | 0.2049 | 0.5075 | 1.15(0.77-1.71) |
| PTSD | class | Bacilli | IVW | 0.3252 | 0.1457 | 0.0257 | 1.38(1.04-1.84) |
| PTSD | class | Bacilli | Simple mode | 0.0092 | 0.3628 | 0.9801 | 1.01(0.50-2.06) |
| PTSD | class | Bacilli | Weighted mode | 0.0008 | 0.3204 | 0.9980 | 1.00(0.53-1.88) |
| PTSD | class | Bacteroidia | MR Egger | -0.3534 | 0.4346 | 0.4351 | 0.70(0.30-1.65) |
| PTSD | class | Bacteroidia | Weighted median | -0.1994 | 0.2611 | 0.4452 | 0.82(0.49-1.37) |
| PTSD | class | Bacteroidia | IVW | -0.2141 | 0.1877 | 0.2540 | 0.81(0.56-1.17) |
| PTSD | class | Bacteroidia | Simple mode | 0.1842 | 0.4936 | 0.7161 | 1.20(0.46-3.16) |
| PTSD | class | Bacteroidia | Weighted mode | -0.6966 | 0.4535 | 0.1527 | 0.50(0.20-1.21) |
| PTSD | class | Betaproteobacteria | MR Egger | -0.0780 | 0.6244 | 0.9037 | 0.93(0.27-3.15) |
| PTSD | class | Betaproteobacteria | Weighted median | 0.0769 | 0.2550 | 0.7628 | 1.08(0.66-1.78) |
| PTSD | class | Betaproteobacteria | IVW | 0.1734 | 0.1973 | 0.3794 | 1.19(0.81-1.75) |
| PTSD | class | Betaproteobacteria | Simple mode | 0.1551 | 0.3980 | 0.7058 | 1.17(0.54-2.55) |
| PTSD | class | Betaproteobacteria | Weighted mode | 0.0967 | 0.3423 | 0.7838 | 1.10(0.56-2.15) |
| PTSD | class | Clostridia | MR Egger | -1.2297 | 0.8486 | 0.1853 | 0.29(0.06-1.54) |
| PTSD | class | Clostridia | Weighted median | 0.0185 | 0.2728 | 0.9460 | 1.02(0.60-1.74) |
| PTSD | class | Clostridia | IVW | -0.0311 | 0.2058 | 0.8800 | 0.97(0.65-1.45) |
| PTSD | class | Clostridia | Simple mode | 0.0639 | 0.4833 | 0.8977 | 1.07(0.41-2.75) |
| PTSD | class | Clostridia | Weighted mode | -0.1019 | 0.4325 | 0.8191 | 0.90(0.39-2.11) |
| PTSD | class | Coriobacteriia | MR Egger | -0.2990 | 0.6273 | 0.6401 | 0.74(0.22-2.54) |
| PTSD | class | Coriobacteriia | Weighted median | -0.0928 | 0.2018 | 0.6458 | 0.91(0.61-1.35) |
| PTSD | class | Coriobacteriia | IVW | -0.0698 | 0.1524 | 0.6470 | 0.93(0.69-1.26) |
| PTSD | class | Coriobacteriia | Simple mode | -0.2179 | 0.3668 | 0.5603 | 0.80(0.39-1.65) |
| PTSD | class | Coriobacteriia | Weighted mode | -0.2111 | 0.3566 | 0.5616 | 0.81(0.40-1.63) |
| PTSD | class | Deltaproteobacteria | MR Egger | -0.6677 | 0.8123 | 0.4303 | 0.51(0.10-2.52) |
| PTSD | class | Deltaproteobacteria | Weighted median | 0.0153 | 0.2431 | 0.9497 | 1.02(0.63-1.64) |
| PTSD | class | Deltaproteobacteria | IVW | 0.0350 | 0.1797 | 0.8456 | 1.04(0.73-1.47) |
| PTSD | class | Deltaproteobacteria | Simple mode | -0.0300 | 0.4008 | 0.9417 | 0.97(0.44-2.13) |
| PTSD | class | Deltaproteobacteria | Weighted mode | -0.0902 | 0.4033 | 0.8271 | 0.91(0.41-2.01) |
| PTSD | class | Erysipelotrichia | MR Egger | 0.9393 | 0.8472 | 0.2912 | 2.56(0.49-13.46) |
| PTSD | class | Erysipelotrichia | Weighted median | -0.0673 | 0.2638 | 0.7987 | 0.93(0.56-1.57) |
| PTSD | class | Erysipelotrichia | IVW | -0.3389 | 0.2068 | 0.1013 | 0.71(0.48-1.07) |
| PTSD | class | Erysipelotrichia | Simple mode | -0.1159 | 0.4653 | 0.8076 | 0.89(0.36-2.22) |
| PTSD | class | Erysipelotrichia | Weighted mode | -0.0968 | 0.4684 | 0.8398 | 0.91(0.36-2.27) |
| PTSD | class | Gammaproteobacteria | MR Egger | 1.2215 | 0.8850 | 0.2260 | 3.39(0.60-19.23) |
| PTSD | class | Gammaproteobacteria | Weighted median | -0.0365 | 0.3238 | 0.9102 | 0.96(0.51-1.82) |
| PTSD | class | Gammaproteobacteria | IVW | -0.2988 | 0.3189 | 0.3489 | 0.74(0.40-1.39) |
| PTSD | class | Gammaproteobacteria | Simple mode | -0.0376 | 0.4398 | 0.9346 | 0.96(0.41-2.28) |
| PTSD | class | Gammaproteobacteria | Weighted mode | -0.0495 | 0.4196 | 0.9100 | 0.95(0.42-2.17) |
| PTSD | class | Lentisphaeria | MR Egger | -0.0100 | 0.4691 | 0.9836 | 0.99(0.39-2.48) |
| PTSD | class | Lentisphaeria | Weighted median | 0.0060 | 0.1588 | 0.9701 | 1.01(0.74-1.37) |
| PTSD | class | Lentisphaeria | IVW | 0.0365 | 0.1219 | 0.7645 | 1.04(0.82-1.32) |
| PTSD | class | Lentisphaeria | Simple mode | -0.2617 | 0.3129 | 0.4306 | 0.77(0.42-1.42) |
| PTSD | class | Lentisphaeria | Weighted mode | -0.2657 | 0.2795 | 0.3735 | 0.77(0.44-1.33) |
| PTSD | class | Melainabacteria | MR Egger | 0.2364 | 0.5216 | 0.6625 | 1.27(0.46-3.52) |
| PTSD | class | Melainabacteria | Weighted median | 0.0385 | 0.1824 | 0.8329 | 1.04(0.73-1.49) |
| PTSD | class | Melainabacteria | IVW | 0.0791 | 0.1623 | 0.6260 | 1.08(0.79-1.49) |
| PTSD | class | Melainabacteria | Simple mode | 0.1576 | 0.3174 | 0.6316 | 1.17(0.63-2.18) |
| PTSD | class | Melainabacteria | Weighted mode | 0.1508 | 0.2681 | 0.5875 | 1.16(0.69-1.97) |
| PTSD | class | Methanobacteria | MR Egger | -0.1394 | 0.4817 | 0.7806 | 0.87(0.34-2.24) |
| PTSD | class | Methanobacteria | Weighted median | 0.2363 | 0.1458 | 0.1051 | 1.27(0.95-1.69) |
| PTSD | class | Methanobacteria | IVW | 0.1636 | 0.1177 | 0.1648 | 1.18(0.94-1.48) |
| PTSD | class | Methanobacteria | Simple mode | 0.3466 | 0.2362 | 0.1804 | 1.41(0.89-2.25) |
| PTSD | class | Methanobacteria | Weighted mode | 0.3119 | 0.1953 | 0.1490 | 1.37(0.93-2.00) |
| PTSD | class | Mollicutes | MR Egger | -0.4070 | 0.5024 | 0.4368 | 0.67(0.25-1.78) |
| PTSD | class | Mollicutes | Weighted median | 0.0309 | 0.1976 | 0.8759 | 1.03(0.70-1.52) |
| PTSD | class | Mollicutes | IVW | 0.0675 | 0.1480 | 0.6483 | 1.07(0.80-1.43) |
| PTSD | class | Mollicutes | Simple mode | -0.1871 | 0.3062 | 0.5537 | 0.83(0.46-1.51) |
| PTSD | class | Mollicutes | Weighted mode | -0.0811 | 0.2844 | 0.7809 | 0.92(0.53-1.61) |
| PTSD | class | Negativicutes | MR Egger | -0.6410 | 0.9307 | 0.5066 | 0.53(0.09-3.26) |
| PTSD | class | Negativicutes | Weighted median | -0.1897 | 0.2794 | 0.4971 | 0.83(0.48-1.43) |
| PTSD | class | Negativicutes | IVW | -0.1703 | 0.2597 | 0.5119 | 0.84(0.51-1.40) |
| PTSD | class | Negativicutes | Simple mode | 0.0852 | 0.5098 | 0.8703 | 1.09(0.40-2.96) |
| PTSD | class | Negativicutes | Weighted mode | -0.3379 | 0.4778 | 0.4942 | 0.71(0.28-1.82) |
| PTSD | class | Verrucomicrobiae | MR Egger | 0.1310 | 0.5332 | 0.8109 | 1.14(0.40-3.24) |
| PTSD | class | Verrucomicrobiae | Weighted median | 0.0339 | 0.2098 | 0.8718 | 1.03(0.69-1.56) |
| PTSD | class | Verrucomicrobiae | IVW | 0.1481 | 0.1497 | 0.3226 | 1.16(0.86-1.56) |
| PTSD | class | Verrucomicrobiae | Simple mode | 0.0268 | 0.3590 | 0.9418 | 1.03(0.51-2.08) |
| PTSD | class | Verrucomicrobiae | Weighted mode | 0.0268 | 0.3126 | 0.9332 | 1.03(0.56-1.90) |
| PTSD | family | Acidaminococcaceae | MR Egger | 0.8638 | 0.9885 | 0.4222 | 2.37(0.34-16.47) |
| PTSD | family | Acidaminococcaceae | Weighted median | 0.0586 | 0.2666 | 0.8259 | 1.06(0.63-1.79) |
| PTSD | family | Acidaminococcaceae | IVW | 0.1207 | 0.3184 | 0.7047 | 1.13(0.60-2.11) |
| PTSD | family | Acidaminococcaceae | Simple mode | -0.5785 | 0.4788 | 0.2725 | 0.56(0.22-1.43) |
| PTSD | family | Acidaminococcaceae | Weighted mode | -0.2343 | 0.4679 | 0.6344 | 0.79(0.32-1.98) |
| PTSD | family | Actinomycetaceae | MR Egger | -0.2150 | 0.4286 | 0.6657 | 0.81(0.35-1.87) |
| PTSD | family | Actinomycetaceae | Weighted median | 0.0560 | 0.2335 | 0.8105 | 1.06(0.67-1.67) |
| PTSD | family | Actinomycetaceae | IVW | 0.1260 | 0.1965 | 0.5216 | 1.13(0.77-1.67) |
| PTSD | family | Actinomycetaceae | Simple mode | 0.0030 | 0.3376 | 0.9936 | 1.00(0.52-1.94) |
| PTSD | family | Actinomycetaceae | Weighted mode | -0.0064 | 0.2629 | 0.9822 | 0.99(0.59-1.66) |
| PTSD | family | Alcaligenaceae | MR Egger | 0.5035 | 0.7932 | 0.5385 | 1.65(0.35-7.83) |
| PTSD | family | Alcaligenaceae | Weighted median | 0.0373 | 0.2484 | 0.8807 | 1.04(0.64-1.69) |
| PTSD | family | Alcaligenaceae | IVW | 0.0122 | 0.1812 | 0.9464 | 1.01(0.71-1.44) |
| PTSD | family | Alcaligenaceae | Simple mode | 0.0968 | 0.4357 | 0.8280 | 1.10(0.47-2.59) |
| PTSD | family | Alcaligenaceae | Weighted mode | 0.0278 | 0.4274 | 0.9492 | 1.03(0.44-2.38) |
| PTSD | family | Bacteroidaceae | MR Egger | -0.3119 | 1.1586 | 0.7968 | 0.73(0.08-7.09) |
| PTSD | family | Bacteroidaceae | Weighted median | -0.0419 | 0.2724 | 0.8776 | 0.96(0.56-1.64) |
| PTSD | family | Bacteroidaceae | IVW | -0.0427 | 0.2192 | 0.8454 | 0.96(0.62-1.47) |
| PTSD | family | Bacteroidaceae | Simple mode | -0.0355 | 0.3911 | 0.9303 | 0.97(0.45-2.08) |
| PTSD | family | Bacteroidaceae | Weighted mode | -0.0400 | 0.3808 | 0.9194 | 0.96(0.46-2.03) |
| PTSD | family | BacteroidalesS24 | MR Egger | -1.8162 | 0.6064 | 0.0242 | 0.16(0.05-0.53) |
| PTSD | family | BacteroidalesS24 | Weighted median | 0.1513 | 0.2315 | 0.5134 | 1.16(0.74-1.83) |
| PTSD | family | BacteroidalesS24 | IVW | 0.0533 | 0.2313 | 0.8179 | 1.05(0.67-1.66) |
| PTSD | family | BacteroidalesS24 | Simple mode | 0.3528 | 0.4634 | 0.4714 | 1.42(0.57-3.53) |
| PTSD | family | BacteroidalesS24 | Weighted mode | 0.3289 | 0.4643 | 0.5016 | 1.39(0.56-3.45) |
| PTSD | family | Bifidobacteriaceae | MR Egger | 0.6729 | 0.5076 | 0.2096 | 1.96(0.72-5.30) |
| PTSD | family | Bifidobacteriaceae | Weighted median | -0.0691 | 0.2010 | 0.7311 | 0.93(0.63-1.38) |
| PTSD | family | Bifidobacteriaceae | IVW | -0.1878 | 0.1437 | 0.1912 | 0.83(0.63-1.10) |
| PTSD | family | Bifidobacteriaceae | Simple mode | -0.5792 | 0.3933 | 0.1647 | 0.56(0.26-1.21) |
| PTSD | family | Bifidobacteriaceae | Weighted mode | 0.1615 | 0.2681 | 0.5573 | 1.18(0.69-1.99) |
| PTSD | family | Christensenellaceae | MR Egger | 0.2115 | 0.2843 | 0.4758 | 1.24(0.71-2.16) |
| PTSD | family | Christensenellaceae | Weighted median | 0.0688 | 0.1975 | 0.7273 | 1.07(0.73-1.58) |
| PTSD | family | Christensenellaceae | IVW | -0.0419 | 0.1472 | 0.7758 | 0.96(0.72-1.28) |
| PTSD | family | Christensenellaceae | Simple mode | 0.2164 | 0.2971 | 0.4831 | 1.24(0.69-2.22) |
| PTSD | family | Christensenellaceae | Weighted mode | 0.1231 | 0.2211 | 0.5899 | 1.13(0.73-1.74) |
| PTSD | family | Clostridiaceae1 | MR Egger | -0.0893 | 0.6405 | 0.8926 | 0.91(0.26-3.21) |
| PTSD | family | Clostridiaceae1 | Weighted median | 0.1797 | 0.2467 | 0.4665 | 1.20(0.74-1.94) |
| PTSD | family | Clostridiaceae1 | IVW | 0.0555 | 0.2091 | 0.7905 | 1.06(0.70-1.59) |
| PTSD | family | Clostridiaceae1 | Simple mode | 0.0754 | 0.3291 | 0.8238 | 1.08(0.57-2.06) |
| PTSD | family | Clostridiaceae1 | Weighted mode | 0.1273 | 0.3050 | 0.6860 | 1.14(0.62-2.06) |
| PTSD | family | ClostridialesvadinBB60group | MR Egger | 0.5060 | 0.3538 | 0.1763 | 1.66(0.83-3.32) |
| PTSD | family | ClostridialesvadinBB60group | Weighted median | 0.0872 | 0.1849 | 0.6373 | 1.09(0.76-1.57) |
| PTSD | family | ClostridialesvadinBB60group | IVW | 0.0049 | 0.1362 | 0.9712 | 1.00(0.77-1.31) |
| PTSD | family | ClostridialesvadinBB60group | Simple mode | 0.2633 | 0.3007 | 0.3960 | 1.30(0.72-2.35) |
| PTSD | family | ClostridialesvadinBB60group | Weighted mode | 0.1741 | 0.2587 | 0.5119 | 1.19(0.72-1.98) |
| PTSD | family | Coriobacteriaceae | MR Egger | -0.2990 | 0.6273 | 0.6401 | 0.74(0.22-2.54) |
| PTSD | family | Coriobacteriaceae | Weighted median | -0.0928 | 0.2087 | 0.6567 | 0.91(0.61-1.37) |
| PTSD | family | Coriobacteriaceae | IVW | -0.0698 | 0.1524 | 0.6470 | 0.93(0.69-1.26) |
| PTSD | family | Coriobacteriaceae | Simple mode | -0.2179 | 0.3877 | 0.5815 | 0.80(0.38-1.72) |
| PTSD | family | Coriobacteriaceae | Weighted mode | -0.2111 | 0.3539 | 0.5587 | 0.81(0.40-1.62) |
| PTSD | family | Defluviitaleaceae | MR Egger | -0.0276 | 0.5824 | 0.9633 | 0.97(0.31-3.05) |
| PTSD | family | Defluviitaleaceae | Weighted median | 0.0379 | 0.1998 | 0.8496 | 1.04(0.70-1.54) |
| PTSD | family | Defluviitaleaceae | IVW | -0.0906 | 0.1616 | 0.5750 | 0.91(0.67-1.25) |
| PTSD | family | Defluviitaleaceae | Simple mode | -0.5734 | 0.4248 | 0.2069 | 0.56(0.25-1.30) |
| PTSD | family | Defluviitaleaceae | Weighted mode | 0.2992 | 0.3824 | 0.4521 | 1.35(0.64-2.85) |
| PTSD | family | Desulfovibrionaceae | MR Egger | -0.3043 | 0.8292 | 0.7245 | 0.74(0.15-3.75) |
| PTSD | family | Desulfovibrionaceae | Weighted median | -0.1016 | 0.2526 | 0.6877 | 0.90(0.55-1.48) |
| PTSD | family | Desulfovibrionaceae | IVW | -0.1352 | 0.2021 | 0.5033 | 0.87(0.59-1.30) |
| PTSD | family | Desulfovibrionaceae | Simple mode | -0.0631 | 0.3835 | 0.8734 | 0.94(0.44-1.99) |
| PTSD | family | Desulfovibrionaceae | Weighted mode | -0.1043 | 0.3641 | 0.7819 | 0.90(0.44-1.84) |
| PTSD | family | Enterobacteriaceae | MR Egger | 1.0696 | 1.3443 | 0.4623 | 2.91(0.21-40.63) |
| PTSD | family | Enterobacteriaceae | Weighted median | 0.1372 | 0.2869 | 0.6324 | 1.15(0.65-2.01) |
| PTSD | family | Enterobacteriaceae | IVW | 0.2124 | 0.2245 | 0.3441 | 1.24(0.80-1.92) |
| PTSD | family | Enterobacteriaceae | Simple mode | 0.0814 | 0.4024 | 0.8464 | 1.08(0.49-2.39) |
| PTSD | family | Enterobacteriaceae | Weighted mode | 0.0814 | 0.4077 | 0.8484 | 1.08(0.49-2.41) |
| PTSD | family | Erysipelotrichaceae | MR Egger | 0.9393 | 0.8472 | 0.2912 | 2.56(0.49-13.46) |
| PTSD | family | Erysipelotrichaceae | Weighted median | -0.0673 | 0.2645 | 0.7991 | 0.93(0.56-1.57) |
| PTSD | family | Erysipelotrichaceae | IVW | -0.3389 | 0.2068 | 0.1013 | 0.71(0.48-1.07) |
| PTSD | family | Erysipelotrichaceae | Simple mode | -0.1159 | 0.5084 | 0.8236 | 0.89(0.33-2.41) |
| PTSD | family | Erysipelotrichaceae | Weighted mode | -0.0968 | 0.4519 | 0.8340 | 0.91(0.37-2.20) |
| PTSD | family | FamilyXI | MR Egger | 0.3413 | 0.7853 | 0.6790 | 1.41(0.30-6.56) |
| PTSD | family | FamilyXI | Weighted median | 0.1570 | 0.1282 | 0.2208 | 1.17(0.91-1.50) |
| PTSD | family | FamilyXI | IVW | 0.1426 | 0.1146 | 0.2132 | 1.15(0.92-1.44) |
| PTSD | family | FamilyXI | Simple mode | 0.1597 | 0.1924 | 0.4340 | 1.17(0.80-1.71) |
| PTSD | family | FamilyXI | Weighted mode | 0.1674 | 0.1717 | 0.3620 | 1.18(0.84-1.66) |
| PTSD | family | FamilyXIII | MR Egger | 1.4392 | 1.2025 | 0.2850 | 4.22(0.40-44.52) |
| PTSD | family | FamilyXIII | Weighted median | -0.1034 | 0.3782 | 0.7845 | 0.90(0.43-1.89) |
| PTSD | family | FamilyXIII | IVW | 0.0020 | 0.3324 | 0.9951 | 1.00(0.52-1.92) |
| PTSD | family | FamilyXIII | Simple mode | -0.6809 | 0.6988 | 0.3675 | 0.51(0.13-1.99) |
| PTSD | family | FamilyXIII | Weighted mode | -0.6497 | 0.6444 | 0.3523 | 0.52(0.15-1.85) |
| PTSD | family | Lachnospiraceae | MR Egger | 0.4610 | 0.5957 | 0.4518 | 1.59(0.49-5.10) |
| PTSD | family | Lachnospiraceae | Weighted median | -0.2389 | 0.2369 | 0.3133 | 0.79(0.49-1.25) |
| PTSD | family | Lachnospiraceae | IVW | -0.1262 | 0.1709 | 0.4603 | 0.88(0.63-1.23) |
| PTSD | family | Lachnospiraceae | Simple mode | -0.3138 | 0.4612 | 0.5066 | 0.73(0.30-1.80) |
| PTSD | family | Lachnospiraceae | Weighted mode | -0.2749 | 0.4180 | 0.5207 | 0.76(0.33-1.72) |
| PTSD | family | Lactobacillaceae | MR Egger | -0.3232 | 0.3055 | 0.3252 | 0.72(0.40-1.32) |
| PTSD | family | Lactobacillaceae | Weighted median | -0.1481 | 0.1698 | 0.3830 | 0.86(0.62-1.20) |
| PTSD | family | Lactobacillaceae | IVW | -0.0740 | 0.1242 | 0.5515 | 0.93(0.73-1.18) |
| PTSD | family | Lactobacillaceae | Simple mode | -0.1610 | 0.2635 | 0.5580 | 0.85(0.51-1.43) |
| PTSD | family | Lactobacillaceae | Weighted mode | -0.2421 | 0.2292 | 0.3217 | 0.79(0.50-1.23) |
| PTSD | family | Methanobacteriaceae | MR Egger | -0.1394 | 0.4817 | 0.7806 | 0.87(0.34-2.24) |
| PTSD | family | Methanobacteriaceae | Weighted median | 0.2363 | 0.1420 | 0.0962 | 1.27(0.96-1.67) |
| PTSD | family | Methanobacteriaceae | IVW | 0.1636 | 0.1177 | 0.1648 | 1.18(0.94-1.48) |
| PTSD | family | Methanobacteriaceae | Simple mode | 0.3466 | 0.2287 | 0.1681 | 1.41(0.90-2.21) |
| PTSD | family | Methanobacteriaceae | Weighted mode | 0.3119 | 0.2008 | 0.1590 | 1.37(0.92-2.02) |
| PTSD | family | Oxalobacteraceae | MR Egger | -0.2306 | 0.3498 | 0.5222 | 0.79(0.40-1.58) |
| PTSD | family | Oxalobacteraceae | Weighted median | -0.0482 | 0.1251 | 0.7002 | 0.95(0.75-1.22) |
| PTSD | family | Oxalobacteraceae | IVW | -0.0910 | 0.0906 | 0.3151 | 0.91(0.76-1.09) |
| PTSD | family | Oxalobacteraceae | Simple mode | -0.0141 | 0.2156 | 0.9490 | 0.99(0.65-1.50) |
| PTSD | family | Oxalobacteraceae | Weighted mode | -0.0250 | 0.2340 | 0.9166 | 0.98(0.62-1.54) |
| PTSD | family | Pasteurellaceae | MR Egger | -0.0414 | 0.2628 | 0.8776 | 0.96(0.57-1.61) |
| PTSD | family | Pasteurellaceae | Weighted median | 0.0982 | 0.1717 | 0.5672 | 1.10(0.79-1.54) |
| PTSD | family | Pasteurellaceae | IVW | 0.1278 | 0.1200 | 0.2868 | 1.14(0.90-1.44) |
| PTSD | family | Pasteurellaceae | Simple mode | 0.1602 | 0.3028 | 0.6064 | 1.17(0.65-2.12) |
| PTSD | family | Pasteurellaceae | Weighted mode | 0.0960 | 0.2997 | 0.7542 | 1.10(0.61-1.98) |
| PTSD | family | Peptococcaceae | MR Egger | -0.2160 | 0.5819 | 0.7201 | 0.81(0.26-2.52) |
| PTSD | family | Peptococcaceae | Weighted median | -0.2771 | 0.2148 | 0.1971 | 0.76(0.50-1.15) |
| PTSD | family | Peptococcaceae | IVW | -0.3431 | 0.1963 | 0.0805 | 0.71(0.48-1.04) |
| PTSD | family | Peptococcaceae | Simple mode | -0.1709 | 0.3513 | 0.6382 | 0.84(0.42-1.68) |
| PTSD | family | Peptococcaceae | Weighted mode | -0.1784 | 0.3065 | 0.5748 | 0.84(0.46-1.53) |
| PTSD | family | Peptostreptococcaceae | MR Egger | 0.0317 | 0.3342 | 0.9260 | 1.03(0.54-1.99) |
| PTSD | family | Peptostreptococcaceae | Weighted median | 0.0528 | 0.1985 | 0.7902 | 1.05(0.71-1.56) |
| PTSD | family | Peptostreptococcaceae | IVW | 0.1543 | 0.1466 | 0.2927 | 1.17(0.88-1.56) |
| PTSD | family | Peptostreptococcaceae | Simple mode | -0.1323 | 0.3094 | 0.6766 | 0.88(0.48-1.61) |
| PTSD | family | Peptostreptococcaceae | Weighted mode | -0.0617 | 0.2750 | 0.8262 | 0.94(0.55-1.61) |
| PTSD | family | Porphyromonadaceae | MR Egger | 0.2318 | 0.8125 | 0.7826 | 1.26(0.26-6.20) |
| PTSD | family | Porphyromonadaceae | Weighted median | -0.3820 | 0.2739 | 0.1631 | 0.68(0.40-1.17) |
| PTSD | family | Porphyromonadaceae | IVW | -0.3850 | 0.2144 | 0.0724 | 0.68(0.45-1.04) |
| PTSD | family | Porphyromonadaceae | Simple mode | -0.1124 | 0.4368 | 0.8027 | 0.89(0.38-2.10) |
| PTSD | family | Porphyromonadaceae | Weighted mode | -0.1029 | 0.4079 | 0.8066 | 0.90(0.41-2.01) |
| PTSD | family | Prevotellaceae | MR Egger | -0.0434 | 0.5434 | 0.9375 | 0.96(0.33-2.78) |
| PTSD | family | Prevotellaceae | Weighted median | 0.0383 | 0.2088 | 0.8546 | 1.04(0.69-1.56) |
| PTSD | family | Prevotellaceae | IVW | -0.0532 | 0.1464 | 0.7165 | 0.95(0.71-1.26) |
| PTSD | family | Prevotellaceae | Simple mode | 0.1136 | 0.3524 | 0.7517 | 1.12(0.56-2.24) |
| PTSD | family | Prevotellaceae | Weighted mode | 0.0940 | 0.3419 | 0.7871 | 1.10(0.56-2.15) |
| PTSD | family | Rhodospirillaceae | MR Egger | -0.0890 | 0.6218 | 0.8885 | 0.91(0.27-3.09) |
| PTSD | family | Rhodospirillaceae | Weighted median | 0.0567 | 0.1641 | 0.7299 | 1.06(0.77-1.46) |
| PTSD | family | Rhodospirillaceae | IVW | 0.0426 | 0.1473 | 0.7724 | 1.04(0.78-1.39) |
| PTSD | family | Rhodospirillaceae | Simple mode | 0.0343 | 0.2556 | 0.8953 | 1.03(0.63-1.71) |
| PTSD | family | Rhodospirillaceae | Weighted mode | 0.0181 | 0.2178 | 0.9351 | 1.02(0.66-1.56) |
| PTSD | family | Rikenellaceae | MR Egger | -0.0805 | 0.4627 | 0.8641 | 0.92(0.37-2.28) |
| PTSD | family | Rikenellaceae | Weighted median | 0.0236 | 0.2046 | 0.9083 | 1.02(0.69-1.53) |
| PTSD | family | Rikenellaceae | IVW | -0.0424 | 0.1482 | 0.7746 | 0.96(0.72-1.28) |
| PTSD | family | Rikenellaceae | Simple mode | 0.1872 | 0.4013 | 0.6468 | 1.21(0.55-2.65) |
| PTSD | family | Rikenellaceae | Weighted mode | 0.1595 | 0.3709 | 0.6727 | 1.17(0.57-2.43) |
| PTSD | family | Ruminococcaceae | MR Egger | -0.4847 | 0.3932 | 0.2527 | 0.62(0.28-1.33) |
| PTSD | family | Ruminococcaceae | Weighted median | -0.3402 | 0.2504 | 0.1743 | 0.71(0.44-1.16) |
| PTSD | family | Ruminococcaceae | IVW | -0.1464 | 0.1813 | 0.4192 | 0.86(0.61-1.23) |
| PTSD | family | Ruminococcaceae | Simple mode | -0.4671 | 0.4430 | 0.3192 | 0.63(0.26-1.49) |
| PTSD | family | Ruminococcaceae | Weighted mode | -0.4307 | 0.3872 | 0.2948 | 0.65(0.30-1.39) |
| PTSD | family | Streptococcaceae | MR Egger | 0.1990 | 0.7213 | 0.7878 | 1.22(0.30-5.02) |
| PTSD | family | Streptococcaceae | Weighted median | 0.0293 | 0.2379 | 0.9020 | 1.03(0.65-1.64) |
| PTSD | family | Streptococcaceae | IVW | 0.1005 | 0.1750 | 0.5656 | 1.11(0.78-1.56) |
| PTSD | family | Streptococcaceae | Simple mode | 0.0083 | 0.4031 | 0.9838 | 1.01(0.46-2.22) |
| PTSD | family | Streptococcaceae | Weighted mode | -0.0715 | 0.4046 | 0.8627 | 0.93(0.42-2.06) |
| PTSD | family | Veillonellaceae | MR Egger | 0.1268 | 0.2551 | 0.6260 | 1.14(0.69-1.87) |
| PTSD | family | Veillonellaceae | Weighted median | 0.2598 | 0.1847 | 0.1595 | 1.30(0.90-1.86) |
| PTSD | family | Veillonellaceae | IVW | 0.1538 | 0.1275 | 0.2278 | 1.17(0.91-1.50) |
| PTSD | family | Veillonellaceae | Simple mode | 0.4187 | 0.3315 | 0.2237 | 1.52(0.79-2.91) |
| PTSD | family | Veillonellaceae | Weighted mode | 0.2939 | 0.2435 | 0.2439 | 1.34(0.83-2.16) |
| PTSD | family | Verrucomicrobiaceae | MR Egger | 0.1289 | 0.5333 | 0.8140 | 1.14(0.40-3.24) |
| PTSD | family | Verrucomicrobiaceae | Weighted median | 0.0338 | 0.2093 | 0.8715 | 1.03(0.69-1.56) |
| PTSD | family | Verrucomicrobiaceae | IVW | 0.1480 | 0.1498 | 0.3231 | 1.16(0.86-1.56) |
| PTSD | family | Verrucomicrobiaceae | Simple mode | 0.0278 | 0.3374 | 0.9359 | 1.03(0.53-1.99) |
| PTSD | family | Verrucomicrobiaceae | Weighted mode | 0.0278 | 0.3350 | 0.9354 | 1.03(0.53-1.98) |
| PTSD | family | Victivallaceae | MR Egger | -0.5479 | 0.4153 | 0.2196 | 0.58(0.26-1.30) |
| PTSD | family | Victivallaceae | Weighted median | 0.0046 | 0.1285 | 0.9712 | 1.00(0.78-1.29) |
| PTSD | family | Victivallaceae | IVW | -0.0219 | 0.0892 | 0.8063 | 0.98(0.82-1.17) |
| PTSD | family | Victivallaceae | Simple mode | 0.1456 | 0.2257 | 0.5334 | 1.16(0.74-1.80) |
| PTSD | family | Victivallaceae | Weighted mode | -0.2188 | 0.2101 | 0.3222 | 0.80(0.53-1.21) |
| PTSD | genus | Clostridiuminnocuumgroup | MR Egger | -0.5861 | 0.5392 | 0.3187 | 0.56(0.19-1.60) |
| PTSD | genus | Clostridiuminnocuumgroup | Weighted median | 0.1471 | 0.1411 | 0.2971 | 1.16(0.88-1.53) |
| PTSD | genus | Clostridiuminnocuumgroup | IVW | 0.0635 | 0.1072 | 0.5536 | 1.07(0.86-1.31) |
| PTSD | genus | Clostridiuminnocuumgroup | Simple mode | 0.1961 | 0.2492 | 0.4572 | 1.22(0.75-1.98) |
| PTSD | genus | Clostridiuminnocuumgroup | Weighted mode | 0.1898 | 0.2432 | 0.4605 | 1.21(0.75-1.95) |
| PTSD | genus | Eubacteriumbrachygroup | MR Egger | 0.3438 | 0.5395 | 0.5418 | 1.41(0.49-4.06) |
| PTSD | genus | Eubacteriumbrachygroup | Weighted median | -0.1575 | 0.1456 | 0.2793 | 0.85(0.64-1.14) |
| PTSD | genus | Eubacteriumbrachygroup | IVW | -0.0493 | 0.1317 | 0.7085 | 0.95(0.74-1.23) |
| PTSD | genus | Eubacteriumbrachygroup | Simple mode | -0.2063 | 0.2689 | 0.4626 | 0.81(0.48-1.38) |
| PTSD | genus | Eubacteriumbrachygroup | Weighted mode | -0.2063 | 0.2872 | 0.4908 | 0.81(0.46-1.43) |
| PTSD | genus | Eubacteriumcoprostanoligenesgroup | MR Egger | 0.1214 | 0.7098 | 0.8676 | 1.13(0.28-4.54) |
| PTSD | genus | Eubacteriumcoprostanoligenesgroup | Weighted median | -0.1028 | 0.2710 | 0.7043 | 0.90(0.53-1.53) |
| PTSD | genus | Eubacteriumcoprostanoligenesgroup | IVW | -0.2078 | 0.1905 | 0.2754 | 0.81(0.56-1.18) |
| PTSD | genus | Eubacteriumcoprostanoligenesgroup | Simple mode | -0.0859 | 0.4659 | 0.8571 | 0.92(0.37-2.29) |
| PTSD | genus | Eubacteriumcoprostanoligenesgroup | Weighted mode | -0.0535 | 0.4180 | 0.9005 | 0.95(0.42-2.15) |
| PTSD | genus | Eubacteriumeligensgroup | MR Egger | 0.3692 | 1.4770 | 0.8149 | 1.45(0.08-26.16) |
| PTSD | genus | Eubacteriumeligensgroup | Weighted median | 0.4435 | 0.3259 | 0.1735 | 1.56(0.82-2.95) |
| PTSD | genus | Eubacteriumeligensgroup | IVW | 0.0345 | 0.3535 | 0.9224 | 1.04(0.52-2.07) |
| PTSD | genus | Eubacteriumeligensgroup | Simple mode | 0.4196 | 0.4322 | 0.3761 | 1.52(0.65-3.55) |
| PTSD | genus | Eubacteriumeligensgroup | Weighted mode | 0.4577 | 0.4086 | 0.3134 | 1.58(0.71-3.52) |
| PTSD | genus | Eubacteriumfissicatenagroup | MR Egger | -0.0762 | 0.5609 | 0.8957 | 0.93(0.31-2.78) |
| PTSD | genus | Eubacteriumfissicatenagroup | Weighted median | 0.2553 | 0.1458 | 0.0798 | 1.29(0.97-1.72) |
| PTSD | genus | Eubacteriumfissicatenagroup | IVW | 0.2400 | 0.1074 | 0.0255 | 1.27(1.03-1.57) |
| PTSD | genus | Eubacteriumfissicatenagroup | Simple mode | 0.2742 | 0.2083 | 0.2246 | 1.32(0.87-1.98) |
| PTSD | genus | Eubacteriumfissicatenagroup | Weighted mode | 0.2499 | 0.2081 | 0.2642 | 1.28(0.85-1.93) |
| PTSD | genus | Eubacteriumhalliigroup | MR Egger | 0.2613 | 0.4248 | 0.5501 | 1.30(0.56-2.99) |
| PTSD | genus | Eubacteriumhalliigroup | Weighted median | 0.2044 | 0.2245 | 0.3625 | 1.23(0.79-1.90) |
| PTSD | genus | Eubacteriumhalliigroup | IVW | 0.1173 | 0.1979 | 0.5532 | 1.12(0.76-1.66) |
| PTSD | genus | Eubacteriumhalliigroup | Simple mode | 0.3819 | 0.4021 | 0.3597 | 1.46(0.67-3.22) |
| PTSD | genus | Eubacteriumhalliigroup | Weighted mode | 0.3052 | 0.3454 | 0.3930 | 1.36(0.69-2.67) |
| PTSD | genus | Eubacteriumnodatumgroup | MR Egger | -0.3942 | 0.3908 | 0.3395 | 0.67(0.31-1.45) |
| PTSD | genus | Eubacteriumnodatumgroup | Weighted median | -0.1073 | 0.1177 | 0.3621 | 0.90(0.71-1.13) |
| PTSD | genus | Eubacteriumnodatumgroup | IVW | -0.0355 | 0.0877 | 0.6853 | 0.97(0.81-1.15) |
| PTSD | genus | Eubacteriumnodatumgroup | Simple mode | -0.0865 | 0.1755 | 0.6328 | 0.92(0.65-1.29) |
| PTSD | genus | Eubacteriumnodatumgroup | Weighted mode | -0.0972 | 0.1723 | 0.5850 | 0.91(0.65-1.27) |
| PTSD | genus | Eubacteriumoxidoreducensgroup | MR Egger | -0.0552 | 0.5973 | 0.9322 | 0.95(0.29-3.05) |
| PTSD | genus | Eubacteriumoxidoreducensgroup | Weighted median | -0.1966 | 0.2029 | 0.3325 | 0.82(0.55-1.22) |
| PTSD | genus | Eubacteriumoxidoreducensgroup | IVW | -0.2490 | 0.1603 | 0.1202 | 0.78(0.57-1.07) |
| PTSD | genus | Eubacteriumoxidoreducensgroup | Simple mode | -0.1902 | 0.2730 | 0.5244 | 0.83(0.48-1.41) |
| PTSD | genus | Eubacteriumoxidoreducensgroup | Weighted mode | -0.1437 | 0.2993 | 0.6561 | 0.87(0.48-1.56) |
| PTSD | genus | Eubacteriumrectalegroup | MR Egger | 0.0347 | 0.7901 | 0.9662 | 1.04(0.22-4.87) |
| PTSD | genus | Eubacteriumrectalegroup | Weighted median | -0.4739 | 0.2762 | 0.0862 | 0.62(0.36-1.07) |
| PTSD | genus | Eubacteriumrectalegroup | IVW | -0.2839 | 0.2097 | 0.1758 | 0.75(0.50-1.14) |
| PTSD | genus | Eubacteriumrectalegroup | Simple mode | -0.6024 | 0.4397 | 0.2079 | 0.55(0.23-1.30) |
| PTSD | genus | Eubacteriumrectalegroup | Weighted mode | -0.6160 | 0.4002 | 0.1623 | 0.54(0.25-1.18) |
| PTSD | genus | Eubacteriumruminantiumgroup | MR Egger | 0.2235 | 0.3286 | 0.5060 | 1.25(0.66-2.38) |
| PTSD | genus | Eubacteriumruminantiumgroup | Weighted median | 0.0877 | 0.1411 | 0.5341 | 1.09(0.83-1.44) |
| PTSD | genus | Eubacteriumruminantiumgroup | IVW | -0.0478 | 0.0972 | 0.6228 | 0.95(0.79-1.15) |
| PTSD | genus | Eubacteriumruminantiumgroup | Simple mode | 0.2233 | 0.2431 | 0.3713 | 1.25(0.78-2.01) |
| PTSD | genus | Eubacteriumruminantiumgroup | Weighted mode | 0.1959 | 0.2178 | 0.3809 | 1.22(0.79-1.86) |
| PTSD | genus | Eubacteriumventriosumgroup | MR Egger | -0.4589 | 0.9564 | 0.6393 | 0.63(0.10-4.12) |
| PTSD | genus | Eubacteriumventriosumgroup | Weighted median | -0.3148 | 0.2335 | 0.1776 | 0.73(0.46-1.15) |
| PTSD | genus | Eubacteriumventriosumgroup | IVW | -0.1716 | 0.2076 | 0.4085 | 0.84(0.56-1.27) |
| PTSD | genus | Eubacteriumventriosumgroup | Simple mode | -0.4186 | 0.4697 | 0.3879 | 0.66(0.26-1.65) |
| PTSD | genus | Eubacteriumventriosumgroup | Weighted mode | -0.4780 | 0.4421 | 0.2979 | 0.62(0.26-1.47) |
| PTSD | genus | Eubacteriumxylanophilumgroup | MR Egger | -0.2816 | 0.6535 | 0.6795 | 0.75(0.21-2.72) |
| PTSD | genus | Eubacteriumxylanophilumgroup | Weighted median | -0.3731 | 0.2472 | 0.1312 | 0.69(0.42-1.12) |
| PTSD | genus | Eubacteriumxylanophilumgroup | IVW | -0.2779 | 0.2052 | 0.1757 | 0.76(0.51-1.13) |
| PTSD | genus | Eubacteriumxylanophilumgroup | Simple mode | -0.5766 | 0.3883 | 0.1759 | 0.56(0.26-1.20) |
| PTSD | genus | Eubacteriumxylanophilumgroup | Weighted mode | -0.5608 | 0.3492 | 0.1470 | 0.57(0.29-1.13) |
| PTSD | genus | Ruminococcusgauvreauiigroup | MR Egger | 0.1137 | 0.6811 | 0.8711 | 1.12(0.29-4.26) |
| PTSD | genus | Ruminococcusgauvreauiigroup | Weighted median | -0.0126 | 0.2317 | 0.9566 | 0.99(0.63-1.56) |
| PTSD | genus | Ruminococcusgauvreauiigroup | IVW | -0.0863 | 0.1657 | 0.6024 | 0.92(0.66-1.27) |
| PTSD | genus | Ruminococcusgauvreauiigroup | Simple mode | -0.4671 | 0.3956 | 0.2651 | 0.63(0.29-1.36) |
| PTSD | genus | Ruminococcusgauvreauiigroup | Weighted mode | -0.4037 | 0.3851 | 0.3191 | 0.67(0.31-1.42) |
| PTSD | genus | Ruminococcusgnavusgroup | MR Egger | 0.0574 | 0.5630 | 0.9210 | 1.06(0.35-3.19) |
| PTSD | genus | Ruminococcusgnavusgroup | Weighted median | -0.0585 | 0.1559 | 0.7074 | 0.94(0.69-1.28) |
| PTSD | genus | Ruminococcusgnavusgroup | IVW | -0.2311 | 0.1172 | 0.0486 | 0.79(0.63-1.00) |
| PTSD | genus | Ruminococcusgnavusgroup | Simple mode | -0.0061 | 0.2846 | 0.9833 | 0.99(0.57-1.74) |
| PTSD | genus | Ruminococcusgnavusgroup | Weighted mode | -0.0028 | 0.2576 | 0.9914 | 1.00(0.60-1.65) |
| PTSD | genus | Ruminococcustorquesgroup | MR Egger | 0.7317 | 0.7732 | 0.3805 | 2.08(0.46-9.46) |
| PTSD | genus | Ruminococcustorquesgroup | Weighted median | 0.1830 | 0.3086 | 0.5532 | 1.20(0.66-2.20) |
| PTSD | genus | Ruminococcustorquesgroup | IVW | 0.0169 | 0.2423 | 0.9445 | 1.02(0.63-1.64) |
| PTSD | genus | Ruminococcustorquesgroup | Simple mode | 0.4788 | 0.5494 | 0.4123 | 1.61(0.55-4.74) |
| PTSD | genus | Ruminococcustorquesgroup | Weighted mode | 0.4716 | 0.5259 | 0.3996 | 1.60(0.57-4.49) |
| PTSD | genus | Actinomyces | MR Egger | -0.5869 | 0.3691 | 0.1727 | 0.56(0.27-1.15) |
| PTSD | genus | Actinomyces | Weighted median | 0.0307 | 0.2065 | 0.8818 | 1.03(0.69-1.55) |
| PTSD | genus | Actinomyces | IVW | -0.0864 | 0.1567 | 0.5814 | 0.92(0.67-1.25) |
| PTSD | genus | Actinomyces | Simple mode | 0.1498 | 0.3008 | 0.6363 | 1.16(0.64-2.09) |
| PTSD | genus | Actinomyces | Weighted mode | 0.0280 | 0.2556 | 0.9165 | 1.03(0.62-1.70) |
| PTSD | genus | Adlercreutzia | MR Egger | 0.2015 | 0.7891 | 0.8070 | 1.22(0.26-5.74) |
| PTSD | genus | Adlercreutzia | Weighted median | -0.1501 | 0.2153 | 0.4858 | 0.86(0.56-1.31) |
| PTSD | genus | Adlercreutzia | IVW | -0.0767 | 0.1654 | 0.6429 | 0.93(0.67-1.28) |
| PTSD | genus | Adlercreutzia | Simple mode | -0.1409 | 0.3378 | 0.6891 | 0.87(0.45-1.68) |
| PTSD | genus | Adlercreutzia | Weighted mode | -0.1409 | 0.3397 | 0.6907 | 0.87(0.45-1.69) |
| PTSD | genus | Akkermansia | MR Egger | 0.1343 | 0.5327 | 0.8060 | 1.14(0.40-3.25) |
| PTSD | genus | Akkermansia | Weighted median | 0.0339 | 0.2087 | 0.8708 | 1.03(0.69-1.56) |
| PTSD | genus | Akkermansia | IVW | 0.1484 | 0.1497 | 0.3216 | 1.16(0.86-1.56) |
| PTSD | genus | Akkermansia | Simple mode | 0.0273 | 0.3373 | 0.9369 | 1.03(0.53-1.99) |
| PTSD | genus | Akkermansia | Weighted mode | 0.0273 | 0.3167 | 0.9328 | 1.03(0.55-1.91) |
| PTSD | genus | Alistipes | MR Egger | -0.8179 | 1.1254 | 0.4841 | 0.44(0.05-4.01) |
| PTSD | genus | Alistipes | Weighted median | -0.0717 | 0.2933 | 0.8069 | 0.93(0.52-1.65) |
| PTSD | genus | Alistipes | IVW | 0.0033 | 0.2318 | 0.9885 | 1.00(0.64-1.58) |
| PTSD | genus | Alistipes | Simple mode | -0.1220 | 0.5372 | 0.8245 | 0.89(0.31-2.54) |
| PTSD | genus | Alistipes | Weighted mode | -0.1025 | 0.5209 | 0.8476 | 0.90(0.33-2.51) |
| PTSD | genus | Allisonella | MR Egger | -0.6911 | 0.8484 | 0.4464 | 0.50(0.09-2.64) |
| PTSD | genus | Allisonella | Weighted median | 0.0278 | 0.1196 | 0.8161 | 1.03(0.81-1.30) |
| PTSD | genus | Allisonella | IVW | 0.0309 | 0.1231 | 0.8018 | 1.03(0.81-1.31) |
| PTSD | genus | Allisonella | Simple mode | 0.1350 | 0.2077 | 0.5364 | 1.14(0.76-1.72) |
| PTSD | genus | Allisonella | Weighted mode | 0.0777 | 0.1866 | 0.6896 | 1.08(0.75-1.56) |
| PTSD | genus | Alloprevotella | MR Egger | 0.1207 | 1.0558 | 0.9162 | 1.13(0.14-8.94) |
| PTSD | genus | Alloprevotella | Weighted median | 0.0012 | 0.1632 | 0.9942 | 1.00(0.73-1.38) |
| PTSD | genus | Alloprevotella | IVW | 0.0891 | 0.1217 | 0.4643 | 1.09(0.86-1.39) |
| PTSD | genus | Alloprevotella | Simple mode | -0.0432 | 0.2165 | 0.8514 | 0.96(0.63-1.46) |
| PTSD | genus | Alloprevotella | Weighted mode | -0.0485 | 0.2144 | 0.8321 | 0.95(0.63-1.45) |
| PTSD | genus | Anaerofilum | MR Egger | -0.0035 | 0.6047 | 0.9955 | 1.00(0.30-3.26) |
| PTSD | genus | Anaerofilum | Weighted median | -0.0257 | 0.1385 | 0.8528 | 0.97(0.74-1.28) |
| PTSD | genus | Anaerofilum | IVW | -0.0374 | 0.1106 | 0.7352 | 0.96(0.78-1.20) |
| PTSD | genus | Anaerofilum | Simple mode | -0.0252 | 0.2187 | 0.9106 | 0.98(0.64-1.50) |
| PTSD | genus | Anaerofilum | Weighted mode | -0.0195 | 0.2269 | 0.9333 | 0.98(0.63-1.53) |
| PTSD | genus | Anaerostipes | MR Egger | 0.9073 | 0.7993 | 0.2805 | 2.48(0.52-11.87) |
| PTSD | genus | Anaerostipes | Weighted median | -0.2950 | 0.2790 | 0.2904 | 0.74(0.43-1.29) |
| PTSD | genus | Anaerostipes | IVW | -0.2247 | 0.2311 | 0.3311 | 0.80(0.51-1.26) |
| PTSD | genus | Anaerostipes | Simple mode | -0.7006 | 0.5847 | 0.2539 | 0.50(0.16-1.56) |
| PTSD | genus | Anaerostipes | Weighted mode | -0.6090 | 0.5454 | 0.2860 | 0.54(0.19-1.58) |
| PTSD | genus | Anaerotruncus | MR Egger | 0.0103 | 0.5084 | 0.9842 | 1.01(0.37-2.74) |
| PTSD | genus | Anaerotruncus | Weighted median | 0.1241 | 0.2424 | 0.6086 | 1.13(0.70-1.82) |
| PTSD | genus | Anaerotruncus | IVW | 0.2766 | 0.1742 | 0.1124 | 1.32(0.94-1.86) |
| PTSD | genus | Anaerotruncus | Simple mode | 0.1198 | 0.3970 | 0.7680 | 1.13(0.52-2.45) |
| PTSD | genus | Anaerotruncus | Weighted mode | 0.0559 | 0.3262 | 0.8668 | 1.06(0.56-2.00) |
| PTSD | genus | Bacteroides | MR Egger | -0.3119 | 1.1586 | 0.7968 | 0.73(0.08-7.09) |
| PTSD | genus | Bacteroides | Weighted median | -0.0419 | 0.2720 | 0.8775 | 0.96(0.56-1.63) |
| PTSD | genus | Bacteroides | IVW | -0.0427 | 0.2192 | 0.8454 | 0.96(0.62-1.47) |
| PTSD | genus | Bacteroides | Simple mode | -0.0355 | 0.3934 | 0.9307 | 0.97(0.45-2.09) |
| PTSD | genus | Bacteroides | Weighted mode | -0.0400 | 0.3937 | 0.9220 | 0.96(0.44-2.08) |
| PTSD | genus | Barnesiella | MR Egger | 0.0199 | 0.6990 | 0.9779 | 1.02(0.26-4.01) |
| PTSD | genus | Barnesiella | Weighted median | -0.1178 | 0.2249 | 0.6005 | 0.89(0.57-1.38) |
| PTSD | genus | Barnesiella | IVW | -0.1724 | 0.1699 | 0.3103 | 0.84(0.60-1.17) |
| PTSD | genus | Barnesiella | Simple mode | -0.5688 | 0.4071 | 0.1899 | 0.57(0.25-1.26) |
| PTSD | genus | Barnesiella | Weighted mode | 0.2366 | 0.3925 | 0.5589 | 1.27(0.59-2.73) |
| PTSD | genus | Bifidobacterium | MR Egger | 0.3859 | 0.3539 | 0.2969 | 1.47(0.74-2.94) |
| PTSD | genus | Bifidobacterium | Weighted median | 0.1255 | 0.1891 | 0.5068 | 1.13(0.78-1.64) |
| PTSD | genus | Bifidobacterium | IVW | -0.1478 | 0.1375 | 0.2824 | 0.86(0.66-1.13) |
| PTSD | genus | Bifidobacterium | Simple mode | 0.1328 | 0.3394 | 0.7019 | 1.14(0.59-2.22) |
| PTSD | genus | Bifidobacterium | Weighted mode | 0.1533 | 0.2181 | 0.4946 | 1.17(0.76-1.79) |
| PTSD | genus | Bilophila | MR Egger | -0.4692 | 0.7988 | 0.5688 | 0.63(0.13-2.99) |
| PTSD | genus | Bilophila | Weighted median | 0.2085 | 0.2307 | 0.3659 | 1.23(0.78-1.94) |
| PTSD | genus | Bilophila | IVW | 0.2361 | 0.1621 | 0.1454 | 1.27(0.92-1.74) |
| PTSD | genus | Bilophila | Simple mode | 0.0533 | 0.3738 | 0.8890 | 1.05(0.51-2.19) |
| PTSD | genus | Bilophila | Weighted mode | 0.0468 | 0.3698 | 0.9014 | 1.05(0.51-2.16) |
| PTSD | genus | Blautia | MR Egger | 0.3743 | 0.5731 | 0.5284 | 1.45(0.47-4.47) |
| PTSD | genus | Blautia | Weighted median | -0.1214 | 0.2787 | 0.6632 | 0.89(0.51-1.53) |
| PTSD | genus | Blautia | IVW | -0.2569 | 0.2261 | 0.2559 | 0.77(0.50-1.20) |
| PTSD | genus | Blautia | Simple mode | 0.3204 | 0.5253 | 0.5543 | 1.38(0.49-3.86) |
| PTSD | genus | Blautia | Weighted mode | 0.3302 | 0.5104 | 0.5309 | 1.39(0.51-3.78) |
| PTSD | genus | Butyricicoccus | MR Egger | -0.4695 | 0.3830 | 0.2662 | 0.63(0.30-1.32) |
| PTSD | genus | Butyricicoccus | Weighted median | -0.2727 | 0.2552 | 0.2853 | 0.76(0.46-1.26) |
| PTSD | genus | Butyricicoccus | IVW | -0.2695 | 0.1968 | 0.1708 | 0.76(0.52-1.12) |
| PTSD | genus | Butyricicoccus | Simple mode | -0.3011 | 0.4112 | 0.4878 | 0.74(0.33-1.66) |
| PTSD | genus | Butyricicoccus | Weighted mode | -0.3596 | 0.3319 | 0.3144 | 0.70(0.36-1.34) |
| PTSD | genus | Butyricimonas | MR Egger | 1.0226 | 0.5331 | 0.0814 | 2.78(0.98-7.90) |
| PTSD | genus | Butyricimonas | Weighted median | 0.1532 | 0.2081 | 0.4618 | 1.17(0.78-1.75) |
| PTSD | genus | Butyricimonas | IVW | 0.0111 | 0.1503 | 0.9414 | 1.01(0.75-1.36) |
| PTSD | genus | Butyricimonas | Simple mode | 0.2792 | 0.3315 | 0.4161 | 1.32(0.69-2.53) |
| PTSD | genus | Butyricimonas | Weighted mode | 0.2155 | 0.2988 | 0.4846 | 1.24(0.69-2.23) |
| PTSD | genus | Butyrivibrio | MR Egger | -0.5799 | 0.3154 | 0.0889 | 0.56(0.30-1.04) |
| PTSD | genus | Butyrivibrio | Weighted median | -0.1453 | 0.0985 | 0.1403 | 0.86(0.71-1.05) |
| PTSD | genus | Butyrivibrio | IVW | -0.1904 | 0.0730 | 0.0091 | 0.83(0.72-0.95) |
| PTSD | genus | Butyrivibrio | Simple mode | -0.0414 | 0.1893 | 0.8299 | 0.96(0.66-1.39) |
| PTSD | genus | Butyrivibrio | Weighted mode | -0.0323 | 0.1917 | 0.8688 | 0.97(0.66-1.41) |
| PTSD | genus | CandidatusSoleaferrea | MR Egger | 0.7951 | 2.2279 | 0.7317 | 2.21(0.03-174.49) |
| PTSD | genus | CandidatusSoleaferrea | Weighted median | 0.0696 | 0.2053 | 0.7345 | 1.07(0.72-1.60) |
| PTSD | genus | CandidatusSoleaferrea | IVW | 0.0116 | 0.2060 | 0.9551 | 1.01(0.68-1.51) |
| PTSD | genus | CandidatusSoleaferrea | Simple mode | 0.4664 | 0.4201 | 0.2992 | 1.59(0.70-3.63) |
| PTSD | genus | CandidatusSoleaferrea | Weighted mode | 0.4588 | 0.4128 | 0.2987 | 1.58(0.70-3.55) |
| PTSD | genus | Catenibacterium | MR Egger | 0.0761 | 1.8100 | 0.9703 | 1.08(0.03-37.48) |
| PTSD | genus | Catenibacterium | Weighted median | -0.0819 | 0.1697 | 0.6296 | 0.92(0.66-1.29) |
| PTSD | genus | Catenibacterium | IVW | -0.0618 | 0.1444 | 0.6686 | 0.94(0.71-1.25) |
| PTSD | genus | Catenibacterium | Simple mode | -0.1261 | 0.2355 | 0.6295 | 0.88(0.56-1.40) |
| PTSD | genus | Catenibacterium | Weighted mode | -0.1244 | 0.2302 | 0.6264 | 0.88(0.56-1.39) |
| PTSD | genus | ChristensenellaceaeR | MR Egger | -0.6407 | 0.8260 | 0.4674 | 0.53(0.10-2.66) |
| PTSD | genus | ChristensenellaceaeR | Weighted median | 0.0366 | 0.3060 | 0.9048 | 1.04(0.57-1.89) |
| PTSD | genus | ChristensenellaceaeR | IVW | -0.0108 | 0.2347 | 0.9634 | 0.99(0.62-1.57) |
| PTSD | genus | ChristensenellaceaeR | Simple mode | 0.0744 | 0.4145 | 0.8626 | 1.08(0.48-2.43) |
| PTSD | genus | ChristensenellaceaeR | Weighted mode | 0.0813 | 0.3977 | 0.8438 | 1.08(0.50-2.37) |
| PTSD | genus | Clostridiumsensustricto1 | MR Egger | -0.1088 | 0.7482 | 0.8915 | 0.90(0.21-3.89) |
| PTSD | genus | Clostridiumsensustricto1 | Weighted median | -0.0842 | 0.2479 | 0.7340 | 0.92(0.57-1.49) |
| PTSD | genus | Clostridiumsensustricto1 | IVW | -0.0402 | 0.2470 | 0.8708 | 0.96(0.59-1.56) |
| PTSD | genus | Clostridiumsensustricto1 | Simple mode | -0.0813 | 0.3310 | 0.8157 | 0.92(0.48-1.76) |
| PTSD | genus | Clostridiumsensustricto1 | Weighted mode | -0.0544 | 0.2961 | 0.8615 | 0.95(0.53-1.69) |
| PTSD | genus | Collinsella | MR Egger | 1.8522 | 0.7905 | 0.0516 | 6.37(1.35-30.01) |
| PTSD | genus | Collinsella | Weighted median | 0.2875 | 0.2824 | 0.3086 | 1.33(0.77-2.32) |
| PTSD | genus | Collinsella | IVW | 0.2240 | 0.2112 | 0.2889 | 1.25(0.83-1.89) |
| PTSD | genus | Collinsella | Simple mode | 0.2944 | 0.4676 | 0.5464 | 1.34(0.54-3.36) |
| PTSD | genus | Collinsella | Weighted mode | 0.2944 | 0.4502 | 0.5314 | 1.34(0.56-3.24) |
| PTSD | genus | Coprobacter | MR Egger | -0.0775 | 0.4778 | 0.8747 | 0.93(0.36-2.36) |
| PTSD | genus | Coprobacter | Weighted median | 0.3208 | 0.1653 | 0.0523 | 1.38(1.00-1.91) |
| PTSD | genus | Coprobacter | IVW | 0.2009 | 0.1221 | 0.0999 | 1.22(0.96-1.55) |
| PTSD | genus | Coprobacter | Simple mode | 0.4418 | 0.2684 | 0.1308 | 1.56(0.92-2.63) |
| PTSD | genus | Coprobacter | Weighted mode | 0.4227 | 0.2676 | 0.1452 | 1.53(0.90-2.58) |
| PTSD | genus | Coprococcus1 | MR Egger | -0.7336 | 0.4225 | 0.1131 | 0.48(0.21-1.10) |
| PTSD | genus | Coprococcus1 | Weighted median | -0.4371 | 0.2458 | 0.0754 | 0.65(0.40-1.05) |
| PTSD | genus | Coprococcus1 | IVW | -0.3060 | 0.1687 | 0.0697 | 0.74(0.53-1.03) |
| PTSD | genus | Coprococcus1 | Simple mode | -0.4596 | 0.3724 | 0.2429 | 0.63(0.30-1.31) |
| PTSD | genus | Coprococcus1 | Weighted mode | -0.4436 | 0.3386 | 0.2169 | 0.64(0.33-1.25) |
| PTSD | genus | Coprococcus2 | MR Egger | -0.0250 | 0.9463 | 0.9796 | 0.98(0.15-6.23) |
| PTSD | genus | Coprococcus2 | Weighted median | -0.0565 | 0.2370 | 0.8116 | 0.95(0.59-1.50) |
| PTSD | genus | Coprococcus2 | IVW | -0.0493 | 0.1745 | 0.7776 | 0.95(0.68-1.34) |
| PTSD | genus | Coprococcus2 | Simple mode | -0.1049 | 0.3433 | 0.7677 | 0.90(0.46-1.76) |
| PTSD | genus | Coprococcus2 | Weighted mode | -0.0802 | 0.3465 | 0.8228 | 0.92(0.47-1.82) |
| PTSD | genus | Coprococcus3 | MR Egger | -1.2346 | 1.3866 | 0.4028 | 0.29(0.02-4.41) |
| PTSD | genus | Coprococcus3 | Weighted median | -0.0375 | 0.2749 | 0.8915 | 0.96(0.56-1.65) |
| PTSD | genus | Coprococcus3 | IVW | -0.0291 | 0.2231 | 0.8962 | 0.97(0.63-1.50) |
| PTSD | genus | Coprococcus3 | Simple mode | -0.0062 | 0.4198 | 0.9886 | 0.99(0.44-2.26) |
| PTSD | genus | Coprococcus3 | Weighted mode | 0.0362 | 0.4102 | 0.9318 | 1.04(0.46-2.32) |
| PTSD | genus | DefluviitaleaceaeUCG011 | MR Egger | -0.0457 | 0.5908 | 0.9405 | 0.96(0.30-3.04) |
| PTSD | genus | DefluviitaleaceaeUCG011 | Weighted median | -0.2556 | 0.2203 | 0.2460 | 0.77(0.50-1.19) |
| PTSD | genus | DefluviitaleaceaeUCG011 | IVW | -0.2093 | 0.1556 | 0.1787 | 0.81(0.60-1.10) |
| PTSD | genus | DefluviitaleaceaeUCG011 | Simple mode | -0.4831 | 0.3740 | 0.2326 | 0.62(0.30-1.28) |
| PTSD | genus | DefluviitaleaceaeUCG011 | Weighted mode | -0.4143 | 0.3491 | 0.2694 | 0.66(0.33-1.31) |
| PTSD | genus | Desulfovibrio | MR Egger | -0.9212 | 0.5243 | 0.1169 | 0.40(0.14-1.11) |
| PTSD | genus | Desulfovibrio | Weighted median | -0.2152 | 0.2192 | 0.3263 | 0.81(0.52-1.24) |
| PTSD | genus | Desulfovibrio | IVW | -0.2686 | 0.1854 | 0.1473 | 0.76(0.53-1.10) |
| PTSD | genus | Desulfovibrio | Simple mode | -0.0019 | 0.4006 | 0.9964 | 1.00(0.46-2.19) |
| PTSD | genus | Desulfovibrio | Weighted mode | -0.1323 | 0.3273 | 0.6954 | 0.88(0.46-1.66) |
| PTSD | genus | Dialister | MR Egger | 0.2255 | 0.8533 | 0.7975 | 1.25(0.24-6.67) |
| PTSD | genus | Dialister | Weighted median | 0.1704 | 0.2385 | 0.4748 | 1.19(0.74-1.89) |
| PTSD | genus | Dialister | IVW | -0.1380 | 0.1992 | 0.4884 | 0.87(0.59-1.29) |
| PTSD | genus | Dialister | Simple mode | 0.3182 | 0.3800 | 0.4220 | 1.37(0.65-2.89) |
| PTSD | genus | Dialister | Weighted mode | 0.3182 | 0.3555 | 0.3918 | 1.37(0.68-2.76) |
| PTSD | genus | Dorea | MR Egger | -0.5349 | 0.5811 | 0.3842 | 0.59(0.19-1.83) |
| PTSD | genus | Dorea | Weighted median | -0.4460 | 0.2850 | 0.1177 | 0.64(0.37-1.12) |
| PTSD | genus | Dorea | IVW | -0.4379 | 0.2088 | 0.0360 | 0.65(0.43-0.97) |
| PTSD | genus | Dorea | Simple mode | -0.3987 | 0.4724 | 0.4206 | 0.67(0.27-1.69) |
| PTSD | genus | Dorea | Weighted mode | -0.4225 | 0.4061 | 0.3253 | 0.66(0.30-1.45) |
| PTSD | genus | Eggerthella | MR Egger | -0.5710 | 0.5287 | 0.3159 | 0.56(0.20-1.59) |
| PTSD | genus | Eggerthella | Weighted median | -0.2246 | 0.1563 | 0.1506 | 0.80(0.59-1.09) |
| PTSD | genus | Eggerthella | IVW | -0.2823 | 0.1183 | 0.0170 | 0.75(0.60-0.95) |
| PTSD | genus | Eggerthella | Simple mode | -0.0532 | 0.2648 | 0.8457 | 0.95(0.56-1.59) |
| PTSD | genus | Eggerthella | Weighted mode | -0.0597 | 0.2435 | 0.8125 | 0.94(0.58-1.52) |
| PTSD | genus | Eisenbergiella | MR Egger | -0.2632 | 0.8236 | 0.7566 | 0.77(0.15-3.86) |
| PTSD | genus | Eisenbergiella | Weighted median | -0.1279 | 0.1492 | 0.3914 | 0.88(0.66-1.18) |
| PTSD | genus | Eisenbergiella | IVW | -0.1016 | 0.1113 | 0.3609 | 0.90(0.73-1.12) |
| PTSD | genus | Eisenbergiella | Simple mode | -0.1927 | 0.2481 | 0.4553 | 0.82(0.51-1.34) |
| PTSD | genus | Eisenbergiella | Weighted mode | -0.1718 | 0.2638 | 0.5296 | 0.84(0.50-1.41) |
| PTSD | genus | Enterorhabdus | MR Egger | 0.3054 | 0.4662 | 0.5482 | 1.36(0.54-3.38) |
| PTSD | genus | Enterorhabdus | Weighted median | -0.1838 | 0.2209 | 0.4053 | 0.83(0.54-1.28) |
| PTSD | genus | Enterorhabdus | IVW | -0.1751 | 0.1769 | 0.3222 | 0.84(0.59-1.19) |
| PTSD | genus | Enterorhabdus | Simple mode | -0.4397 | 0.3761 | 0.2951 | 0.64(0.31-1.35) |
| PTSD | genus | Enterorhabdus | Weighted mode | 0.0581 | 0.2948 | 0.8514 | 1.06(0.59-1.89) |
| PTSD | genus | Erysipelatoclostridium | MR Egger | -0.5079 | 0.4809 | 0.3101 | 0.60(0.23-1.54) |
| PTSD | genus | Erysipelatoclostridium | Weighted median | -0.0692 | 0.1662 | 0.6770 | 0.93(0.67-1.29) |
| PTSD | genus | Erysipelatoclostridium | IVW | -0.0392 | 0.1231 | 0.7500 | 0.96(0.76-1.22) |
| PTSD | genus | Erysipelatoclostridium | Simple mode | -0.2022 | 0.3278 | 0.5473 | 0.82(0.43-1.55) |
| PTSD | genus | Erysipelatoclostridium | Weighted mode | -0.2202 | 0.3305 | 0.5161 | 0.80(0.42-1.53) |
| PTSD | genus | ErysipelotrichaceaeUCG003 | MR Egger | 0.3717 | 0.4226 | 0.3930 | 1.45(0.63-3.32) |
| PTSD | genus | ErysipelotrichaceaeUCG003 | Weighted median | -0.2659 | 0.1888 | 0.1591 | 0.77(0.53-1.11) |
| PTSD | genus | ErysipelotrichaceaeUCG003 | IVW | -0.0784 | 0.1513 | 0.6042 | 0.92(0.69-1.24) |
| PTSD | genus | ErysipelotrichaceaeUCG003 | Simple mode | -0.3666 | 0.3944 | 0.3664 | 0.69(0.32-1.50) |
| PTSD | genus | ErysipelotrichaceaeUCG003 | Weighted mode | -0.3432 | 0.3202 | 0.2997 | 0.71(0.38-1.33) |
| PTSD | genus | Escherichia | MR Egger | -0.0907 | 0.6124 | 0.8859 | 0.91(0.28-3.03) |
| PTSD | genus | Escherichia | Weighted median | 0.5856 | 0.2614 | 0.0251 | 1.80(1.08-3.00) |
| PTSD | genus | Escherichia | IVW | 0.2742 | 0.1890 | 0.1468 | 1.32(0.91-1.91) |
| PTSD | genus | Escherichia | Simple mode | 0.7502 | 0.4580 | 0.1359 | 2.12(0.86-5.20) |
| PTSD | genus | Escherichia | Weighted mode | 0.7617 | 0.4520 | 0.1263 | 2.14(0.88-5.20) |
| PTSD | genus | Faecalibacterium | MR Egger | -0.0955 | 0.3522 | 0.7930 | 0.91(0.46-1.81) |
| PTSD | genus | Faecalibacterium | Weighted median | -0.1174 | 0.2207 | 0.5946 | 0.89(0.58-1.37) |
| PTSD | genus | Faecalibacterium | IVW | -0.1063 | 0.1701 | 0.5320 | 0.90(0.64-1.25) |
| PTSD | genus | Faecalibacterium | Simple mode | -0.2276 | 0.3286 | 0.5061 | 0.80(0.42-1.52) |
| PTSD | genus | Faecalibacterium | Weighted mode | -0.1325 | 0.2350 | 0.5866 | 0.88(0.55-1.39) |
| PTSD | genus | FamilyXIIIAD3011group | MR Egger | -0.8728 | 0.7694 | 0.2808 | 0.42(0.09-1.89) |
| PTSD | genus | FamilyXIIIAD3011group | Weighted median | 0.2043 | 0.2135 | 0.3385 | 1.23(0.81-1.86) |
| PTSD | genus | FamilyXIIIAD3011group | IVW | 0.0507 | 0.1622 | 0.7547 | 1.05(0.77-1.45) |
| PTSD | genus | FamilyXIIIAD3011group | Simple mode | 0.2880 | 0.3774 | 0.4601 | 1.33(0.64-2.79) |
| PTSD | genus | FamilyXIIIAD3011group | Weighted mode | 0.2983 | 0.3515 | 0.4127 | 1.35(0.68-2.68) |
| PTSD | genus | FamilyXIIIUCG001 | MR Egger | -0.1412 | 0.5883 | 0.8183 | 0.87(0.27-2.75) |
| PTSD | genus | FamilyXIIIUCG001 | Weighted median | 0.1517 | 0.2429 | 0.5323 | 1.16(0.72-1.87) |
| PTSD | genus | FamilyXIIIUCG001 | IVW | 0.1981 | 0.1896 | 0.2961 | 1.22(0.84-1.77) |
| PTSD | genus | FamilyXIIIUCG001 | Simple mode | 0.1542 | 0.4037 | 0.7137 | 1.17(0.53-2.57) |
| PTSD | genus | FamilyXIIIUCG001 | Weighted mode | 0.1292 | 0.3861 | 0.7477 | 1.14(0.53-2.43) |
| PTSD | genus | Flavonifractor | MR Egger | 0.4261 | 0.8899 | 0.6648 | 1.53(0.27-8.76) |
| PTSD | genus | Flavonifractor | Weighted median | 0.2339 | 0.2869 | 0.4150 | 1.26(0.72-2.22) |
| PTSD | genus | Flavonifractor | IVW | 0.3355 | 0.2226 | 0.1317 | 1.40(0.90-2.16) |
| PTSD | genus | Flavonifractor | Simple mode | 0.1533 | 0.3467 | 0.6813 | 1.17(0.59-2.30) |
| PTSD | genus | Flavonifractor | Weighted mode | 0.1605 | 0.3520 | 0.6721 | 1.17(0.59-2.34) |
| PTSD | genus | Fusicatenibacter | MR Egger | 0.5891 | 0.5622 | 0.3093 | 1.80(0.60-5.42) |
| PTSD | genus | Fusicatenibacter | Weighted median | 0.2626 | 0.1997 | 0.1885 | 1.30(0.88-1.92) |
| PTSD | genus | Fusicatenibacter | IVW | 0.2225 | 0.1502 | 0.1385 | 1.25(0.93-1.68) |
| PTSD | genus | Fusicatenibacter | Simple mode | 0.4181 | 0.3948 | 0.3036 | 1.52(0.70-3.29) |
| PTSD | genus | Fusicatenibacter | Weighted mode | 0.4181 | 0.3945 | 0.3032 | 1.52(0.70-3.29) |
| PTSD | genus | Gordonibacter | MR Egger | 0.2335 | 0.4160 | 0.5884 | 1.26(0.56-2.85) |
| PTSD | genus | Gordonibacter | Weighted median | -0.0425 | 0.1282 | 0.7404 | 0.96(0.75-1.23) |
| PTSD | genus | Gordonibacter | IVW | -0.0637 | 0.0958 | 0.5061 | 0.94(0.78-1.13) |
| PTSD | genus | Gordonibacter | Simple mode | -0.0455 | 0.1995 | 0.8240 | 0.96(0.65-1.41) |
| PTSD | genus | Gordonibacter | Weighted mode | -0.0411 | 0.1848 | 0.8285 | 0.96(0.67-1.38) |
| PTSD | genus | Haemophilus | MR Egger | 0.2039 | 0.3036 | 0.5233 | 1.23(0.68-2.22) |
| PTSD | genus | Haemophilus | Weighted median | 0.2472 | 0.1817 | 0.1737 | 1.28(0.90-1.83) |
| PTSD | genus | Haemophilus | IVW | 0.2684 | 0.1354 | 0.0474 | 1.31(1.00-1.71) |
| PTSD | genus | Haemophilus | Simple mode | 0.2774 | 0.2911 | 0.3685 | 1.32(0.75-2.33) |
| PTSD | genus | Haemophilus | Weighted mode | 0.1827 | 0.3071 | 0.5684 | 1.20(0.66-2.19) |
| PTSD | genus | Holdemanella | MR Egger | 0.3714 | 0.4611 | 0.4412 | 1.45(0.59-3.58) |
| PTSD | genus | Holdemanella | Weighted median | 0.1724 | 0.1650 | 0.2963 | 1.19(0.86-1.64) |
| PTSD | genus | Holdemanella | IVW | 0.0355 | 0.1575 | 0.8217 | 1.04(0.76-1.41) |
| PTSD | genus | Holdemanella | Simple mode | 0.3385 | 0.2935 | 0.2756 | 1.40(0.79-2.49) |
| PTSD | genus | Holdemanella | Weighted mode | 0.3562 | 0.2851 | 0.2400 | 1.43(0.82-2.50) |
| PTSD | genus | Holdemania | MR Egger | -0.1286 | 0.3749 | 0.7375 | 0.88(0.42-1.83) |
| PTSD | genus | Holdemania | Weighted median | 0.1242 | 0.1725 | 0.4716 | 1.13(0.81-1.59) |
| PTSD | genus | Holdemania | IVW | 0.0350 | 0.1249 | 0.7795 | 1.04(0.81-1.32) |
| PTSD | genus | Holdemania | Simple mode | 0.1270 | 0.3325 | 0.7087 | 1.14(0.59-2.18) |
| PTSD | genus | Holdemania | Weighted mode | 0.1738 | 0.2870 | 0.5552 | 1.19(0.68-2.09) |
| PTSD | genus | Howardella | MR Egger | 0.5072 | 0.3729 | 0.2160 | 1.66(0.80-3.45) |
| PTSD | genus | Howardella | Weighted median | -0.0216 | 0.1198 | 0.8567 | 0.98(0.77-1.24) |
| PTSD | genus | Howardella | IVW | -0.0865 | 0.0938 | 0.3569 | 0.92(0.76-1.10) |
| PTSD | genus | Howardella | Simple mode | -0.0267 | 0.1718 | 0.8804 | 0.97(0.70-1.36) |
| PTSD | genus | Howardella | Weighted mode | -0.0184 | 0.1625 | 0.9126 | 0.98(0.71-1.35) |
| PTSD | genus | Hungatella | MR Egger | 0.6008 | 0.8724 | 0.5405 | 1.82(0.33-10.08) |
| PTSD | genus | Hungatella | Weighted median | 0.0080 | 0.1878 | 0.9658 | 1.01(0.70-1.46) |
| PTSD | genus | Hungatella | IVW | -0.0313 | 0.1439 | 0.8276 | 0.97(0.73-1.28) |
| PTSD | genus | Hungatella | Simple mode | 0.0706 | 0.2457 | 0.7883 | 1.07(0.66-1.74) |
| PTSD | genus | Hungatella | Weighted mode | 0.0867 | 0.2462 | 0.7425 | 1.09(0.67-1.77) |
| PTSD | genus | Intestinibacter | MR Egger | -0.3781 | 0.4436 | 0.4094 | 0.69(0.29-1.63) |
| PTSD | genus | Intestinibacter | Weighted median | -0.0461 | 0.1900 | 0.8084 | 0.95(0.66-1.39) |
| PTSD | genus | Intestinibacter | IVW | 0.0024 | 0.1365 | 0.9858 | 1.00(0.77-1.31) |
| PTSD | genus | Intestinibacter | Simple mode | -0.1933 | 0.2930 | 0.5202 | 0.82(0.46-1.46) |
| PTSD | genus | Intestinibacter | Weighted mode | -0.1622 | 0.2932 | 0.5887 | 0.85(0.48-1.51) |
| PTSD | genus | Intestinimonas | MR Egger | 0.1536 | 0.4176 | 0.7185 | 1.17(0.51-2.64) |
| PTSD | genus | Intestinimonas | Weighted median | -0.0299 | 0.1900 | 0.8748 | 0.97(0.67-1.41) |
| PTSD | genus | Intestinimonas | IVW | -0.0240 | 0.1445 | 0.8682 | 0.98(0.74-1.30) |
| PTSD | genus | Intestinimonas | Simple mode | 0.0412 | 0.3363 | 0.9041 | 1.04(0.54-2.01) |
| PTSD | genus | Intestinimonas | Weighted mode | 0.0095 | 0.3324 | 0.9777 | 1.01(0.53-1.94) |
| PTSD | genus | Lachnoclostridium | MR Egger | -0.3874 | 0.6321 | 0.5524 | 0.68(0.20-2.34) |
| PTSD | genus | Lachnoclostridium | Weighted median | -0.1605 | 0.2470 | 0.5157 | 0.85(0.52-1.38) |
| PTSD | genus | Lachnoclostridium | IVW | 0.1047 | 0.1836 | 0.5686 | 1.11(0.77-1.59) |
| PTSD | genus | Lachnoclostridium | Simple mode | -0.2610 | 0.4752 | 0.5929 | 0.77(0.30-1.96) |
| PTSD | genus | Lachnoclostridium | Weighted mode | -0.2851 | 0.4187 | 0.5088 | 0.75(0.33-1.71) |
| PTSD | genus | Lachnospira | MR Egger | 0.0985 | 1.6157 | 0.9543 | 1.10(0.05-26.19) |
| PTSD | genus | Lachnospira | Weighted median | -0.0786 | 0.3396 | 0.8171 | 0.92(0.48-1.80) |
| PTSD | genus | Lachnospira | IVW | -0.2417 | 0.2729 | 0.3757 | 0.79(0.46-1.34) |
| PTSD | genus | Lachnospira | Simple mode | 0.0190 | 0.4773 | 0.9698 | 1.02(0.40-2.60) |
| PTSD | genus | Lachnospira | Weighted mode | -0.0232 | 0.4381 | 0.9599 | 0.98(0.41-2.31) |
| PTSD | genus | LachnospiraceaeFCS020group | MR Egger | -0.1435 | 0.4191 | 0.7385 | 0.87(0.38-1.97) |
| PTSD | genus | LachnospiraceaeFCS020group | Weighted median | 0.0537 | 0.2024 | 0.7910 | 1.06(0.71-1.57) |
| PTSD | genus | LachnospiraceaeFCS020group | IVW | 0.0905 | 0.1515 | 0.5504 | 1.09(0.81-1.47) |
| PTSD | genus | LachnospiraceaeFCS020group | Simple mode | 0.2180 | 0.3294 | 0.5207 | 1.24(0.65-2.37) |
| PTSD | genus | LachnospiraceaeFCS020group | Weighted mode | 0.1357 | 0.3448 | 0.7008 | 1.15(0.58-2.25) |
| PTSD | genus | LachnospiraceaeNC2004group | MR Egger | -0.7904 | 0.5940 | 0.2250 | 0.45(0.14-1.45) |
| PTSD | genus | LachnospiraceaeNC2004group | Weighted median | 0.0809 | 0.1727 | 0.6396 | 1.08(0.77-1.52) |
| PTSD | genus | LachnospiraceaeNC2004group | IVW | -0.0116 | 0.1544 | 0.9401 | 0.99(0.73-1.34) |
| PTSD | genus | LachnospiraceaeNC2004group | Simple mode | 0.1341 | 0.2497 | 0.6059 | 1.14(0.70-1.87) |
| PTSD | genus | LachnospiraceaeNC2004group | Weighted mode | 0.0829 | 0.2162 | 0.7115 | 1.09(0.71-1.66) |
| PTSD | genus | LachnospiraceaeND3007group | MR Egger | -5.6183 | 6.1969 | 0.5312 | 0.00(0.00-683.80) |
| PTSD | genus | LachnospiraceaeND3007group | Weighted median | 0.2139 | 0.4743 | 0.6519 | 1.24(0.49-3.14) |
| PTSD | genus | LachnospiraceaeND3007group | IVW | 0.2750 | 0.3682 | 0.4551 | 1.32(0.64-2.71) |
| PTSD | genus | LachnospiraceaeND3007group | Simple mode | 0.0750 | 0.5330 | 0.9010 | 1.08(0.38-3.06) |
| PTSD | genus | LachnospiraceaeND3007group | Weighted mode | 0.1120 | 0.5388 | 0.8546 | 1.12(0.39-3.22) |
| PTSD | genus | LachnospiraceaeNK4A136group | MR Egger | -0.0018 | 0.3196 | 0.9956 | 1.00(0.53-1.87) |
| PTSD | genus | LachnospiraceaeNK4A136group | Weighted median | -0.0305 | 0.2212 | 0.8902 | 0.97(0.63-1.50) |
| PTSD | genus | LachnospiraceaeNK4A136group | IVW | -0.0440 | 0.1542 | 0.7755 | 0.96(0.71-1.29) |
| PTSD | genus | LachnospiraceaeNK4A136group | Simple mode | -0.5101 | 0.3834 | 0.2047 | 0.60(0.28-1.27) |
| PTSD | genus | LachnospiraceaeNK4A136group | Weighted mode | -0.0518 | 0.2260 | 0.8221 | 0.95(0.61-1.48) |
| PTSD | genus | LachnospiraceaeUCG001 | MR Egger | 0.9263 | 0.5614 | 0.1272 | 2.53(0.84-7.59) |
| PTSD | genus | LachnospiraceaeUCG001 | Weighted median | 0.1880 | 0.1804 | 0.2976 | 1.21(0.85-1.72) |
| PTSD | genus | LachnospiraceaeUCG001 | IVW | 0.0935 | 0.1296 | 0.4707 | 1.10(0.85-1.42) |
| PTSD | genus | LachnospiraceaeUCG001 | Simple mode | 0.1955 | 0.3131 | 0.5440 | 1.22(0.66-2.25) |
| PTSD | genus | LachnospiraceaeUCG001 | Weighted mode | 0.2664 | 0.3092 | 0.4059 | 1.31(0.71-2.39) |
| PTSD | genus | LachnospiraceaeUCG004 | MR Egger | -0.4245 | 0.8581 | 0.6305 | 0.65(0.12-3.52) |
| PTSD | genus | LachnospiraceaeUCG004 | Weighted median | 0.0867 | 0.2422 | 0.7205 | 1.09(0.68-1.75) |
| PTSD | genus | LachnospiraceaeUCG004 | IVW | 0.1465 | 0.1938 | 0.4497 | 1.16(0.79-1.69) |
| PTSD | genus | LachnospiraceaeUCG004 | Simple mode | 0.0030 | 0.3928 | 0.9941 | 1.00(0.46-2.17) |
| PTSD | genus | LachnospiraceaeUCG004 | Weighted mode | 0.0118 | 0.3961 | 0.9768 | 1.01(0.47-2.20) |
| PTSD | genus | LachnospiraceaeUCG008 | MR Egger | -0.1449 | 0.6165 | 0.8201 | 0.87(0.26-2.90) |
| PTSD | genus | LachnospiraceaeUCG008 | Weighted median | -0.1546 | 0.1518 | 0.3085 | 0.86(0.64-1.15) |
| PTSD | genus | LachnospiraceaeUCG008 | IVW | -0.1676 | 0.1227 | 0.1719 | 0.85(0.66-1.08) |
| PTSD | genus | LachnospiraceaeUCG008 | Simple mode | -0.1091 | 0.2358 | 0.6546 | 0.90(0.56-1.42) |
| PTSD | genus | LachnospiraceaeUCG008 | Weighted mode | -0.0869 | 0.2259 | 0.7093 | 0.92(0.59-1.43) |
| PTSD | genus | LachnospiraceaeUCG010 | MR Egger | 0.6316 | 0.5646 | 0.2958 | 1.88(0.62-5.69) |
| PTSD | genus | LachnospiraceaeUCG010 | Weighted median | 0.1454 | 0.2475 | 0.5569 | 1.16(0.71-1.88) |
| PTSD | genus | LachnospiraceaeUCG010 | IVW | 0.0774 | 0.1838 | 0.6738 | 1.08(0.75-1.55) |
| PTSD | genus | LachnospiraceaeUCG010 | Simple mode | 0.1956 | 0.3698 | 0.6097 | 1.22(0.59-2.51) |
| PTSD | genus | LachnospiraceaeUCG010 | Weighted mode | 0.1833 | 0.4253 | 0.6766 | 1.20(0.52-2.76) |
| PTSD | genus | Lactobacillus | MR Egger | -0.0578 | 0.4192 | 0.8948 | 0.94(0.41-2.15) |
| PTSD | genus | Lactobacillus | Weighted median | -0.2787 | 0.1823 | 0.1263 | 0.76(0.53-1.08) |
| PTSD | genus | Lactobacillus | IVW | -0.1522 | 0.1517 | 0.3158 | 0.86(0.64-1.16) |
| PTSD | genus | Lactobacillus | Simple mode | -0.2231 | 0.2873 | 0.4628 | 0.80(0.46-1.40) |
| PTSD | genus | Lactobacillus | Weighted mode | -0.2871 | 0.2303 | 0.2526 | 0.75(0.48-1.18) |
| PTSD | genus | Lactococcus | MR Egger | -0.4123 | 0.7327 | 0.5940 | 0.66(0.16-2.78) |
| PTSD | genus | Lactococcus | Weighted median | 0.2330 | 0.1610 | 0.1478 | 1.26(0.92-1.73) |
| PTSD | genus | Lactococcus | IVW | 0.1774 | 0.1564 | 0.2566 | 1.19(0.88-1.62) |
| PTSD | genus | Lactococcus | Simple mode | 0.2798 | 0.2411 | 0.2840 | 1.32(0.82-2.12) |
| PTSD | genus | Lactococcus | Weighted mode | 0.2901 | 0.2436 | 0.2726 | 1.34(0.83-2.15) |
| PTSD | genus | Marvinbryantia | MR Egger | -0.5295 | 0.6900 | 0.4649 | 0.59(0.15-2.28) |
| PTSD | genus | Marvinbryantia | Weighted median | 0.0243 | 0.2406 | 0.9197 | 1.02(0.64-1.64) |
| PTSD | genus | Marvinbryantia | IVW | -0.0416 | 0.1760 | 0.8131 | 0.96(0.68-1.35) |
| PTSD | genus | Marvinbryantia | Simple mode | 0.0358 | 0.3748 | 0.9261 | 1.04(0.50-2.16) |
| PTSD | genus | Marvinbryantia | Weighted mode | 0.0074 | 0.3165 | 0.9819 | 1.01(0.54-1.87) |
| PTSD | genus | Methanobrevibacter | MR Egger | 0.4487 | 0.5263 | 0.4420 | 1.57(0.56-4.39) |
| PTSD | genus | Methanobrevibacter | Weighted median | 0.1912 | 0.1758 | 0.2767 | 1.21(0.86-1.71) |
| PTSD | genus | Methanobrevibacter | IVW | 0.0960 | 0.1321 | 0.4676 | 1.10(0.85-1.43) |
| PTSD | genus | Methanobrevibacter | Simple mode | 0.2610 | 0.2524 | 0.3485 | 1.30(0.79-2.13) |
| PTSD | genus | Methanobrevibacter | Weighted mode | 0.2466 | 0.2490 | 0.3675 | 1.28(0.79-2.08) |
| PTSD | genus | Odoribacter | MR Egger | 0.4001 | 0.6757 | 0.5795 | 1.49(0.40-5.61) |
| PTSD | genus | Odoribacter | Weighted median | 0.1485 | 0.2754 | 0.5898 | 1.16(0.68-1.99) |
| PTSD | genus | Odoribacter | IVW | 0.1672 | 0.2166 | 0.4401 | 1.18(0.77-1.81) |
| PTSD | genus | Odoribacter | Simple mode | 0.2272 | 0.4098 | 0.5993 | 1.26(0.56-2.80) |
| PTSD | genus | Odoribacter | Weighted mode | 0.2053 | 0.3802 | 0.6087 | 1.23(0.58-2.59) |
| PTSD | genus | Olsenella | MR Egger | -0.2181 | 0.3895 | 0.5909 | 0.80(0.37-1.73) |
| PTSD | genus | Olsenella | Weighted median | -0.1199 | 0.1353 | 0.3756 | 0.89(0.68-1.16) |
| PTSD | genus | Olsenella | IVW | -0.1189 | 0.1149 | 0.3009 | 0.89(0.71-1.11) |
| PTSD | genus | Olsenella | Simple mode | -0.0791 | 0.2166 | 0.7234 | 0.92(0.60-1.41) |
| PTSD | genus | Olsenella | Weighted mode | -0.1197 | 0.1843 | 0.5323 | 0.89(0.62-1.27) |
| PTSD | genus | Oscillibacter | MR Egger | 0.3282 | 0.5894 | 0.5888 | 1.39(0.44-4.41) |
| PTSD | genus | Oscillibacter | Weighted median | -0.0263 | 0.1892 | 0.8897 | 0.97(0.67-1.41) |
| PTSD | genus | Oscillibacter | IVW | 0.0148 | 0.1500 | 0.9216 | 1.01(0.76-1.36) |
| PTSD | genus | Oscillibacter | Simple mode | -0.3529 | 0.3627 | 0.3498 | 0.70(0.35-1.43) |
| PTSD | genus | Oscillibacter | Weighted mode | -0.3191 | 0.3533 | 0.3842 | 0.73(0.36-1.45) |
| PTSD | genus | Oscillospira | MR Egger | -0.2308 | 0.7232 | 0.7604 | 0.79(0.19-3.28) |
| PTSD | genus | Oscillospira | Weighted median | 0.0633 | 0.2258 | 0.7791 | 1.07(0.68-1.66) |
| PTSD | genus | Oscillospira | IVW | 0.0976 | 0.1711 | 0.5686 | 1.10(0.79-1.54) |
| PTSD | genus | Oscillospira | Simple mode | -0.1445 | 0.3564 | 0.6972 | 0.87(0.43-1.74) |
| PTSD | genus | Oscillospira | Weighted mode | -0.1347 | 0.3335 | 0.6985 | 0.87(0.45-1.68) |
| PTSD | genus | Oxalobacter | MR Egger | 0.1540 | 0.4780 | 0.7546 | 1.17(0.46-2.98) |
| PTSD | genus | Oxalobacter | Weighted median | 0.0373 | 0.1328 | 0.7786 | 1.04(0.80-1.35) |
| PTSD | genus | Oxalobacter | IVW | -0.0356 | 0.0972 | 0.7138 | 0.96(0.80-1.17) |
| PTSD | genus | Oxalobacter | Simple mode | 0.0643 | 0.2593 | 0.8093 | 1.07(0.64-1.77) |
| PTSD | genus | Oxalobacter | Weighted mode | 0.0643 | 0.2444 | 0.7980 | 1.07(0.66-1.72) |
| PTSD | genus | Parabacteroides | MR Egger | 0.8730 | 1.6779 | 0.6388 | 2.39(0.09-64.18) |
| PTSD | genus | Parabacteroides | Weighted median | -0.1583 | 0.3564 | 0.6569 | 0.85(0.42-1.72) |
| PTSD | genus | Parabacteroides | IVW | -0.0362 | 0.2692 | 0.8930 | 0.96(0.57-1.63) |
| PTSD | genus | Parabacteroides | Simple mode | -0.2740 | 0.5016 | 0.6140 | 0.76(0.28-2.03) |
| PTSD | genus | Parabacteroides | Weighted mode | -0.2599 | 0.4516 | 0.5958 | 0.77(0.32-1.87) |
| PTSD | genus | Paraprevotella | MR Egger | 0.4840 | 0.4032 | 0.2551 | 1.62(0.74-3.58) |
| PTSD | genus | Paraprevotella | Weighted median | -0.0757 | 0.1470 | 0.6067 | 0.93(0.69-1.24) |
| PTSD | genus | Paraprevotella | IVW | -0.0319 | 0.1086 | 0.7689 | 0.97(0.78-1.20) |
| PTSD | genus | Paraprevotella | Simple mode | -0.2114 | 0.2424 | 0.4002 | 0.81(0.50-1.30) |
| PTSD | genus | Paraprevotella | Weighted mode | -0.2014 | 0.2242 | 0.3867 | 0.82(0.53-1.27) |
| PTSD | genus | Parasutterella | MR Egger | -0.2798 | 0.3738 | 0.4685 | 0.76(0.36-1.57) |
| PTSD | genus | Parasutterella | Weighted median | -0.3492 | 0.1860 | 0.0605 | 0.71(0.49-1.02) |
| PTSD | genus | Parasutterella | IVW | -0.2453 | 0.1295 | 0.0583 | 0.78(0.61-1.01) |
| PTSD | genus | Parasutterella | Simple mode | -0.3857 | 0.2933 | 0.2112 | 0.68(0.38-1.21) |
| PTSD | genus | Parasutterella | Weighted mode | -0.3928 | 0.2479 | 0.1372 | 0.68(0.42-1.10) |
| PTSD | genus | Peptococcus | MR Egger | 0.6007 | 0.3778 | 0.1401 | 1.82(0.87-3.82) |
| PTSD | genus | Peptococcus | Weighted median | 0.1132 | 0.1380 | 0.4122 | 1.12(0.85-1.47) |
| PTSD | genus | Peptococcus | IVW | 0.0499 | 0.0990 | 0.6144 | 1.05(0.87-1.28) |
| PTSD | genus | Peptococcus | Simple mode | 0.1161 | 0.2442 | 0.6431 | 1.12(0.70-1.81) |
| PTSD | genus | Peptococcus | Weighted mode | 0.1362 | 0.2283 | 0.5618 | 1.15(0.73-1.79) |
| PTSD | genus | Phascolarctobacterium | MR Egger | -1.3421 | 0.7724 | 0.1258 | 0.26(0.06-1.19) |
| PTSD | genus | Phascolarctobacterium | Weighted median | -0.2581 | 0.2229 | 0.2471 | 0.77(0.50-1.20) |
| PTSD | genus | Phascolarctobacterium | IVW | 0.0055 | 0.1713 | 0.9743 | 1.01(0.72-1.41) |
| PTSD | genus | Phascolarctobacterium | Simple mode | -0.3416 | 0.3807 | 0.3958 | 0.71(0.34-1.50) |
| PTSD | genus | Phascolarctobacterium | Weighted mode | -0.3416 | 0.3737 | 0.3874 | 0.71(0.34-1.48) |
| PTSD | genus | Prevotella7 | MR Egger | -0.1994 | 0.6289 | 0.7584 | 0.82(0.24-2.81) |
| PTSD | genus | Prevotella7 | Weighted median | 0.1318 | 0.1280 | 0.3034 | 1.14(0.89-1.47) |
| PTSD | genus | Prevotella7 | IVW | 0.0031 | 0.1039 | 0.9759 | 1.00(0.82-1.23) |
| PTSD | genus | Prevotella7 | Simple mode | 0.2038 | 0.2137 | 0.3628 | 1.23(0.81-1.86) |
| PTSD | genus | Prevotella7 | Weighted mode | 0.1911 | 0.2203 | 0.4059 | 1.21(0.79-1.86) |
| PTSD | genus | Prevotella9 | MR Egger | 0.3490 | 0.4249 | 0.4262 | 1.42(0.62-3.26) |
| PTSD | genus | Prevotella9 | Weighted median | -0.0153 | 0.1858 | 0.9346 | 0.98(0.68-1.42) |
| PTSD | genus | Prevotella9 | IVW | -0.1059 | 0.1476 | 0.4730 | 0.90(0.67-1.20) |
| PTSD | genus | Prevotella9 | Simple mode | 0.0093 | 0.3634 | 0.9799 | 1.01(0.50-2.06) |
| PTSD | genus | Prevotella9 | Weighted mode | 0.1674 | 0.2782 | 0.5571 | 1.18(0.69-2.04) |
| PTSD | genus | RikenellaceaeRC9gutgroup | MR Egger | -0.5506 | 0.7253 | 0.4672 | 0.58(0.14-2.39) |
| PTSD | genus | RikenellaceaeRC9gutgroup | Weighted median | 0.0287 | 0.1219 | 0.8140 | 1.03(0.81-1.31) |
| PTSD | genus | RikenellaceaeRC9gutgroup | IVW | -0.0568 | 0.1131 | 0.6158 | 0.94(0.76-1.18) |
| PTSD | genus | RikenellaceaeRC9gutgroup | Simple mode | 0.0193 | 0.1713 | 0.9127 | 1.02(0.73-1.43) |
| PTSD | genus | RikenellaceaeRC9gutgroup | Weighted mode | 0.0271 | 0.1734 | 0.8792 | 1.03(0.73-1.44) |
| PTSD | genus | Romboutsia | MR Egger | 0.1785 | 0.4843 | 0.7188 | 1.20(0.46-3.09) |
| PTSD | genus | Romboutsia | Weighted median | -0.1791 | 0.2102 | 0.3943 | 0.84(0.55-1.26) |
| PTSD | genus | Romboutsia | IVW | -0.1486 | 0.1595 | 0.3517 | 0.86(0.63-1.18) |
| PTSD | genus | Romboutsia | Simple mode | -0.2280 | 0.3284 | 0.4997 | 0.80(0.42-1.52) |
| PTSD | genus | Romboutsia | Weighted mode | -0.2206 | 0.3278 | 0.5128 | 0.80(0.42-1.52) |
| PTSD | genus | Roseburia | MR Egger | -0.8333 | 0.6148 | 0.2003 | 0.43(0.13-1.45) |
| PTSD | genus | Roseburia | Weighted median | -0.1936 | 0.2647 | 0.4647 | 0.82(0.49-1.38) |
| PTSD | genus | Roseburia | IVW | -0.0502 | 0.2053 | 0.8067 | 0.95(0.64-1.42) |
| PTSD | genus | Roseburia | Simple mode | -0.4923 | 0.4994 | 0.3422 | 0.61(0.23-1.63) |
| PTSD | genus | Roseburia | Weighted mode | -0.4734 | 0.4662 | 0.3283 | 0.62(0.25-1.55) |
| PTSD | genus | Ruminiclostridium5 | MR Egger | 0.1898 | 1.0500 | 0.8606 | 1.21(0.15-9.47) |
| PTSD | genus | Ruminiclostridium5 | Weighted median | 0.0981 | 0.2782 | 0.7243 | 1.10(0.64-1.90) |
| PTSD | genus | Ruminiclostridium5 | IVW | -0.1452 | 0.2398 | 0.5450 | 0.86(0.54-1.38) |
| PTSD | genus | Ruminiclostridium5 | Simple mode | 0.4048 | 0.4591 | 0.3985 | 1.50(0.61-3.69) |
| PTSD | genus | Ruminiclostridium5 | Weighted mode | 0.3560 | 0.4862 | 0.4809 | 1.43(0.55-3.70) |
| PTSD | genus | Ruminiclostridium6 | MR Egger | -0.4244 | 0.3683 | 0.2700 | 0.65(0.32-1.35) |
| PTSD | genus | Ruminiclostridium6 | Weighted median | 0.2414 | 0.2184 | 0.2690 | 1.27(0.83-1.95) |
| PTSD | genus | Ruminiclostridium6 | IVW | 0.1198 | 0.1490 | 0.4213 | 1.13(0.84-1.51) |
| PTSD | genus | Ruminiclostridium6 | Simple mode | 0.3479 | 0.3949 | 0.3932 | 1.42(0.65-3.07) |
| PTSD | genus | Ruminiclostridium6 | Weighted mode | 0.3540 | 0.4358 | 0.4302 | 1.42(0.61-3.35) |
| PTSD | genus | Ruminiclostridium9 | MR Egger | -0.4024 | 1.3002 | 0.7674 | 0.67(0.05-8.55) |
| PTSD | genus | Ruminiclostridium9 | Weighted median | -0.2130 | 0.3034 | 0.4826 | 0.81(0.45-1.46) |
| PTSD | genus | Ruminiclostridium9 | IVW | -0.0779 | 0.2529 | 0.7581 | 0.93(0.56-1.52) |
| PTSD | genus | Ruminiclostridium9 | Simple mode | -0.4234 | 0.4820 | 0.4088 | 0.65(0.25-1.68) |
| PTSD | genus | Ruminiclostridium9 | Weighted mode | -0.3588 | 0.5429 | 0.5299 | 0.70(0.24-2.02) |
| PTSD | genus | RuminococcaceaeNK4A214group | MR Egger | -0.2566 | 0.6140 | 0.6841 | 0.77(0.23-2.58) |
| PTSD | genus | RuminococcaceaeNK4A214group | Weighted median | 0.4102 | 0.2402 | 0.0877 | 1.51(0.94-2.41) |
| PTSD | genus | RuminococcaceaeNK4A214group | IVW | 0.1767 | 0.1826 | 0.3331 | 1.19(0.83-1.71) |
| PTSD | genus | RuminococcaceaeNK4A214group | Simple mode | 0.4660 | 0.4191 | 0.2880 | 1.59(0.70-3.62) |
| PTSD | genus | RuminococcaceaeNK4A214group | Weighted mode | 0.4856 | 0.4723 | 0.3242 | 1.63(0.64-4.10) |
| PTSD | genus | RuminococcaceaeUCG002 | MR Egger | 0.1451 | 0.3306 | 0.6659 | 1.16(0.60-2.21) |
| PTSD | genus | RuminococcaceaeUCG002 | Weighted median | 0.0712 | 0.1845 | 0.6997 | 1.07(0.75-1.54) |
| PTSD | genus | RuminococcaceaeUCG002 | IVW | 0.1279 | 0.1277 | 0.3166 | 1.14(0.88-1.46) |
| PTSD | genus | RuminococcaceaeUCG002 | Simple mode | 0.2370 | 0.3338 | 0.4863 | 1.27(0.66-2.44) |
| PTSD | genus | RuminococcaceaeUCG002 | Weighted mode | -0.0535 | 0.2434 | 0.8285 | 0.95(0.59-1.53) |
| PTSD | genus | RuminococcaceaeUCG003 | MR Egger | 0.4197 | 0.6940 | 0.5588 | 1.52(0.39-5.93) |
| PTSD | genus | RuminococcaceaeUCG003 | Weighted median | -0.2325 | 0.2278 | 0.3073 | 0.79(0.51-1.24) |
| PTSD | genus | RuminococcaceaeUCG003 | IVW | -0.2084 | 0.2099 | 0.3207 | 0.81(0.54-1.23) |
| PTSD | genus | RuminococcaceaeUCG003 | Simple mode | -0.3892 | 0.3774 | 0.3245 | 0.68(0.32-1.42) |
| PTSD | genus | RuminococcaceaeUCG003 | Weighted mode | -0.3221 | 0.3243 | 0.3420 | 0.72(0.38-1.37) |
| PTSD | genus | RuminococcaceaeUCG004 | MR Egger | -1.6827 | 0.7890 | 0.0617 | 0.19(0.04-0.87) |
| PTSD | genus | RuminococcaceaeUCG004 | Weighted median | 0.1063 | 0.1856 | 0.5667 | 1.11(0.77-1.60) |
| PTSD | genus | RuminococcaceaeUCG004 | IVW | 0.0037 | 0.1433 | 0.9794 | 1.00(0.76-1.33) |
| PTSD | genus | RuminococcaceaeUCG004 | Simple mode | 0.2024 | 0.3089 | 0.5271 | 1.22(0.67-2.24) |
| PTSD | genus | RuminococcaceaeUCG004 | Weighted mode | 0.1966 | 0.3020 | 0.5298 | 1.22(0.67-2.20) |
| PTSD | genus | RuminococcaceaeUCG005 | MR Egger | -0.2308 | 0.5197 | 0.6649 | 0.79(0.29-2.20) |
| PTSD | genus | RuminococcaceaeUCG005 | Weighted median | 0.0677 | 0.2159 | 0.7539 | 1.07(0.70-1.63) |
| PTSD | genus | RuminococcaceaeUCG005 | IVW | 0.0733 | 0.1875 | 0.6961 | 1.08(0.75-1.55) |
| PTSD | genus | RuminococcaceaeUCG005 | Simple mode | 0.3009 | 0.3739 | 0.4354 | 1.35(0.65-2.81) |
| PTSD | genus | RuminococcaceaeUCG005 | Weighted mode | 0.2660 | 0.3361 | 0.4429 | 1.30(0.68-2.52) |
| PTSD | genus | RuminococcaceaeUCG009 | MR Egger | -0.4400 | 0.6448 | 0.5121 | 0.64(0.18-2.28) |
| PTSD | genus | RuminococcaceaeUCG009 | Weighted median | 0.2904 | 0.1768 | 0.1004 | 1.34(0.95-1.89) |
| PTSD | genus | RuminococcaceaeUCG009 | IVW | 0.1412 | 0.1672 | 0.3985 | 1.15(0.83-1.60) |
| PTSD | genus | RuminococcaceaeUCG009 | Simple mode | 0.4069 | 0.3037 | 0.2101 | 1.50(0.83-2.72) |
| PTSD | genus | RuminococcaceaeUCG009 | Weighted mode | 0.3773 | 0.2925 | 0.2261 | 1.46(0.82-2.59) |
| PTSD | genus | RuminococcaceaeUCG010 | MR Egger | -0.2205 | 0.6549 | 0.7533 | 0.80(0.22-2.90) |
| PTSD | genus | RuminococcaceaeUCG010 | Weighted median | -0.1794 | 0.2755 | 0.5150 | 0.84(0.49-1.43) |
| PTSD | genus | RuminococcaceaeUCG010 | IVW | -0.2532 | 0.2135 | 0.2356 | 0.78(0.51-1.18) |
| PTSD | genus | RuminococcaceaeUCG010 | Simple mode | 0.0529 | 0.3668 | 0.8910 | 1.05(0.51-2.16) |
| PTSD | genus | RuminococcaceaeUCG010 | Weighted mode | -0.1776 | 0.3237 | 0.6068 | 0.84(0.44-1.58) |
| PTSD | genus | RuminococcaceaeUCG011 | MR Egger | -0.4311 | 0.5037 | 0.4249 | 0.65(0.24-1.74) |
| PTSD | genus | RuminococcaceaeUCG011 | Weighted median | -0.1489 | 0.1337 | 0.2653 | 0.86(0.66-1.12) |
| PTSD | genus | RuminococcaceaeUCG011 | IVW | -0.0839 | 0.1008 | 0.4056 | 0.92(0.75-1.12) |
| PTSD | genus | RuminococcaceaeUCG011 | Simple mode | -0.1797 | 0.1980 | 0.3943 | 0.84(0.57-1.23) |
| PTSD | genus | RuminococcaceaeUCG011 | Weighted mode | -0.1971 | 0.1844 | 0.3205 | 0.82(0.57-1.18) |
| PTSD | genus | RuminococcaceaeUCG013 | MR Egger | -0.3745 | 0.6517 | 0.5796 | 0.69(0.19-2.47) |
| PTSD | genus | RuminococcaceaeUCG013 | Weighted median | -0.4837 | 0.2446 | 0.0480 | 0.62(0.38-1.00) |
| PTSD | genus | RuminococcaceaeUCG013 | IVW | -0.2060 | 0.2149 | 0.3378 | 0.81(0.53-1.24) |
| PTSD | genus | RuminococcaceaeUCG013 | Simple mode | -0.5552 | 0.3437 | 0.1374 | 0.57(0.29-1.13) |
| PTSD | genus | RuminococcaceaeUCG013 | Weighted mode | -0.5552 | 0.3182 | 0.1117 | 0.57(0.31-1.07) |
| PTSD | genus | RuminococcaceaeUCG014 | MR Egger | -0.4005 | 0.3741 | 0.3156 | 0.67(0.32-1.39) |
| PTSD | genus | RuminococcaceaeUCG014 | Weighted median | -0.1567 | 0.2284 | 0.4927 | 0.85(0.55-1.34) |
| PTSD | genus | RuminococcaceaeUCG014 | IVW | -0.0627 | 0.1625 | 0.6997 | 0.94(0.68-1.29) |
| PTSD | genus | RuminococcaceaeUCG014 | Simple mode | -0.2547 | 0.3444 | 0.4784 | 0.78(0.39-1.52) |
| PTSD | genus | RuminococcaceaeUCG014 | Weighted mode | -0.2049 | 0.2424 | 0.4199 | 0.81(0.51-1.31) |
| PTSD | genus | Ruminococcus1 | MR Egger | 0.8063 | 0.7022 | 0.2841 | 2.24(0.57-8.87) |
| PTSD | genus | Ruminococcus1 | Weighted median | 0.2048 | 0.2781 | 0.4615 | 1.23(0.71-2.12) |
| PTSD | genus | Ruminococcus1 | IVW | -0.0556 | 0.2748 | 0.8396 | 0.95(0.55-1.62) |
| PTSD | genus | Ruminococcus1 | Simple mode | 0.4619 | 0.5015 | 0.3810 | 1.59(0.59-4.24) |
| PTSD | genus | Ruminococcus1 | Weighted mode | 0.5080 | 0.4822 | 0.3196 | 1.66(0.65-4.28) |
| PTSD | genus | Ruminococcus2 | MR Egger | 0.4885 | 0.4144 | 0.2596 | 1.63(0.72-3.67) |
| PTSD | genus | Ruminococcus2 | Weighted median | 0.1598 | 0.2206 | 0.4689 | 1.17(0.76-1.81) |
| PTSD | genus | Ruminococcus2 | IVW | -0.2068 | 0.1857 | 0.2654 | 0.81(0.57-1.17) |
| PTSD | genus | Ruminococcus2 | Simple mode | 0.1397 | 0.4457 | 0.7586 | 1.15(0.48-2.75) |
| PTSD | genus | Ruminococcus2 | Weighted mode | 0.2923 | 0.2655 | 0.2894 | 1.34(0.80-2.25) |
| PTSD | genus | Sellimonas | MR Egger | -0.1064 | 0.4859 | 0.8328 | 0.90(0.35-2.33) |
| PTSD | genus | Sellimonas | Weighted median | -0.0866 | 0.1213 | 0.4753 | 0.92(0.72-1.16) |
| PTSD | genus | Sellimonas | IVW | -0.1781 | 0.0887 | 0.0446 | 0.84(0.70-1.00) |
| PTSD | genus | Sellimonas | Simple mode | -0.0738 | 0.1979 | 0.7190 | 0.93(0.63-1.37) |
| PTSD | genus | Sellimonas | Weighted mode | -0.0710 | 0.2034 | 0.7359 | 0.93(0.63-1.39) |
| PTSD | genus | Senegalimassilia | MR Egger | 0.1153 | 0.9876 | 0.9145 | 1.12(0.16-7.78) |
| PTSD | genus | Senegalimassilia | Weighted median | 0.0970 | 0.2635 | 0.7128 | 1.10(0.66-1.85) |
| PTSD | genus | Senegalimassilia | IVW | -0.0131 | 0.2233 | 0.9532 | 0.99(0.64-1.53) |
| PTSD | genus | Senegalimassilia | Simple mode | 0.2162 | 0.3235 | 0.5405 | 1.24(0.66-2.34) |
| PTSD | genus | Senegalimassilia | Weighted mode | 0.1845 | 0.3050 | 0.5778 | 1.20(0.66-2.19) |
| PTSD | genus | Slackia | MR Egger | -0.5659 | 1.0745 | 0.6263 | 0.57(0.07-4.66) |
| PTSD | genus | Slackia | Weighted median | 0.2706 | 0.2061 | 0.1892 | 1.31(0.88-1.96) |
| PTSD | genus | Slackia | IVW | 0.2170 | 0.1643 | 0.1866 | 1.24(0.90-1.71) |
| PTSD | genus | Slackia | Simple mode | 0.3313 | 0.2777 | 0.2863 | 1.39(0.81-2.40) |
| PTSD | genus | Slackia | Weighted mode | 0.3282 | 0.2734 | 0.2837 | 1.39(0.81-2.37) |
| PTSD | genus | Streptococcus | MR Egger | 0.5450 | 0.6648 | 0.4297 | 1.72(0.47-6.35) |
| PTSD | genus | Streptococcus | Weighted median | 0.0167 | 0.2495 | 0.9465 | 1.02(0.62-1.66) |
| PTSD | genus | Streptococcus | IVW | 0.0661 | 0.1743 | 0.7044 | 1.07(0.76-1.50) |
| PTSD | genus | Streptococcus | Simple mode | 0.5670 | 0.4694 | 0.2504 | 1.76(0.70-4.42) |
| PTSD | genus | Streptococcus | Weighted mode | -0.1184 | 0.4405 | 0.7927 | 0.89(0.37-2.11) |
| PTSD | genus | Subdoligranulum | MR Egger | -0.8537 | 0.4621 | 0.0978 | 0.43(0.17-1.05) |
| PTSD | genus | Subdoligranulum | Weighted median | 0.0051 | 0.2457 | 0.9833 | 1.01(0.62-1.63) |
| PTSD | genus | Subdoligranulum | IVW | -0.0746 | 0.1785 | 0.6758 | 0.93(0.65-1.32) |
| PTSD | genus | Subdoligranulum | Simple mode | 0.4069 | 0.4368 | 0.3735 | 1.50(0.64-3.54) |
| PTSD | genus | Subdoligranulum | Weighted mode | 0.2750 | 0.3725 | 0.4774 | 1.32(0.63-2.73) |
| PTSD | genus | Sutterella | MR Egger | -0.1319 | 0.7017 | 0.8547 | 0.88(0.22-3.47) |
| PTSD | genus | Sutterella | Weighted median | -0.0230 | 0.2180 | 0.9161 | 0.98(0.64-1.50) |
| PTSD | genus | Sutterella | IVW | -0.0335 | 0.1619 | 0.8359 | 0.97(0.70-1.33) |
| PTSD | genus | Sutterella | Simple mode | -0.0532 | 0.3740 | 0.8894 | 0.95(0.46-1.97) |
| PTSD | genus | Sutterella | Weighted mode | -0.0402 | 0.3693 | 0.9153 | 0.96(0.47-1.98) |
| PTSD | genus | Terrisporobacter | MR Egger | 0.1001 | 0.6721 | 0.8911 | 1.11(0.30-4.13) |
| PTSD | genus | Terrisporobacter | Weighted median | -0.1021 | 0.2593 | 0.6938 | 0.90(0.54-1.50) |
| PTSD | genus | Terrisporobacter | IVW | -0.1561 | 0.1986 | 0.4320 | 0.86(0.58-1.26) |
| PTSD | genus | Terrisporobacter | Simple mode | 0.1272 | 0.3729 | 0.7502 | 1.14(0.55-2.36) |
| PTSD | genus | Terrisporobacter | Weighted mode | 0.0445 | 0.3627 | 0.9083 | 1.05(0.51-2.13) |
| PTSD | genus | Turicibacter | MR Egger | -0.4070 | 0.6135 | 0.5284 | 0.67(0.20-2.22) |
| PTSD | genus | Turicibacter | Weighted median | 0.2774 | 0.2152 | 0.1974 | 1.32(0.87-2.01) |
| PTSD | genus | Turicibacter | IVW | 0.2706 | 0.1519 | 0.0749 | 1.31(0.97-1.77) |
| PTSD | genus | Turicibacter | Simple mode | 0.2622 | 0.3311 | 0.4513 | 1.30(0.68-2.49) |
| PTSD | genus | Turicibacter | Weighted mode | 0.3083 | 0.2987 | 0.3322 | 1.36(0.76-2.44) |
| PTSD | genus | Tyzzerella3 | MR Egger | -0.0675 | 0.5831 | 0.9102 | 0.93(0.30-2.93) |
| PTSD | genus | Tyzzerella3 | Weighted median | -0.0386 | 0.1278 | 0.7625 | 0.96(0.75-1.24) |
| PTSD | genus | Tyzzerella3 | IVW | -0.0364 | 0.1045 | 0.7279 | 0.96(0.79-1.18) |
| PTSD | genus | Tyzzerella3 | Simple mode | -0.0472 | 0.2141 | 0.8295 | 0.95(0.63-1.45) |
| PTSD | genus | Tyzzerella3 | Weighted mode | -0.0472 | 0.1978 | 0.8157 | 0.95(0.65-1.41) |
| PTSD | genus | Veillonella | MR Egger | 7.5602 | 5.8199 | 0.2848 | 1920.24(0.02-172738172.74) |
| PTSD | genus | Veillonella | Weighted median | -0.0334 | 0.3058 | 0.9131 | 0.97(0.53-1.76) |
| PTSD | genus | Veillonella | IVW | -0.0267 | 0.2723 | 0.9218 | 0.97(0.57-1.66) |
| PTSD | genus | Veillonella | Simple mode | 0.2706 | 0.4533 | 0.5827 | 1.31(0.54-3.19) |
| PTSD | genus | Veillonella | Weighted mode | 0.2554 | 0.4437 | 0.5957 | 1.29(0.54-3.08) |
| PTSD | genus | Victivallis | MR Egger | 0.0255 | 0.5756 | 0.9656 | 1.03(0.33-3.17) |
| PTSD | genus | Victivallis | Weighted median | -0.0907 | 0.1232 | 0.4617 | 0.91(0.72-1.16) |
| PTSD | genus | Victivallis | IVW | -0.1004 | 0.0886 | 0.2567 | 0.90(0.76-1.08) |
| PTSD | genus | Victivallis | Simple mode | -0.2587 | 0.1980 | 0.2206 | 0.77(0.52-1.14) |
| PTSD | genus | Victivallis | Weighted mode | -0.2559 | 0.1835 | 0.1933 | 0.77(0.54-1.11) |
| PTSD | order | Actinomycetales | MR Egger | -0.2167 | 0.4296 | 0.6641 | 0.81(0.35-1.87) |
| PTSD | order | Actinomycetales | Weighted median | 0.0558 | 0.2345 | 0.8118 | 1.06(0.67-1.67) |
| PTSD | order | Actinomycetales | IVW | 0.1259 | 0.1967 | 0.5220 | 1.13(0.77-1.67) |
| PTSD | order | Actinomycetales | Simple mode | 0.0041 | 0.3293 | 0.9907 | 1.00(0.53-1.91) |
| PTSD | order | Actinomycetales | Weighted mode | -0.0052 | 0.2482 | 0.9847 | 0.99(0.61-1.62) |
| PTSD | order | Bacillales | MR Egger | -0.2874 | 0.3928 | 0.4918 | 0.75(0.35-1.62) |
| PTSD | order | Bacillales | Weighted median | -0.1108 | 0.1262 | 0.3798 | 0.90(0.70-1.15) |
| PTSD | order | Bacillales | IVW | -0.0635 | 0.0978 | 0.5165 | 0.94(0.77-1.14) |
| PTSD | order | Bacillales | Simple mode | -0.1720 | 0.1956 | 0.4084 | 0.84(0.57-1.24) |
| PTSD | order | Bacillales | Weighted mode | -0.1495 | 0.1886 | 0.4538 | 0.86(0.60-1.25) |
| PTSD | order | Bacteroidales | MR Egger | -0.3534 | 0.4346 | 0.4351 | 0.70(0.30-1.65) |
| PTSD | order | Bacteroidales | Weighted median | -0.1994 | 0.2712 | 0.4622 | 0.82(0.48-1.39) |
| PTSD | order | Bacteroidales | IVW | -0.2141 | 0.1877 | 0.2540 | 0.81(0.56-1.17) |
| PTSD | order | Bacteroidales | Simple mode | 0.1842 | 0.4543 | 0.6930 | 1.20(0.49-2.93) |
| PTSD | order | Bacteroidales | Weighted mode | -0.6966 | 0.4084 | 0.1161 | 0.50(0.22-1.11) |
| PTSD | order | Bifidobacteriales | MR Egger | 0.6729 | 0.5076 | 0.2096 | 1.96(0.72-5.30) |
| PTSD | order | Bifidobacteriales | Weighted median | -0.0691 | 0.1989 | 0.7283 | 0.93(0.63-1.38) |
| PTSD | order | Bifidobacteriales | IVW | -0.1878 | 0.1437 | 0.1912 | 0.83(0.63-1.10) |
| PTSD | order | Bifidobacteriales | Simple mode | -0.5792 | 0.3620 | 0.1336 | 0.56(0.28-1.14) |
| PTSD | order | Bifidobacteriales | Weighted mode | 0.1615 | 0.2593 | 0.5442 | 1.18(0.71-1.95) |
| PTSD | order | Burkholderiales | MR Egger | -0.2653 | 0.6141 | 0.6772 | 0.77(0.23-2.56) |
| PTSD | order | Burkholderiales | Weighted median | 0.0635 | 0.2361 | 0.7879 | 1.07(0.67-1.69) |
| PTSD | order | Burkholderiales | IVW | 0.2493 | 0.1945 | 0.2000 | 1.28(0.88-1.88) |
| PTSD | order | Burkholderiales | Simple mode | 0.0276 | 0.3855 | 0.9446 | 1.03(0.48-2.19) |
| PTSD | order | Burkholderiales | Weighted mode | 0.0225 | 0.3890 | 0.9551 | 1.02(0.48-2.19) |
| PTSD | order | Clostridiales | MR Egger | -1.4484 | 0.9743 | 0.1713 | 0.23(0.03-1.59) |
| PTSD | order | Clostridiales | Weighted median | -0.1758 | 0.2734 | 0.5202 | 0.84(0.49-1.43) |
| PTSD | order | Clostridiales | IVW | -0.2485 | 0.2411 | 0.3027 | 0.78(0.49-1.25) |
| PTSD | order | Clostridiales | Simple mode | -0.1317 | 0.4307 | 0.7661 | 0.88(0.38-2.04) |
| PTSD | order | Clostridiales | Weighted mode | -0.2339 | 0.3906 | 0.5627 | 0.79(0.37-1.70) |
| PTSD | order | Coriobacteriales | MR Egger | -0.2990 | 0.6273 | 0.6401 | 0.74(0.22-2.54) |
| PTSD | order | Coriobacteriales | Weighted median | -0.0928 | 0.2039 | 0.6492 | 0.91(0.61-1.36) |
| PTSD | order | Coriobacteriales | IVW | -0.0698 | 0.1524 | 0.6470 | 0.93(0.69-1.26) |
| PTSD | order | Coriobacteriales | Simple mode | -0.2179 | 0.3645 | 0.5579 | 0.80(0.39-1.64) |
| PTSD | order | Coriobacteriales | Weighted mode | -0.2111 | 0.3694 | 0.5751 | 0.81(0.39-1.67) |
| PTSD | order | Desulfovibrionales | MR Egger | -0.5900 | 0.7956 | 0.4772 | 0.55(0.12-2.64) |
| PTSD | order | Desulfovibrionales | Weighted median | -0.0508 | 0.2516 | 0.8399 | 0.95(0.58-1.56) |
| PTSD | order | Desulfovibrionales | IVW | -0.0348 | 0.1864 | 0.8521 | 0.97(0.67-1.39) |
| PTSD | order | Desulfovibrionales | Simple mode | -0.0525 | 0.3663 | 0.8889 | 0.95(0.46-1.95) |
| PTSD | order | Desulfovibrionales | Weighted mode | -0.1104 | 0.3350 | 0.7485 | 0.90(0.46-1.73) |
| PTSD | order | Enterobacteriales | MR Egger | 1.0696 | 1.3443 | 0.4623 | 2.91(0.21-40.63) |
| PTSD | order | Enterobacteriales | Weighted median | 0.1372 | 0.2709 | 0.6125 | 1.15(0.67-1.95) |
| PTSD | order | Enterobacteriales | IVW | 0.2124 | 0.2245 | 0.3441 | 1.24(0.80-1.92) |
| PTSD | order | Enterobacteriales | Simple mode | 0.0814 | 0.4114 | 0.8497 | 1.08(0.48-2.43) |
| PTSD | order | Enterobacteriales | Weighted mode | 0.0814 | 0.4256 | 0.8547 | 1.08(0.47-2.50) |
| PTSD | order | Erysipelotrichales | MR Egger | 0.9393 | 0.8472 | 0.2912 | 2.56(0.49-13.46) |
| PTSD | order | Erysipelotrichales | Weighted median | -0.0673 | 0.2747 | 0.8065 | 0.93(0.55-1.60) |
| PTSD | order | Erysipelotrichales | IVW | -0.3389 | 0.2068 | 0.1013 | 0.71(0.48-1.07) |
| PTSD | order | Erysipelotrichales | Simple mode | -0.1159 | 0.4724 | 0.8104 | 0.89(0.35-2.25) |
| PTSD | order | Erysipelotrichales | Weighted mode | -0.0968 | 0.4636 | 0.8382 | 0.91(0.37-2.25) |
| PTSD | order | Gastranaerophilales | MR Egger | 0.0867 | 0.5272 | 0.8741 | 1.09(0.39-3.06) |
| PTSD | order | Gastranaerophilales | Weighted median | 0.0414 | 0.1799 | 0.8180 | 1.04(0.73-1.48) |
| PTSD | order | Gastranaerophilales | IVW | 0.1598 | 0.1646 | 0.3317 | 1.17(0.85-1.62) |
| PTSD | order | Gastranaerophilales | Simple mode | 0.2874 | 0.2859 | 0.3441 | 1.33(0.76-2.33) |
| PTSD | order | Gastranaerophilales | Weighted mode | 0.2195 | 0.2698 | 0.4394 | 1.25(0.73-2.11) |
| PTSD | order | Lactobacillales | MR Egger | 0.1951 | 0.4775 | 0.6894 | 1.22(0.48-3.10) |
| PTSD | order | Lactobacillales | Weighted median | 0.0356 | 0.2228 | 0.8729 | 1.04(0.67-1.60) |
| PTSD | order | Lactobacillales | IVW | 0.2182 | 0.1770 | 0.2176 | 1.24(0.88-1.76) |
| PTSD | order | Lactobacillales | Simple mode | 0.0094 | 0.3495 | 0.9789 | 1.01(0.51-2.00) |
| PTSD | order | Lactobacillales | Weighted mode | -0.0068 | 0.3060 | 0.9825 | 0.99(0.55-1.81) |
| PTSD | order | Methanobacteriales | MR Egger | -0.1394 | 0.4817 | 0.7806 | 0.87(0.34-2.24) |
| PTSD | order | Methanobacteriales | Weighted median | 0.2363 | 0.1503 | 0.1159 | 1.27(0.94-1.70) |
| PTSD | order | Methanobacteriales | IVW | 0.1636 | 0.1177 | 0.1648 | 1.18(0.94-1.48) |
| PTSD | order | Methanobacteriales | Simple mode | 0.3466 | 0.2086 | 0.1352 | 1.41(0.94-2.13) |
| PTSD | order | Methanobacteriales | Weighted mode | 0.3119 | 0.1916 | 0.1421 | 1.37(0.94-1.99) |
| PTSD | order | MollicutesRF9 | MR Egger | 0.4739 | 0.4307 | 0.2969 | 1.61(0.69-3.74) |
| PTSD | order | MollicutesRF9 | Weighted median | 0.0808 | 0.1906 | 0.6714 | 1.08(0.75-1.58) |
| PTSD | order | MollicutesRF9 | IVW | 0.0737 | 0.1416 | 0.6027 | 1.08(0.82-1.42) |
| PTSD | order | MollicutesRF9 | Simple mode | 0.1408 | 0.3155 | 0.6641 | 1.15(0.62-2.14) |
| PTSD | order | MollicutesRF9 | Weighted mode | 0.1104 | 0.2833 | 0.7042 | 1.12(0.64-1.95) |
| PTSD | order | NB1n | MR Egger | -0.0044 | 0.4067 | 0.9917 | 1.00(0.45-2.21) |
| PTSD | order | NB1n | Weighted median | -0.0350 | 0.1318 | 0.7907 | 0.97(0.75-1.25) |
| PTSD | order | NB1n | IVW | 0.0431 | 0.1000 | 0.6662 | 1.04(0.86-1.27) |
| PTSD | order | NB1n | Simple mode | -0.0605 | 0.2081 | 0.7767 | 0.94(0.63-1.42) |
| PTSD | order | NB1n | Weighted mode | -0.0605 | 0.1883 | 0.7540 | 0.94(0.65-1.36) |
| PTSD | order | Pasteurellales | MR Egger | -0.0414 | 0.2628 | 0.8776 | 0.96(0.57-1.61) |
| PTSD | order | Pasteurellales | Weighted median | 0.0982 | 0.1773 | 0.5795 | 1.10(0.78-1.56) |
| PTSD | order | Pasteurellales | IVW | 0.1278 | 0.1200 | 0.2868 | 1.14(0.90-1.44) |
| PTSD | order | Pasteurellales | Simple mode | 0.1602 | 0.3106 | 0.6154 | 1.17(0.64-2.16) |
| PTSD | order | Pasteurellales | Weighted mode | 0.0960 | 0.3500 | 0.7885 | 1.10(0.55-2.19) |
| PTSD | order | Rhodospirillales | MR Egger | -0.1375 | 0.4685 | 0.7747 | 0.87(0.35-2.18) |
| PTSD | order | Rhodospirillales | Weighted median | 0.0293 | 0.1640 | 0.8582 | 1.03(0.75-1.42) |
| PTSD | order | Rhodospirillales | IVW | 0.0448 | 0.1201 | 0.7090 | 1.05(0.83-1.32) |
| PTSD | order | Rhodospirillales | Simple mode | 0.0796 | 0.2751 | 0.7773 | 1.08(0.63-1.86) |
| PTSD | order | Rhodospirillales | Weighted mode | 0.1412 | 0.2587 | 0.5953 | 1.15(0.69-1.91) |
| PTSD | order | Selenomonadales | MR Egger | -0.6410 | 0.9307 | 0.5066 | 0.53(0.09-3.26) |
| PTSD | order | Selenomonadales | Weighted median | -0.1897 | 0.2805 | 0.4987 | 0.83(0.48-1.43) |
| PTSD | order | Selenomonadales | IVW | -0.1703 | 0.2597 | 0.5119 | 0.84(0.51-1.40) |
| PTSD | order | Selenomonadales | Simple mode | 0.0852 | 0.5400 | 0.8774 | 1.09(0.38-3.14) |
| PTSD | order | Selenomonadales | Weighted mode | -0.3379 | 0.4663 | 0.4838 | 0.71(0.29-1.78) |
| PTSD | order | Verrucomicrobiales | MR Egger | 0.1310 | 0.5332 | 0.8109 | 1.14(0.40-3.24) |
| PTSD | order | Verrucomicrobiales | Weighted median | 0.0339 | 0.2069 | 0.8700 | 1.03(0.69-1.55) |
| PTSD | order | Verrucomicrobiales | IVW | 0.1481 | 0.1497 | 0.3226 | 1.16(0.86-1.56) |
| PTSD | order | Verrucomicrobiales | Simple mode | 0.0268 | 0.3597 | 0.9419 | 1.03(0.51-2.08) |
| PTSD | order | Verrucomicrobiales | Weighted mode | 0.0268 | 0.3114 | 0.9330 | 1.03(0.56-1.89) |
| PTSD | order | Victivallales | MR Egger | -0.0100 | 0.4691 | 0.9836 | 0.99(0.39-2.48) |
| PTSD | order | Victivallales | Weighted median | 0.0060 | 0.1576 | 0.9698 | 1.01(0.74-1.37) |
| PTSD | order | Victivallales | IVW | 0.0365 | 0.1219 | 0.7645 | 1.04(0.82-1.32) |
| PTSD | order | Victivallales | Simple mode | -0.2617 | 0.3117 | 0.4289 | 0.77(0.42-1.42) |
| PTSD | order | Victivallales | Weighted mode | -0.2657 | 0.2997 | 0.4048 | 0.77(0.43-1.38) |
| PTSD | phylum | Actinobacteria | MR Egger | 0.1610 | 0.9195 | 0.8639 | 1.17(0.19-7.12) |
| PTSD | phylum | Actinobacteria | Weighted median | 0.1772 | 0.2621 | 0.4989 | 1.19(0.71-2.00) |
| PTSD | phylum | Actinobacteria | IVW | -0.0042 | 0.2131 | 0.9843 | 1.00(0.66-1.51) |
| PTSD | phylum | Actinobacteria | Simple mode | 0.1822 | 0.4652 | 0.7017 | 1.20(0.48-2.99) |
| PTSD | phylum | Actinobacteria | Weighted mode | 0.1822 | 0.3290 | 0.5892 | 1.20(0.63-2.29) |
| PTSD | phylum | Bacteroidetes | MR Egger | -0.6506 | 0.4373 | 0.1751 | 0.52(0.22-1.23) |
| PTSD | phylum | Bacteroidetes | Weighted median | -0.0135 | 0.2687 | 0.9600 | 0.99(0.58-1.67) |
| PTSD | phylum | Bacteroidetes | IVW | -0.1071 | 0.1996 | 0.5916 | 0.90(0.61-1.33) |
| PTSD | phylum | Bacteroidetes | Simple mode | 0.1353 | 0.4802 | 0.7845 | 1.14(0.45-2.93) |
| PTSD | phylum | Bacteroidetes | Weighted mode | 0.1074 | 0.4637 | 0.8220 | 1.11(0.45-2.76) |
| PTSD | phylum | Cyanobacteria | MR Egger | 0.8063 | 0.6046 | 0.2307 | 2.24(0.68-7.33) |
| PTSD | phylum | Cyanobacteria | Weighted median | 0.2621 | 0.2074 | 0.2063 | 1.30(0.87-1.95) |
| PTSD | phylum | Cyanobacteria | IVW | 0.1497 | 0.1740 | 0.3895 | 1.16(0.83-1.63) |
| PTSD | phylum | Cyanobacteria | Simple mode | 0.3787 | 0.3558 | 0.3225 | 1.46(0.73-2.93) |
| PTSD | phylum | Cyanobacteria | Weighted mode | 0.4029 | 0.3070 | 0.2308 | 1.50(0.82-2.73) |
| PTSD | phylum | Euryarchaeota | MR Egger | 0.6485 | 0.4073 | 0.1458 | 1.91(0.86-4.25) |
| PTSD | phylum | Euryarchaeota | Weighted median | 0.0546 | 0.1201 | 0.6492 | 1.06(0.83-1.34) |
| PTSD | phylum | Euryarchaeota | IVW | 0.0479 | 0.0915 | 0.6007 | 1.05(0.88-1.26) |
| PTSD | phylum | Euryarchaeota | Simple mode | -0.1774 | 0.2200 | 0.4388 | 0.84(0.54-1.29) |
| PTSD | phylum | Euryarchaeota | Weighted mode | 0.2353 | 0.2069 | 0.2820 | 1.27(0.84-1.90) |
| PTSD | phylum | Firmicutes | MR Egger | -0.4486 | 0.4249 | 0.3118 | 0.64(0.28-1.47) |
| PTSD | phylum | Firmicutes | Weighted median | -0.2848 | 0.2172 | 0.1897 | 0.75(0.49-1.15) |
| PTSD | phylum | Firmicutes | IVW | -0.1340 | 0.1621 | 0.4084 | 0.87(0.64-1.20) |
| PTSD | phylum | Firmicutes | Simple mode | -0.3332 | 0.3608 | 0.3726 | 0.72(0.35-1.45) |
| PTSD | phylum | Firmicutes | Weighted mode | -0.3332 | 0.2889 | 0.2696 | 0.72(0.41-1.26) |
| PTSD | phylum | Lentisphaerae | MR Egger | 0.0483 | 0.4756 | 0.9220 | 1.05(0.41-2.67) |
| PTSD | phylum | Lentisphaerae | Weighted median | 0.2011 | 0.1583 | 0.2039 | 1.22(0.90-1.67) |
| PTSD | phylum | Lentisphaerae | IVW | 0.0822 | 0.1165 | 0.4808 | 1.09(0.86-1.36) |
| PTSD | phylum | Lentisphaerae | Simple mode | 0.3731 | 0.2936 | 0.2394 | 1.45(0.82-2.58) |
| PTSD | phylum | Lentisphaerae | Weighted mode | 0.3731 | 0.3007 | 0.2498 | 1.45(0.81-2.62) |
| PTSD | phylum | Proteobacteria | MR Egger | 0.0522 | 0.5496 | 0.9262 | 1.05(0.36-3.09) |
| PTSD | phylum | Proteobacteria | Weighted median | 0.1613 | 0.2471 | 0.5139 | 1.18(0.72-1.91) |
| PTSD | phylum | Proteobacteria | IVW | 0.1545 | 0.1895 | 0.4151 | 1.17(0.80-1.69) |
| PTSD | phylum | Proteobacteria | Simple mode | 0.3230 | 0.3756 | 0.4082 | 1.38(0.66-2.88) |
| PTSD | phylum | Proteobacteria | Weighted mode | 0.1927 | 0.3239 | 0.5640 | 1.21(0.64-2.29) |
| PTSD | phylum | Tenericutes | MR Egger | -0.4070 | 0.5024 | 0.4368 | 0.67(0.25-1.78) |
| PTSD | phylum | Tenericutes | Weighted median | 0.0309 | 0.1989 | 0.8767 | 1.03(0.70-1.52) |
| PTSD | phylum | Tenericutes | IVW | 0.0675 | 0.1480 | 0.6483 | 1.07(0.80-1.43) |
| PTSD | phylum | Tenericutes | Simple mode | -0.1871 | 0.2960 | 0.5403 | 0.83(0.46-1.48) |
| PTSD | phylum | Tenericutes | Weighted mode | -0.0811 | 0.2826 | 0.7795 | 0.92(0.53-1.60) |
| PTSD | phylum | Verrucomicrobia | MR Egger | -0.4336 | 0.4705 | 0.3785 | 0.65(0.26-1.63) |
| PTSD | phylum | Verrucomicrobia | Weighted median | -0.1150 | 0.2161 | 0.5946 | 0.89(0.58-1.36) |
| PTSD | phylum | Verrucomicrobia | IVW | -0.1790 | 0.1735 | 0.3022 | 0.84(0.60-1.17) |
| PTSD | phylum | Verrucomicrobia | Simple mode | -0.1507 | 0.3672 | 0.6895 | 0.86(0.42-1.77) |
| PTSD | phylum | Verrucomicrobia | Weighted mode | -0.1676 | 0.2814 | 0.5636 | 0.85(0.49-1.47) |

SNP, single nucleotide polymorphism; OR, odds ratio; CI, confidential interval, MR, mendelian randomization; PTSD, post-traumatic stress disorder

**Table S5** The MR-Egger and Cochran’s Q test of gut microbiome on PTSD in FinnGen datasets in the MR analysis

| Exposure | Outcome | Methods | *P*-Egger intercept | Egger_intercept | Cochran’s | Cochran’s *P* |
| --- | --- | --- | --- | --- | --- | --- |
|  | **class** |  |  |  |  |  |
| PTSD | Bacilli | IVW |  |  | 16.12 | 0.5157 |
|  |  | MR-Egger | 0.8756 | 0.0047 | 16.09 | 0.4467 |
|  | **genus** |  |  |  |  |  |
| PTSD | Eubacteriumfissicatenagroup | IVW |  |  | 6.52 | 0.5896 |
|  |  | MR-Egger | 0.5837 | 0.0415 | 6.19 | 0.5182 |
| PTSD | Ruminococcusgnavusgroup | IVW |  |  | 7.24 | 0.7026 |
|  |  | MR-Egger | 21.79 | 21.79 | 6.97 | 0.6407 |
| PTSD | Butyrivibrio | IVW |  |  | 9.94 | 0.6988 |
|  |  | MR-Egger | 0.2265 | 0.0572 | 9.94 | 0.6988 |
| PTSD | Dorea | IVW |  |  | 6.87 | 0.6505 |
|  |  | MR-Egger | 0.8625 | 0.0068 | 6.84 | 0.5541 |
| PTSD | Eggerthella | IVW |  |  | 4.66 | 0.7930 |
|  |  | MR-Egger | 0.5927 | 0.0325 | 4.35 | 0.7389 |
| PTSD | Haemophilus | IVW |  |  | 5.85 | 0.6636 |
|  |  | MR-Egger | 0.8193 | 0.0084 | 5.80 | 0.5636 |
| PTSD | Sellimonas | IVW |  |  | ss | 0.5406 |
|  |  | MR-Egger | 0.8849 | -0.0103 | 6.94 | 0.4351 |

MR, mendelian randomization; OR, odds ratio; CI; confidential interval; PTSD, post-traumatic stress disorder

**Table S6** Single-nucleotide polymorphisms used as instrumental variables for Freeze 2 datasets in the reverse MR analysis

| **SNP** | **effect allele** | **other allele** | **eaf** | **SE** | **P** | **beta** |
| --- | --- | --- | --- | --- | --- | --- |
| rs34517852 | A | T | 0.3560 | 0.0185 | 3.16E-09 | 0.1095 |
| rs9364611 | T | C | 0.1245 | 0.0226 | 4.36E-08 | -0.1239 |
| rs57753395 | A | G | 0.0179 | 0.0705 | 9.13E-08 | 0.3764 |
| rs17108326 | A | G | 0.8505 | 0.0209 | 3.91E-07 | 0.1060 |
| rs36127550 | T | G | 0.1705 | 0.0204 | 4.63E-07 | -0.1027 |
| rs4078035 | T | G | 0.0526 | 0.0354 | 1.10E-06 | 0.1726 |
| rs62583551 | A | G | 0.2076 | 0.0199 | 1.22E-06 | -0.0966 |
| rs140928208 | A | G | 0.0158 | 0.0929 | 1.46E-06 | 0.4474 |
| rs149509653 | A | G | 0.1997 | 0.0189 | 1.74E-06 | 0.0905 |
| rs1444764 | A | G | 0.2912 | 0.0164 | 1.90E-06 | 0.0781 |
| rs2163050 | A | G | 0.1458 | 0.0211 | 2.12E-06 | 0.1000 |
| rs111771848 | G | GA | 0.9823 | 0.0750 | 2.18E-06 | -0.3552 |
| rs1136201 | A | G | 0.7539 | 0.0171 | 2.34E-06 | -0.0805 |
| rs80074987 | T | G | 0.0340 | 0.0507 | 2.64E-06 | 0.2381 |
| rs12528870 | A | G | 0.0458 | 0.0375 | 2.69E-06 | 0.1760 |
| rs139591016 | T | C | 0.9759 | 0.0532 | 2.86E-06 | -0.2489 |
| rs12706983 | T | G | 0.7206 | 0.0192 | 3.01E-06 | -0.0896 |
| rs35334143 | C | G | 0.7521 | 0.0171 | 3.15E-06 | -0.0797 |
| rs77537694 | A | G | 0.9156 | 0.0280 | 3.25E-06 | 0.1302 |
| rs73154700 | A | G | 0.0861 | 0.0272 | 3.45E-06 | 0.1260 |
| rs17041470 | A | G | 0.9827 | 0.0644 | 3.66E-06 | -0.2981 |
| rs78608260 | A | G | 0.9818 | 0.0694 | 3.71E-06 | -0.3214 |
| rs7807190 | A | G | 0.7859 | 0.0181 | 4.04E-06 | 0.0834 |
| rs114981056 | A | T | 0.9659 | 0.0441 | 5.10E-06 | -0.2011 |
| rs34501457 | G | GA | 0.7308 | 0.0182 | 5.19E-06 | 0.0827 |
| rs72914051 | T | G | 0.9106 | 0.0292 | 5.61E-06 | -0.1327 |
| rs117453028 | T | C | 0.0206 | 0.0630 | 5.81E-06 | 0.2858 |
| rs114514029 | T | G | 0.9678 | 0.0451 | 5.84E-06 | -0.2042 |
| rs142333870 | A | T | 0.9718 | 0.0491 | 6.09E-06 | -0.2222 |
| rs10281893 | A | G | 0.3993 | 0.0152 | 6.46E-06 | -0.0684 |
| rs70961877 | CT | C | 0.5162 | 0.0163 | 6.50E-06 | -0.0733 |
| rs72657988 | T | G | 0.0823 | 0.0327 | 6.61E-06 | 0.1472 |
| rs74901405 | T | G | 0.1142 | 0.0261 | 6.81E-06 | 0.1174 |
| rs146676625 | A | G | 0.0240 | 0.0518 | 6.94E-06 | 0.2330 |
| rs1486086 | C | G | 0.1552 | 0.0212 | 7.11E-06 | -0.0952 |
| rs33516 | A | T | 0.3110 | 0.0161 | 7.34E-06 | 0.0723 |
| rs184613502 | A | G | 0.0160 | 0.0658 | 7.36E-06 | 0.2949 |
| rs1363508 | A | G | 0.2982 | 0.0161 | 7.39E-06 | -0.0720 |
| rs2293995 | T | C | 0.9571 | 0.0374 | 7.62E-06 | 0.1675 |
| rs79317376 | T | G | 0.0160 | 0.0730 | 8.39E-06 | 0.3252 |
| rs73157523 | T | C | 0.8229 | 0.0195 | 8.65E-06 | -0.0865 |

SNP, single nucleotide polymorphism; MR, mendelian randomization; PTSD, post-traumatic stress disorder

**Table S7** Single-nucleotide polymorphisms used as instrumental variables for FinnGen datasets in the reverse MR analysis

| **SNP** | **ref** | **alt** | **pval** | **beta** | **se** | **eaf** |
| --- | --- | --- | --- | --- | --- | --- |
| rs61751012 | G | A | 2.41E-07 | 0.7040 | 0.1363 | 0.0092 |
| rs2192513 | G | T | 5.90E-06 | 0.2442 | 0.0539 | 0.0881 |
| rs4851073 | C | T | 7.63E-06 | -0.2047 | 0.0457 | 0.1628 |
| rs112719448 | T | TAC | 8.54E-06 | 0.2554 | 0.0574 | 0.8995 |
| rs6442896 | A | T | 3.54E-06 | 1.8172 | 0.3919 | 0.0007 |
| rs6855181 | T | C | 1.51E-06 | -0.1578 | 0.0328 | 0.6071 |
| rs76209611 | T | G | 2.31E-06 | 0.2786 | 0.0590 | 0.0712 |
| rs72732181 | A | G | 3.72E-06 | -0.5203 | 0.1125 | 0.0298 |
| rs9292906 | A | C | 6.84E-06 | 0.1490 | 0.0331 | 0.3649 |
| rs10060769 | C | T | 5.77E-06 | 0.1566 | 0.0345 | 0.3062 |
| rs148642988 | C | T | 8.34E-06 | 0.7784 | 0.1747 | 0.0057 |
| rs118093140 | T | G | 7.62E-06 | 0.3004 | 0.0671 | 0.0563 |
| rs7867845 | C | G | 7.44E-06 | -0.2230 | 0.0498 | 0.1325 |
| rs9423643 | C | T | 7.69E-06 | -0.1635 | 0.0365 | 0.2886 |
| rs59774618 | T | TAA | 3.76E-06 | 3.4139 | 0.7383 | 0.0001 |
| rs9971308 | A | G | 4.20E-06 | 0.1863 | 0.0405 | 0.7857 |
| rs4548693 | A | C | 7.36E-06 | -0.1633 | 0.0364 | 0.7512 |
| rs149884297 | G | A | 7.47E-06 | 0.9730 | 0.2172 | 0.0034 |
| rs17636116 | C | T | 1.94E-06 | -0.4491 | 0.0944 | 0.0398 |
| rs57729369 | A | G | 5.29E-06 | 0.3087 | 0.0678 | 0.0526 |
| rs6504128 | A | C | 7.44E-06 | -0.3850 | 0.0859 | 0.9693 |
| rs60856912 | G | T | 6.13E-06 | 0.1717 | 0.0380 | 0.2175 |
| rs6021953 | G | A | 3.13E-06 | -0.1746 | 0.0374 | 0.2688 |
| rs367001 | G | T | 1.37E-06 | -0.1573 | 0.0326 | 0.4779 |

SNP, single nucleotide polymorphism; MR, mendelian randomization; PTSD, post-traumatic stress disorder

**Table S8** The MR-Egger and Cochran’s Q test of PTSD on gut microbiome in Freeze 2 datasets in reverse MR analysis

| Exposure | Outcome | Methods | Egger_intercept | *P*-Egger intercept | Cochran’s | Cochran’s *P* |
| --- | --- | --- | --- | --- | --- | --- |
|  | **family** |  |  |  |  |  |
[truncated: 3,478 more chars]
